# Chlorogenic Acid-Rich Fraction from Green Coffee Beans and Its Combination with Paclitaxel Attenuate *Porphyromonas gingivalis* LPS-Stimulated Esophageal Squamous Cell Carcinoma Related to the NF-ĸB and p38 Pathways

**DOI:** 10.3390/ijms27219464

**Published:** 2026-10-23

**Authors:** Atita Panyathep, Khanittha Punturee, Teera Chewonarin

**Affiliations:** 1 School of Medicine, Mae Fah Luang University, Chiang Rai 57100, Thailand; atita.pan@mfu.ac.th; 2 Integrative Natural Therapeutics and Health Innovation Research Group (INTHI), Mae Fah Luang University, Chiang Rai 57100, Thailand; 3 Cancer Research Unit of Associated Medical Sciences (AMS-CRU), Faculties of Associated Medical Sciences, Chiang Mai University, Chiang Mai 50200, Thailand; khanittha.taneyhill@cmu.ac.th; 4 Department of Biochemistry, Faculty of Medicine, Chiang Mai University, Chiang Mai 50200, Thailand

**Keywords:** *Porphyromonas gingivalis*, esophageal squamous cell carcinoma, paclitaxel, lipopolysaccharide, green coffee bean, chlorogenic acid

## Abstract

Esophageal squamous cell carcinoma (ESCC) is linked to *Porphyromonas gingivalis* (*Pg*). *Pg* lipopolysaccharide (LPS-PG) triggers inflammatory responses contributing to cancer progression. Paclitaxel (PTX) is frequently used in combination regimens for ESCC therapy. This study aimed to investigate the role of a chlorogenic acid (CGA)-rich fraction from green coffee bean extract, known as the ethyl acetate-insoluble fraction (EIF), in inflammatory responses and cancer progression in LPS-PG-triggered ESCC (KE5 and T.T cells), as well as its combination with PTX. Both EIF and pure CGA were assessed for their effects on LPS-pretreated ESCC cell lines. Cell survival, adhesion, migration, and invasion assays were performed. Key inflammatory and metastatic mediators were measured using Western blotting, ELISA, and zymography. The study also examined the effects of combining PTX with EIF. Our results showed that LPS-PG stimulated inflammatory responses and malignant phenotypes in ESCC. EIF effectively diminished these effects by involving the NF-κB and p38 MAPK pathways. Furthermore, in T.T cells, the combination of EIF (25 μg/mL) and PTX (3 μg/mL) reduced the expression of cancer- and inflammation-related markers compared with PTX alone, without further reducing cell viability. Therefore, EIF represents a candidate agent, either alone or in combination with PTX, to attenuate *Pg*-driven ESCC progression.

## 1. Introduction

Esophageal cancer (EC) is one of the most prevalent cancers of the upper digestive tract and was one of the top ten causes of cancer-related deaths in 2022, according to GLOBOCAN [1]. For EC, the 5-year survival rate remained below 25% [2]. In general, the most common EC subtype is esophageal squamous cell carcinoma (ESCC) [3]. The high incidence of ESCC is observed in males, the elderly, smokers, alcoholics, and those with genetic predisposition, poor dietary habits, viral infections, and inflammation [3,4]. Microorganisms in this region, particularly *Porphyromonas gingivalis* (*Pg*), have been identified as risk factors for ESCC [5]. *P. gingivalis* is a key bacterial pathogen of periodontitis and has been linked to poor prognosis, inflammatory response, cancer progression, chemoresistance, and mortality in ESCC [6,7,8,9,10,11]. According to prior studies, lipopolysaccharide (LPS-PG), a common virulence factor of *Pg*, contributes to increased inflammatory cytokines, proliferation, radiation resistance, and chemoresistance in several tumor cells [12,13,14,15,16,17]. Normally, LPS-PG is primarily a toll-like receptor 4 (TLR4) agonist and differs in structure and potency compared with other bacterial species [18,19,20]. Although evidence on the effects of LPS-PG on ESCC remains limited, prior research [21,22] has found that LPS-TLR4 binding induces inflammatory responses and promotes cancer progression in ESCC, primarily through the p38 MAPK pathway. In fact, p38 and NF-κB are upregulated and associated with ESCC metastasis, cancer progression, and the inflammatory response. They also regulate numerous downstream proteins, including TNF-α, IL-1β, COX2, MMP2, MMP9, VEGF, uPA, and uPAR [16,23,24]. Consequently, a therapeutic target should be downstream of LPS activity.

The standard treatment of ESCC includes endoscopic resection, chemotherapy, chemoradiotherapy, and surgery, depending on cancer stage and patient condition [25]. Paclitaxel (PTX) is a lipophilic, broad-spectrum chemotherapeutic agent derived from the plant *Taxus baccata*. Previously, several PTX combination regimens were designed to incorporate radiotherapy or chemical agents, such as platinum-based drugs (Cisplatin/Carboplatin) and PD-1 inhibitors. However, these combinations frequently lead to cumulative toxicities, including severe peripheral neuropathy, myelosuppression, and gastrointestinal and mucosal toxicity [26,27,28,29,30,31]. Given that high doses of PTX can affect multiple organs and act as an inflammatory inducer, these limitations must be carefully considered [32,33]. Consequently, natural compounds—such as flavonoids [34,35,36], borneol [37], and Qige San [38]—have gained significant interest and demonstrated success as adjuvants alongside PTX. These plant-derived biocompounds offer distinct therapeutic advantages, notably their low toxicity, high selectivity, reduced side effects, multi-targeting capabilities, and potential to reverse drug resistance [34,39]. Thus, it is interesting to identify new natural compounds that can interact with PTX to enhance its effectiveness while reducing its effective dose. Green coffee bean (GCB) extract from *Coffea canephora* (robusta) possesses an anti-periodontal bacterial effect [40], and our earlier studies established the role of the chlorogenic acid (CGA)-rich fraction derived from GCB in attenuating cancer progression in LPS-PG-stimulated OSCC and inflammatory response in LPS-stimulated colon cancer [41,42]. Building on this, the current study expands this scope to ESCC, another malignancy highly susceptible to *P. gingivalis*. This study provides valuable translational insights by demonstrating that the CGA-rich fraction from GCB, either alone or in combination with PTX, exhibits enhanced efficacy in the LPS-PG-stimulated ESCC model.

## 2. Results

### 2.1. Effects of EIF and CGA on Cell Viability of ESCCs and LPS-PG-Pretreated ESCCs

**Figure 1:**
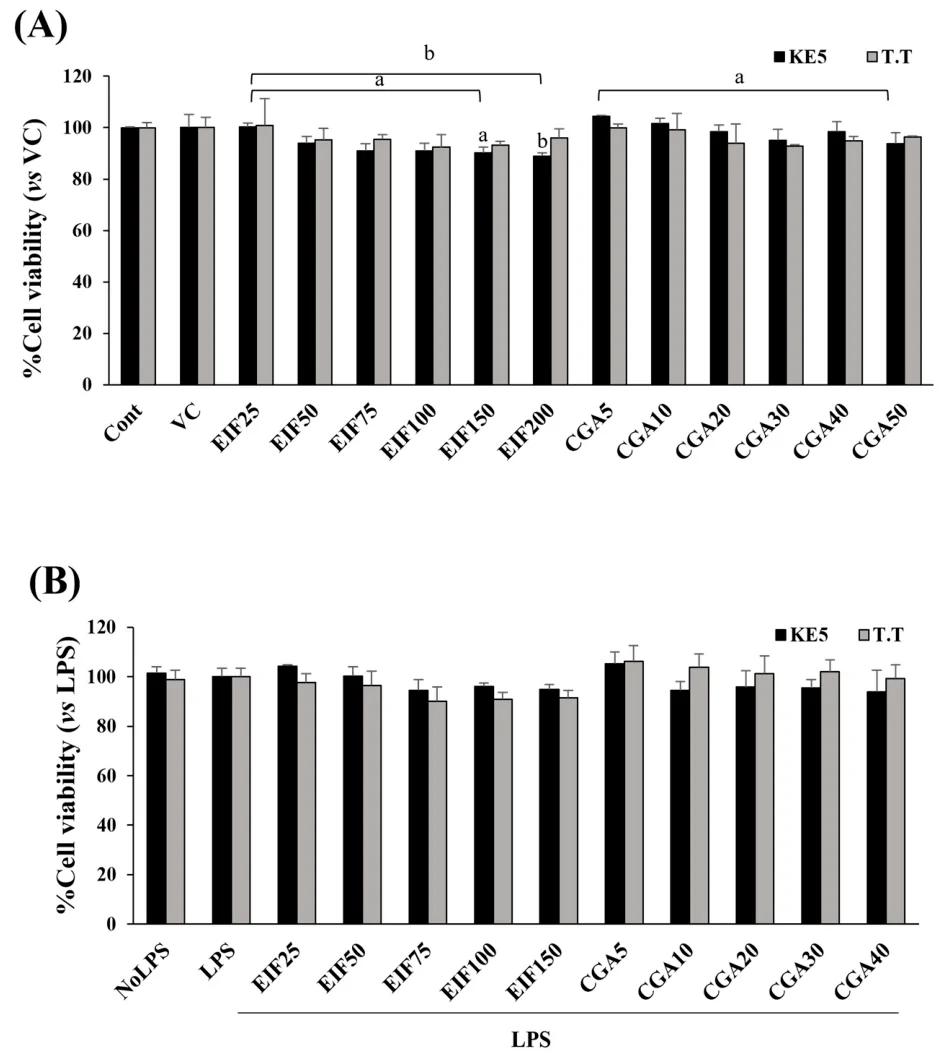
**Effects of EIF and CGA on cell survival of ESCC and LPS-PG-pretreated ESCC cells.** The toxicity of EIF and CGA to KE5 and T.T cells (**A**) and LPS-PG-pretreated KE5 and T.T cells (**B**) was assessed by MTT assay. Significant values (vs. vehicle control or LPS), indicated by letters a and b, are *p* < 0.05 and *p* < 0.01, respectively. (VC = vehicle control, cont = control).

EIF and CGA had low toxicity to ESCC viability at concentrations of 25–200 μg/mL and 5–50 μg/mL, respectively. Although KE5 cells were more sensitive than T.T cells, their viability remained above 85% (Figure 1A). Furthermore, pretreating ESCC cells with 1 μg/mL LPS-PG for two hours did not cause them to proliferate. The tested concentrations of EIF (25–150 μg/mL) and CGA (5–40 μg/mL) were also non-toxic to ESCC cells pretreated with LPS-PG (Figure 1B). Therefore, we selected experimental conditions of EIF 25, 50, and 75 µg/mL, which were evaluated alongside pure CGA at comparable doses of 5, 10, and 20 μg/mL (equivalent to 14.11, 28.22, and 56.45 μM) to match earlier research. Based on our previous HPLC analysis, the calculated CGA content in EIF at concentrations of 25, 50, and 75 μg/mL was approximately 4.4 μg/mL (12.42 µM), 8.8 μg/mL (24.84 µM), and 13.3 μg/mL (37.54 µM), respectively. Although the CGA content in the EIF did not identically match that of the pure CGA control, these doses were sufficient to elucidate the distinct biological effects of CGA as a single compound versus its action within the multi-component mixture of EIF.

### 2.2. Effects of EIF and CGA on the Expression of Mediators-Related to Inflammatory Response in LPS-PG-Pretreated ESCCs

**Figure 2:**
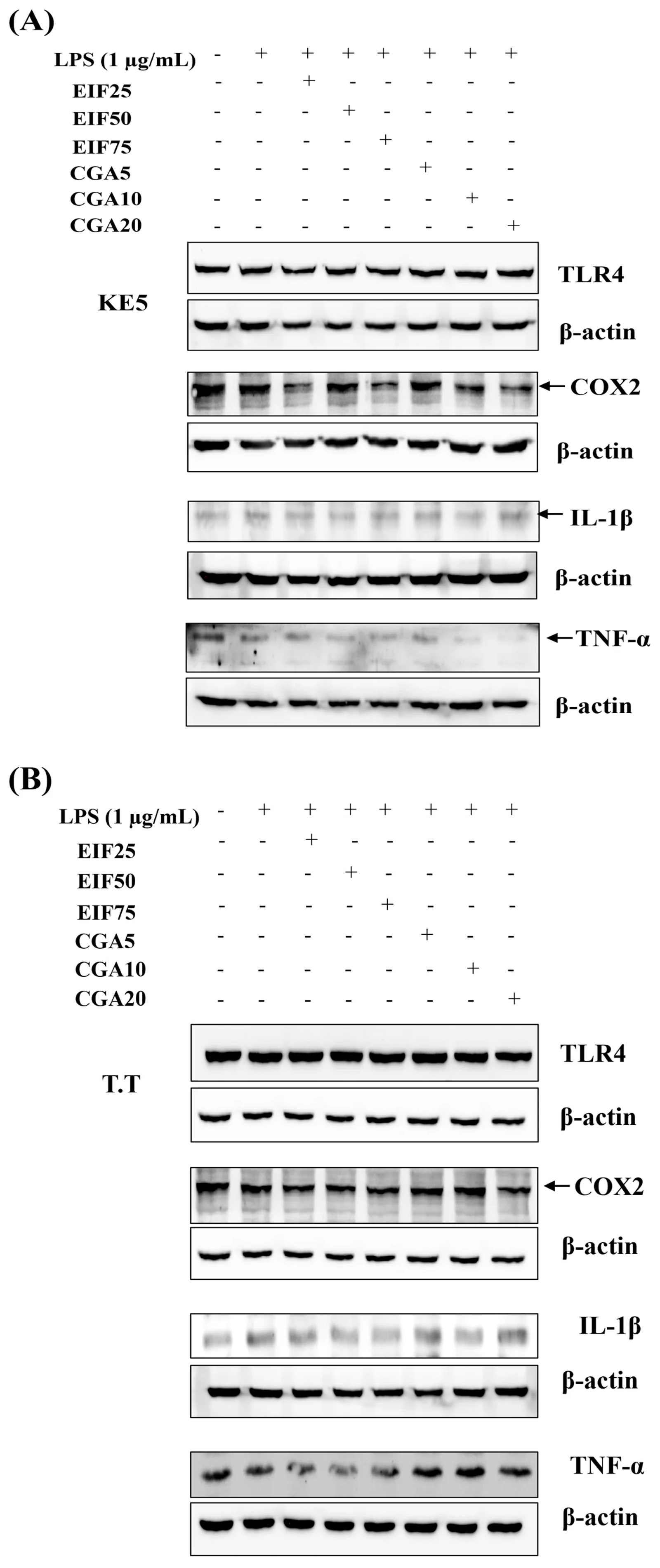

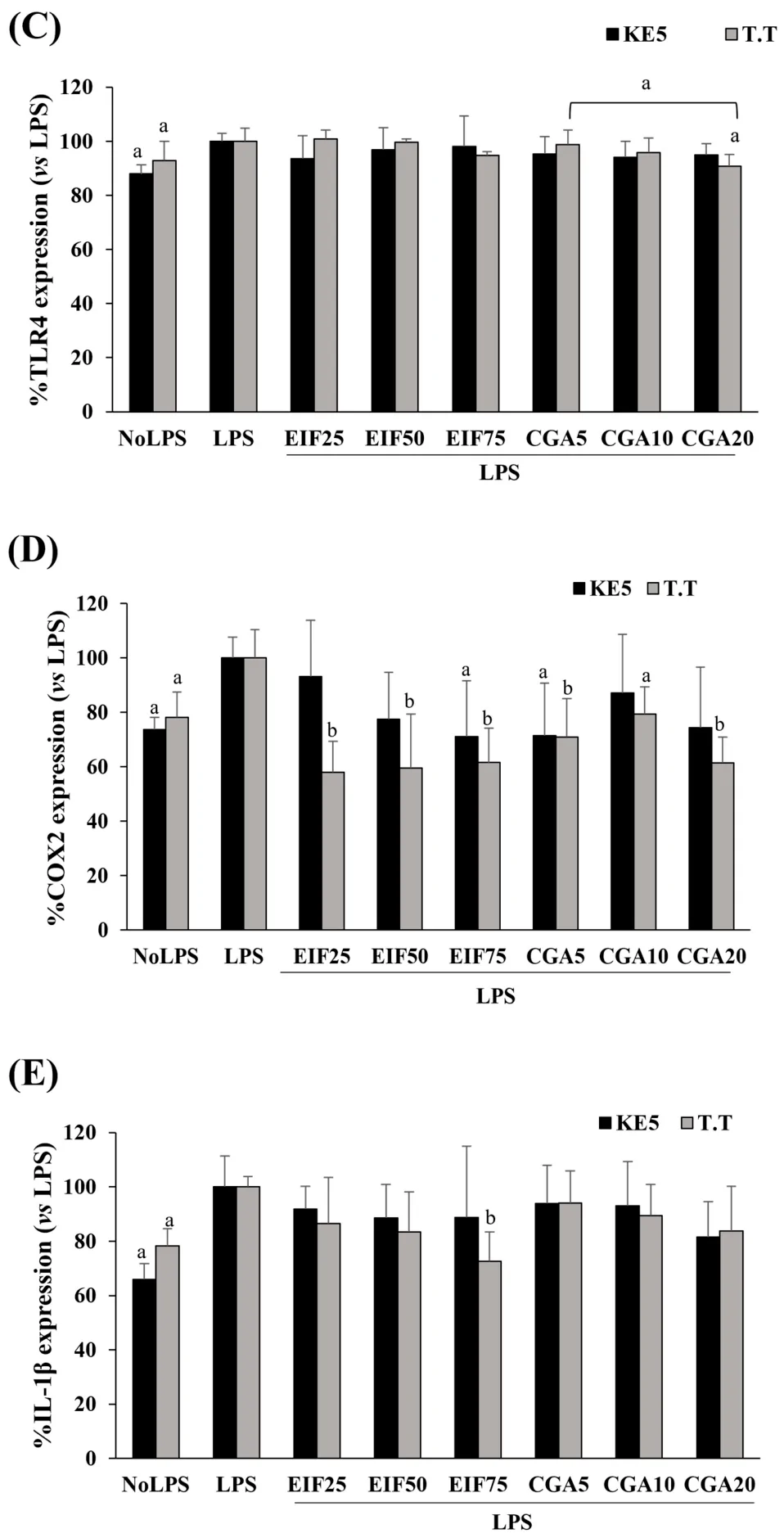

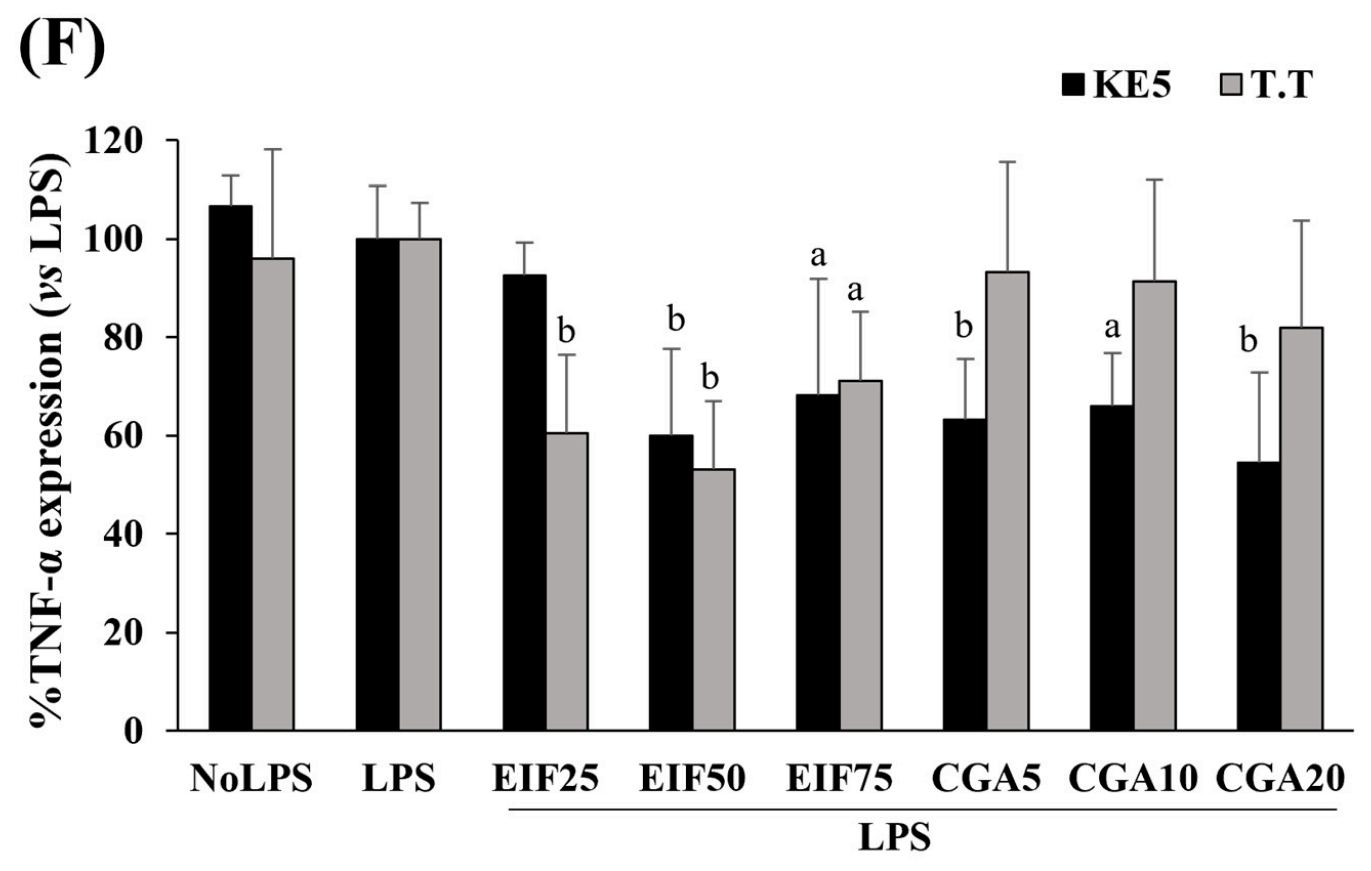
**Effects of EIF and CGA on the expression of mediators-related to inflammatory responses in LPS-PG-pretreated ESCCs.** Several treated cell lysate samples from LPS-PG-pretreated KE5 (**A**) and T.T (**B**) cells were used to analyze TLR4 (**C**), COX2 (**D**), IL-1β (**E**), and TNF-α (**F**) expression using a Western blot assay. Significant values (compared with the LPS control), *p* < 0.05 and *p* < 0.01, are denoted by letters a and b, respectively.

The inflammatory response directly results from LPS action via TLR4 signaling. According to our findings, LPS-PG only slightly increased TLR4 expression in both ESCCs (about 7–13%). Even though this LPS-PG-stimulated TLR4 action was rarely affected by EIF and CGA (Figure 2C), in the case of other downstream inflammatory mediators, LPS-PG specifically increased the production of COX2 (Figure 2D) and IL-1β (Figure 2E) but not TNF-α (Figure 2F) in both ESCCs. Interestingly, EIF selectively decreased these mediators (apart from IL-1β) in KE5 cells but generally inhibited them in T.T cells. Nevertheless, CGA’s inhibitory activity differed and did not correspond to EIF. CGA significantly reduced LPS-PG-stimulated COX2 in T.T cells and KE5 cells (only at 5 μg/mL), as well as TNF-α in KE5 cells. Cell type and dosage appeared to affect the inconsistent CGA activity.

### 2.3. Effects of EIF and CGA on the Expression of Mediators-Related to Cancer Progression in LPS-PG-Pretreated ESCCs

**Figure 3:**
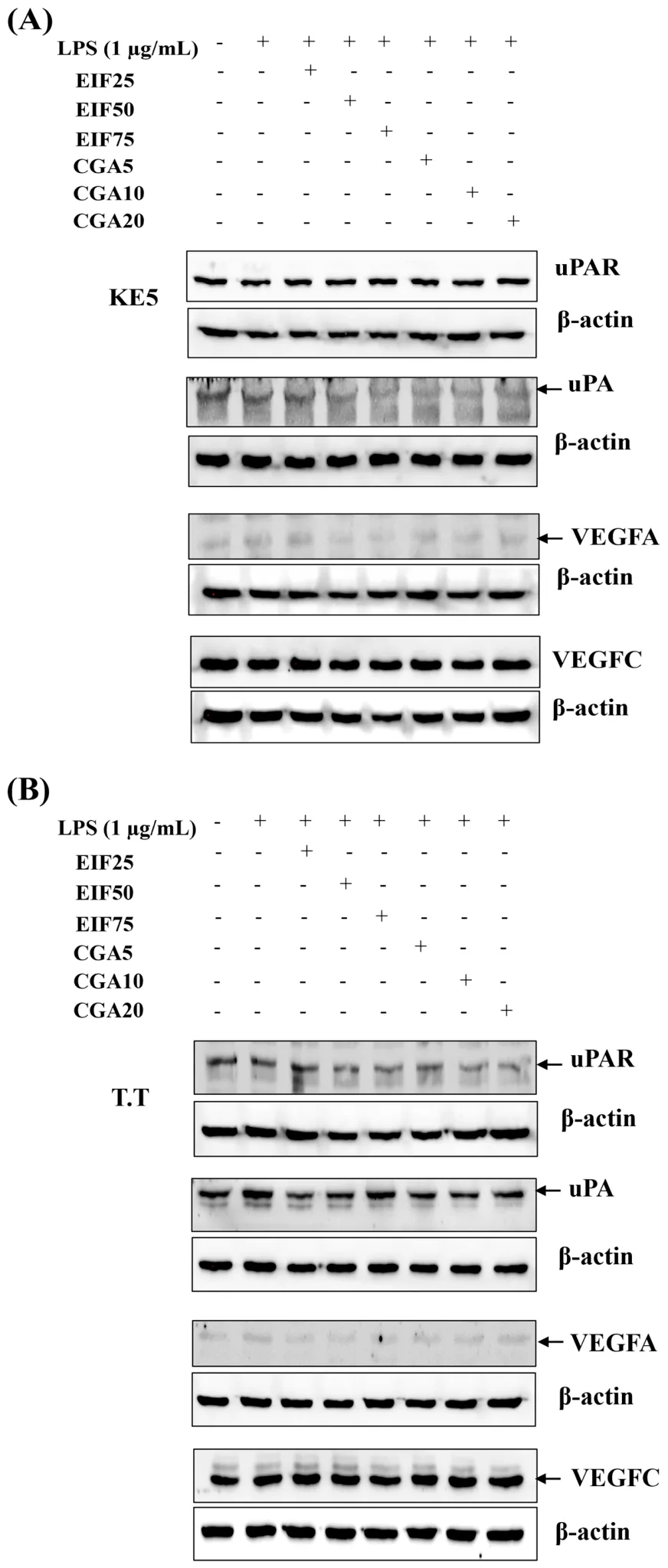

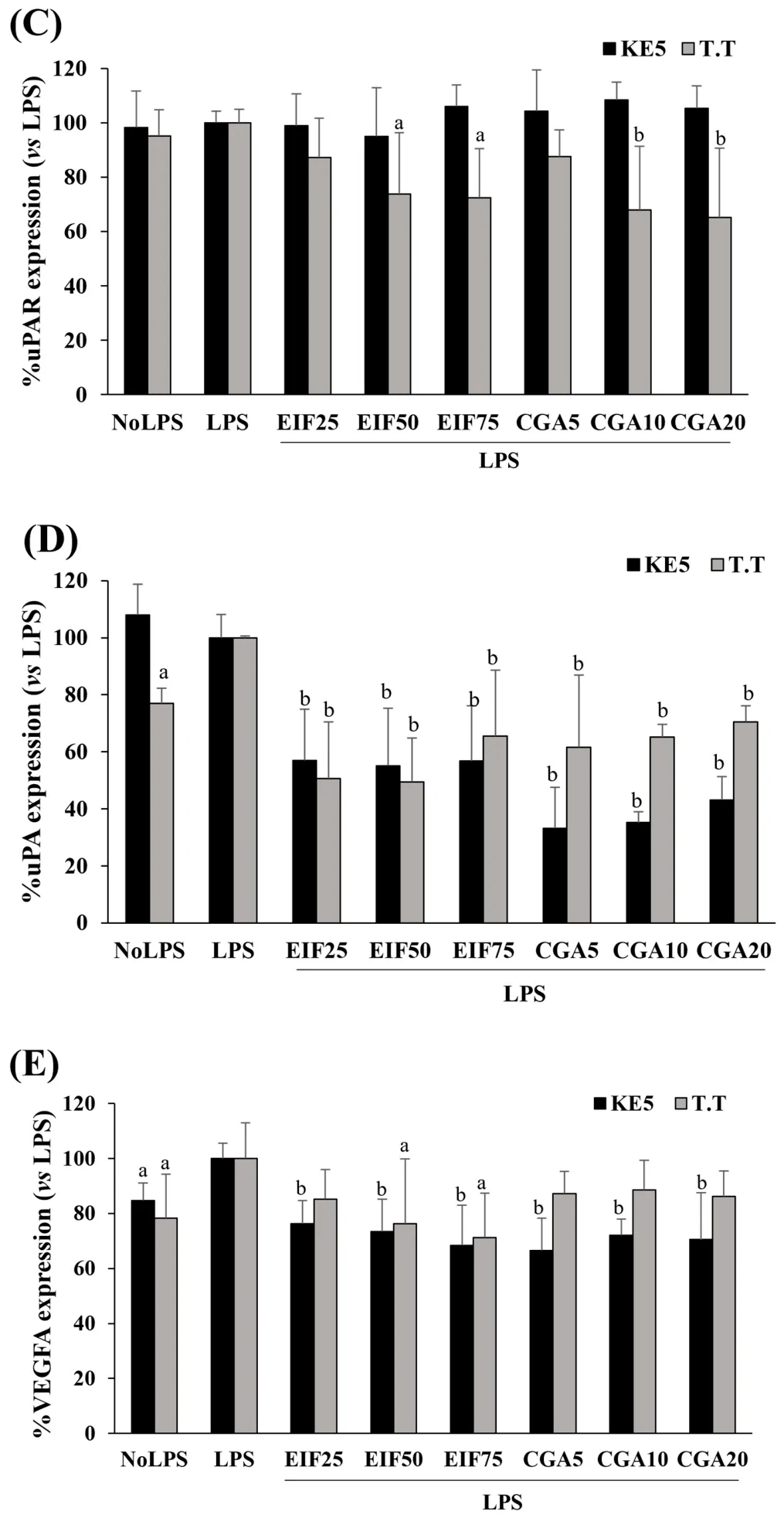

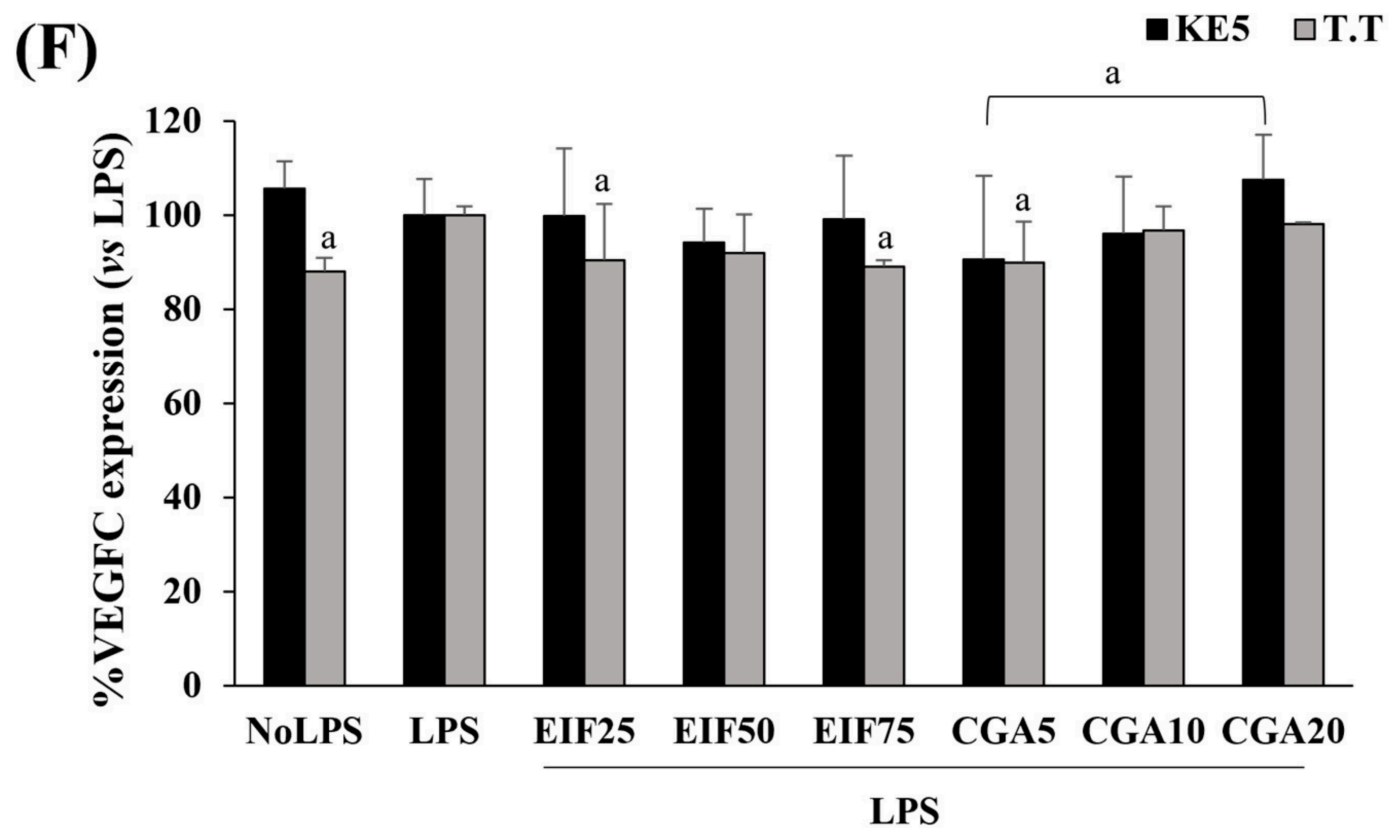
**Effects of EIF and CGA on the expression of mediators-related to cancer progression in LPS-PG-pretreated ESCCs.** Several EIF- and CGA-treated cell lysate samples from LPS-PG-pretreated KE5 (**A**) and T.T (**B**) cells were used to analyze uPAR (**C**), uPA (**D**), VEGFA (**E**), and VEGFC (**F**) expression using a Western blot assay. Significant values (*p* < 0.05 and *p* < 0.01, respectively) are denoted by the letters a and b.

Inflammation-related cancer progression resulting from LPS-PG was also studied. The current findings demonstrated that LPS-PG was more effective in T.T. cells. In T.T. cells, LPS-PG stimulated uPA, VEGFC, and VEGFA; in KE5 cells, only VEGFA was increased. In both ESCC cell lines, LPS-PG did not affect uPAR expression (Figure 3C). In both ESCC cell lines, the inhibitory effects of CGA and EIF differed. EIF and CGA significantly reduced uPA and VEGFA in KE5 cells. EIF, particularly at the high dose of 75 μg/mL, broadly inhibited T.T cells by reducing uPAR, uPA, VEGFA, and VEGFC production. While CGA action on T.T cells was more limited, uPAR and uPA expression were reduced. Intriguingly, CGA exhibited a cell-line-specific selective effect on VEGFC expression. CGA slightly increased VEGFC expression in KE5 cells, particularly at the high dose (20 μg/mL), showing a non-significant increase of about 7% compared with the LPS-alone group (*p* > 0.05). Conversely, a slight but significant reduction of approximately 10% was observed in T.T cells at the low dose of 5 μg/mL CGA (*p* < 0.05) (Figure 3F).

### 2.4. Effects of EIF and CGA on the Secretion and Activity of Mediators-Related to Cancer Progression in LPS-PG-Pretreated ESCCs

**Figure 4:**
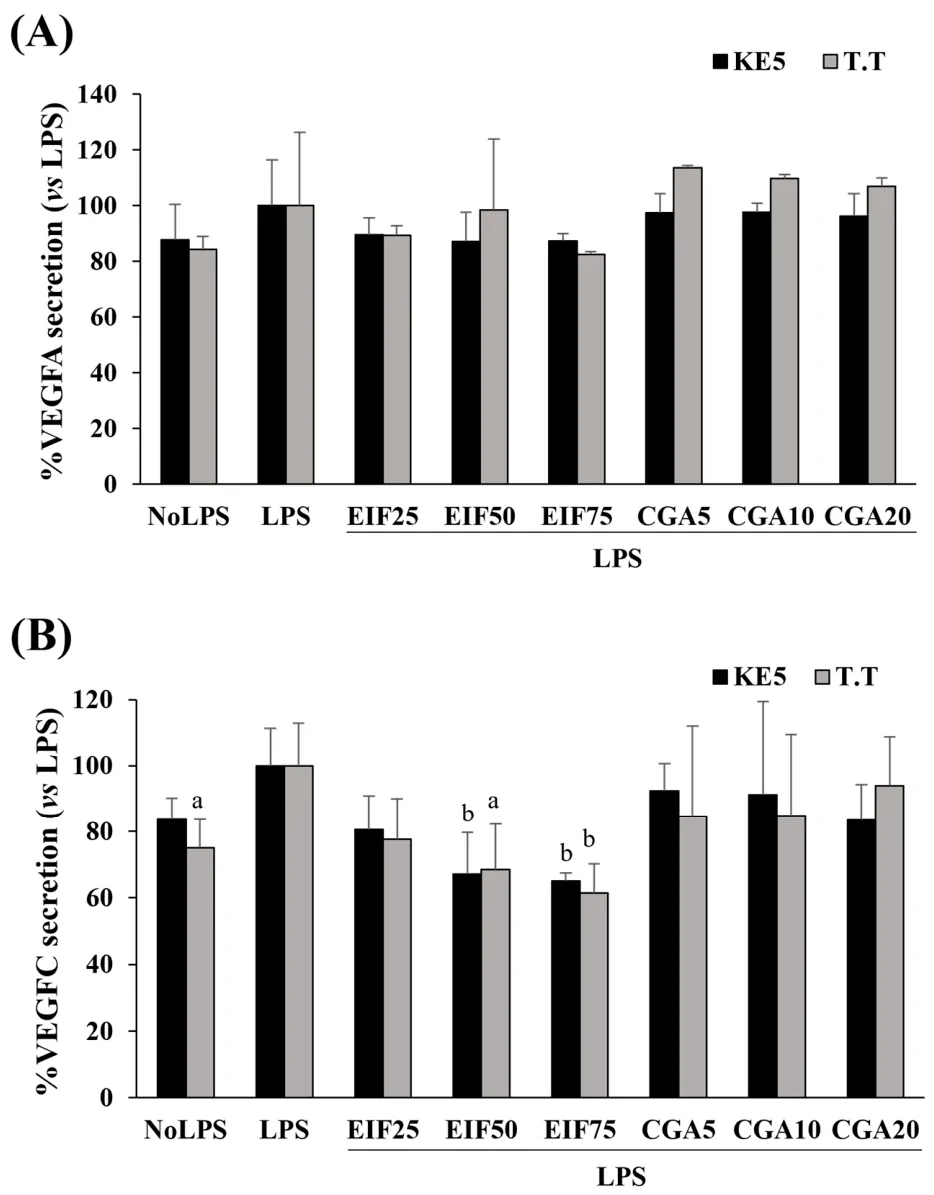

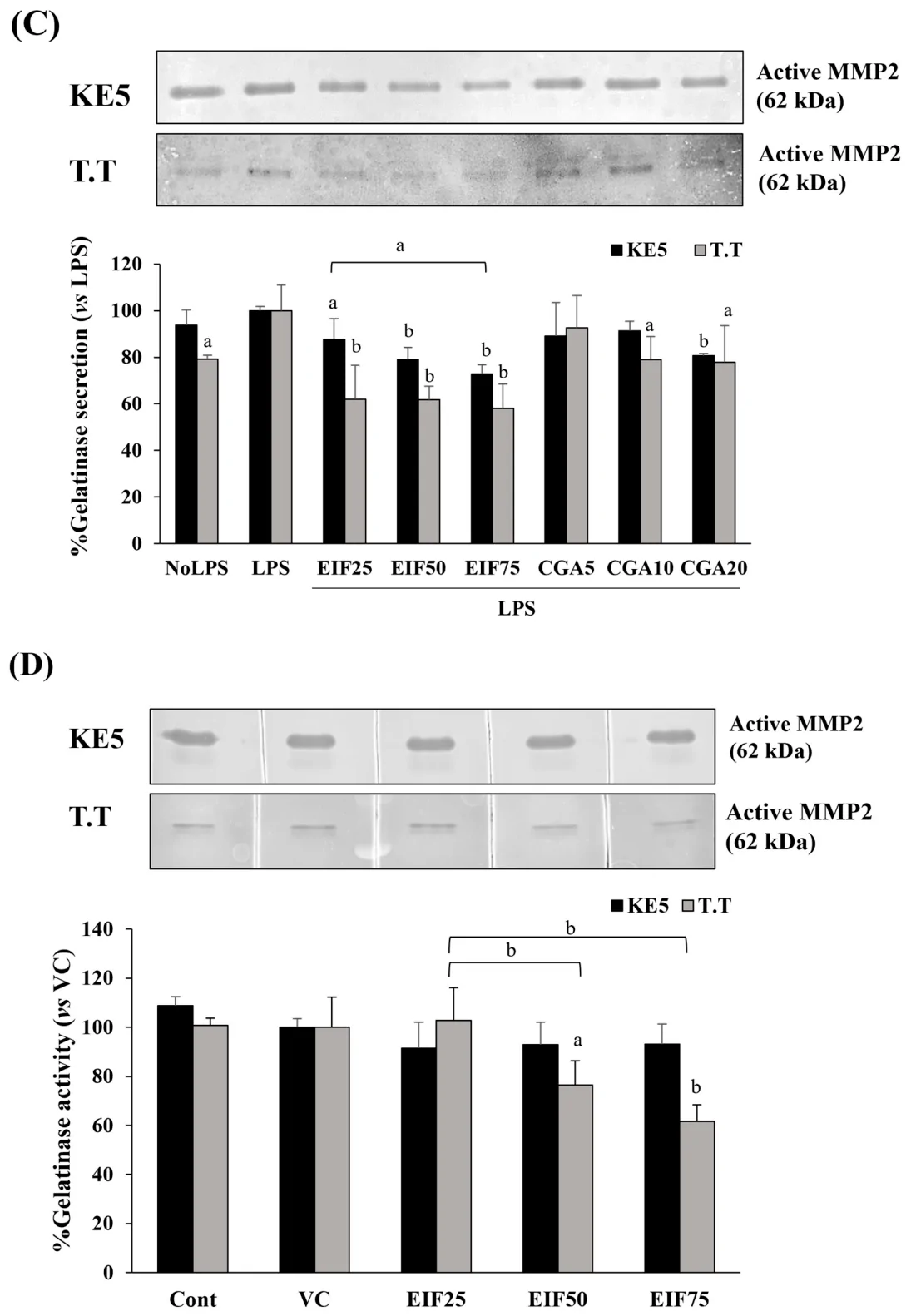

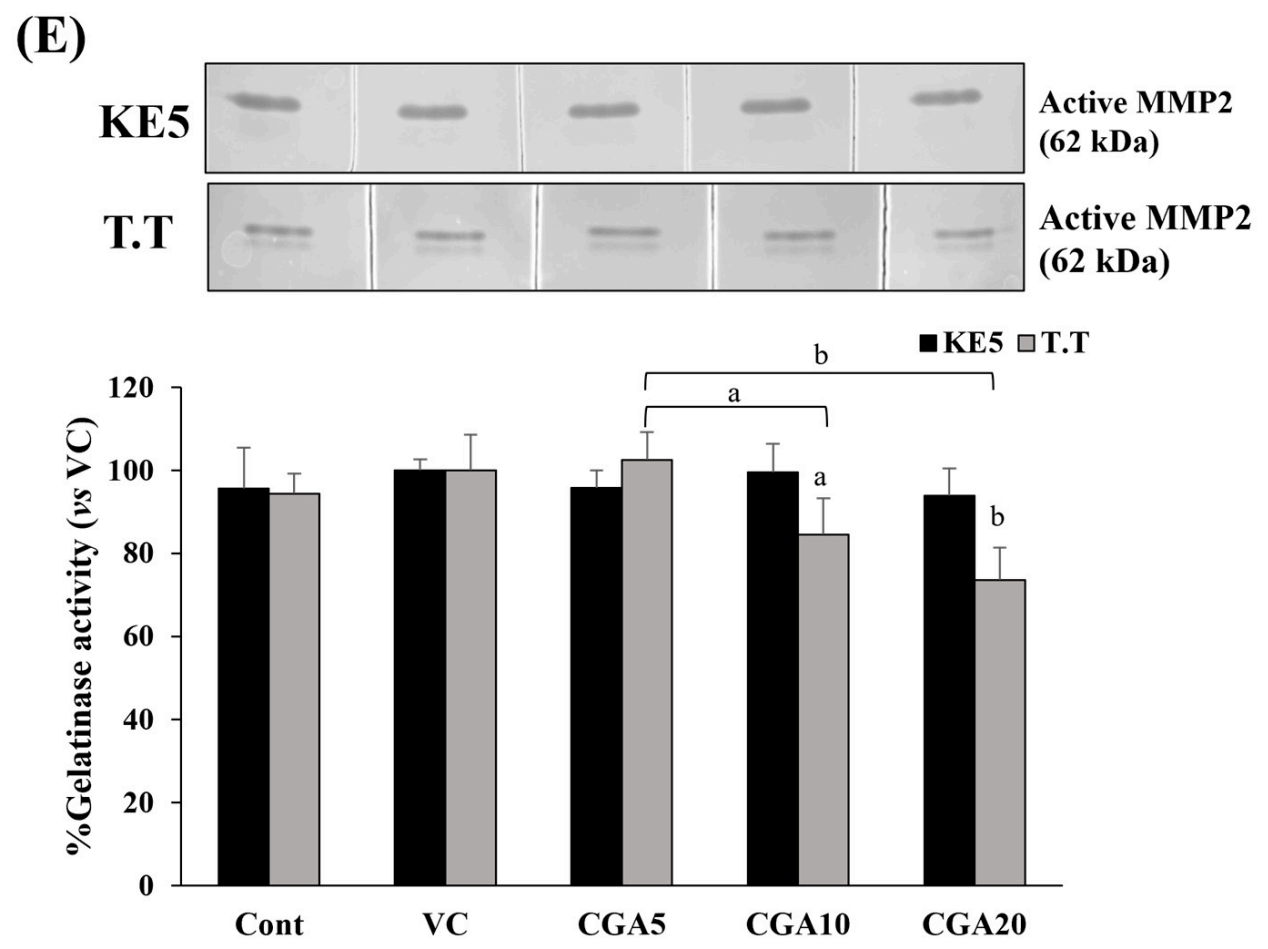
**Effects of EIF and CGA on the secretion and activity of mediators-related to cancer progression in LPS-PG-pretreated ESCCs.** Conditioned media from various treated conditions of LPS-PG-pretreated ESCCs were used to examine the action of EIF and CGA on VEGFA (**A**), VEGFC (**B**), and gelatinase secretion (**C**). Meanwhile, the culture supernatant of LPS-PG-pretreated ESCCs was collected to analyze the role of EIF (**D**) and CGA (**E**) in gelatinase activity. All results were obtained using ELISA and gelatin zymography. Significant differences (*p* < 0.05 and *p* < 0.01) are indicated by the letters ‘a’ and ‘b’, respectively, compared with VC in (**D**,**E**) and the LPS control in (**A**–**C**).

Researchers frequently study VEGF and MMP2 (gelatinase) secretion and MMP2 activity in cancer progression. First, VEGFA secretion was unchanged in either cell line with LPS-PG pretreatment or EIF and CGA treatment (Figure 4A). In contrast, LPS-PG stimulated VEGFC secretion in T.T cells but not in KE5 cells. Thereafter, EIF treatment decreased VEGFC secretion in both LPS-PG-pretreated ESCCs, whereas CGA did not (Figure 4B). Next, zymogram results showed that LPS-PG specifically enhanced MMP2 secretion in T.T cells, but not in KE5 cells. However, EIF and CGA treatment diminished MMP2 secretion in both LPS-PG-pretreated ESCCs (Figure 4C). Regarding MMP2 activity, both EIF (Figure 4D) and CGA (Figure 4E) reduced it only in T.T cells. Additionally, MMP9 was undetectable in both cell types by zymography.

### 2.5. Effects of EIF and CGA on the Activity of Cancer Progression in LPS-PG-Pretreated ESCCs

**Figure 5:**
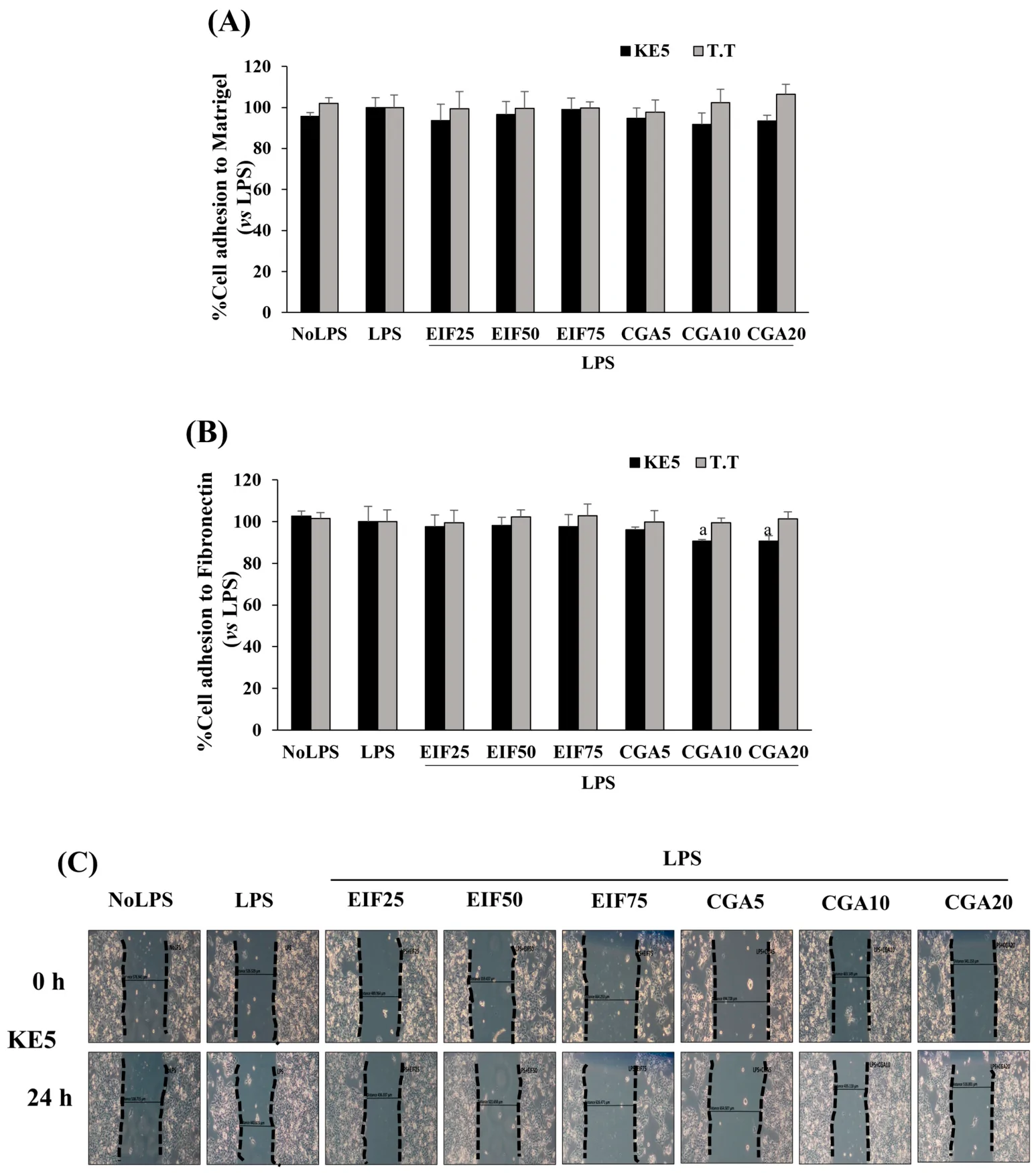

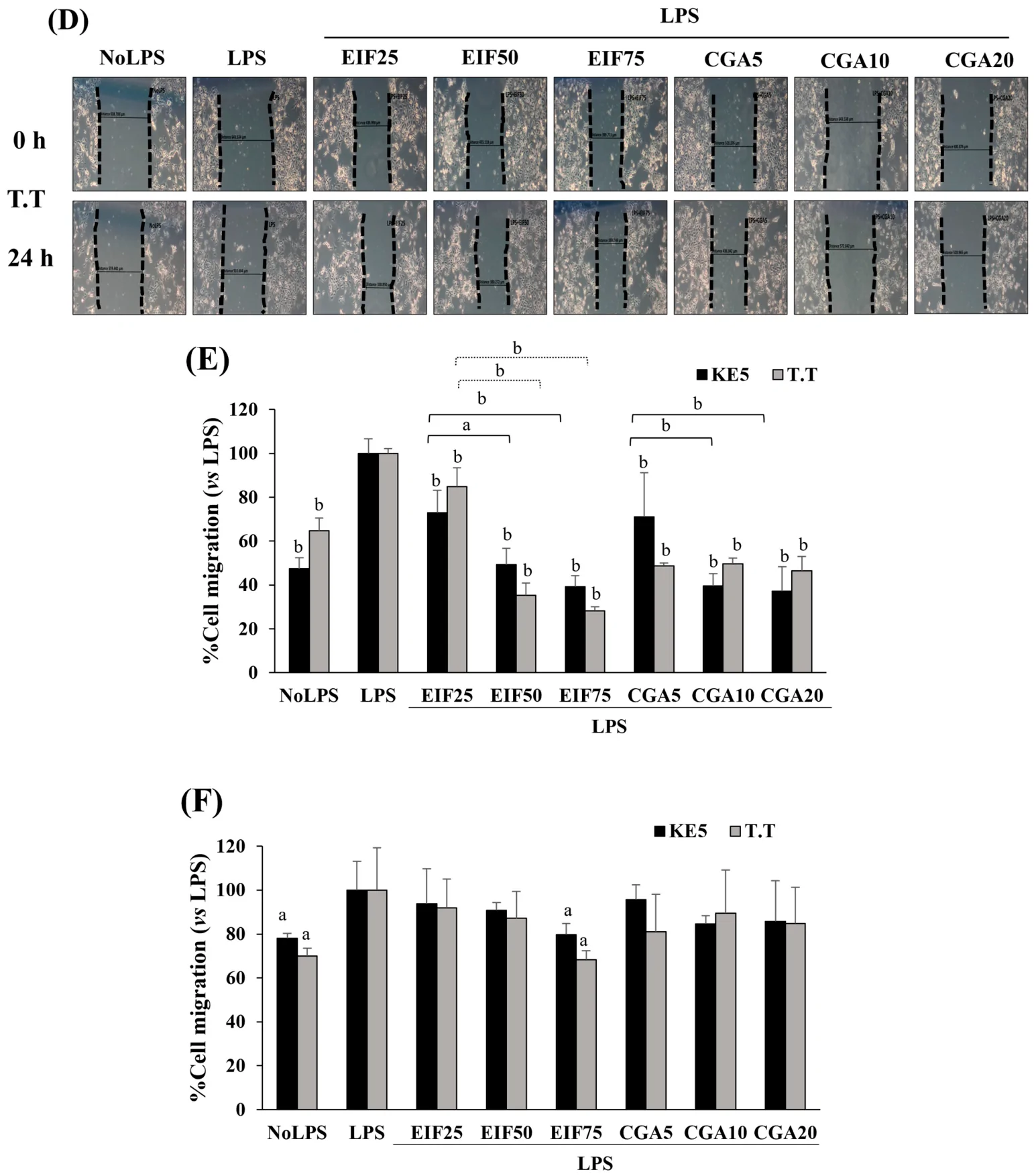

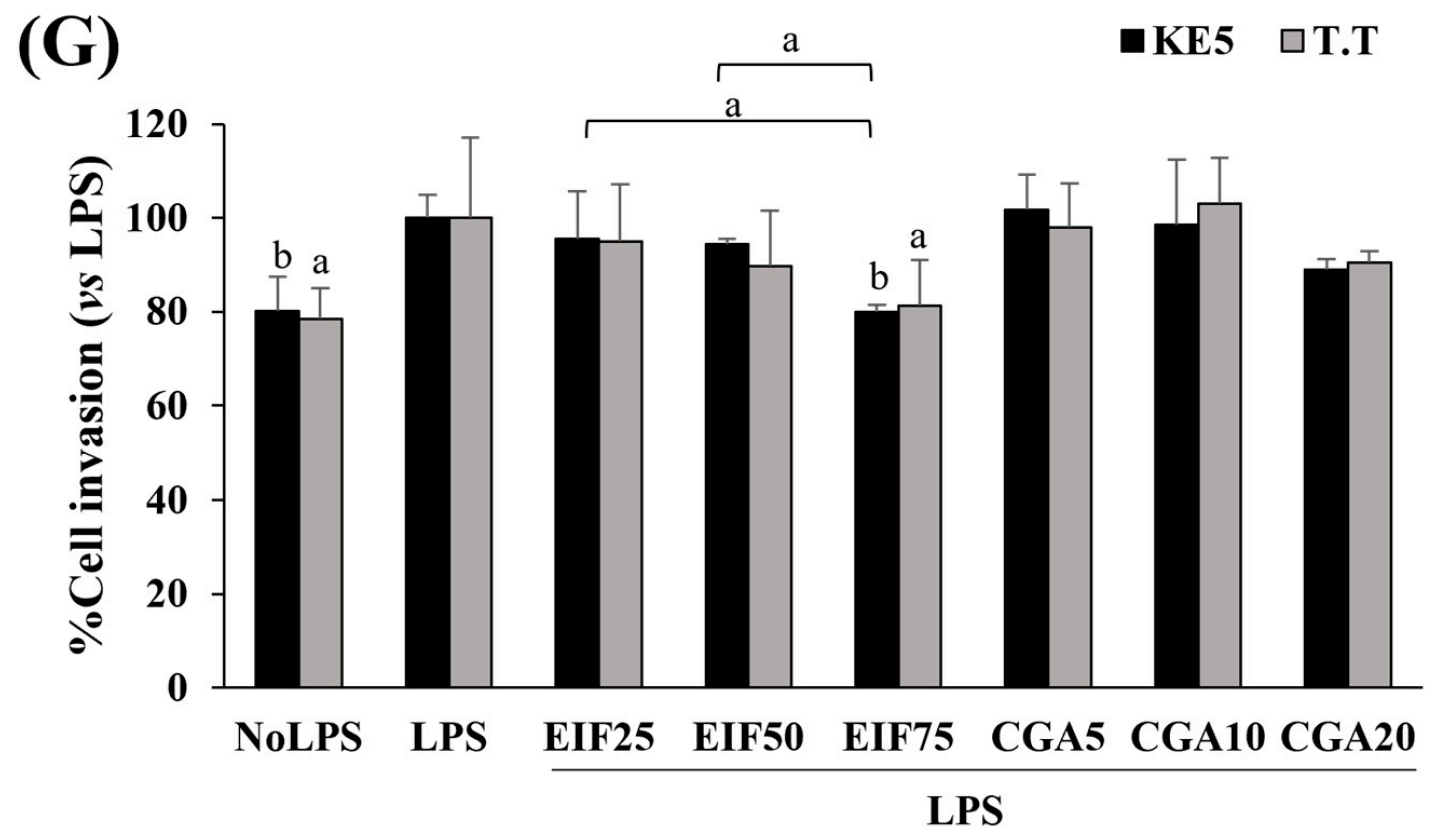
**Effects of EIF and CGA on the activity of cancer progression in LPS-PG-pretreated ESCCs.** LPS-pretreated KE-5 and T.T cells were examined for the role of EIF and CGA on cell-Matrigel (**A**) and fibronectin (**B**) adhesion, cell 2D (**C**–**E**) and 3D (**F**) migration, and cell invasion (**G**). These were assessed using cell adhesion, cell migration (wound healing and Transwell assay), and cell invasion (Transwell assay) assays. The significant values (compared to the LPS control), shown by the letters a and b, are *p* < 0.05 and *p* < 0.01, respectively.

Three main activities contribute to cancer development: cell adhesion, migration, and invasion. Our findings showed that LPS-PG pretreatment (1 µg/mL for 2 h) resulted in no alterations in cell adhesion (Figure 5A,B), whereas LPS-PG increased cell migration in 2D (Figure 5C–E) and 3D (Figure 5F) patterns, as well as cell invasion (Figure 5G) in both ESCC cell lines. In cell adhesion assays, only cell-fibronectin adhesion slightly decreased in KE5 cells treated with CGA (approximately 4–10% vs. LPS-PG). Regarding cell migration and invasion, EIF administration reduced these LPS-PG-activated events, especially at 75 μg/mL, whereas CGA attenuated LPS-PG-stimulated ESCC migration only in 2D.

### 2.6. Effects of EIF and CGA on the pp65/p65 and pp38/p38 Pathways in LPS-PG-Pretreated ESCCs

**Figure 6:**
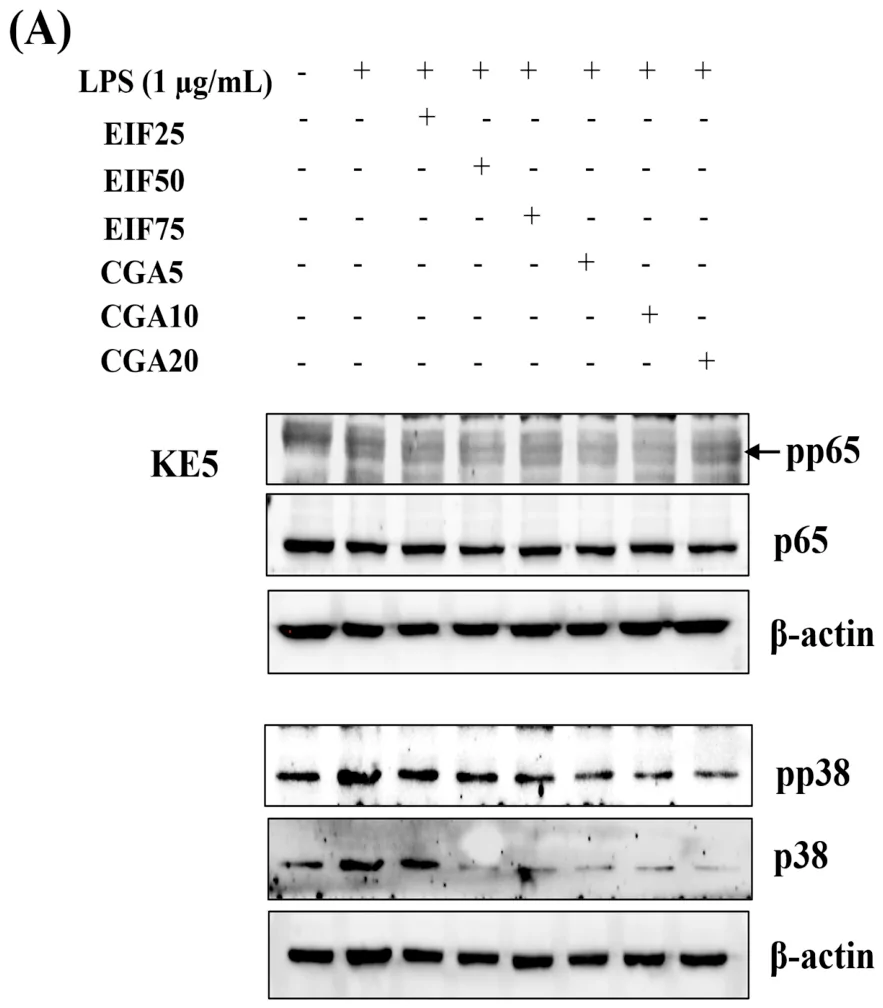

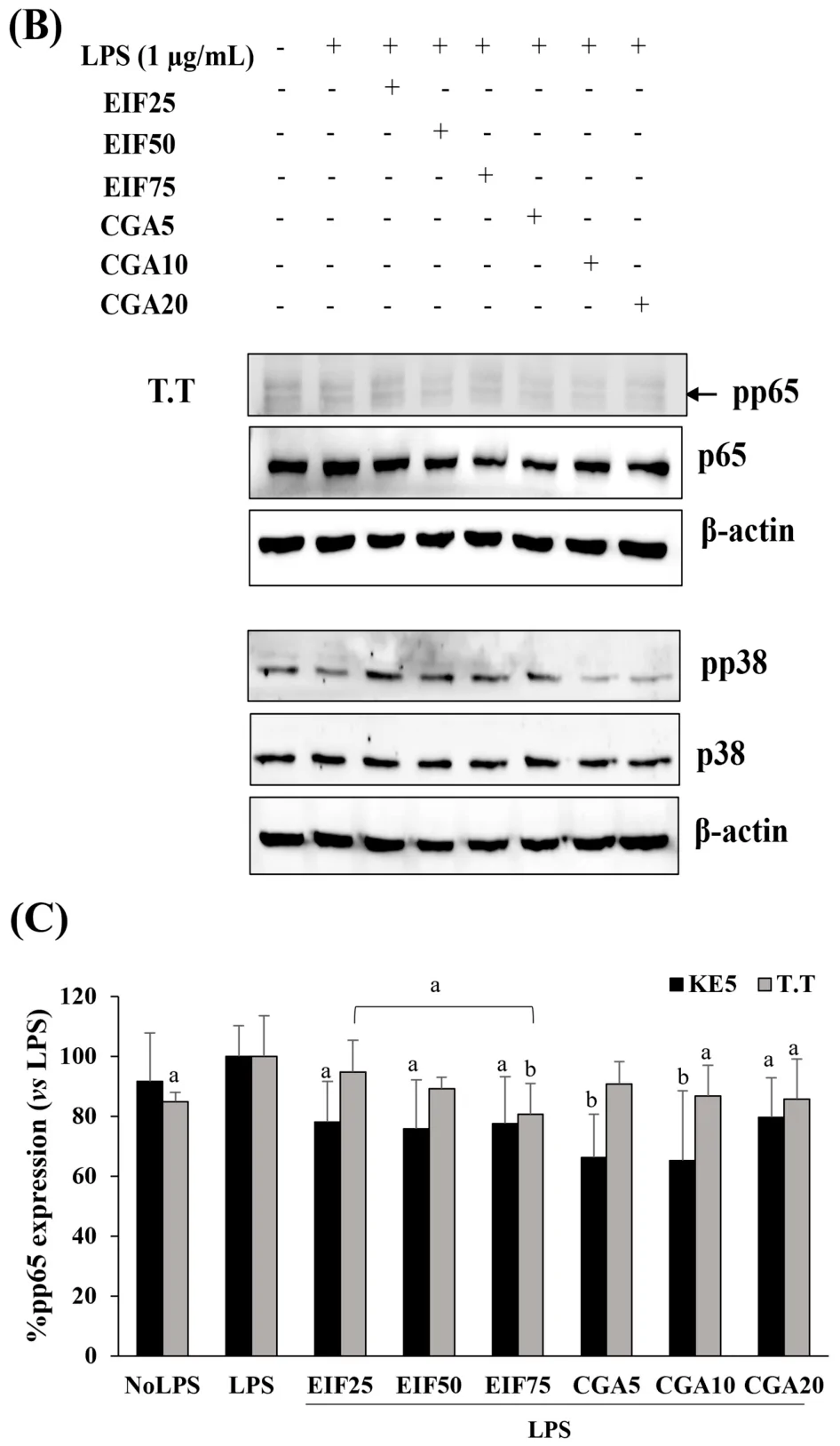

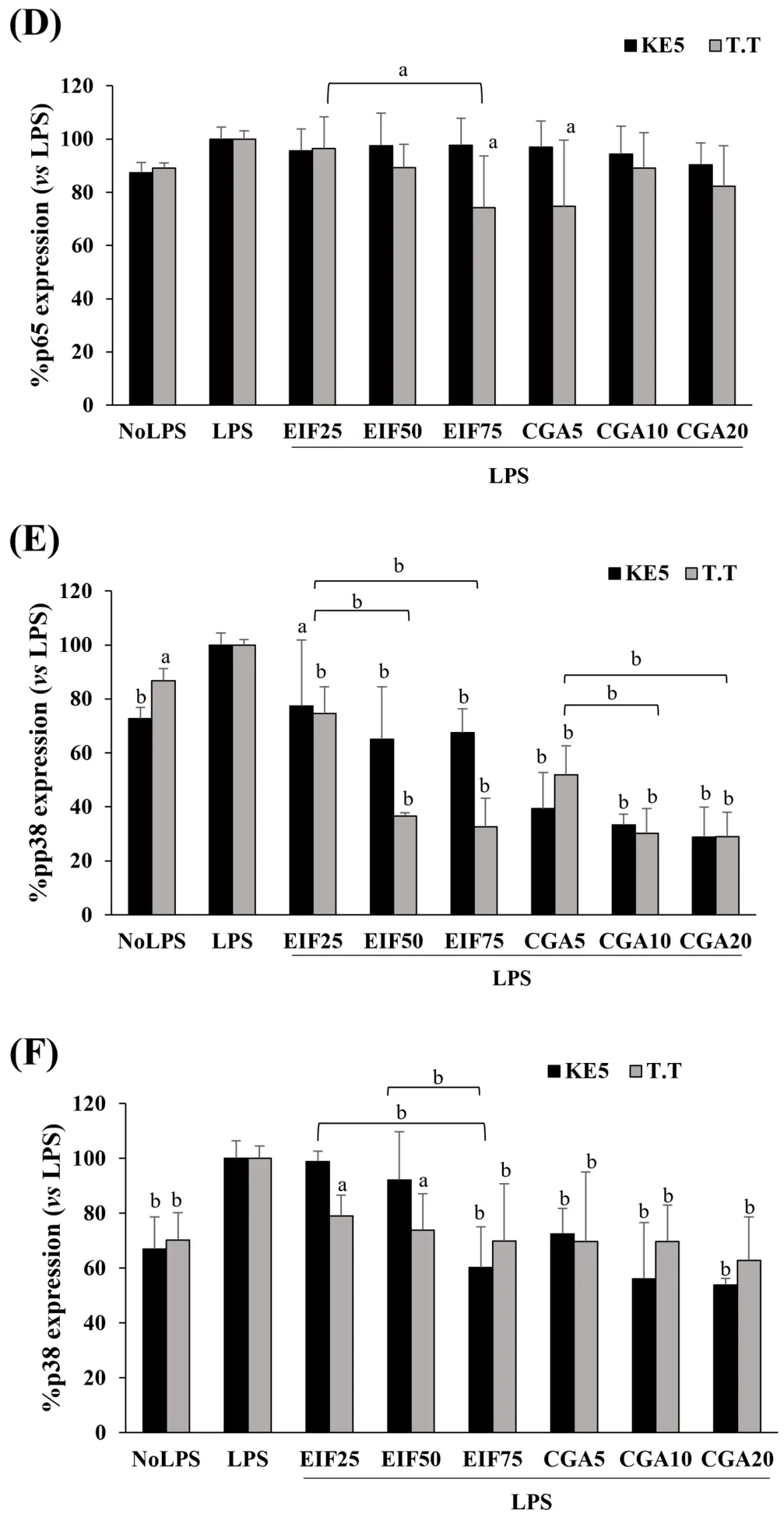

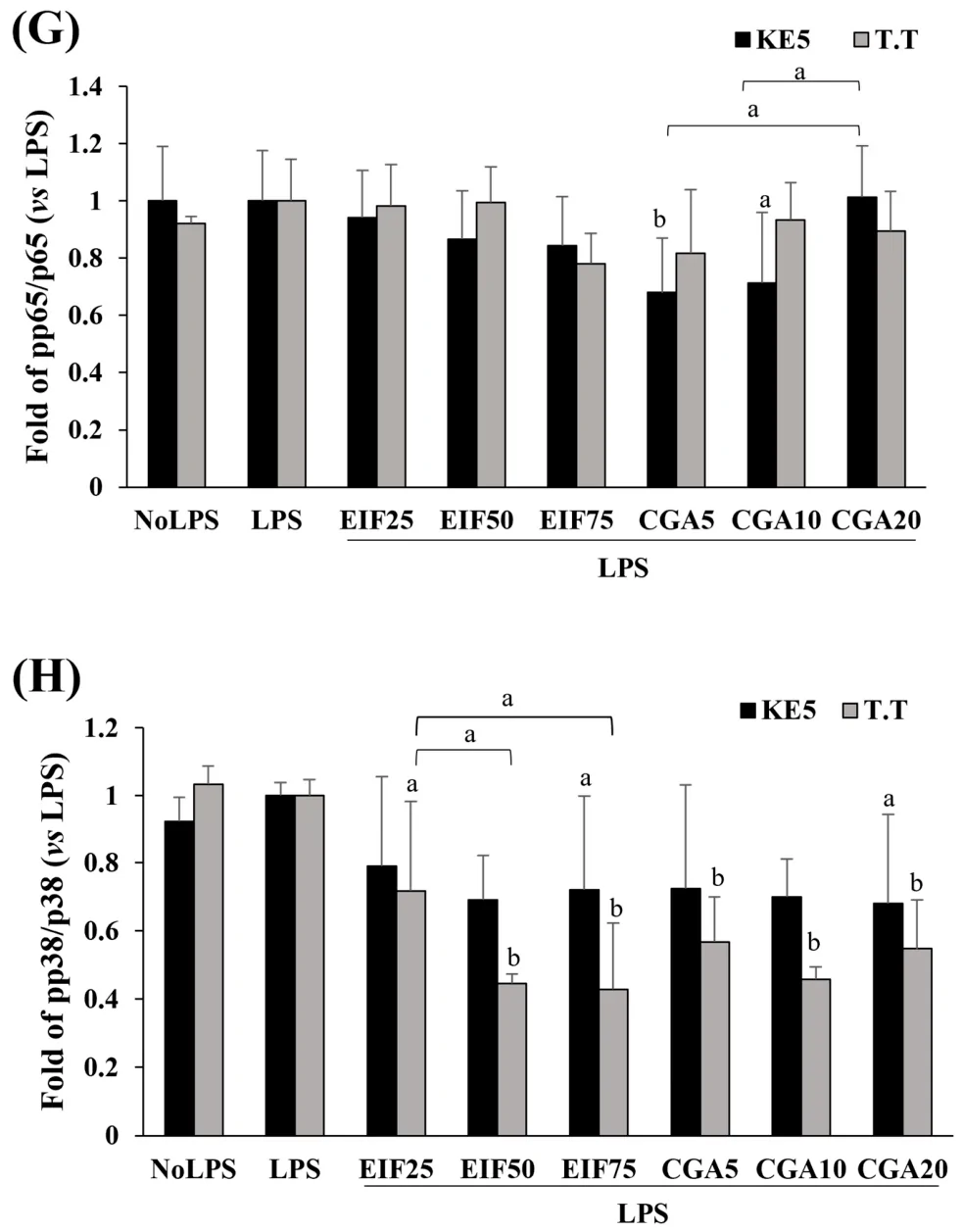
**Effects of EIF and CGA on the pp65/p65 and pp38/p38 pathways in LPS-PG-pretreated ESCCs.** Different EIF- and CGA-treated lysates of LPS-PG-pretreated KE5 (**A**) and T.T (**B**) cells were obtained to assess the regulatory mediators pp65 (**C**), p65 (**D**), pp38 (**E**), and p38 (**F**) using Western blot analysis. The pp65/p65 (**G**) and pp38/p38 (**H**) ratios are also shown. The significant values (compared to LPS), shown by the letters a and b, are *p* < 0.05 and *p* < 0.01, respectively.

Our findings show that LPS-PG affected each ESCC cell line differently. In both cell types, LPS-PG stimulated both pp38 and p38 (Figure 6E,F), and the pp38/p38 ratio did not differ (Figure 6H). In contrast, only pp65 in T.T cells responded mildly to LPS-PG, increasing by 16% (Figure 6C). However, this shift was not enough to change the pp65/p65 ratio (Figure 6G). Nonetheless, CGA and EIF exhibited a similar inhibitory pattern across cell types. Their inhibitory action covered all pp65/p65 and pp38/p38 in T.T cells and only pp65 and pp38/p38 in KE5 cells. Remarkably, CGA gradually raised pp65 expression at the higher dose in KE5 cells, as also reflected in the pp65/p65 ratio (Figure 6G). Overall, the pp38/p38 ratio (Figure 6H) changed more than the pp65/p65 ratio (Figure 6G), indicating that pp38/p38 may be the main target of EIF and CGA.

### 2.7. Effect of the Combination of Paclitaxel and EIF in LPS-PG-Pretreated ESCC

**Figure 7:**
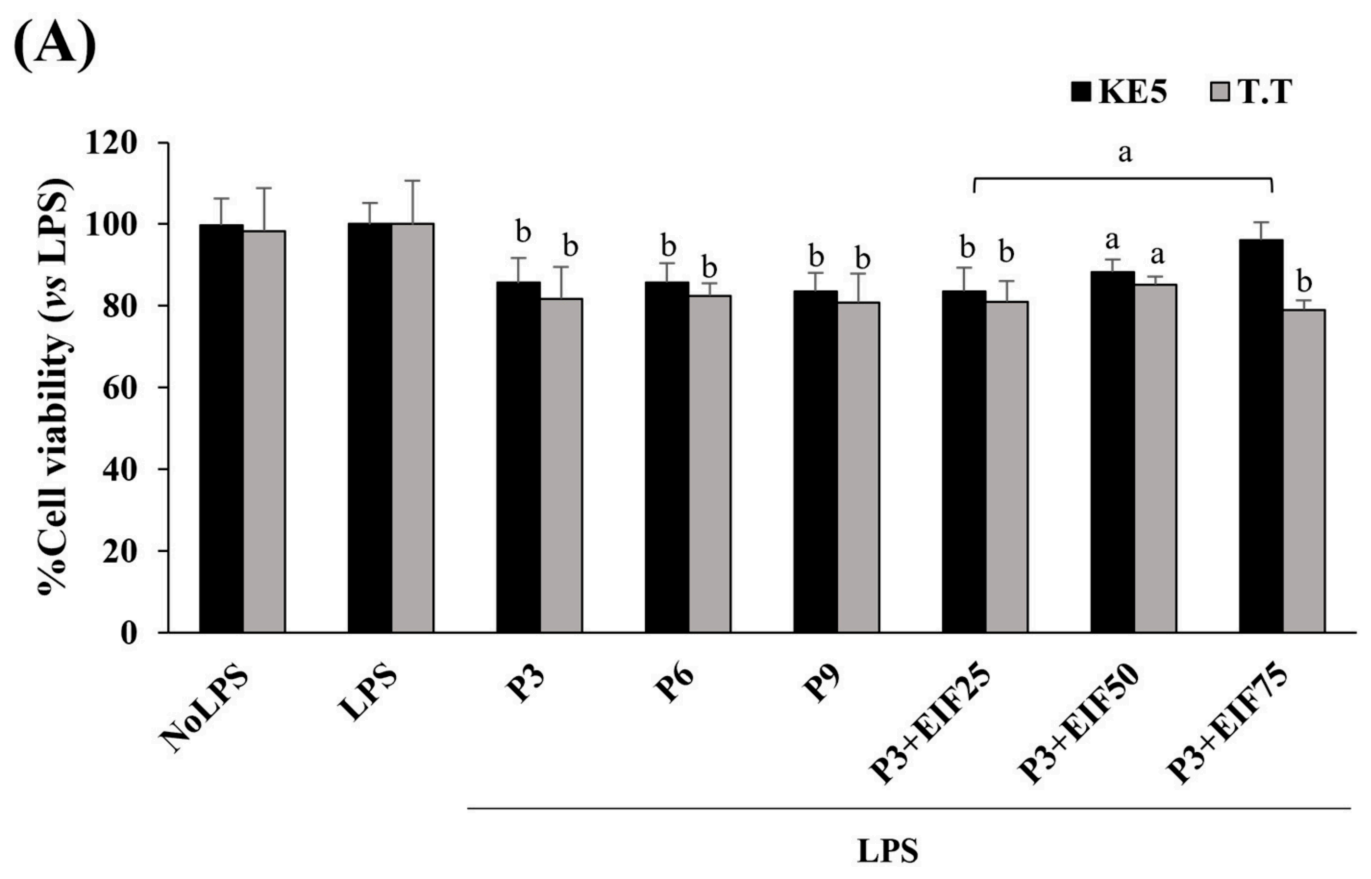

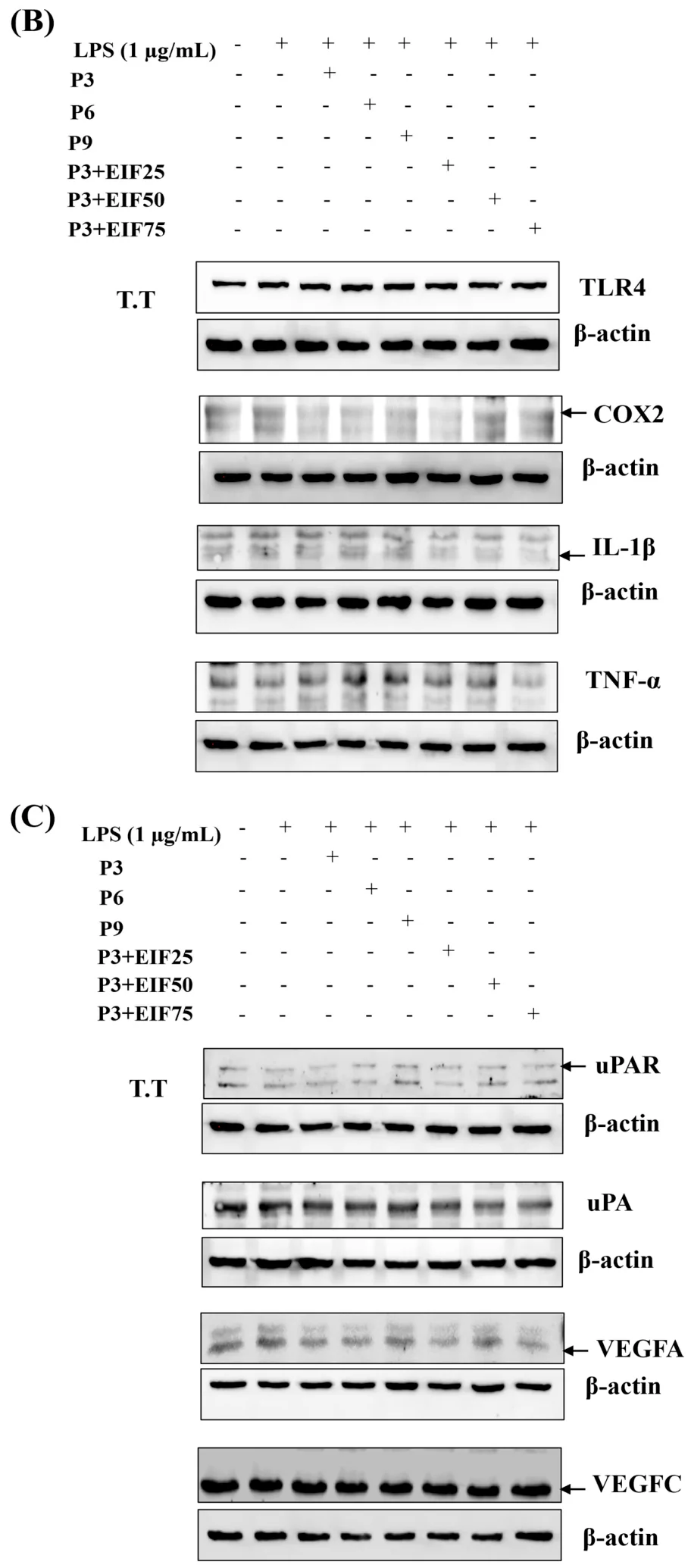

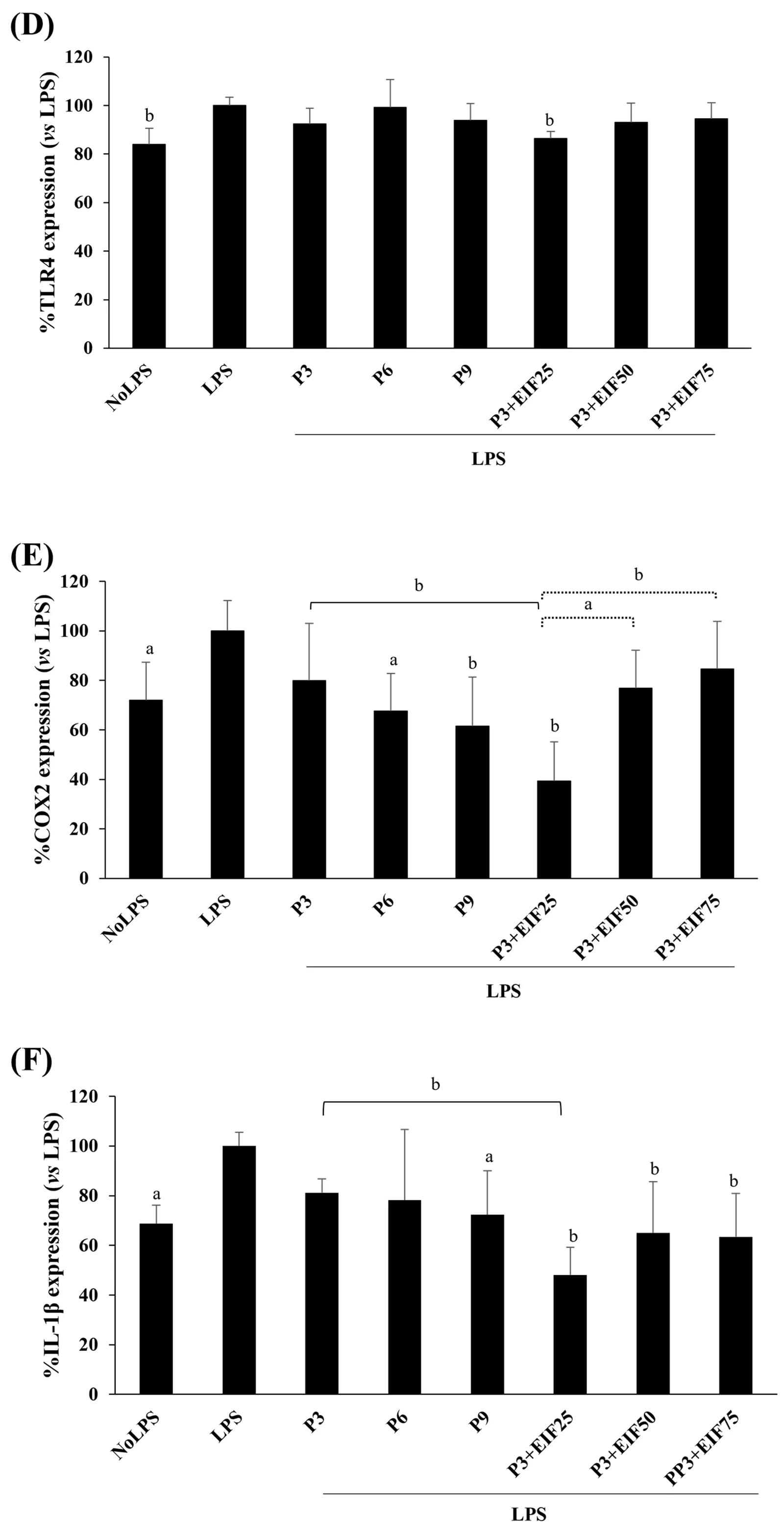

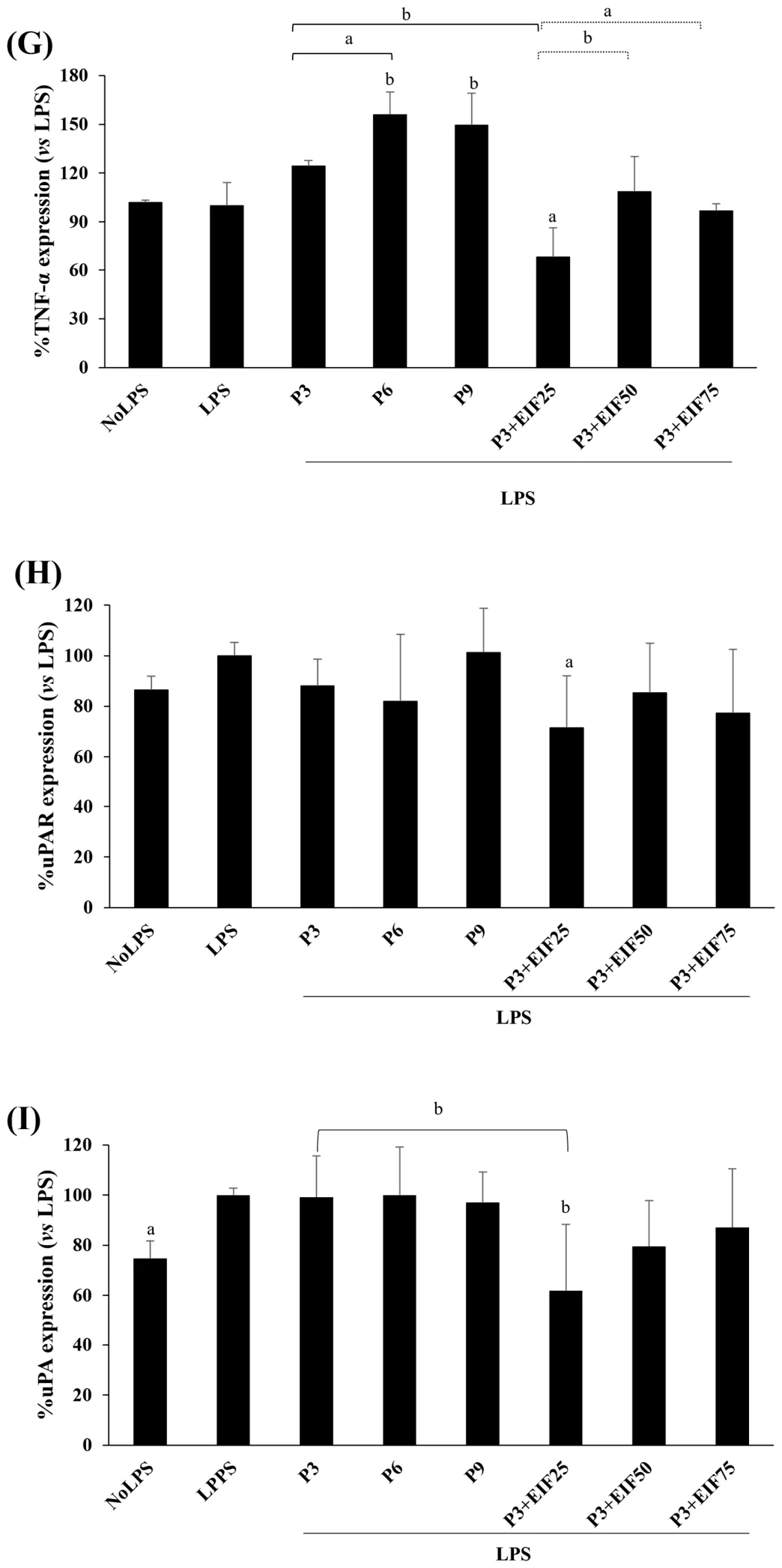

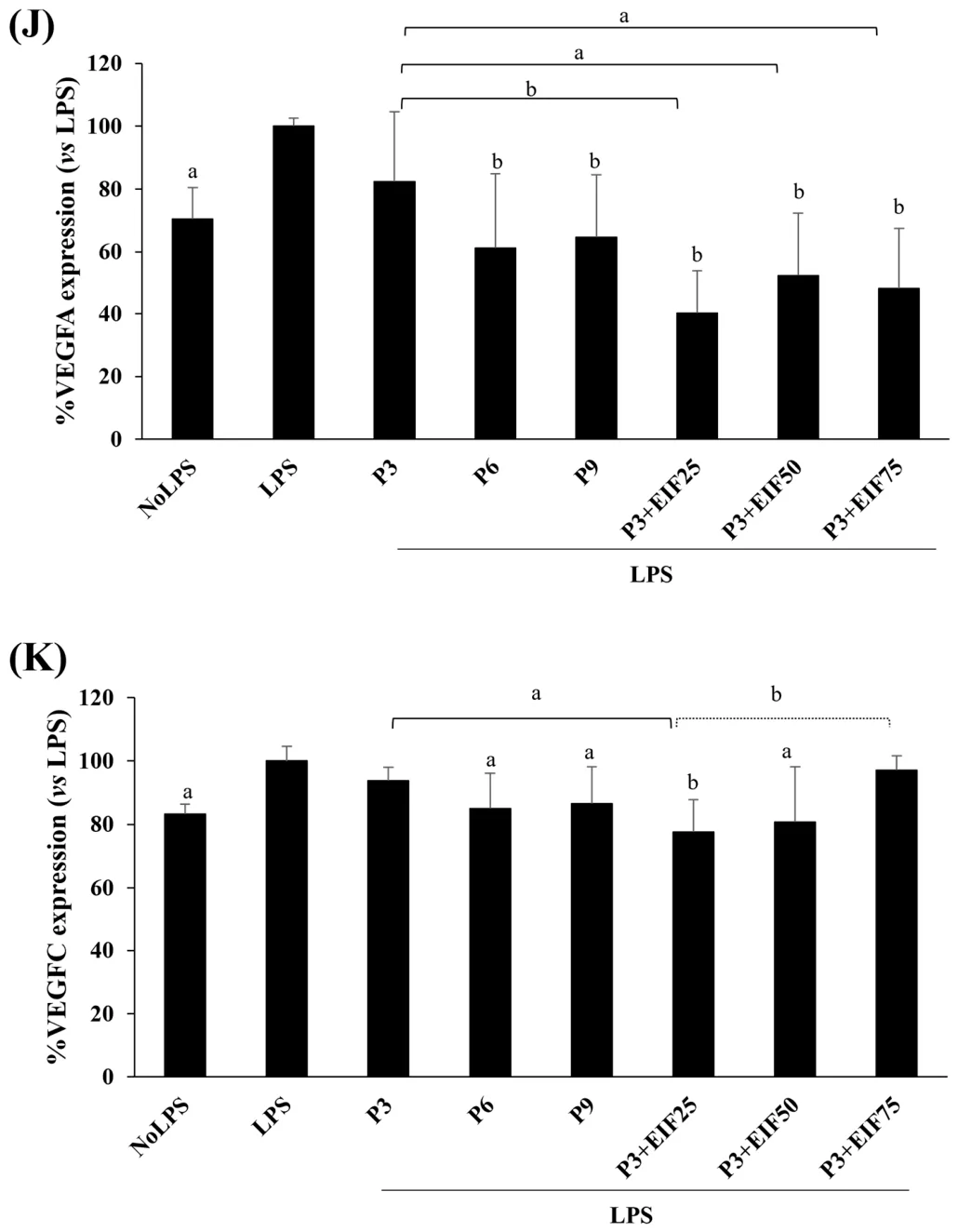

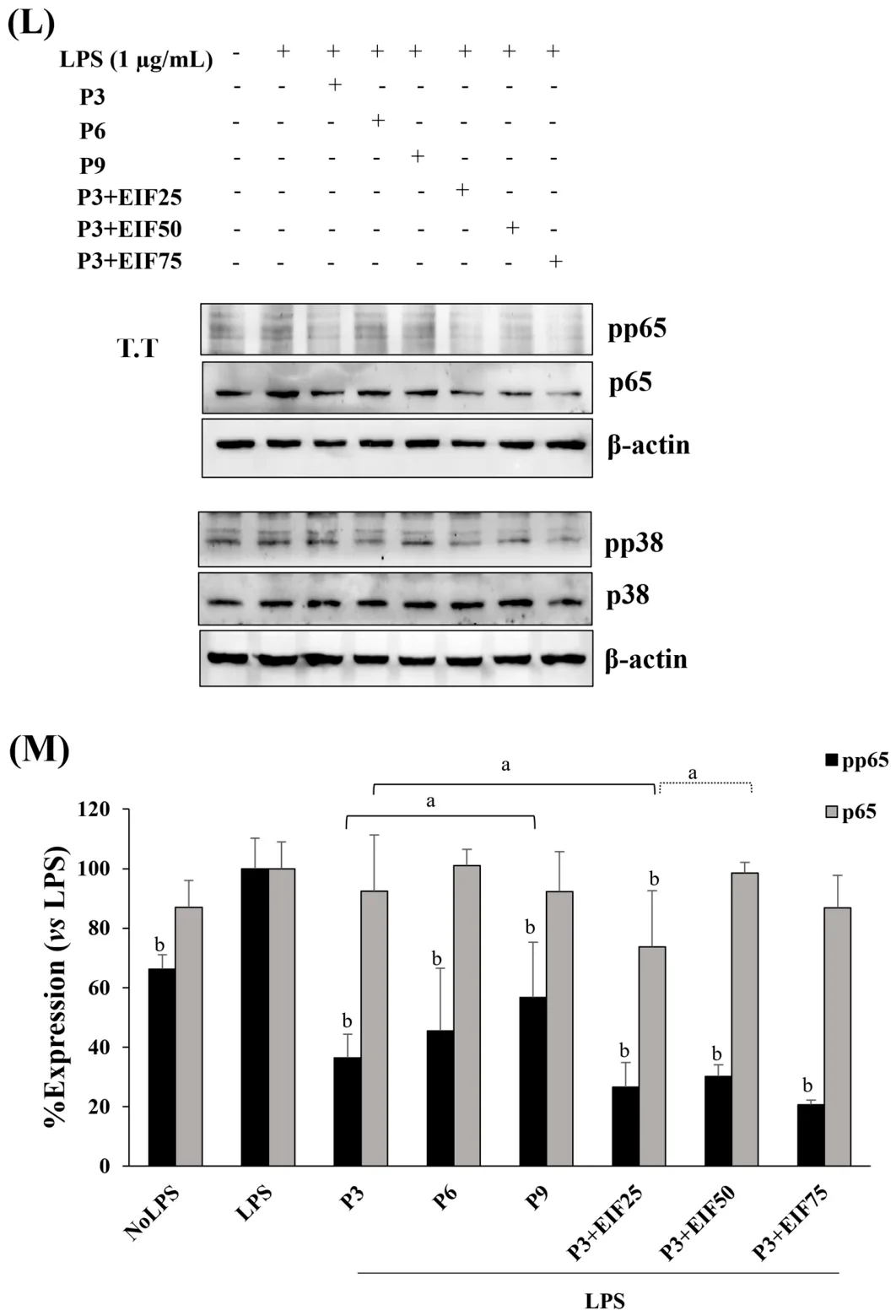

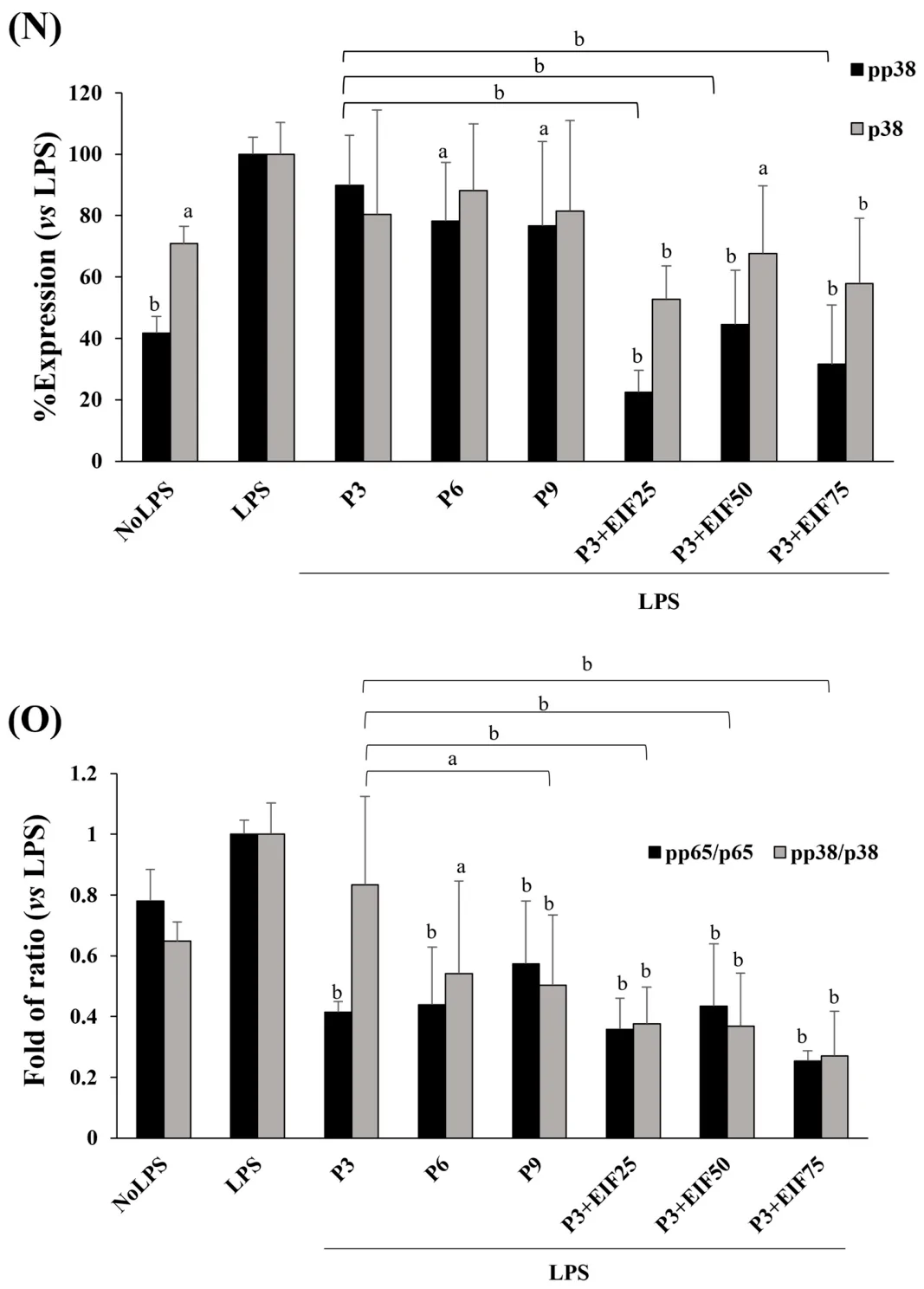
**Effect of the combination of paclitaxel and EIF in LPS-PG-pretreated ESCC.** The MTT assay was used to assess the role of PTX and its combination with EIF in the survival of LPS-PG-pretreated KE5 and T.T cells (**A**). Moreover, the Western blot assay was also used to assess the roles of PTX and its combination with EIF in the inflammatory response (**B**,**D**–**G**), cancer progression (**C**,**H**–**K**), and the regulatory pathway (**L**–**O**) of LPS-PG-pretreated T.T cells. The letters a and b indicate significant differences relative to LPS (*p* < 0.05 and *p* < 0.01, respectively). (P = Paclitaxel or PTX).

Paclitaxel (PTX) is a chemotherapeutic drug that is commonly used in combination with other agents. In this study, the inhibitory cytotoxic effects of PTX alone (3–9 μg/mL) achieved a limited cytotoxicity of roughly 15–20%; no dose response was present in both LPS-PG-pretreated ESCCs. Meanwhile, PTX (3 μg/mL) combined with EIF (25–75 μg/mL) was comparable in T.T cells. Notably, co-treatment with PTX and EIF in KE5 cells showed a slight, gradual increase in cell viability at higher concentrations, reaching approximately 11% at 75 µg/mL EIF compared with PTX alone (Figure 7A); however, this variation was not statistically significant (*p* > 0.05). Consequently, based on a post hoc inspection of the viability data, further combination analyses were selectively focused on T.T cells, as the data remained stable without upward fluctuation among the groups (*p* > 0.05), and they demonstrated greater responsiveness to EIF (as shown in the previous results). Next, our results on inflammatory response mediators (Figure 7B,D–G) showed that PTX did not significantly alter LPS-PG-stimulated TLR4, COX2, and IL-1β, while TNF-α was gradually elevated in a dose-dependent manner. In cancer-related mediators (Figure 7C,H–K), single PTX hardly altered uPA and uPAR levels, except that PTX at 6 and 9 μg/mL depleted LPS-PG-stimulated VEGFA and VEGFC expression. Remarkably, combining PTX at 3 μg/mL with EIF, especially at 25 μg/mL, gave the greatest inhibitory effect. In other words, EIF at 25 μg/mL enhanced the inhibitory efficacy of PTX at 3 μg/mL. Next, to study the regulatory pathways (NF-ĸB and p38) (Figure 7L–N), single PTX downregulated LPS-PG-stimulated pp65 and pp38, but not p65 and p38 in T.T cells. In combination with EIF, pp65 and p65 were slightly lower, while pp38 and p38 were more depleted than with PTX alone at 3 μg/mL. Consistently, the pp65/p65 ratio in the combination rarely changed, whereas the pp38/p38 ratio was clearly depleted compared with PTX alone at 3 μg/mL (Figure 7O). Interestingly, pp65 levels rose similarly to TNF-α, and the pp65/p65 ratio gradually increased with higher PTX concentrations. Across all results, EIF increased PTX potency and the number of affected targets, particularly at 25 μg/mL.

## 3. Discussion

Periodontal bacteria, especially *P. gingivalis*, have been repeatedly linked to ESCC development in numerous studies. Additionally, this study demonstrated a link between LPS-PG, inflammatory responses, and ESCC progression. First, our results showed that LPS-PG effects on ESCC also varied. Indeed, LPS action depends on cell type, LPS structure, dose, and treatment duration [15,43,44,45,46]. Researchers widely use high LPS concentrations (100 ng/mL to 1 μg/mL) in vitro because they effectively induce a robust inflammatory response [47], while short-term exposure minimizes potential cytotoxicity. In our study, LPS-PG at a dose of 1 μg/mL for 2 h did not alter ESCC cell survival, varying marginally by only 1–2% in both KE5 and T.T cells. This finding suggested that the LPS-PG condition may be insufficient to enhance ESCC survival. Nevertheless, LPS-PG has been revealed to promote proliferation and inhibit apoptosis in ESCC cells, as previously reported in our prior study [12]. However, further studies should assess LPS-PG dose and duration, key regulators of apoptosis and the cell cycle, and additional methods (e.g., p53 assessment, apoptosis, and clonogenic assays). Moreover, the EIF and CGA concentrations used in this study had minimal effects on ESCC cell viability, with or without LPS-PG pretreatment. However, the cell viability assay (MTT) has a limitation with CGA and EIF, since MTT can interact directly with residual CGA or EIF to produce color, and standard washouts cannot completely rule out chemical interference. The study should have included a cell-free MTT control or an orthogonal viability assay, but it did not. Thus, the cell viability data should be interpreted with caution. Consequently, low-range concentrations with no toxicity of EIF (25–75 μg/mL) and CGA (5–20 μg/mL) were selected for subsequent experiments.

Next, our study focused on the classical pro-inflammatory cascade activated by LPS-PG by verifying the expression of TLR4, TNF-α, IL-1β, and COX2. The LPS-PG treatment (1 μg/mL for 2 h) upregulated TLR4, COX2, and IL-1β, whereas TNF-α remained unaffected in both ESCC cell lines. EIF inhibited these expressions, with greater inhibition in T.T. cells (COX2, IL-1β, and TNF-α) than in KE5 cells (COX2 and TNF-α), especially at a high EIF dose. In CGA treatment, the inhibitory pattern varied and rarely coincided with EIF; its selective activity targeted TNF-α in KE5 cells and COX2 in T.T. cells. However, broader profiling of cytokine expression and secretion, e.g., IL-6 and IL-8, is also essential for further study in the future to fully elucidate the responsiveness of LPS-PG. In the context of malignant phenotypes, LPS-PG induction (1 μg/mL for 2 h) was more effective at inducing expression of several cancer mediators (e.g., uPA, VEGFA, and VEGFC) in T.T. cells. In contrast, it induced less in KE5 cells, except for VEGFA, which increased by 16%. EIF inhibition was more effective in T.T. cells, while only uPA and VEGFA decreased in KE5 cells. Even so, CGA abrogation was narrower than EIF in T.T. cells (observed only in uPAR, uPA, and VEGFC at a low CGA dose) but comparable to EIF in KE5 cells. Remarkably, high-dose CGA caused a mild, non-significant upregulation of VEGFC expression in KE5 cells (around a 7% increase at 20 μg/mL CGA) but not in T.T cells, suggesting a cell-line-specific response that warrants careful consideration.

Furthermore, our findings demonstrated that LPS-PG (1 μg/mL for 2 h) weakly and selectively activated certain cancer-related activities in both ESCC cell lines, including VEGFC and gelatinase secretion, cell migration, and invasion, with the most pronounced effects found in T.T. cells. As mentioned above, EIF was more effective than CGA at inhibiting LPS-PG-stimulated cancer progression, particularly in T.T. cells, e.g., VEGFC and gelatinase secretion, gelatinase activity, cell migration (2D & 3D movement), and cell invasion. Specifically, the highest concentration of EIF (75 μg/mL) markedly suppressed LPS-PG-stimulated cell migration and invasion in both cell lines, achieving approximately 20–30% inhibition. In contrast, this treatment had only a minor impact on cell proliferation, reflected by a reduction in cell survival of only 6–10%. These observations indicate that the anti-migratory and anti-invasive effects of EIF were not primarily driven by cytotoxicity. Interestingly, VEGFA secretion did not differ significantly between the two cell types across any treatment, including LPS-PG, EIF, and CGA. Notably, their effects on VEGFA secretion were unrelated to VEGFA expression (as described above), suggesting that these compounds primarily act at the expression level rather than regulating the VEGFA secretory system. Overall, CGA exhibited limited and weak potency across almost all assessed oncogenic activities. Interestingly, CGA uniquely and modestly suppressed KE5 cell adhesion to fibronectin (approximately 4–10% inhibition), while the other conditions did not.

The two main regulatory pathways (NF-κB and p38 MAPK) responded differently in the two ESCC cell lines after LPS-PG treatment (1 μg/mL for 2 h), especially the p38 pathway. LPS-PG enhanced both pp38 and p38 expression, but the ratio pp38/p38 was unchanged. This suggests that the rise in pp38 with LPS-PG activation may affect total p38 expression more than phosphorylation. Regarding the NF-κB pathway, only pp65 in T.T cells responded to LPS-PG stimulation, exhibiting a marginal 16% induction. This minor increase was associated with a negligible change in the pp65/p65 ratio. Later, EIF abrogated the effect on both pp65 and p65 expression in T.T. cells, while the pp65/p65 ratio was barely altered, suggesting that EIF primarily downregulates total p65 levels in this cell line. In KE5 cells, EIF only modestly suppressed pp65 levels (22–24%), which was insufficient to significantly alter the pp65/p65 ratio. Meanwhile, CGA’s effects on pp65 and total p65 were nearly identical to EIF’s. Interestingly, in KE5 cells, although lower doses showed slight pp65 depletion, higher concentrations showed a gradual increase in pp65 levels. Correspondingly, the pp65/p65 ratio increased in parallel, indicating that CGA primarily modulates NF-κB activation status rather than altering total p65 protein expression. In T.T cells, pp65 levels were mildly suppressed by CGA (10–15%), while total p65 levels were also modestly reduced (11–26%), which did not substantially alter the pp65/p65 ratio. Thus, the differential effects of EIF and CGA appear to depend on dose and cell type. In the p38 MAPK pathway, our results showed that both EIF and CGA attenuated pp38 and total p38 expression, as well as the pp38/p38 ratio in both ESCC cell lines. This indicates that EIF and CGA reduced phosphorylated p38 more than total p38. Of all, broad EIF inhibition seemed to regulate the pathway primarily through changes in protein levels.

Overall, LPS-PG at 1 μg/mL for 2 h mildly induced inflammatory responses and several malignant phenotypes in both ESCC cell lines. It is postulated that standard-grade LPS-PG (lot no. 5916-47-01, cat: tlrl-pglps, standard grade, InvivoGen, CA, USA), which contains co-purified bacterial lipopeptides, acts as a dual TLR2 and TLR4 agonist. Although TLR4 is widely recognized as a primary target of LPS-PG as described in the literature [48], the observed lack of a robust downstream response led to the hypothesis that these cellular activities may be partially driven by the concurrent activation of alternative receptors, such as TLR2. Activation of the TLR2 signaling pathway leads to a distinct, weaker inflammatory response [49], potentially explaining the attenuated downstream cascades observed in our models. Consequently, future investigations should include TLR4 or TLR2 knockdown or inhibitor experiments to comprehensively elucidate these pathways.

Next, the anti-inflammatory and anti-progression effects against LPS-PG stimulation were explored in both ESCC cell lines. EIF exhibited more potent effects, appearing to particularly target T.T cells. In addition, EIF downregulated the NF-ĸB and p38 MAPK pathways activated by LPS-PG in ESCC. It revealed that the inhibitory effect of EIF was related to both pathways. Interestingly, T.T cells (derived from the metastasis site) showed greater responsiveness to LPS-PG-stimulated malignant behaviors. This postulates that metastatic cells exhibit a functional dependency on hyperactivated NF-κB signaling to remodel their phenotype and drive tumor progression. Consequently, downregulating this pathway severely affects metastatic cells far more than non-metastatic tumor cells [50,51,52].

Although CGA did not have a major impact in this study, prior research has shown that ESCC subtype and high CGA dosage affected CGA’s anti-metastasis effectiveness in ESCC [53,54]. Similarly, evidence supports that the CGA-rich fraction from GCB is more effective and broader-acting than pure CGA in various biological activities [55]. Consequently, the multi-component mixture within EIF potentially exerts a more dominant biological effect than pure CGA alone. However, further comprehensive characterization—including LC-MS profiling, batch variability assessment, and targeted validation using pure caffeine or CGA-caffeine combinations—is required to definitively substantiate this hypothesis.

Although this study demonstrated the effects of LPS-PG on ESCC, it did not assess its impact on normal esophageal cells. To achieve a more comprehensive understanding, further investigation on normal cells is warranted in the future.

Next, the effects of paclitaxel (PTX) and its combination with EIF were investigated in LPS-PG-stimulated ESCC cells. Interestingly, PTX at concentrations of 3–9 µg/mL showed no clear dose-dependent cytotoxicity in LPS-PG-pretreated ESCC cells after 24 h of exposure, with only approximately 15–20% inhibition. Recent studies have demonstrated that LPS-PG promotes chemoresistance and radioresistance in both OSCC and ESCC, which may contribute to the reduced PTX sensitivity observed in the present study. Notably, regarding the combination of EIF and PTX in LPS-PG-pretreated KE5 cells, although a slight upward trend in cell viability was visually observed at EIF 75 μg/mL (nearly 11% compared with PTX alone), this alteration was not statistically significant compared with the PTX-alone group. Meanwhile, the combination in LPS-PG-pretreated T.T cells was comparable to PTX alone (around 15–21% inhibition). Remarkably, the unchanged or slightly increased cell viability in the PTX + EIF combination should also be interpreted with caution due to possible interference from CGA within residual EIF. As noted, a cell-free MTT control should have been included to account for direct chemical reduction. Beyond the lack of a cell-free control, other limitations include the narrow PTX concentration range (3–9 μg/mL) tested at a single 24 h time point and the absence of complementary apoptosis, clonogenic, or orthogonal viability assays. To conclusively clarify these survival profiles, future research should incorporate these comprehensive approaches.

To further investigate the therapeutic potential of this combination in the cell line most responsive to EIF, based on the overall findings, T.T cells (highly metastatic ESCC) were selected for subsequent comprehensive studies, focusing on both inflammatory responses and ESCC progression. Our results showed that PTX at 6 and 9 μg/mL increased TNF-α expression (approximately 50–55% increase), which in turn produced inflammation. According to Elzaitony et al. (2024) [56], PTX attenuates LPS-induced inflammation at low doses while acting like LPS or an inflammatory inducer at high doses.

In LPS-PG-stimulated ESCC progression, PTX at higher doses (6 and 9 μg/mL) inhibited VEGFA and VEGFC expression. Although PTX alone at 3 μg/mL had no significant effect, combining it with EIF enhanced this lower dose, revealing an enhanced effect against LPS-PG-stimulated inflammatory responses and ESCC progression, particularly with EIF at 25 μg/mL. However, these benefits gradually diminished at higher EIF doses. Previous reports showed that caffeine counteracts PTX efficacy by modulating key molecular events, such as inhibiting PTX-induced α-tubulin acetylation and preventing PTX-mediated Mcl-1 downregulation across various cancer cell lines [57,58]. Because α-tubulin acetylation and Mcl-1 downregulation are critical for effective anticancer responses [59,60], caffeine present within EIF might interfere with and reduce the efficacy of the EIF and PTX combination. Therefore, this hypothesis warrants further validation using caffeine-only or CGA-plus-caffeine controls to definitively elucidate the underlying mechanisms. Correspondingly, our results show that PTX also downregulated LPS-PG-impacted NF-κB and p38 MAPK. Notably, the reduction in pp65 levels became less pronounced with increasing PTX concentrations, accompanied by a concomitant increase in TNF-α expression (as noted above) and the pp65/p65 ratio. Thus, the goal should be a low-dose PTX regimen with no undesirable side effects, as previous literature also indicated that combining low-dose PTX with other agents enhances efficacy and reduces toxicity [61,62]. However, the pp65/p65 and pp38/p38 ratio results suggested that combining EIF with PTX preferentially modulated the p38 MAPK pathway, whereas its impact on NF-κB was less pronounced. Accordingly, combining EIF and PTX at 3 μg/mL produced a broader and more potent inhibitory effect; in particular, the most potent combination was EIF 25 μg/mL and PTX 3 μg/mL.

**Figure 8:**
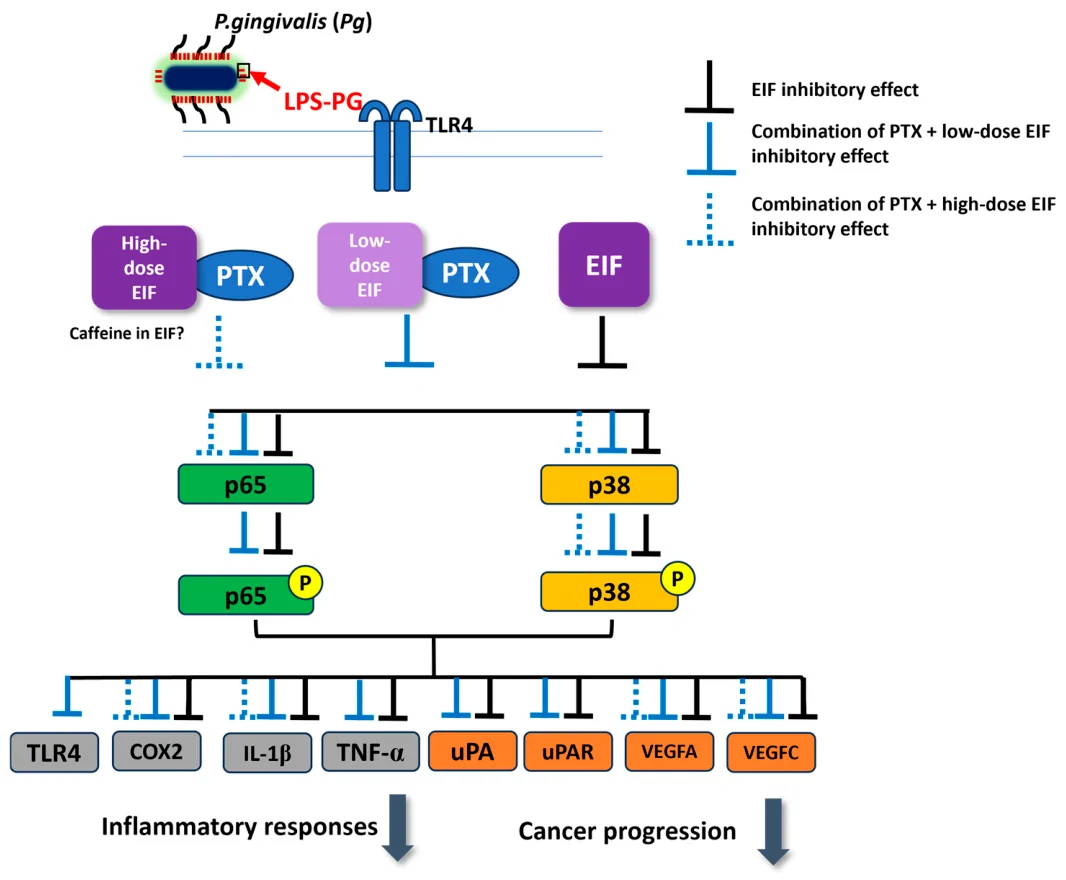
**Summary scheme of EIF actions alone and in combination with PTX.** Schematic diagram illustrating a hypothetical model of EIF and its combination with paclitaxel (PTX) in modulating the NF-κB and p38 MAPK signaling pathways within LPS-PG-stimulated ESCC cells. The model distinguishes between the potent inhibitory effects of low-dose EIF combined with PTX (solid blue lines) and the diminished efficacy observed at high-dose EIF (dotted blue lines), which is hypothesized to be counteracted by caffeine present within EIF.

Nevertheless, the summary scheme in Figure 8 shows that this is the first to highlight the role of EIF in enhancing PTX efficacy in LPS-PG-stimulated ESCC, potentially involving the NF-κB and p38 MAPK pathways. However, PTX sensitivity may also be regulated by other mechanisms. For instance, C-terminal binding protein 1 (CtBP1) has been implicated in ESCC progression and PTX resistance [63]. Therefore, future studies should investigate these alternative pathways and evaluate this combination treatment in dedicated PTX-resistant models.

In addition, this research on the combination with PTX is preliminary and remains limited, as it was evaluated only by cell viability and selected protein markers in T.T cells, without functional migration or invasion assays. Therefore, including these comprehensive functional assays represents a crucial next step for subsequent investigations.

Ultimately, although EIF enhanced PTX’s effect, it is unclear whether this contribution reflects synergy. Further studies incorporating appropriate combination analyses, such as the Chou–Talalay, Bliss, or Loewe methods, are required to determine whether EIF exerts synergistic activity. Despite several limitations, this research serves as an initial study exploring the effects of EIF and its combination with PTX in ESCC. Future studies aimed at overcoming these limitations are therefore essential to provide a more comprehensive understanding.

## 4. Materials and Methods

### 4.1. Plant Extract

The fine powder of dried green coffee beans (*Coffea canephora* or *Coffea robusta*) from Doi Pha Hee, Chiang Rai province, was combined with 1 L of 60% methanol (1:20 (*w*/*v*)), filtered, and evaporated to obtain crude methanolic extract (CME). Thereafter, CME was mixed with hexane (1:1 ratio) to collect the hexane-insoluble fraction (HIF). HIF was continuously separated by ethyl acetate (1:1 ratio) to obtain the ethyl acetate-insoluble fraction (EIF). Chlorogenic acid (CGA), caffeine, and other compounds were quantified in dried EIF using high-performance liquid chromatography (HPLC). The results indicated that CGA and caffeine were the main constituents in EIF, accounting for 17.7 g and 6.6 g per 100 g of extract, respectively. Thus, EIF is also called the CGA-rich fraction of GCB.

### 4.2. Cell Culture

The human esophageal squamous cell carcinoma (ESCC) cell lines, including KE5 and T.T cells, are the main in vitro models. They were obtained from the Japanese Collection of Research Bioresources Cell Bank (JCRB, Osaka, Japan). This was a newly purchased cell line, and the quality control and verification provided by the supplier (JCRB Cell Bank) at the time of purchase were relied upon; thus, no additional cell authentication or mycoplasma testing was performed in our own laboratory. KE5 cells (JCRB1911, P9) are from the primary tumor, while T.T cells (JCRB0262, P101) are an aggressive carcinoma cell line with high metastatic capacity. Cells were cultured in Dulbecco’s Modified Eagle Medium (DMEM) (Invitrogen Corporation, Grand Island, NY, USA) supplemented with 10% fetal bovine serum (FBS) in a CO_2_ incubator. Cells were pretreated with LPS-PG (1 µg/mL for 2 h) (lot no. 5916-47-01, cat: tlrl-pglps, standard grade, InvivoGen, San Diego, CA, USA) in serum-free media. The target medication was paclitaxel (PTX) at various concentrations (Sigma-Aldrich, St. Louis, MO, USA), which was also utilized in a combined investigation. Standard CGA (cat. C3878, Sigma-Aldrich, St. Louis, MO, USA) was evaluated in parallel with EIF for comparative purposes.

### 4.3. Cell Viability Assay

ESCC cells were seeded overnight at 37 °C in a 96-well plate, then the old media was replaced with LPS-PG (1 µg/mL) in serum-free media, and the cells were incubated for 2 h at 37 °C. After incubation, LPS-PG solution was aspirated, and the cells were treated with various concentrations of the investigated substances (EIF, CGA, PTX, and PTX plus EIF). Following a 24-h incubation period at 37 °C, the conditioned media were removed, and the cells were washed with serum-free medium. Thereafter, cells were exposed to 3-(4,5-Dimethylthiazol-2-yl)-2,5-diphenyltetrazolium bromide (MTT) solution (Sigma-Aldrich, St. Louis, MO, USA) for 2 h at 37 °C, and the crystals were dissolved in dimethyl sulfoxide (DMSO). The absorbance was measured immediately at 540 nm and converted to a percentage of cell viability (relative to LPS, set to 100%).

### 4.4. Western Blot Immunoassay

ESCC cells were seeded in a 60 mm dish overnight at 37 °C, and then the medium was removed, and the cells were incubated with LPS-PG (1 μg/mL) in serum-free media for 2 h at 37 °C. Afterward, the LPS-PG was removed, and the cells were incubated under various treatment conditions in serum-free media for 24 h at 37 °C. Each cell lysate was collected. An equal amount of protein from each cell lysate was loaded onto a 10% sodium dodecyl sulfate-polyacrylamide gel electrophoresis (SDS-PAGE) gel. Proteins were then moved to a polyvinylidene difluoride (PVDF) membrane. After soaking the membrane in the blocking solution, each primary antibody (Santa Cruz Biotechnology, Dallas, TX, USA) was added at a 1:1000 dilution ratio, including TLR4 (sc-293072), TNF-α (sc-52746), COX2 (sc-19999), IL-1β (sc-12742), uPA (sc-59727), uPAR (sc-13522), VEGF (sc-7269), VEGFC (sc-374628), p65 (sc-8008), pp65 (sc-136548), p38 (sc-7972), pp38 (sc-166182), and β-actin (sc-47778). The membrane was reacted with HRP-conjugated secondary antibodies at a 1:1000 dilution (AP124P, Merck Millipore, Burlington, MA, USA) and enhanced chemiluminescence (ECL) substrate (Bio-Rad^®^ Laboratories, Hercules, CA, USA) for detection. A ChemiDoc Imager (Bio-Rad^®^ Laboratories, CA, USA) and GelPro32 software version 3.0 were used to gather and analyze the target bands, respectively.

### 4.5. Enzyme-Linked Immunosorbent Assay (ELISA)

Following the instructions of the ELISA kit (Sigma-Aldrich, St. Louis, MO, USA), the conditioned media obtained from the same treated cells as described in the Section 4.4 were collected to detect secreted target proteins, including VEGFA and VEGFC. The biotinylated HRP-TMB reaction generated color, which was quantified at 450 nm. A standard curve was used to calculate their values, which were then normalized to their total protein content.

### 4.6. Gelatin Zymography

The cell supernatant (collected from serum-free media after 48 h of incubation following a 2 h LPS-PG (1 μg/mL) pretreatment) and the conditioned media (obtained from the same treated cells as described in the Section 4.4) were collected to analyze gelatinase activity and secretion, respectively. SDS-PAGE containing 1% gelatin was used to separate equal amounts of protein from each sample. Gels were soaked in 2.5% Triton X-100 to remove SDS and then incubated in activation buffer overnight. Clear bands indicating gelatinase activity were shown by Coomassie Blue staining and destaining gels.

### 4.7. Adhesion Assay

Cell-ECM adhesion activity was measured using 96-well plates coated with Matrigel (Corning^®^, Corning, NY, USA) and fibronectin (Sigma-Aldrich, St. Louis, MO, USA). ESCC cells pretreated with LPS-PG (1 μg/mL for 2 h) were mixed 1:1 with the different tested conditions in serum-free media, seeded in duplicate into the coated plates, and incubated for 1 h at 37 °C. After incubation, non-adherent cells were removed. The remaining adherent cells were washed with serum-free media and quantified using the MTT assay.

### 4.8. Wound Assay

This technique was used to assess cell migration in 2D. ESCC cells were cultured in a 6-well plate until fully confluent and then pretreated with LPS-PG (1 µg/mL) for 2 h in serum-free media. Afterward, the LPS-PG was removed, and a sterile pipette tip was used to create a wound gap. Then, cells were incubated with various treatment conditions in serum-free media, and the initial wound distance was quantified at 0 h. Following incubation at 37 °C for 24 h, the wound distance was quantified again. The distance was measured using an inverted microscope (10×) and analyzed via the Zen imaging software version 3.10.

### 4.9. Transwell Migration and Invasion Assay

Chemoattractant-driven 3D cell movement was studied using a Transwell^®^ polycarbonate membrane (NEST Biotechnology, Wuxi, China) with a pore size of 8 µm. For cell preparation, cells were pretreated with LPS-PG (1 μg/mL) for 2 h, after which the LPS-PG-containing media was removed. These cells were then trypsinized and mixed with various concentrations of EIF or CGA in serum-free media at a 1:1 ratio. Next, FBS at 10% (chemoattractant) was added to the lower chamber, and a mixture of LPS-PG-pretreated ESCC cells under different test conditions was added to the top chamber. After a 24-h incubation at 37 °C, cell migration was assessed by staining the membrane with 1% crystal violet in 50% methanol, and then non-migrated cells were removed with a cotton swab. For detection, the membrane was cut and measured at 570 nm after soaking in 20% acetic acid. In the invasion assay, similar to the Transwell migration test, cells move through the extracellular matrix (ECM) using a Matrigel-coated membrane. After adding the mixture of LPS-PG-pretreated cells and different tested conditions to the upper chamber, cell invasion was likewise observed 24 h later.

### 4.10. Statistical Analysis

The data were derived from at least three independent biological replicates (*n* ≥ 3) and presented as the mean ± SD. The LPS mean was subsequently normalized to 100% for data presentation only. Statistical significance was assessed by one-way analysis of variance (ANOVA) using SPSS software version 21. The post hoc Bonferroni correction was used to account for multiple comparisons. Differences were considered statistically significant at *p* < 0.05 and *p* < 0.01.

## 5. Conclusions

In summary, the CGA-rich fraction from green coffee beans attenuated LPS-PG-stimulated inflammatory responses and cancer progression associated with NF-κB and p38 MAPK in ESCC, particularly in highly metastatic cells. As in our earlier study, the multi-component EIF yielded a more pronounced effect than pure CGA. This research also suggests that this fraction warrants further investigation as a potential combination with PTX-based therapy for ESCC, as the present study was conducted exclusively on highly metastatic T.T cells. Future in vivo studies and clinical trials remain essential to fully establish its clinical utility and enhance ESCC survival rates.

## Data Availability

The data presented in this study are available on request from the corresponding author due to institutional data sharing policies and to ensure the proper context and interpretation of the data. The supplementary figures supporting the findings of this study are available online alongside the main article.

## Supplementary Materials

The following supporting information can be downloaded at: https://www.mdpi.com/article/10.3390/ijms27219464/s1.
