# Supplementary material for: Chlorogenic Acid-Rich Fraction from Green Coffee Beans and Its Combination with Paclitaxel Attenuate Porphyromonas gingivalis LPS-Stimulated Esophageal Squamous Cell Carcinoma Related to the NF-ĸB and p38 Pathways
Source: Int J Mol Sci. 2026 Oct 23;27(21):9464. doi: 10.3390/ijms27219464 (PMC13762937; doi:10.3390/ijms27219464)

Supplementary material

Figure S1. Uncropped gelatin zymography gel showing gelatinase secretion in KE5 cells. The full gel image corresponds to the cropped data presented in Figure 4C.

(lane 1: NoLPS, lane 2: LPS-PG, lane 3: LPS-PG + EIF25, lane 4: LPS-PG + EIF50, lane 5: LPS-PG + EIF75, lane 6: LPS-PG + CGA5, lane 7: LPS-PG + CGA10, lane 8: LPS-PG + CGA20)

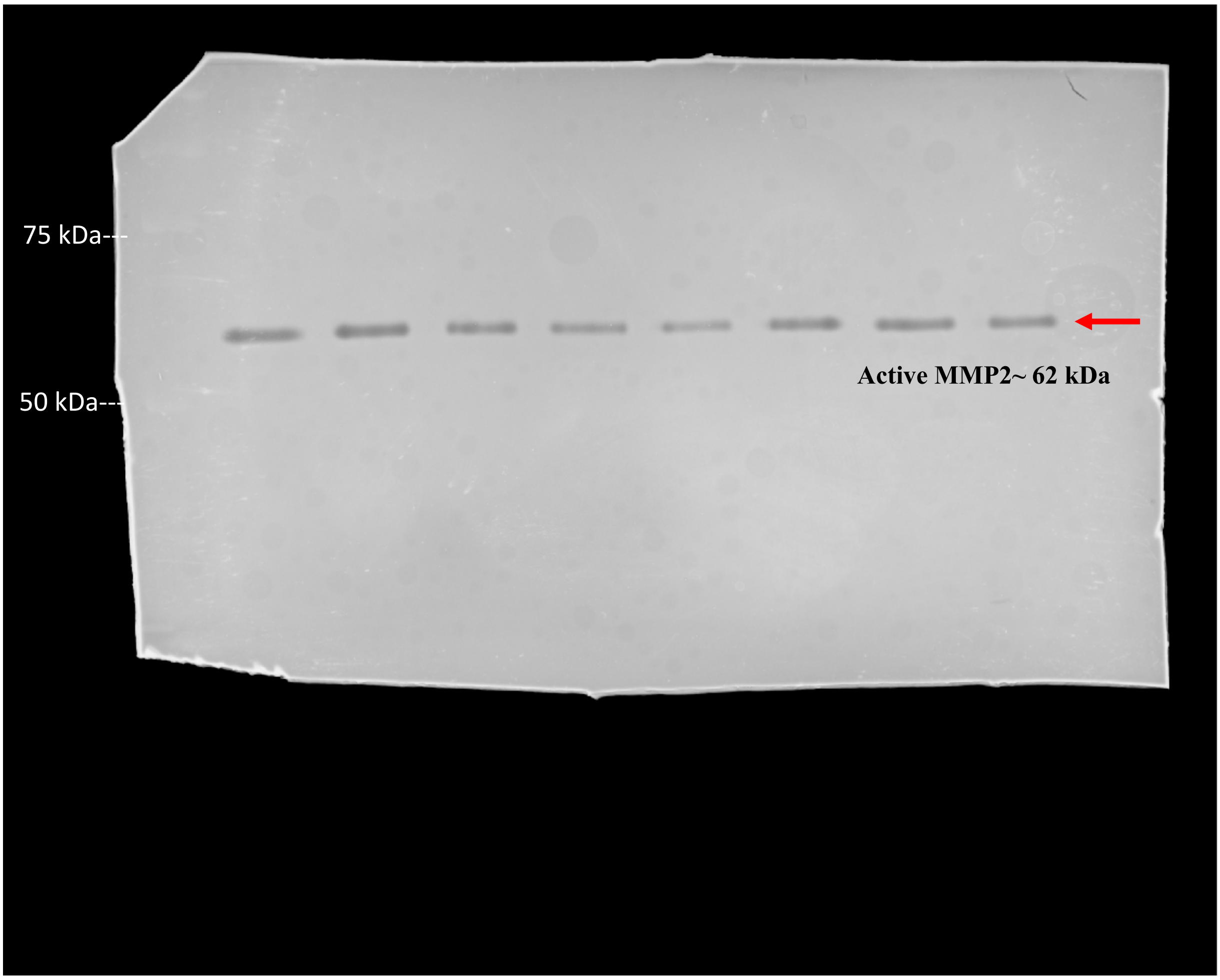

Figure S2. Uncropped gelatin zymography gel showing Gelatinase secretion of T.T cells. The full gel image corresponds to the cropped data presented in Figure 4C.

(lane 1: NoLPS, lane 2: LPS-PG, lane 3: LPS-PG + EIF25, lane 4: LPS-PG + EIF50, lane 5: LPS-PG + EIF75, lane 6: LPS-PG + CGA5, lane 7: LPS-PG + CGA10, lane 8: LPS-PG + CGA20)

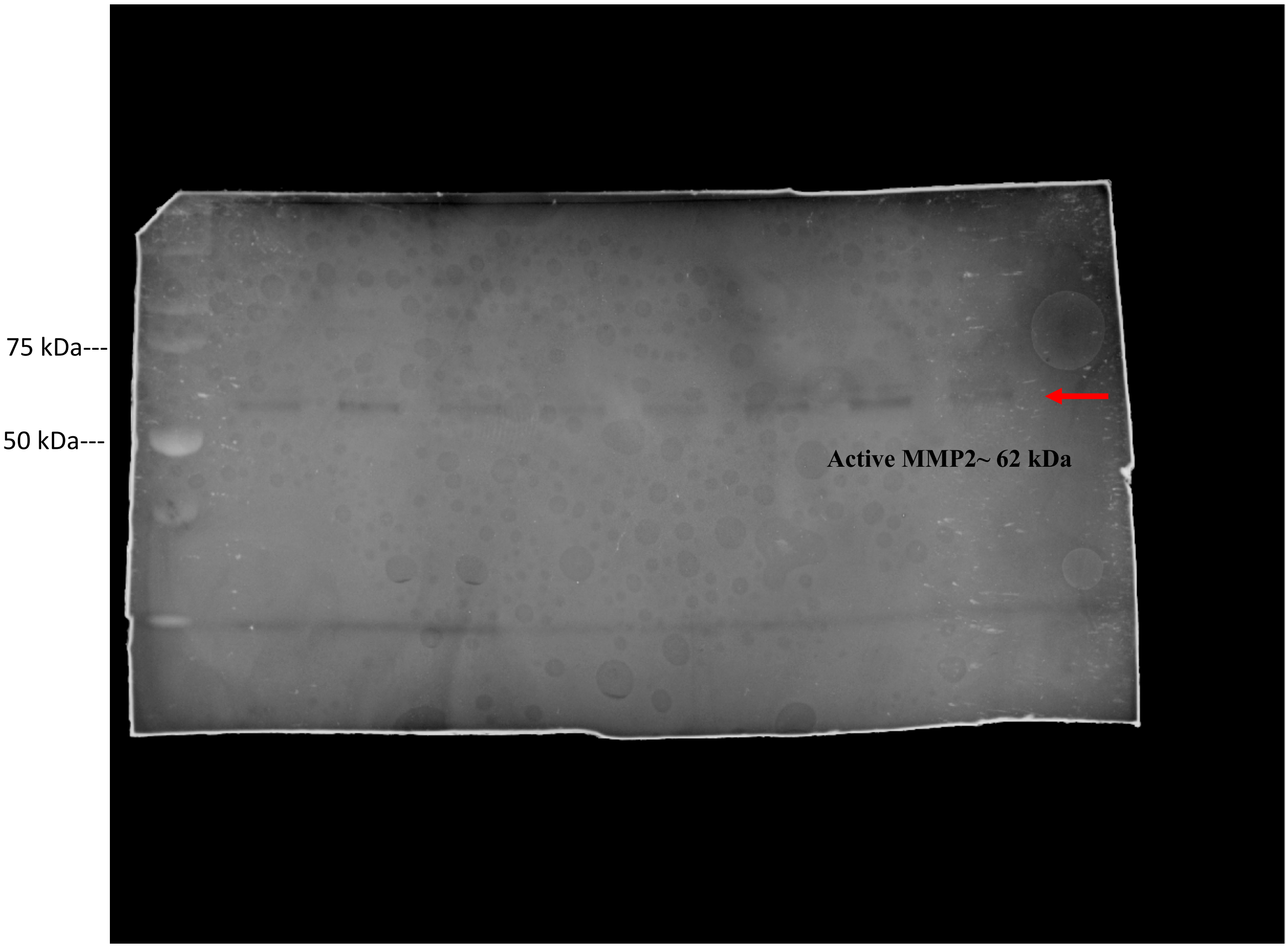

Figure S3. Uncropped gelatin zymography gel showing Gelatinase activity of KE5 cells treated with EIF. The full gel image corresponds to the cropped data presented in Figure 4D.

(lane 1: cont., lane 2: VC, lane 3: EIF25, lane 4: EIF50, lane 5: EIF75)

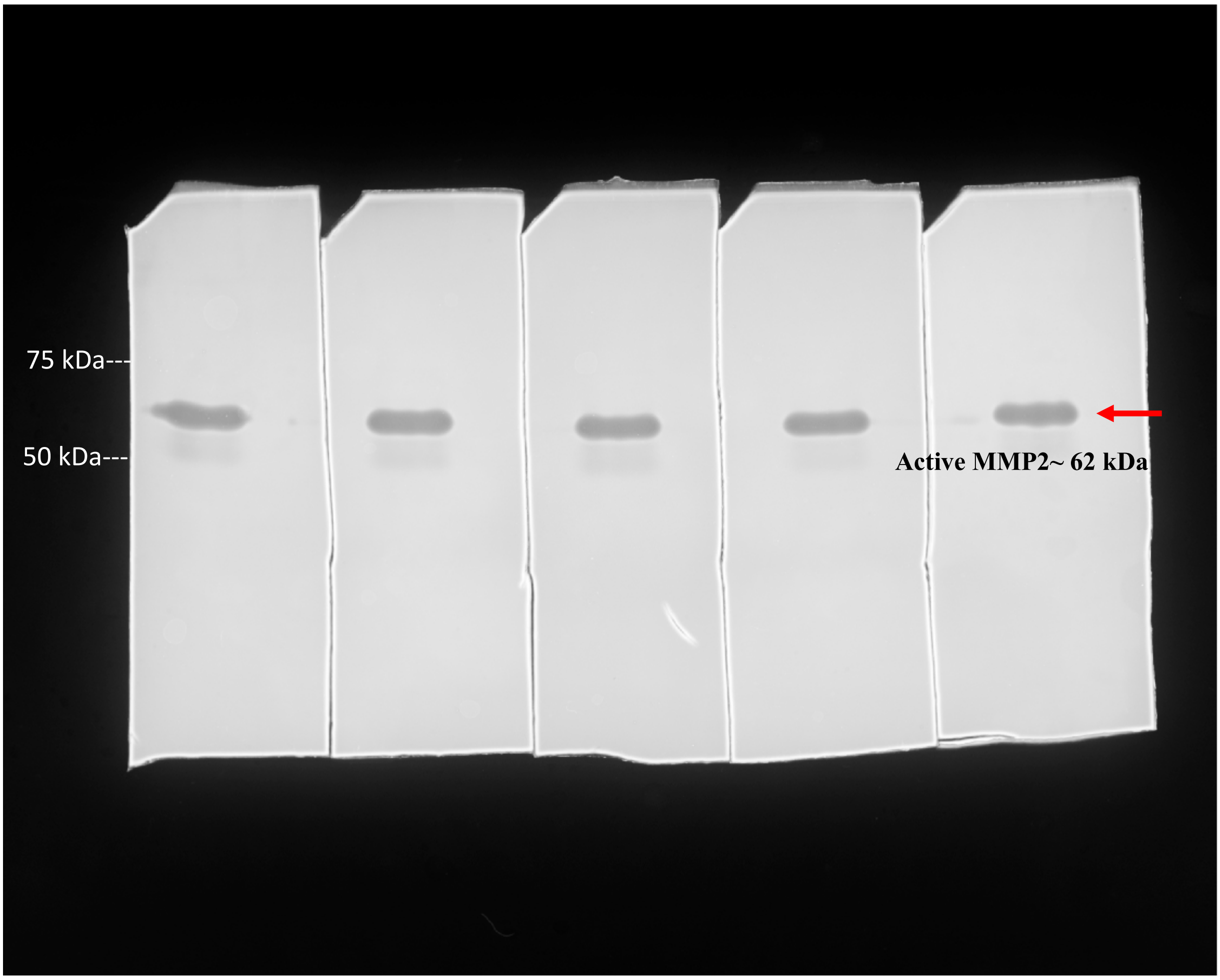

Figure S4. Uncropped gelatin zymography gel showing Gelatinase activity of T.T cells treated with EIF. The full gel image corresponds to the cropped data presented in Figure 4D.

(lane 1: cont., lane 2: VC, lane 3: EIF25, lane 4: EIF50, lane 5: EIF75)

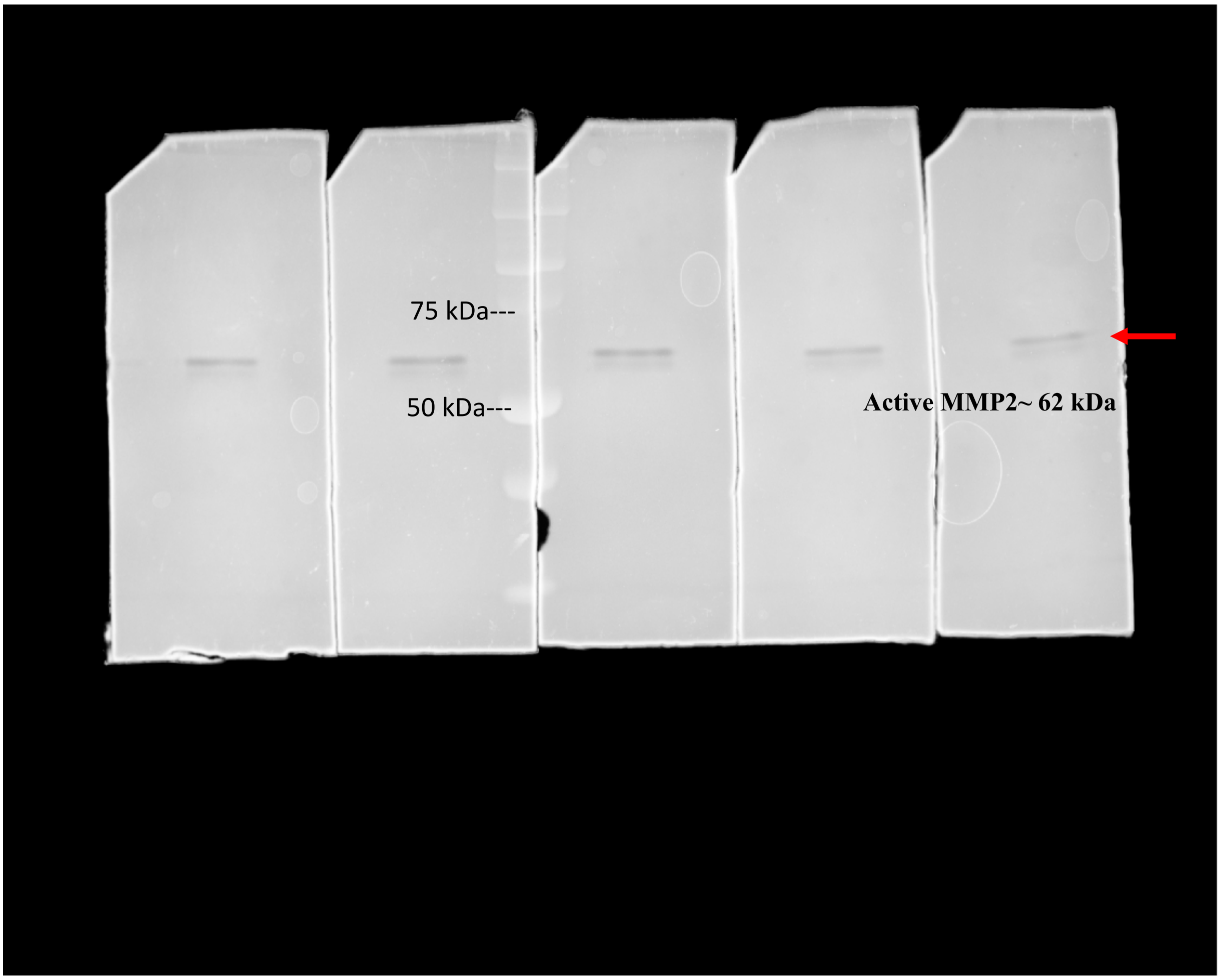

Figure S5. Uncropped gelatin zymography gel showing Gelatinase activity of KE5 cells treated with CGA. The full gel image corresponds to the cropped data presented in Figure 4E.

(lane 1: cont., lane 2: VC, lane 3: CGA5, lane 4: CGA10, lane 5: CGA20)

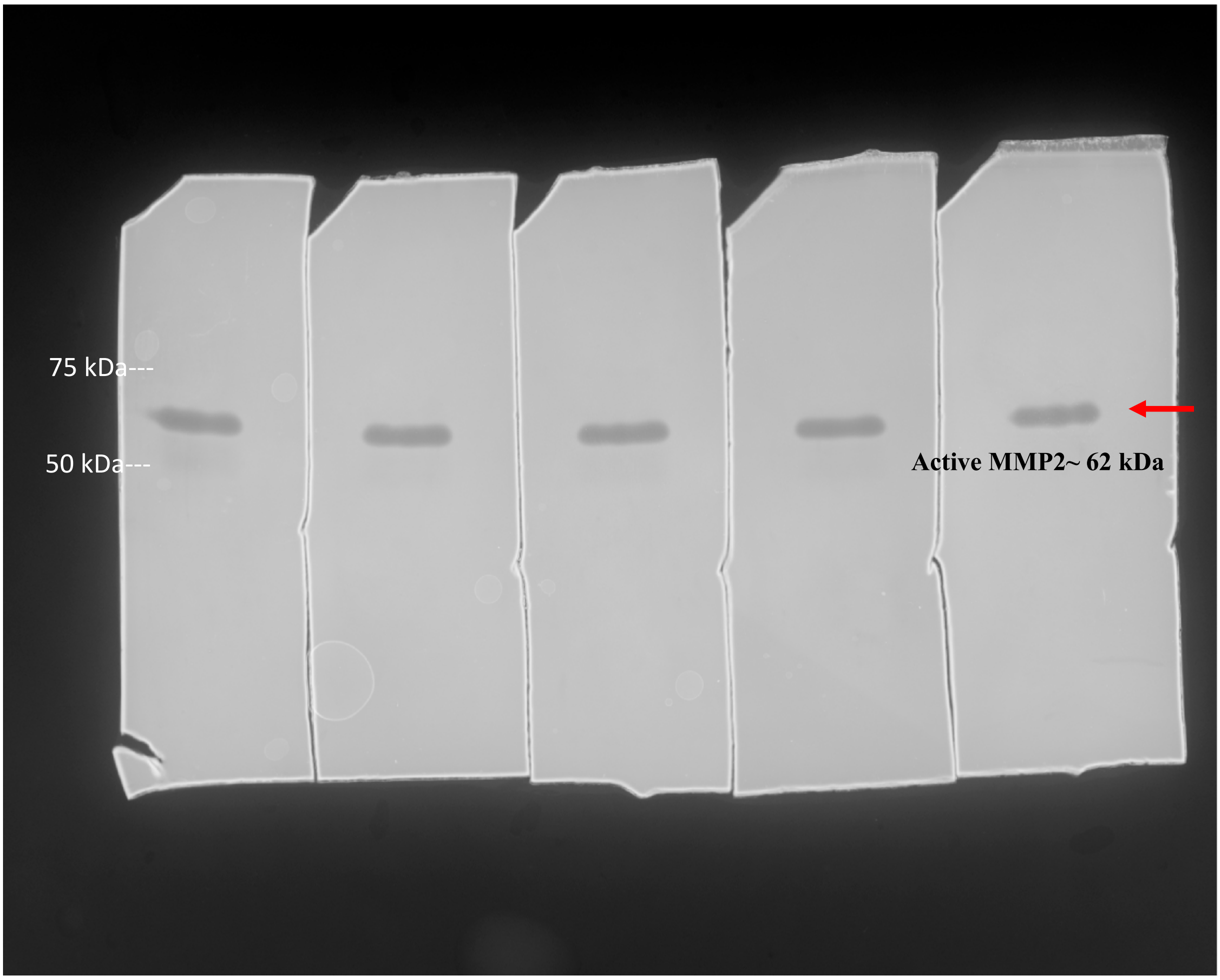

Figure S6. Uncropped gelatin zymography gel showing Gelatinase activity of T.T cells treated with CGA. The full gel image corresponds to the cropped data presented in Figure 4E.

(lane 1: cont., lane 2: VC, lane 3: CGA5, lane 4: CGA10, lane 5: CGA20)

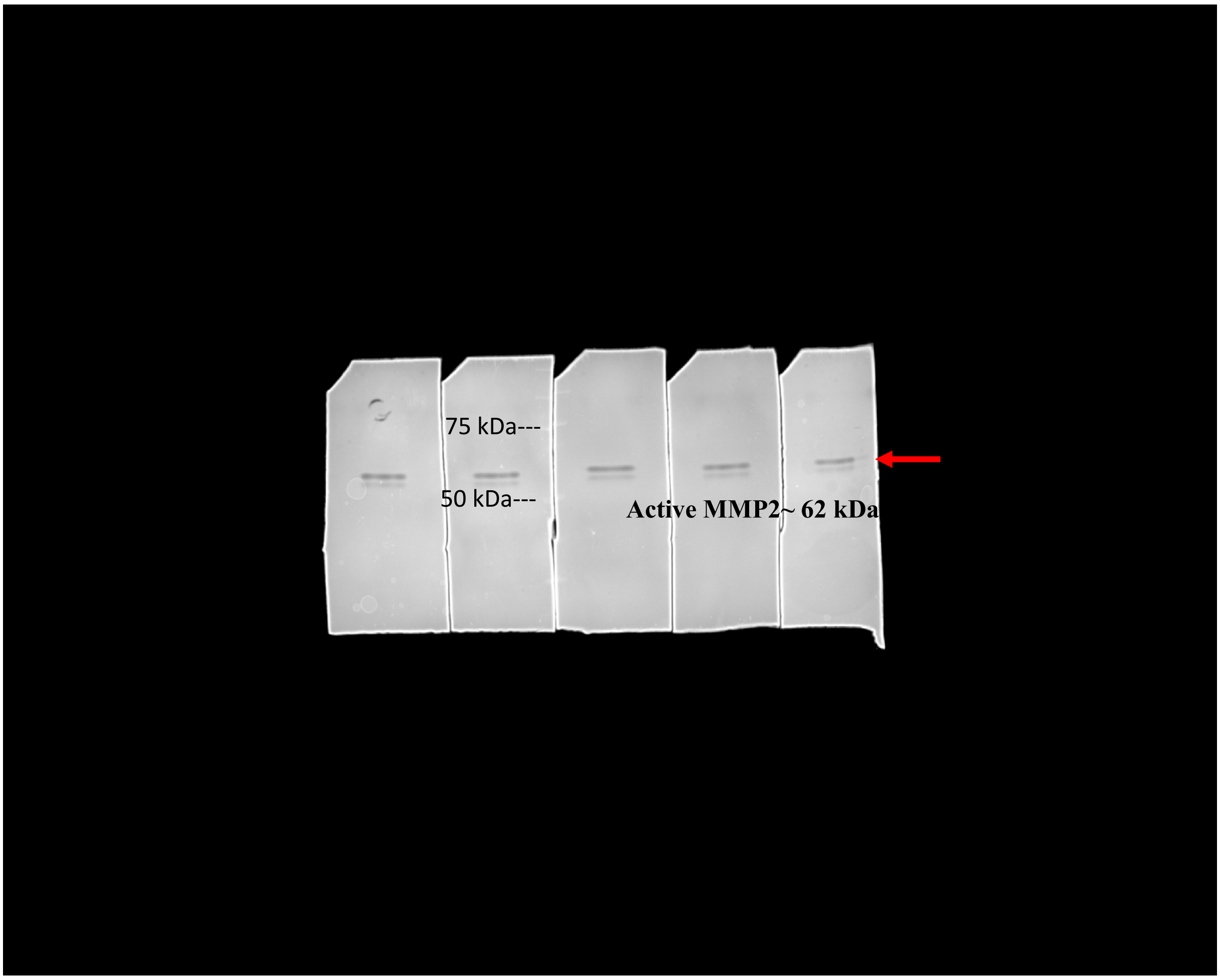

Figure S7. Uncropped PVDF membrane showing TLR4 in KE5 cells. The raw image corresponds to the cropped data presented in Figure 2A.

Note: The membrane was sectioned according to the expected molecular weights of the target proteins prior to antibody incubation to enable the detection of multiple proteins from the same membrane. Representative blot images from the corresponding membrane sections are shown.

TLR4 of KE5 cells (lane 1: NoLPS, lane 2: LPS-PG, lane 3: LPS-PG + EIF25, lane 4: LPS-PG + EIF50, lane 5: LPS-PG + EIF75, lane 6: LPS-PG + CGA5, lane 7: LPS-PG + CGA10, lane 8: LPS-PG + CGA20)

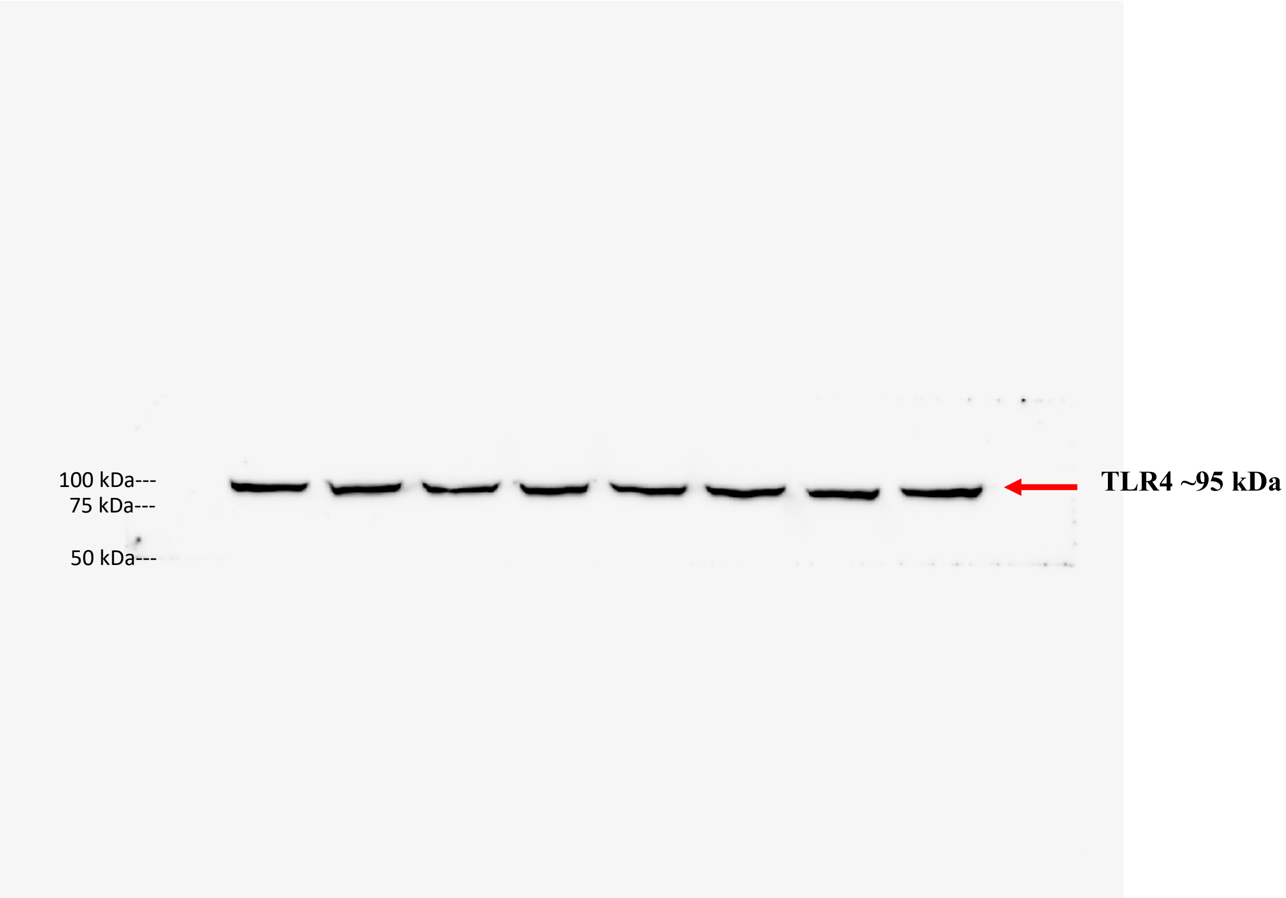

Figure S8. Uncropped PVDF membrane showing β-actin in KE5 cells. The raw image corresponds to the cropped data presented in Figure 2A.

Note: The membrane was sectioned according to the expected molecular weights of the target proteins before antibody incubation to enable the detection of multiple proteins from the same membrane. Representative blot images from the corresponding membrane sections are shown.

β-actin of KE5 cells (lane 1: NoLPS, lane 2: LPS-PG, lane 3: LPS-PG + EIF25, lane 4: LPS-PG + EIF50, lane 5: LPS-PG + EIF75, lane 6: LPS-PG + CGA5, lane 7: LPS-PG + CGA10, lane 8: LPS-PG + CGA20)

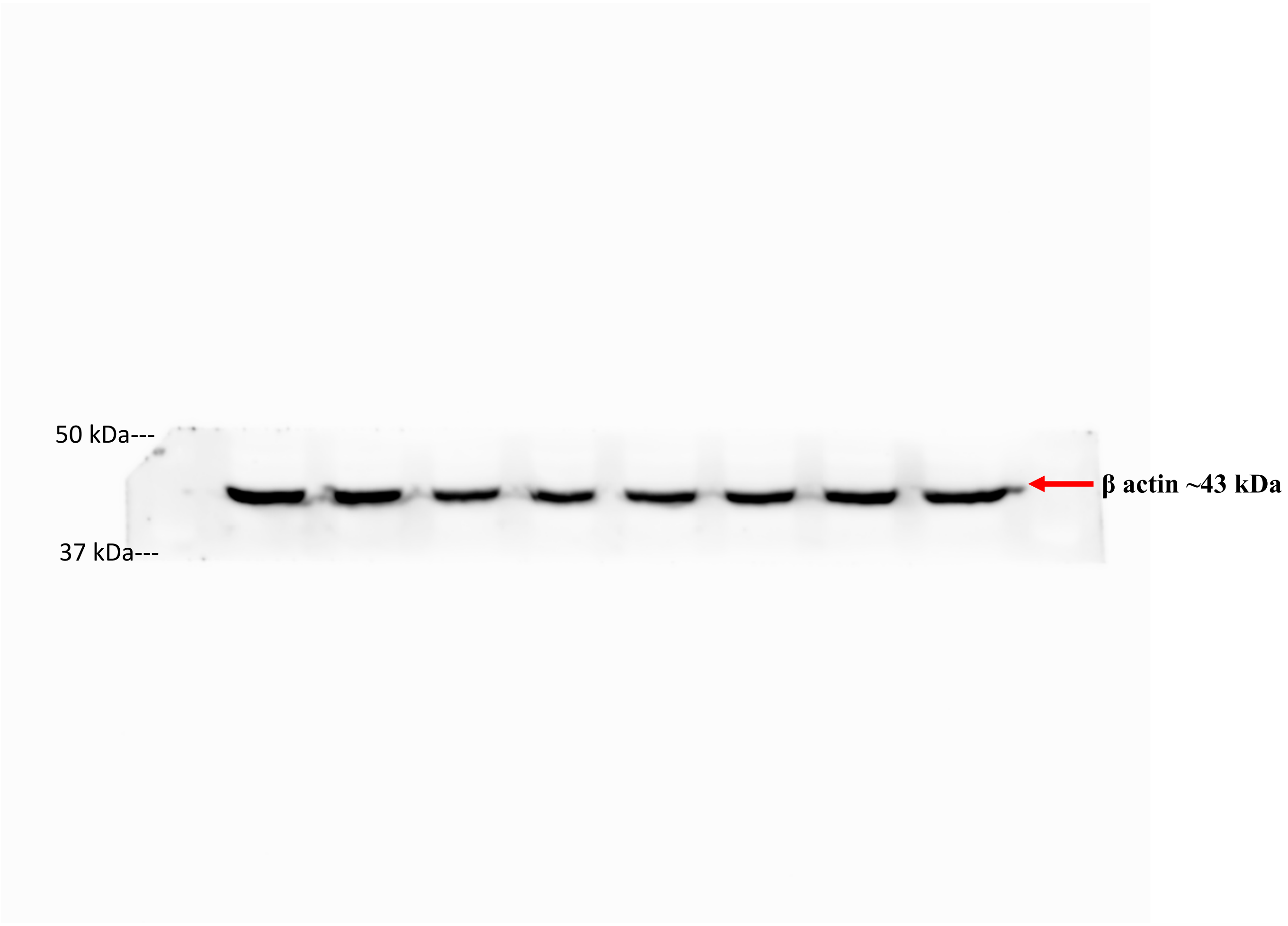

Figure S9. Uncropped PVDF membrane showing COX2 in KE5 cells. The raw image corresponds to the cropped data presented in Figure 2A.

Note: The membrane was sectioned according to the expected molecular weights of the target proteins before antibody incubation to enable the detection of multiple proteins from the same membrane. Representative blot images from the corresponding membrane sections are shown.

COX2 of KE5 cells (lane 1: NoLPS, lane 2: LPS-PG, lane 3: LPS-PG + EIF25, lane 4: LPS-PG + EIF50, lane 5: LPS-PG + EIF75, lane 6: LPS-PG + CGA5, lane 7: LPS-PG + CGA10, lane 8: LPS-PG + CGA20)

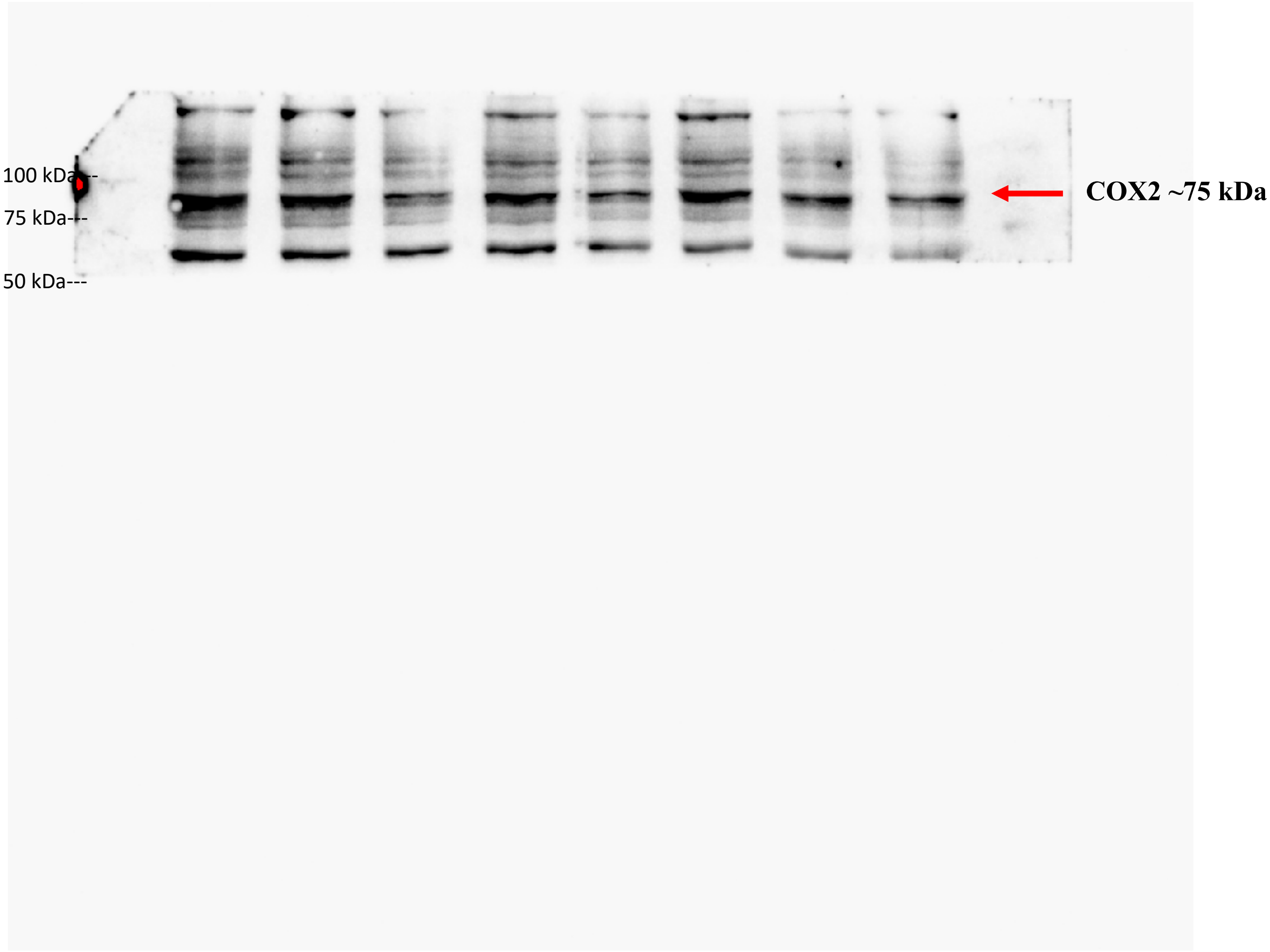

Figure S10. Uncropped PVDF membrane showing β-actin in KE5 cells. The raw image corresponds to the cropped data presented in Figure 2A.

Note: The membrane was sectioned according to the expected molecular weights of the target proteins before antibody incubation to enable the detection of multiple proteins from the same membrane. Representative blot images from the corresponding membrane sections are shown.

β-actin of KE5 cells (lane 1: NoLPS, lane 2: LPS-PG, lane 3: LPS-PG + EIF25, lane 4: LPS-PG + EIF50, lane 5: LPS-PG + EIF75, lane 6: LPS-PG + CGA5, lane 7: LPS-PG + CGA10, lane 8: LPS-PG + CGA20)

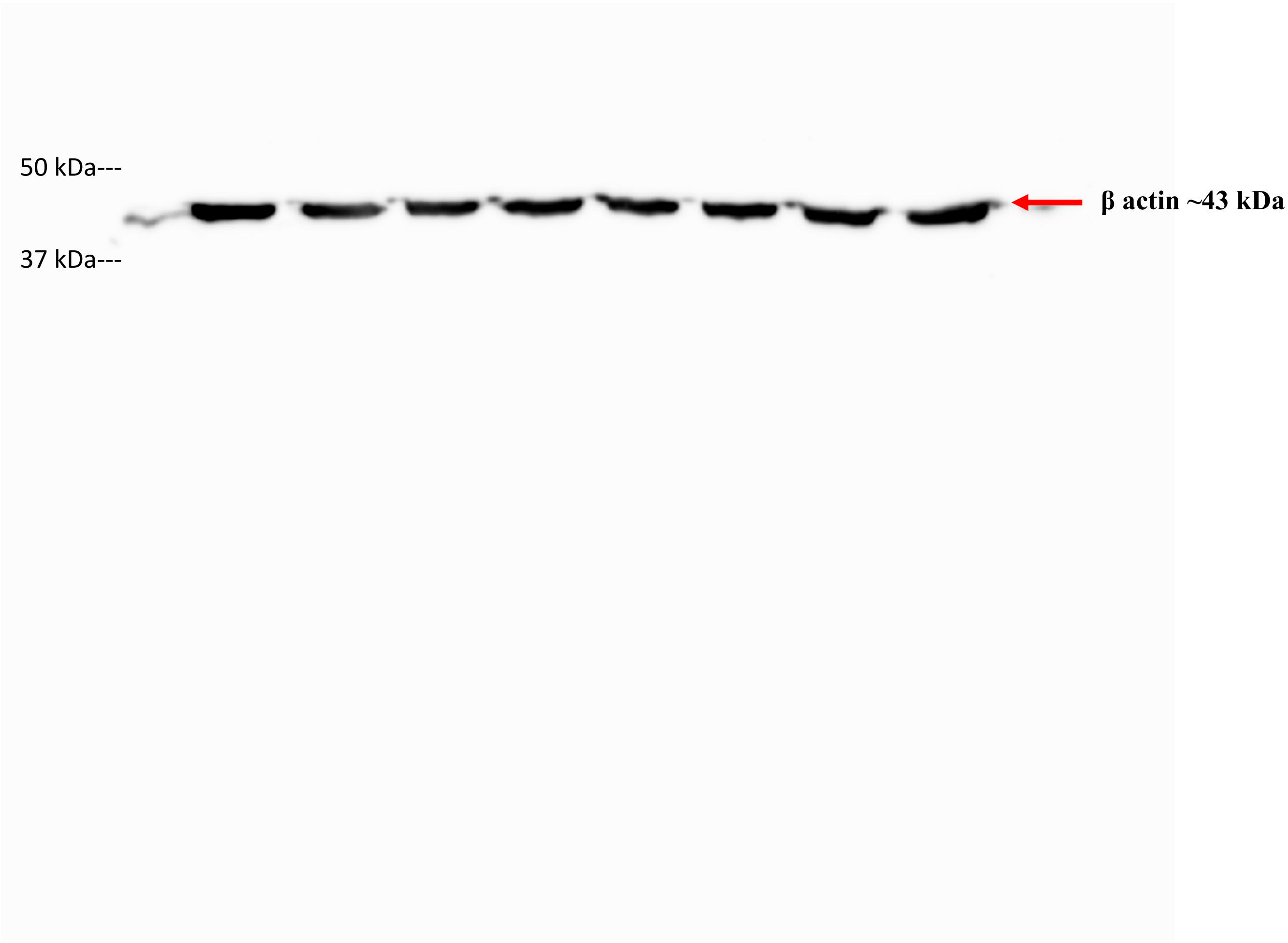

Figure S11. Uncropped PVDF membrane showing IL-1β in KE5 cells. The raw image corresponds to the cropped data presented in Figure 2A.

Note: The membrane was sectioned according to the expected molecular weights of the target proteins before antibody incubation to enable the detection of multiple proteins from the same membrane. Representative blot images from the corresponding membrane sections are shown.

IL-1β of KE5 cells (lane 1: NoLPS, lane 2: LPS-PG, lane 3: LPS-PG + EIF25, lane 4: LPS-PG + EIF50, lane 5: LPS-PG + EIF75, lane 6: LPS-PG + CGA5, lane 7: LPS-PG + CGA10, lane 8: LPS-PG + CGA20)

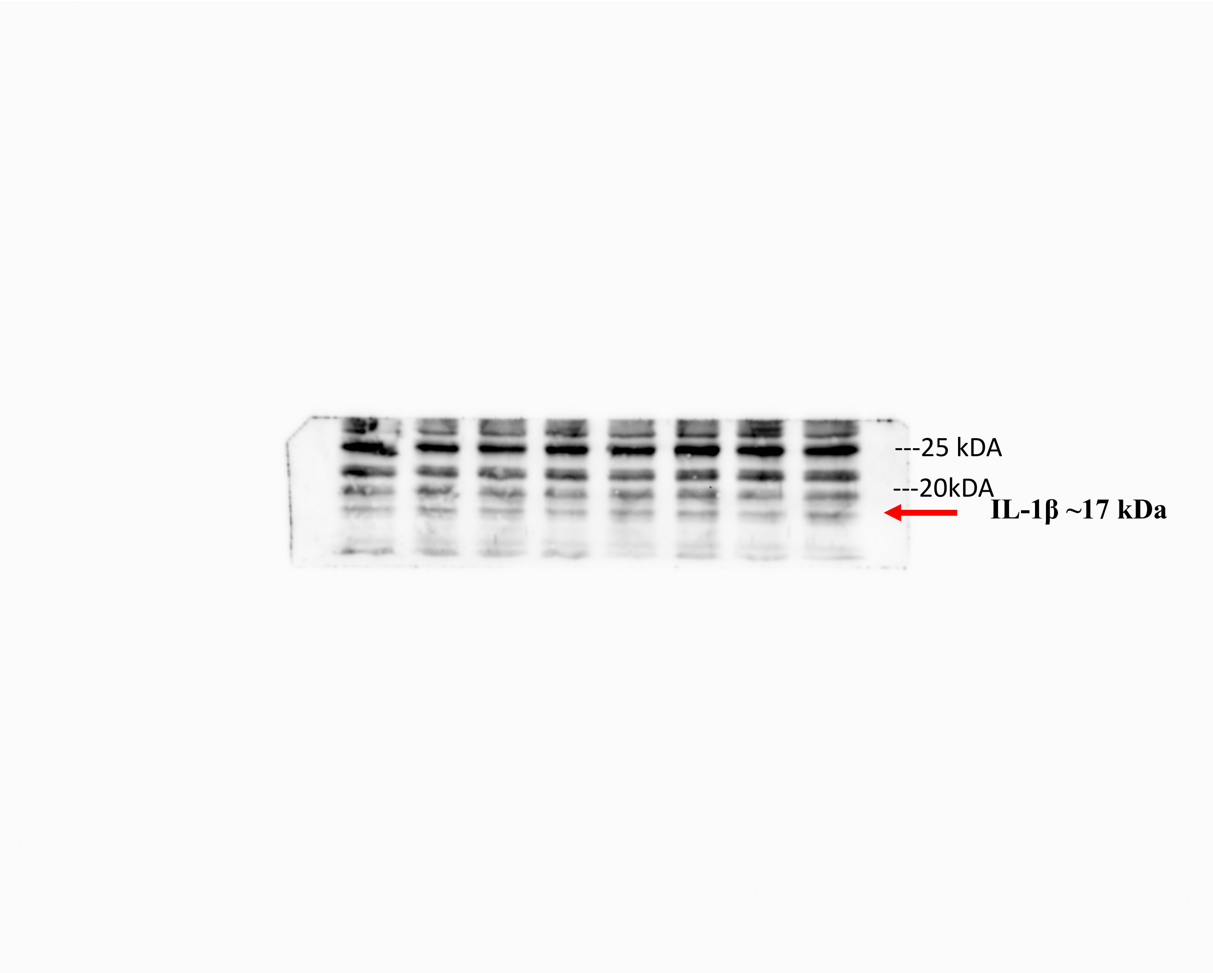

Figure S12. Uncropped PVDF membrane showing β-actin in KE5 cells. The raw image corresponds to the cropped data presented in Figure 2A.

Note: The membrane was sectioned according to the expected molecular weights of the target proteins before antibody incubation to enable the detection of multiple proteins from the same membrane. Representative blot images from the corresponding membrane sections are shown.

β-actin of KE5 cells (lane 1: NoLPS, lane 2: LPS-PG, lane 3: LPS-PG + EIF25, lane 4: LPS-PG + EIF50, lane 5: LPS-PG + EIF75, lane 6: LPS-PG + CGA5, lane 7: LPS-PG + CGA10, lane 8: LPS-PG + CGA20)

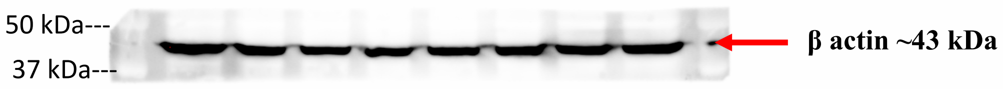

Figure S13. Uncropped PVDF membrane showing TNF-α in KE5 cells. The raw image corresponds to the cropped data presented in Figure 2A.

Note: The membrane was sectioned according to the expected molecular weights of the target proteins before antibody incubation to enable the detection of multiple proteins from the same membrane. Representative blot images from the corresponding membrane sections are shown.

TNF-α of KE5 cells (lane 1: NoLPS, lane 2: LPS-PG, lane 3: LPS-PG + EIF25, lane 4: LPS-PG + EIF50, lane 5: LPS-PG + EIF75, lane 6: LPS-PG + CGA5, lane 7: LPS-PG + CGA10, lane 8: LPS-PG + CGA20)

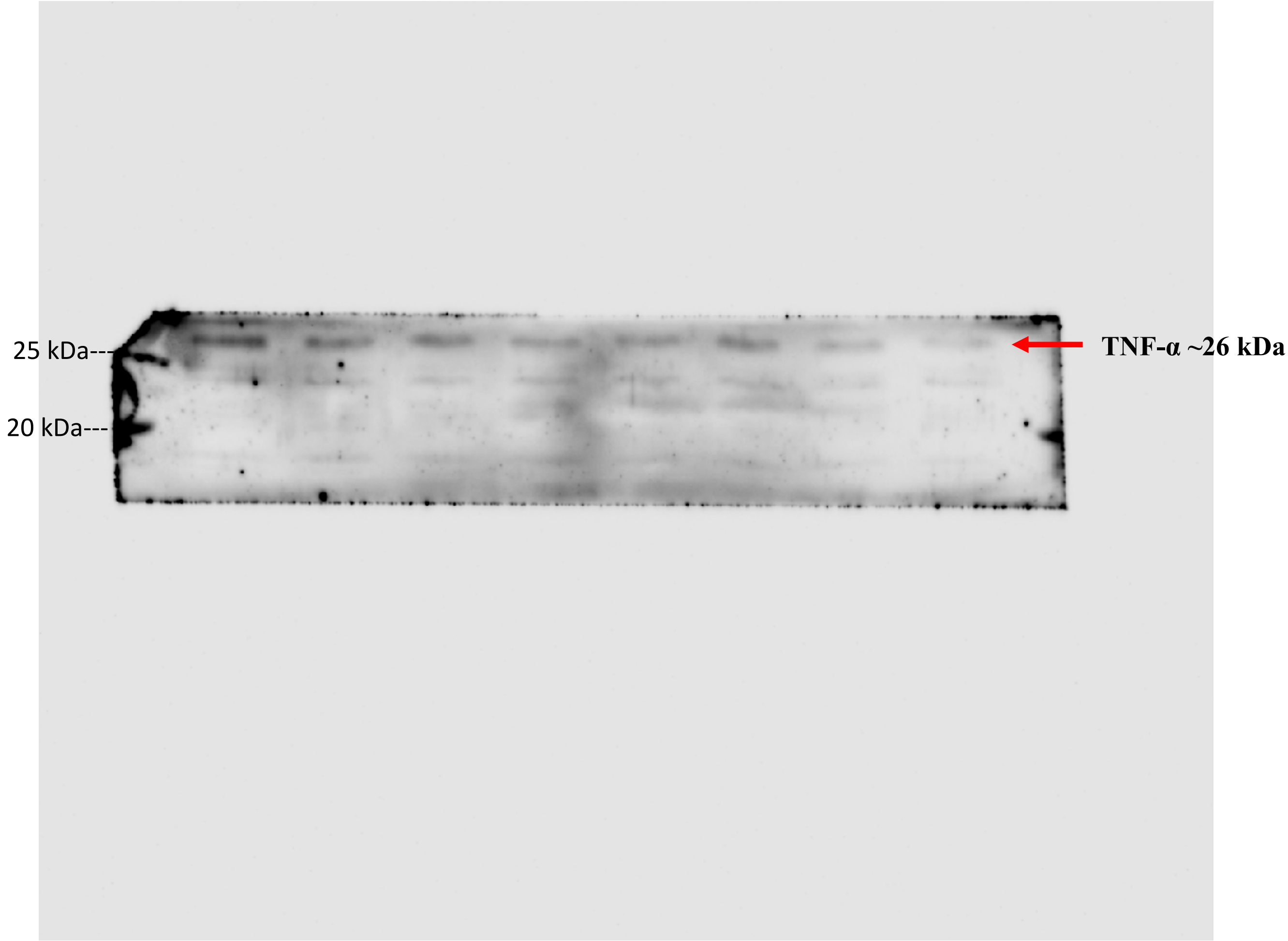

Figure S14. Uncropped PVDF membrane showing β-actin in KE5 cells. The raw image corresponds to the cropped data presented in Figure 2A.

Note: The membrane was sectioned according to the expected molecular weights of the target proteins before antibody incubation to enable the detection of multiple proteins from the same membrane. Representative blot images from the corresponding membrane sections are shown.

β-actin of KE5 cells (lane 1: NoLPS, lane 2: LPS-PG, lane 3: LPS-PG + EIF25, lane 4: LPS-PG + EIF50, lane 5: LPS-PG + EIF75, lane 6: LPS-PG + CGA5, lane 7: LPS-PG + CGA10, lane 8: LPS-PG + CGA20)

The identical β-actin band was utilized as a shared loading control for both TNF-α and uPAR of KE5 cells, which were analyzed on the same membrane using cutting and stripping/reprobing methods to ensure accurate normalization.

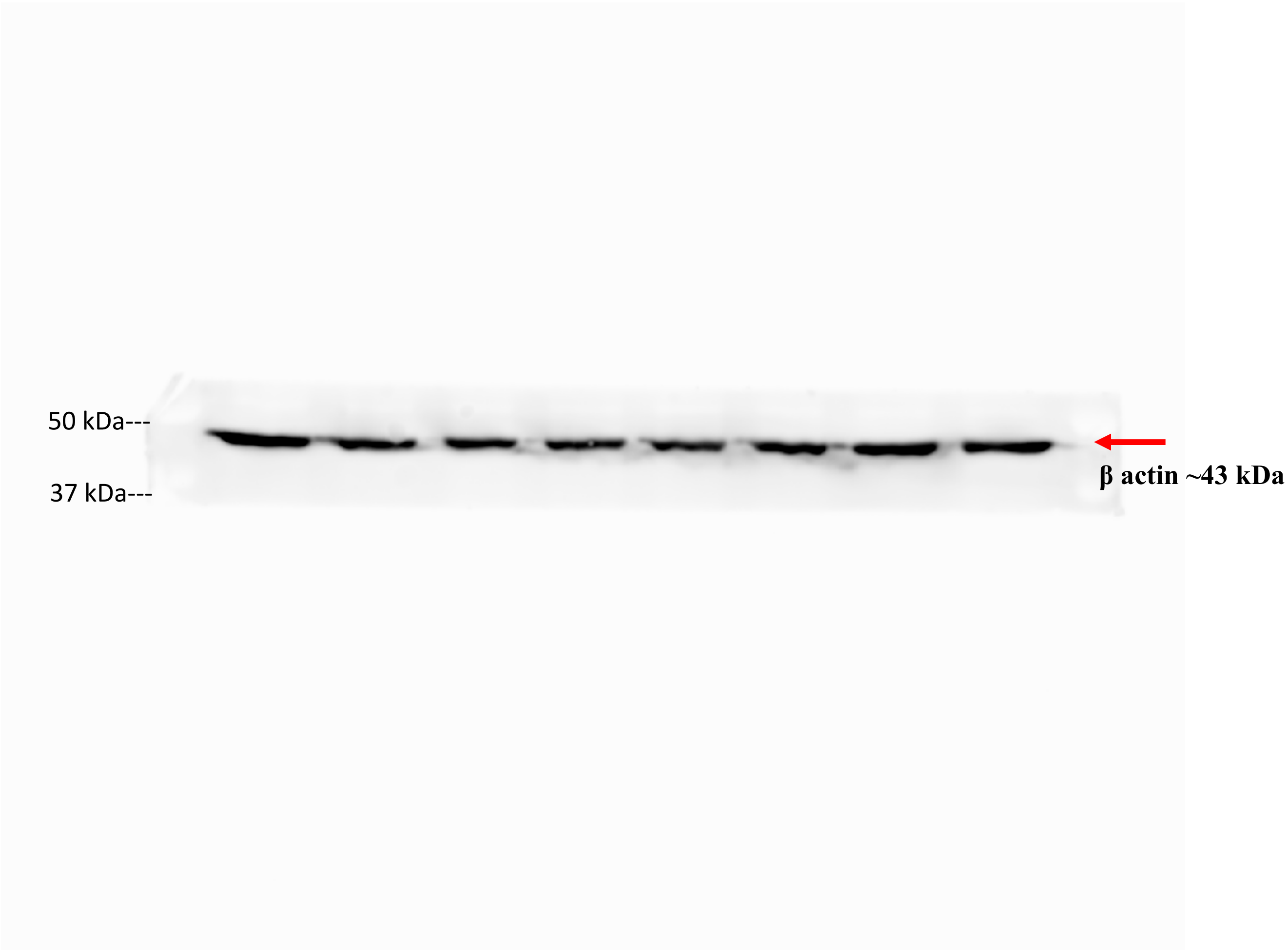

Figure S15. Uncropped PVDF membrane showing TLR4 in T.T cells. The raw image corresponds to the cropped data presented in Figure 2B.

Note: The membrane was sectioned according to the expected molecular weights of the target proteins before antibody incubation to enable the detection of multiple proteins from the same membrane. Representative blot images from the corresponding membrane sections are shown.

TLR4 of T.T cells (lane 1: NoLPS, lane 2: LPS-PG, lane 3: LPS-PG + EIF25, lane 4: LPS-PG + EIF50, lane 5: LPS-PG + EIF75, lane 6: LPS-PG + CGA5, lane 7: LPS-PG + CGA10, lane 8: LPS-PG + CGA20)

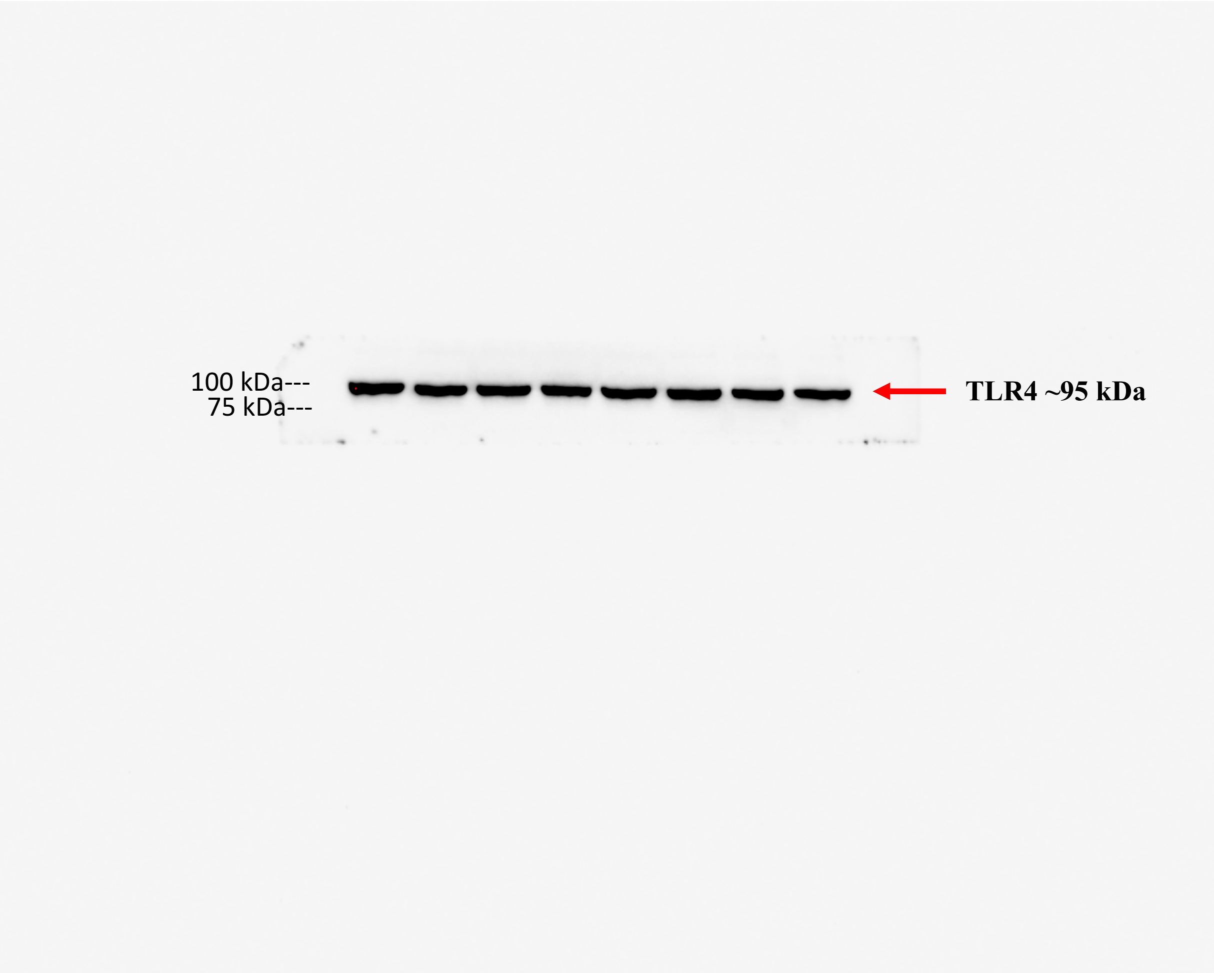

Figure S16. Uncropped PVDF membrane showing β-actin inT.T cells. The raw image corresponds to the cropped data presented in Figure 2B.

Note: The membrane was sectioned according to the expected molecular weights of the target proteins before antibody incubation to enable the detection of multiple proteins from the same membrane. Representative blot images from the corresponding membrane sections are shown.

β-actin of T.T cells (lane 1: NoLPS, lane 2: LPS-PG, lane 3: LPS-PG + EIF25, lane 4: LPS-PG + EIF50, lane 5: LPS-PG + EIF75, lane 6: LPS-PG + CGA5, lane 7: LPS-PG + CGA10, lane 8: LPS-PG + CGA20)

The identical β-actin band was utilized as a shared loading control for TLR4 and COX2 of T.T, which were analyzed on the same membrane using cutting and stripping/reprobing methods to ensure accurate normalization.

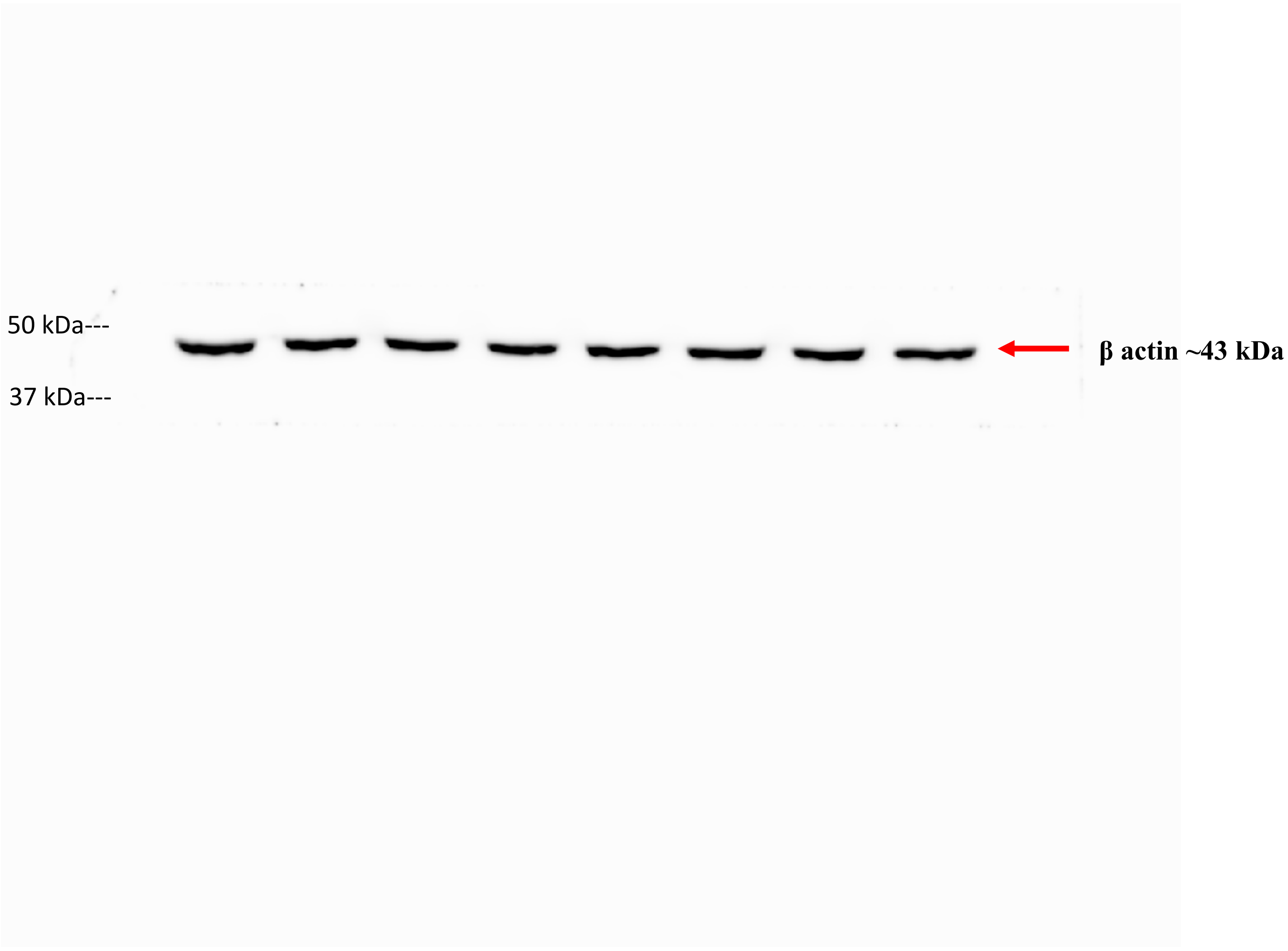

Figure S17. Uncropped PVDF membrane showing COX2 in T.T cells. The raw image corresponds to the cropped data presented in Figure 2B.

Note: The membrane was sectioned according to the expected molecular weights of the target proteins before antibody incubation to enable the detection of multiple proteins from the same membrane. Representative blot images from the corresponding membrane sections are shown.

COX2 of T.T cells (lane 1: NoLPS, lane 2: LPS-PG, lane 3: LPS-PG + EIF25, lane 4: LPS-PG + EIF50, lane 5: LPS-PG + EIF75, lane 6: LPS-PG + CGA5, lane 7: LPS-PG + CGA10, lane 8: LPS-PG + CGA20)

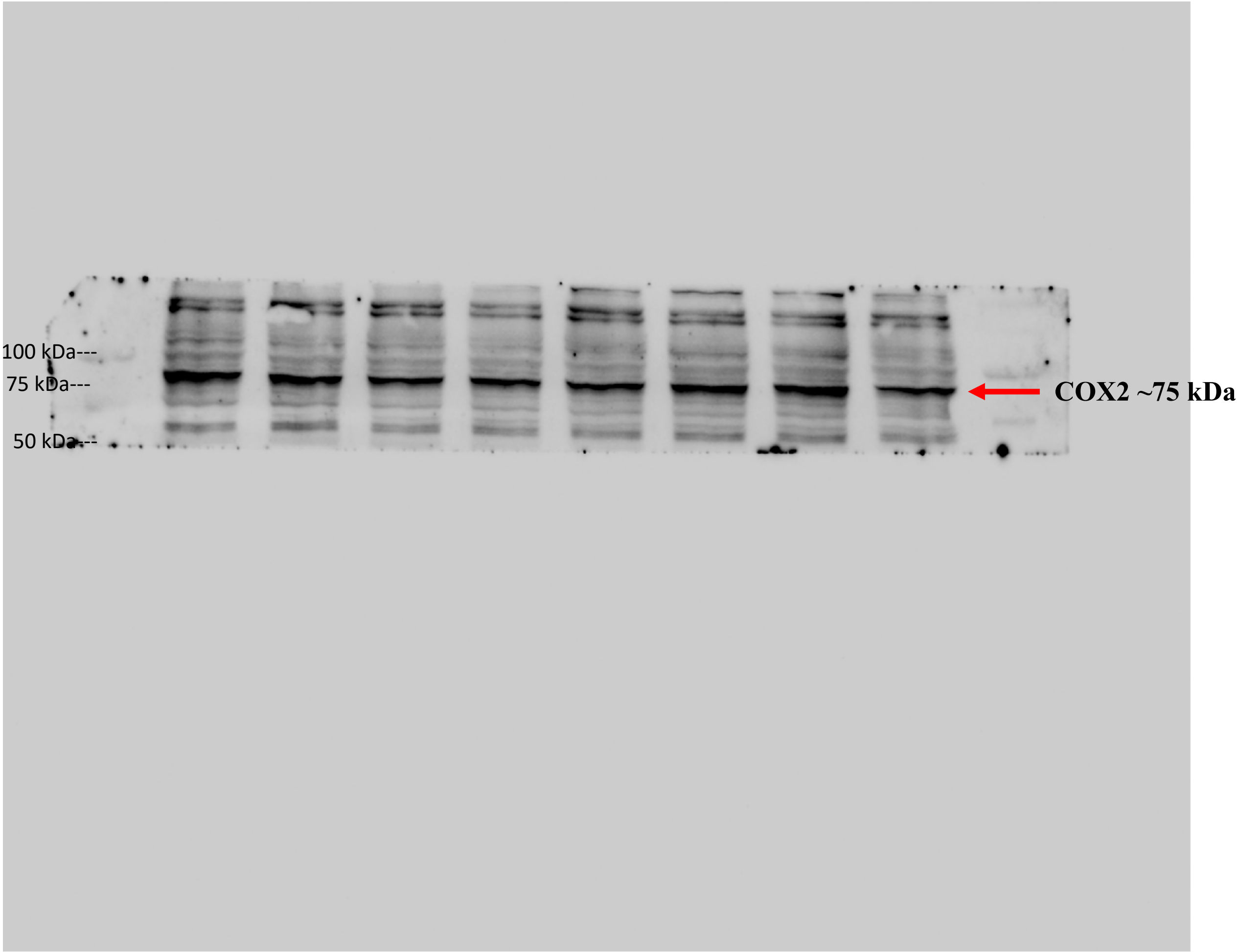

Figure S18. Uncropped PVDF membrane showing β-actin in T.T cells. The raw image corresponds to the cropped data presented in Figure 2B.

Note: The membrane was sectioned according to the expected molecular weights of the target proteins before antibody incubation to enable the detection of multiple proteins from the same membrane. Representative blot images from the corresponding membrane sections are shown.

β-actin of T.T cells (lane 1: NoLPS, lane 2: LPS-PG, lane 3: LPS-PG + EIF25, lane 4: LPS-PG + EIF50, lane 5: LPS-PG + EIF75, lane 6: LPS-PG + CGA5, lane 7: LPS-PG + CGA10, lane 8: LPS-PG + CGA20)

The identical β-actin band was utilized as a shared loading control for TLR4 and COX2 of T.T , which were analyzed on the same membrane using cutting and stripping/reprobing methods to ensure accurate normalization.

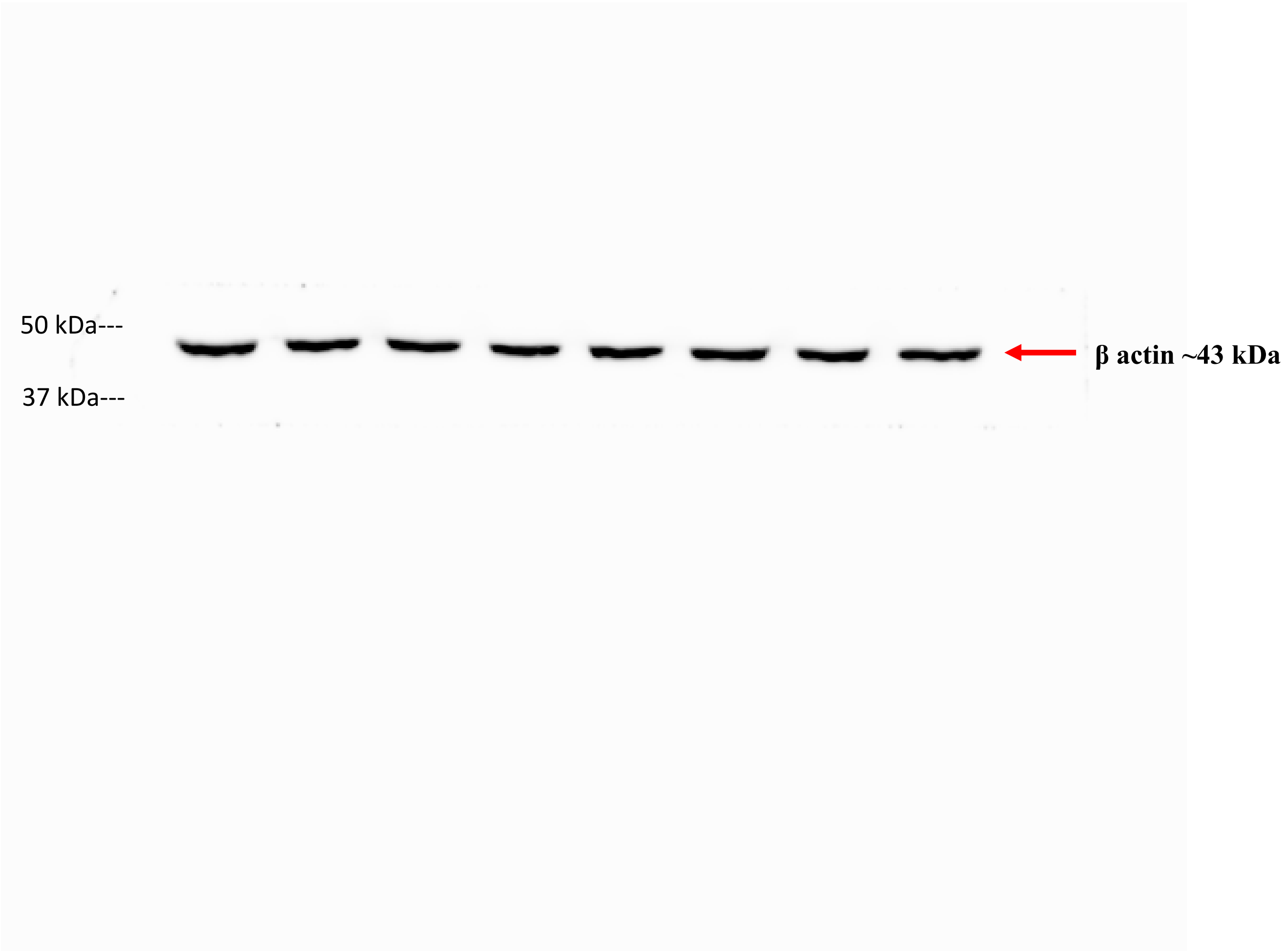

Figure S19. Uncropped PVDF membrane showing IL-1β in T.T cells. The raw image corresponds to the cropped data presented in Figure 2B.

Note: The membrane was sectioned according to the expected molecular weights of the target proteins before antibody incubation to enable the detection of multiple proteins from the same membrane. Representative blot images from the corresponding membrane sections are shown.

IL-1β of T.T cells (lane 1: NoLPS, lane 2: LPS-PG, lane 3: LPS-PG + EIF25, lane 4: LPS-PG + EIF50, lane 5: LPS-PG + EIF75, lane 6: LPS-PG + CGA5, lane 7: LPS-PG + CGA10, lane 8: LPS-PG + CGA20)

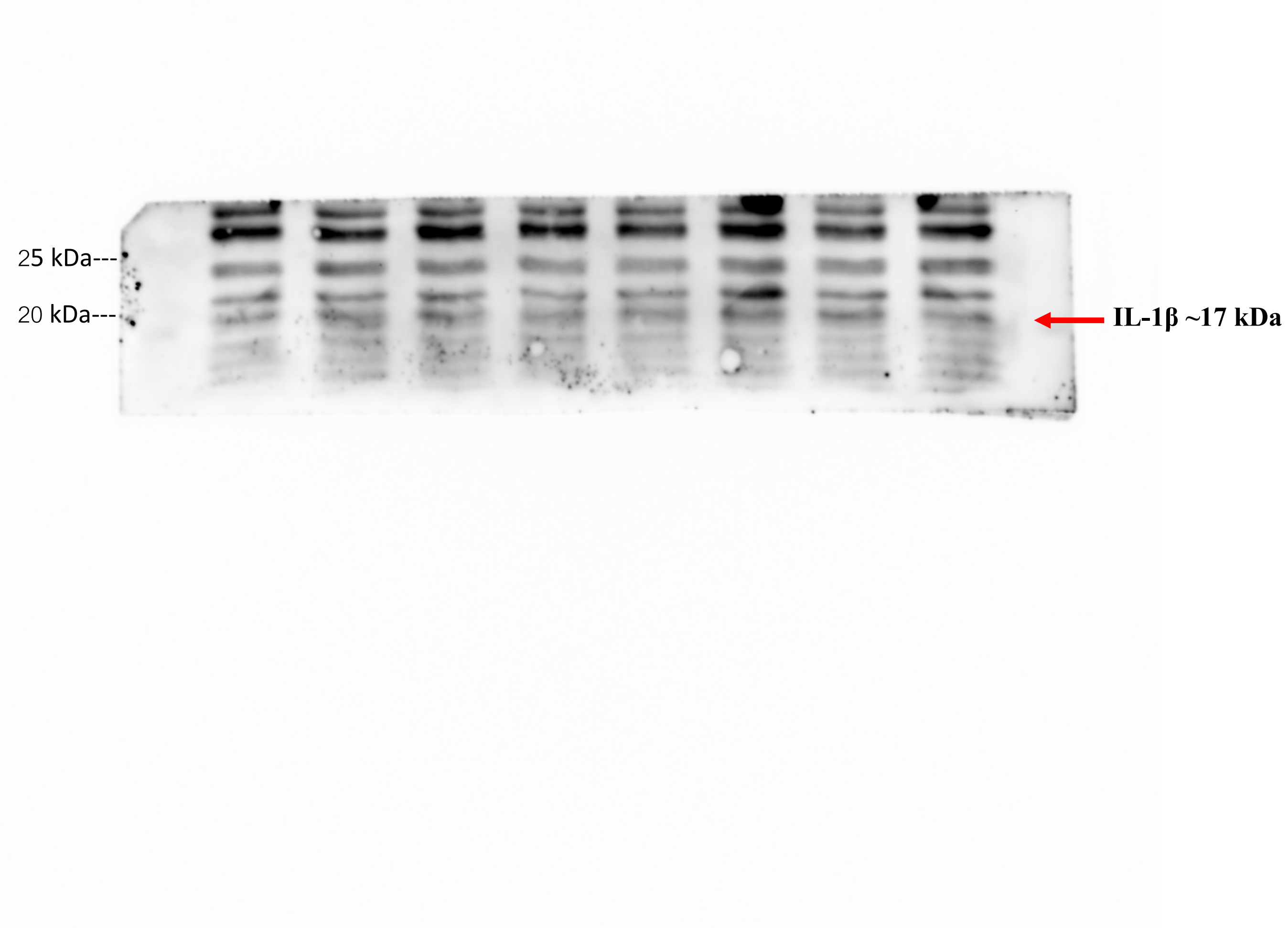

Figure S20. Uncropped PVDF membrane showing β-actin in T.T cells. The raw image corresponds to the cropped data presented in Figure 2B.

Note: The membrane was sectioned according to the expected molecular weights of the target proteins before antibody incubation to enable the detection of multiple proteins from the same membrane. Representative blot images from the corresponding membrane sections are shown.

β-actin of T.T cells (lane 1: NoLPS, lane 2: LPS-PG, lane 3: LPS-PG + EIF25, lane 4: LPS-PG + EIF50, lane 5: LPS-PG + EIF75, lane 6: LPS-PG + CGA5, lane 7: LPS-PG + CGA10, lane 8: LPS-PG + CGA20)

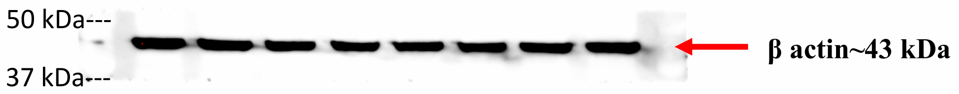

Figure S21. Uncropped PVDF membrane showing TNF-α in T.T cells. The raw image corresponds to the cropped data presented in Figure 2B.

Note: The membrane was sectioned according to the expected molecular weights of the target proteins before antibody incubation to enable the detection of multiple proteins from the same membrane. Representative blot images from the corresponding membrane sections are shown.

TNF-α of T.T cells (lane 1: NoLPS, lane 2: LPS-PG, lane 3: LPS-PG + EIF25, lane 4: LPS-PG + EIF50, lane 5: LPS-PG + EIF75, lane 6: LPS-PG + CGA5, lane 7: LPS-PG + CGA10, lane 8: LPS-PG + CGA20)

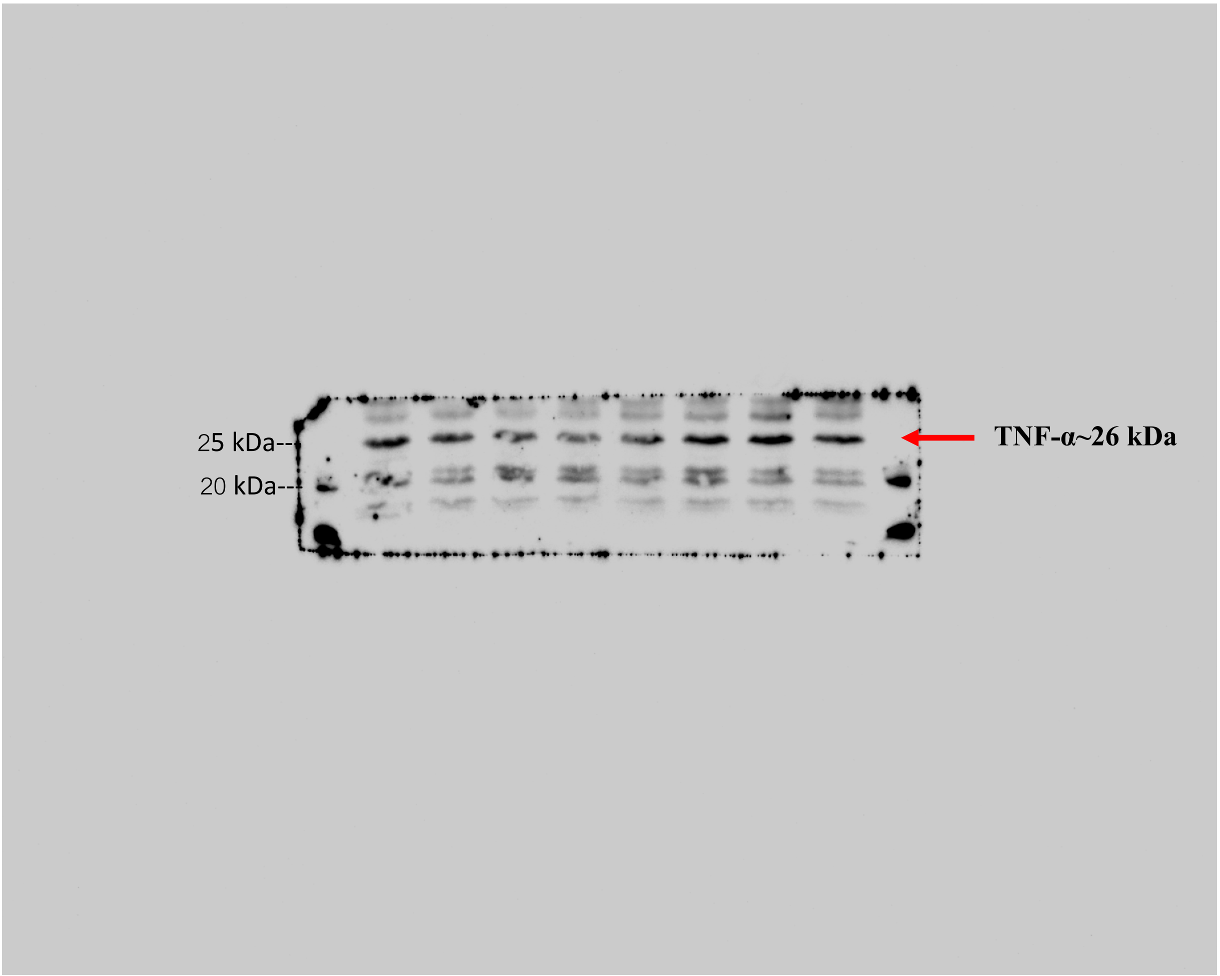

Figure S22. Uncropped PVDF membrane showing β-actin in T.T cells. The raw image corresponds to the cropped data presented in Figure 2B.

Note: The membrane was sectioned according to the expected molecular weights of the target proteins before antibody incubation to enable the detection of multiple proteins from the same membrane. Representative blot images from the corresponding membrane sections are shown.

β-actin of T.T cells (lane 1: NoLPS, lane 2: LPS-PG, lane 3: LPS-PG + EIF25, lane 4: LPS-PG + EIF50, lane 5: LPS-PG + EIF75, lane 6: LPS-PG + CGA5, lane 7: LPS-PG + CGA10, lane 8: LPS-PG + CGA20)

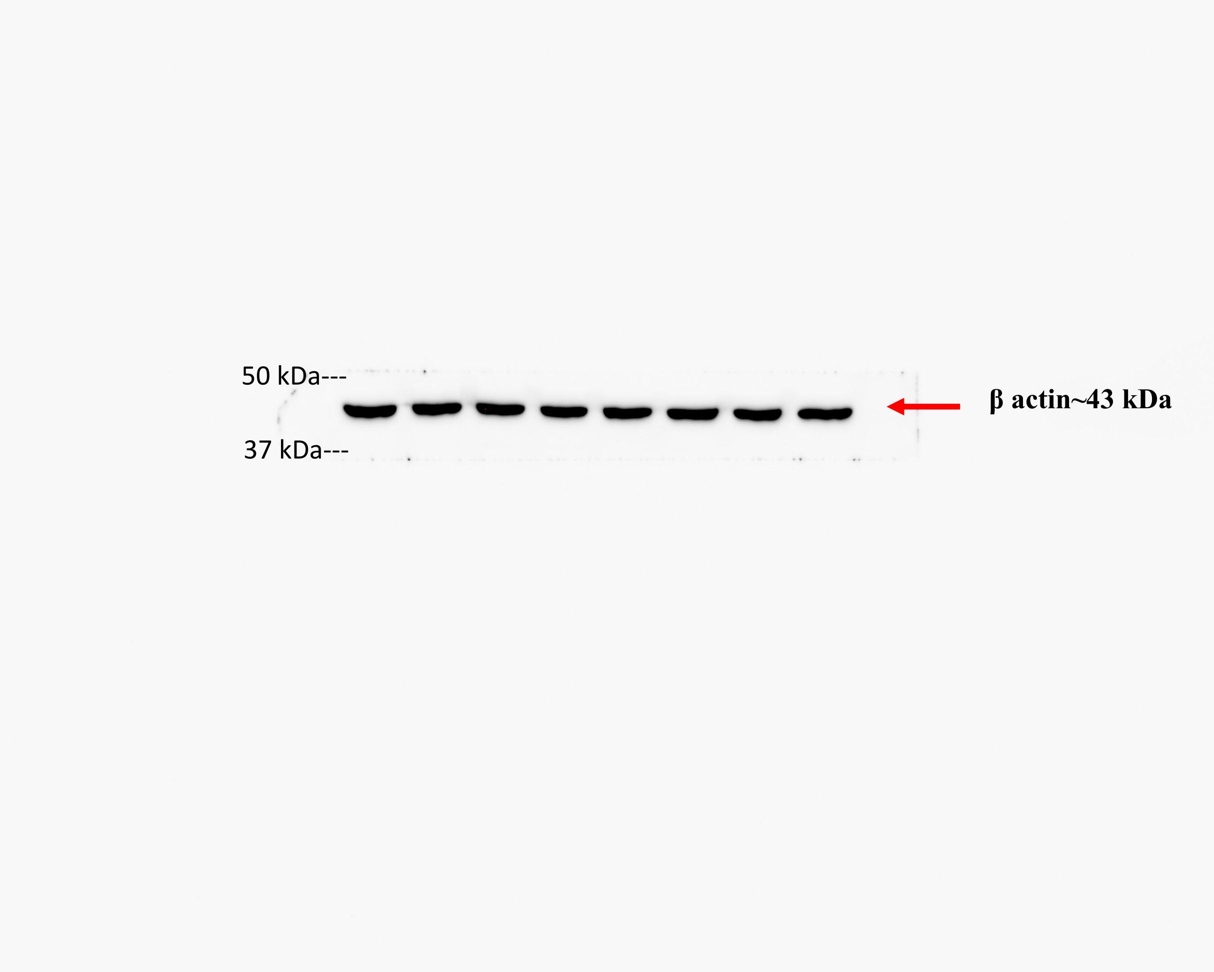

Figure S23. Uncropped PVDF membrane showing uPAR in KE5 cells. The raw image corresponds to the cropped data presented in Figure 3A.

Note: The membrane was sectioned according to the expected molecular weights of the target proteins before antibody incubation to enable the detection of multiple proteins from the same membrane. Representative blot images from the corresponding membrane sections are shown.

uPAR of KE5 cells (lane 1: NoLPS, lane 2: LPS-PG, lane 3: LPS-PG + EIF25, lane 4: LPS-PG + EIF50, lane 5: LPS-PG + EIF75, lane 6: LPS-PG + CGA5, lane 7: LPS-PG + CGA10, lane 8: LPS-PG + CGA20)

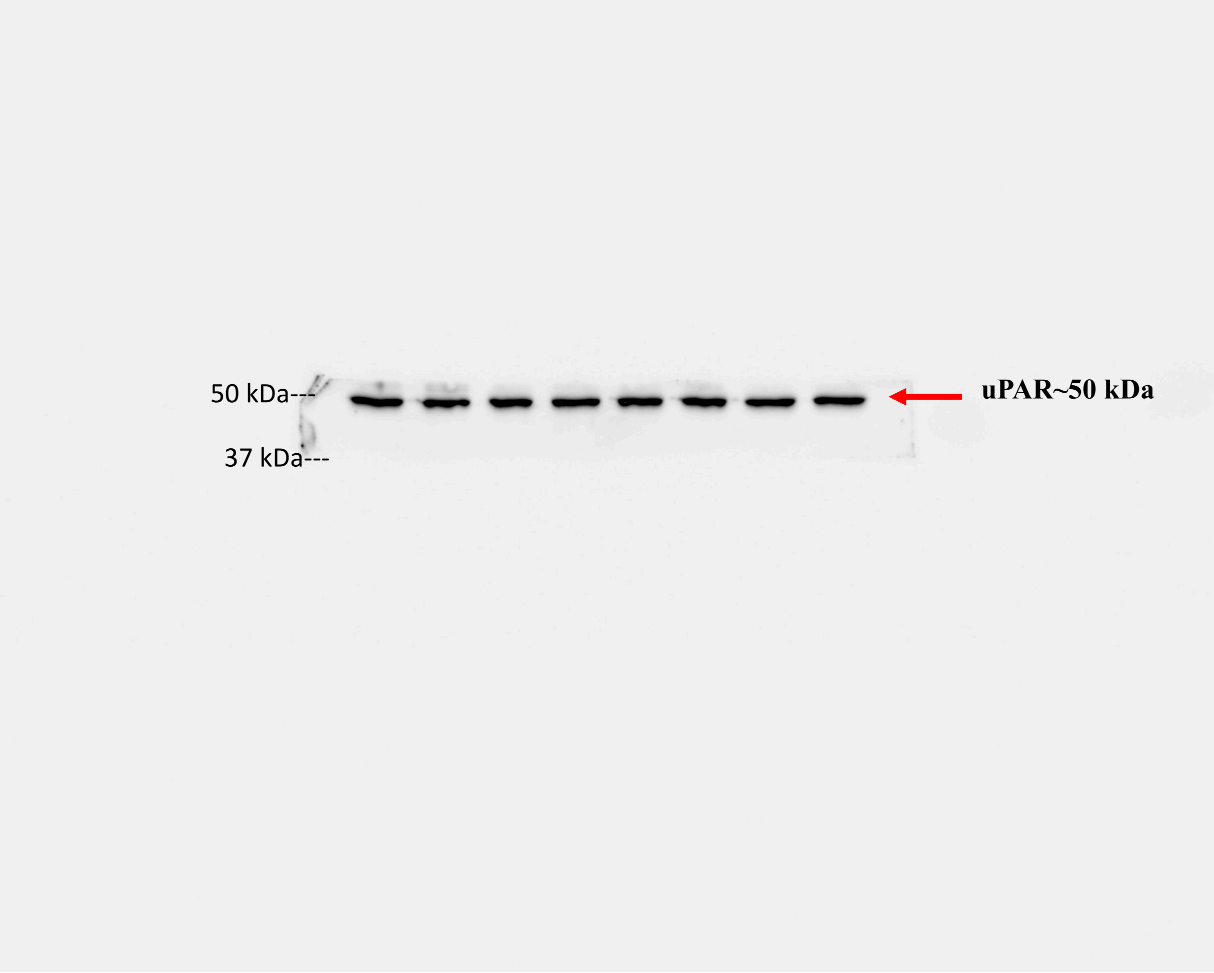

Figure S24. Uncropped PVDF membrane showing β-actin in KE5 cells. The raw image corresponds to the cropped data presented in Figure 3A.

Note: The membrane was sectioned according to the expected molecular weights of the target proteins before antibody incubation to enable the detection of multiple proteins from the same membrane. Representative blot images from the corresponding membrane sections are shown.

β-actin of KE5 cells (lane 1: NoLPS, lane 2: LPS-PG, lane 3: LPS-PG + EIF25, lane 4: LPS-PG + EIF50, lane 5: LPS-PG + EIF75, lane 6: LPS-PG + CGA5, lane 7: LPS-PG + CGA10, lane 8: LPS-PG + CGA20)

The identical β-actin band was utilized as a shared loading control for TNF-α and uPAR of KE5 cells , which were analyzed on the same membrane using cutting and stripping/reprobing methods to ensure accurate normalization.

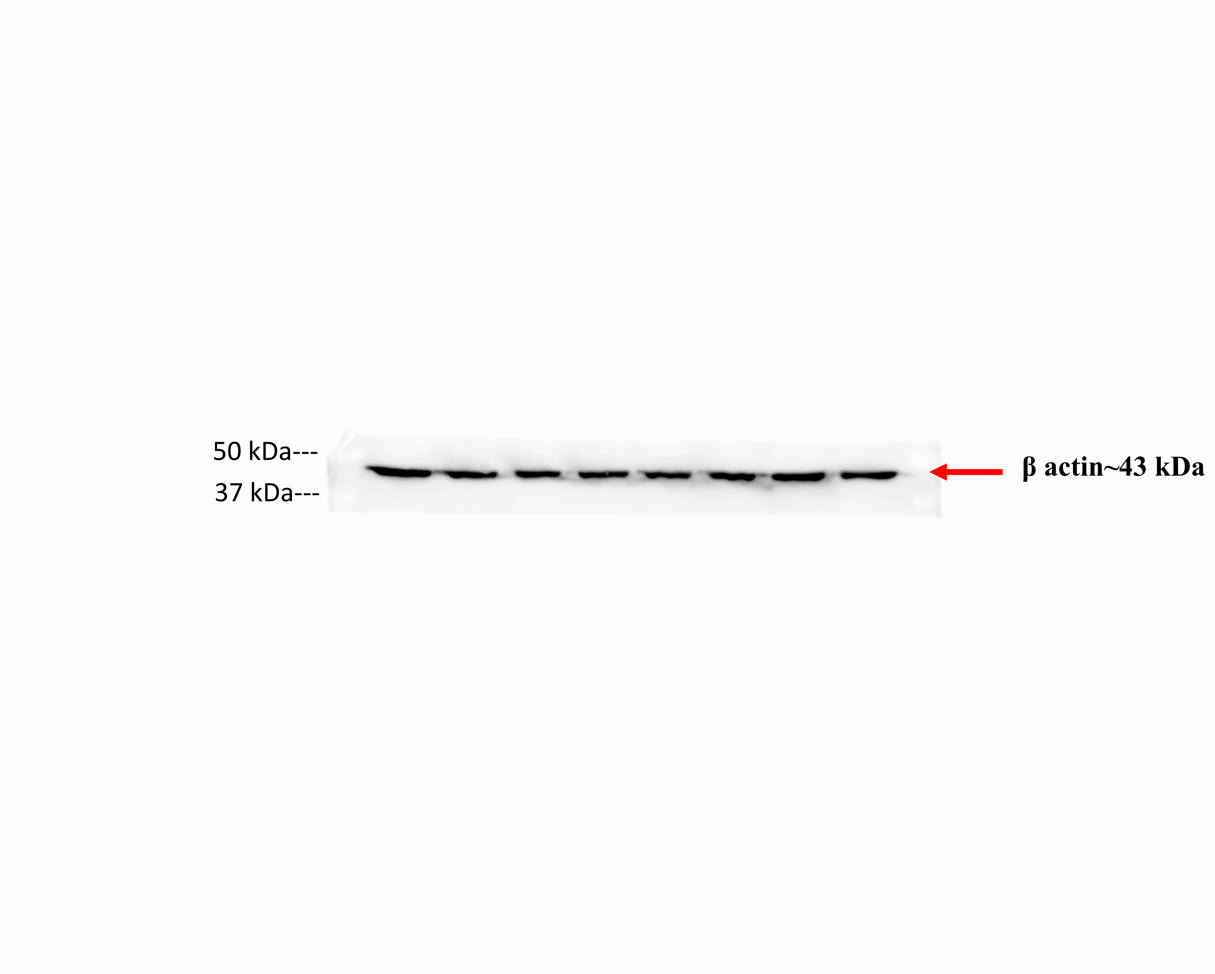

Figure S25. Uncropped PVDF membrane showing uPA in KE5 cells. The raw image corresponds to the cropped data presented in Figure 3A.

Note: The membrane was sectioned according to the expected molecular weights of the target proteins before antibody incubation to enable the detection of multiple proteins from the same membrane. Representative blot images from the corresponding membrane sections are shown.

uPA of KE5 cells (lane 1: NoLPS, lane 2: LPS-PG, lane 3: LPS-PG + EIF25, lane 4: LPS-PG + EIF50, lane 5: LPS-PG + EIF75, lane 6: LPS-PG + CGA5, lane 7: LPS-PG + CGA10, lane 8: LPS-PG + CGA20)

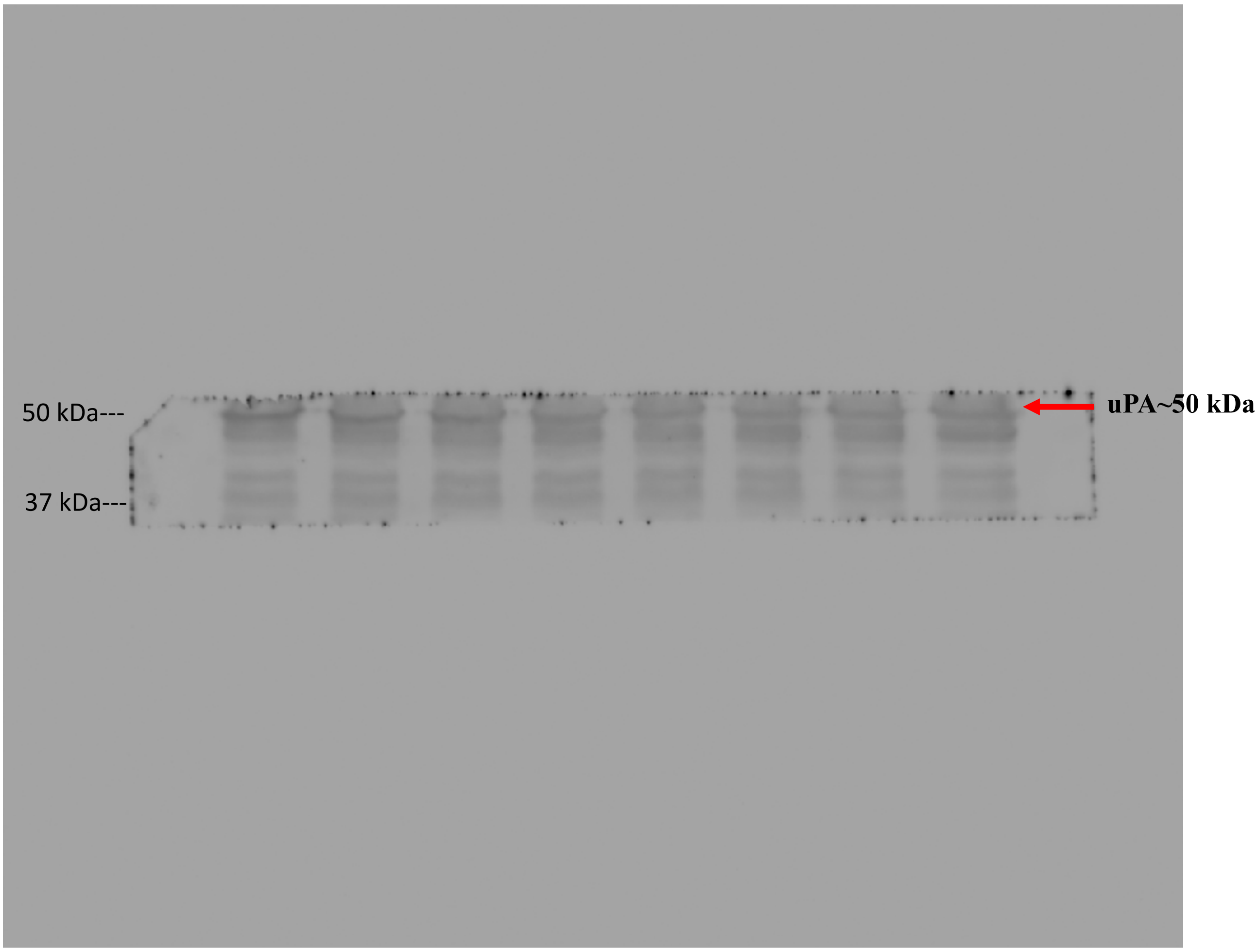

Figure S26. Uncropped PVDF membrane showing β-actin in KE5 cells. The raw image corresponds to the cropped data presented in Figure 3A.

Note: The membrane was sectioned according to the expected molecular weights of the target proteins before antibody incubation to enable the detection of multiple proteins from the same membrane. Representative blot images from the corresponding membrane sections are shown.

β-actin of KE5 cells (lane 1: NoLPS, lane 2: LPS-PG, lane 3: LPS-PG + EIF25, lane 4: LPS-PG + EIF50, lane 5: LPS-PG + EIF75, lane 6: LPS-PG + CGA5, lane 7: LPS-PG + CGA10, lane 8: LPS-PG + CGA20)

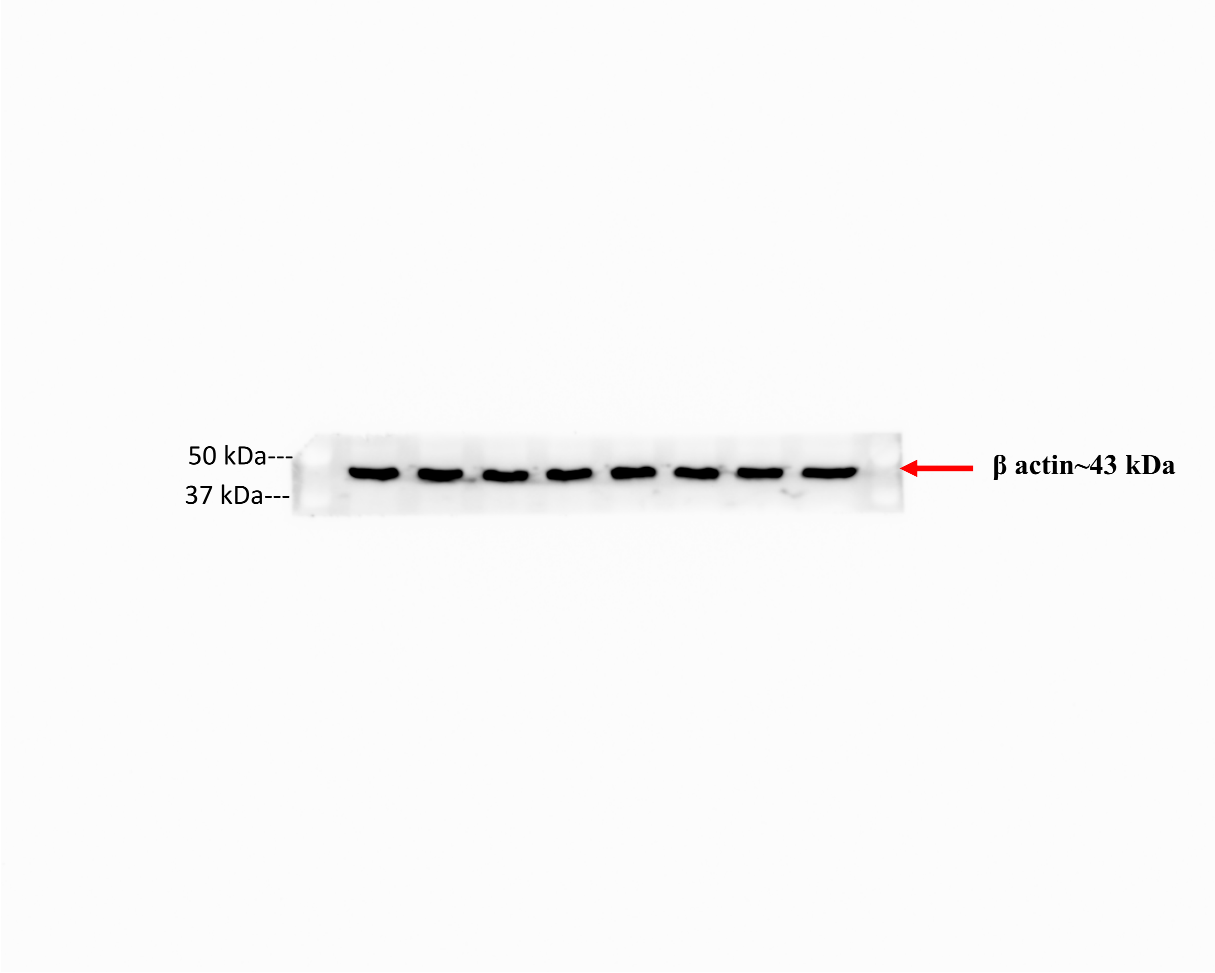

Figure S27. Uncropped PVDF membrane showing VEGFA in KE5 cells. The raw image corresponds to the cropped data presented in Figure 3A.

Note: The membrane was sectioned according to the expected molecular weights of the target proteins before antibody incubation to enable the detection of multiple proteins from the same membrane. Representative blot images from the corresponding membrane sections are shown.

VEGFA of KE5 cells (lane 1: NoLPS, lane 2: LPS-PG, lane 3: LPS-PG + EIF25, lane 4: LPS-PG + EIF50, lane 5: LPS-PG + EIF75, lane 6: LPS-PG + CGA5, lane 7: LPS-PG + CGA10, lane 8: LPS-PG + CGA20)

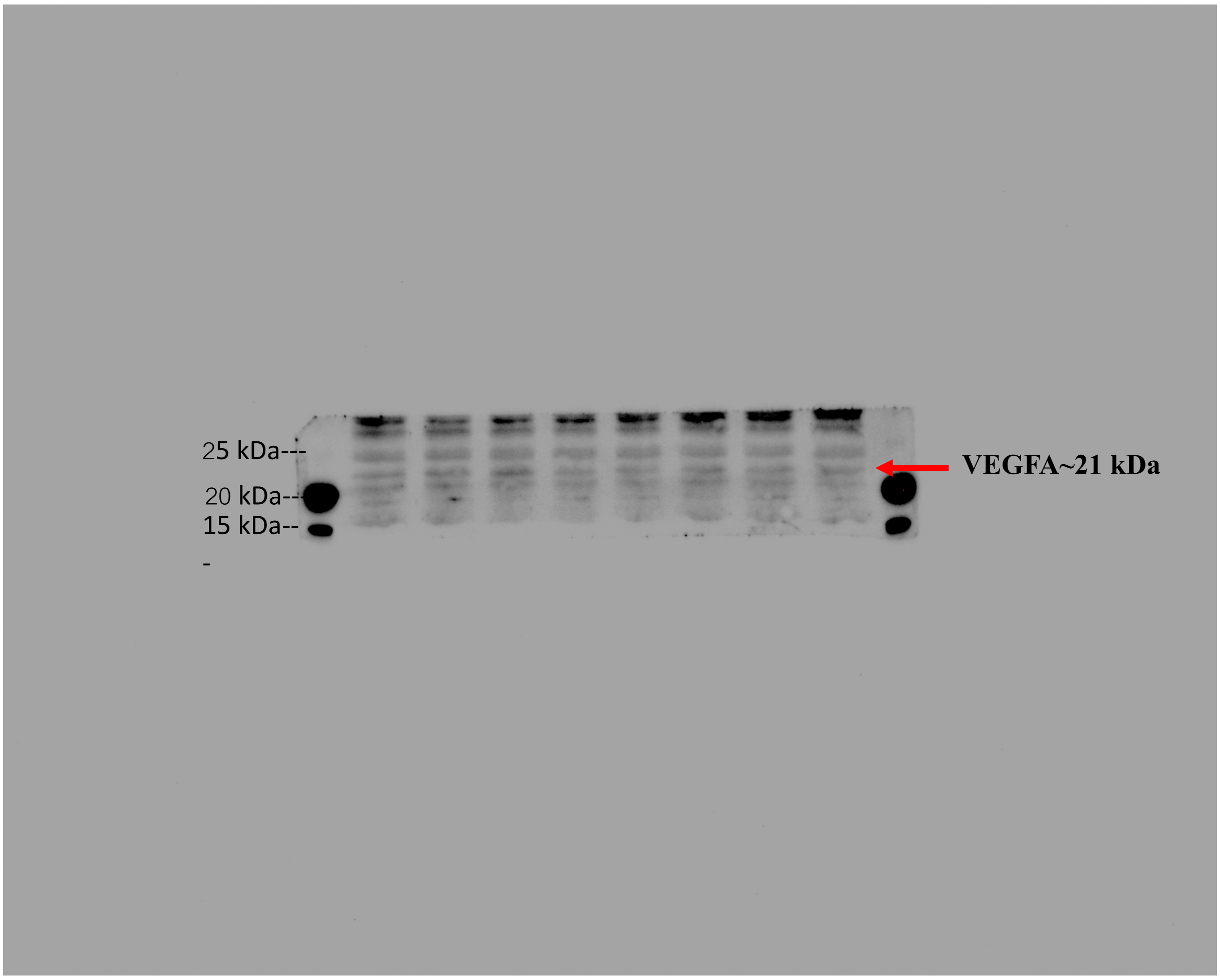

Figure S28. Uncropped PVDF membrane showing β-actin in KE5 cells. The raw image corresponds to the cropped data presented in Figure 3A.

Note: The membrane was sectioned according to the expected molecular weights of the target proteins before antibody incubation to enable the detection of multiple proteins from the same membrane. Representative blot images from the corresponding membrane sections are shown.

β-actin of KE5 cells (lane 1: NoLPS, lane 2: LPS-PG, lane 3: LPS-PG + EIF25, lane 4: LPS-PG + EIF50, lane 5: LPS-PG + EIF75, lane 6: LPS-PG + CGA5, lane 7: LPS-PG + CGA10, lane 8: LPS-PG + CGA20)

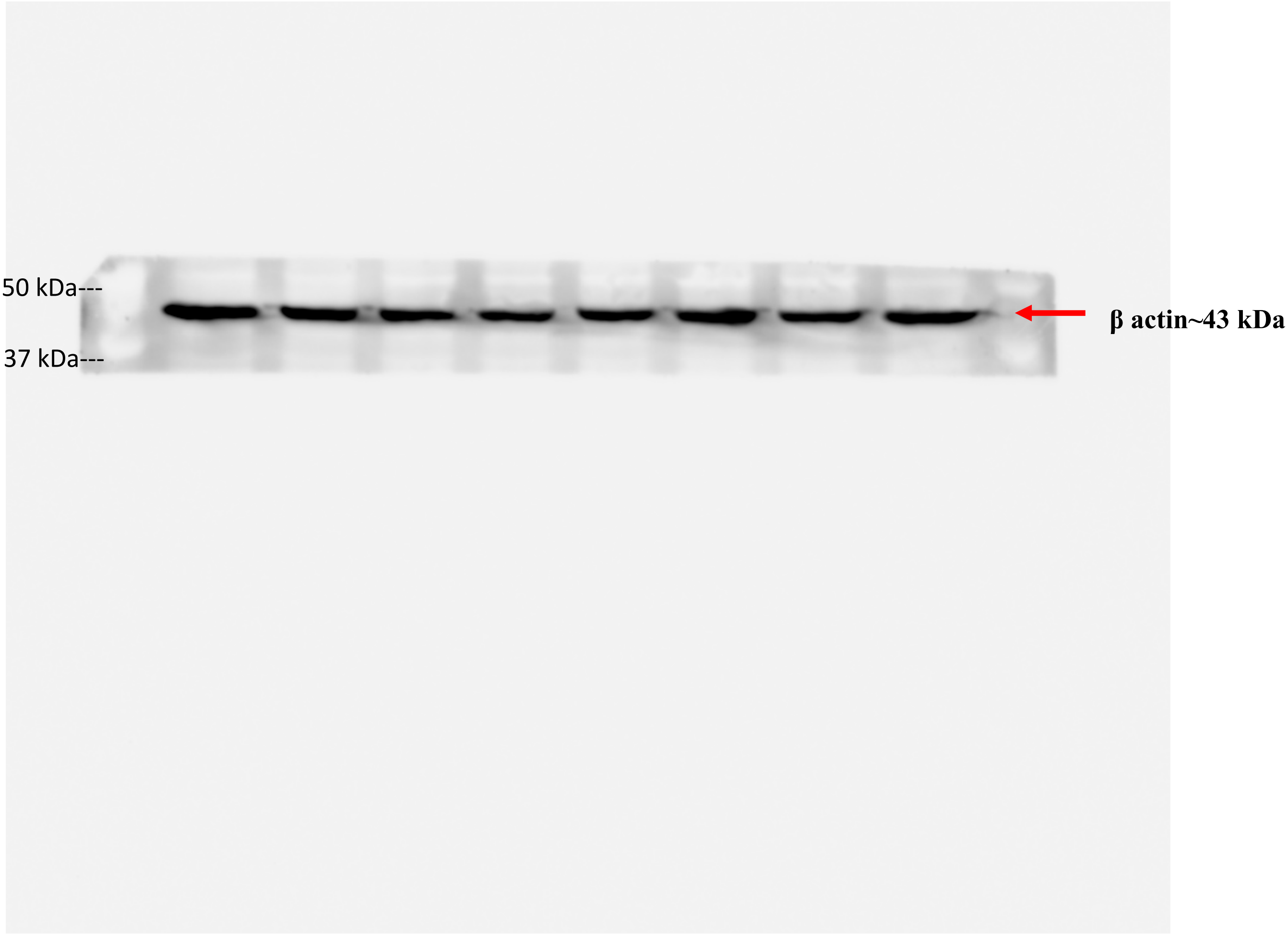

Figure S29. Uncropped PVDF membrane showing VEGFC in KE5 cells. The raw image corresponds to the cropped data presented in Figure 3A.

Note: The membrane was sectioned according to the expected molecular weights of the target proteins before antibody incubation to enable the detection of multiple proteins from the same membrane. Representative blot images from the corresponding membrane sections are shown.

VEGFC of KE5 cells (lane 1: NoLPS, lane 2: LPS-PG, lane 3: LPS-PG + EIF25, lane 4: LPS-PG + EIF50, lane 5: LPS-PG + EIF75, lane 6: LPS-PG + CGA5, lane 7: LPS-PG + CGA10, lane 8: LPS-PG + CGA20)

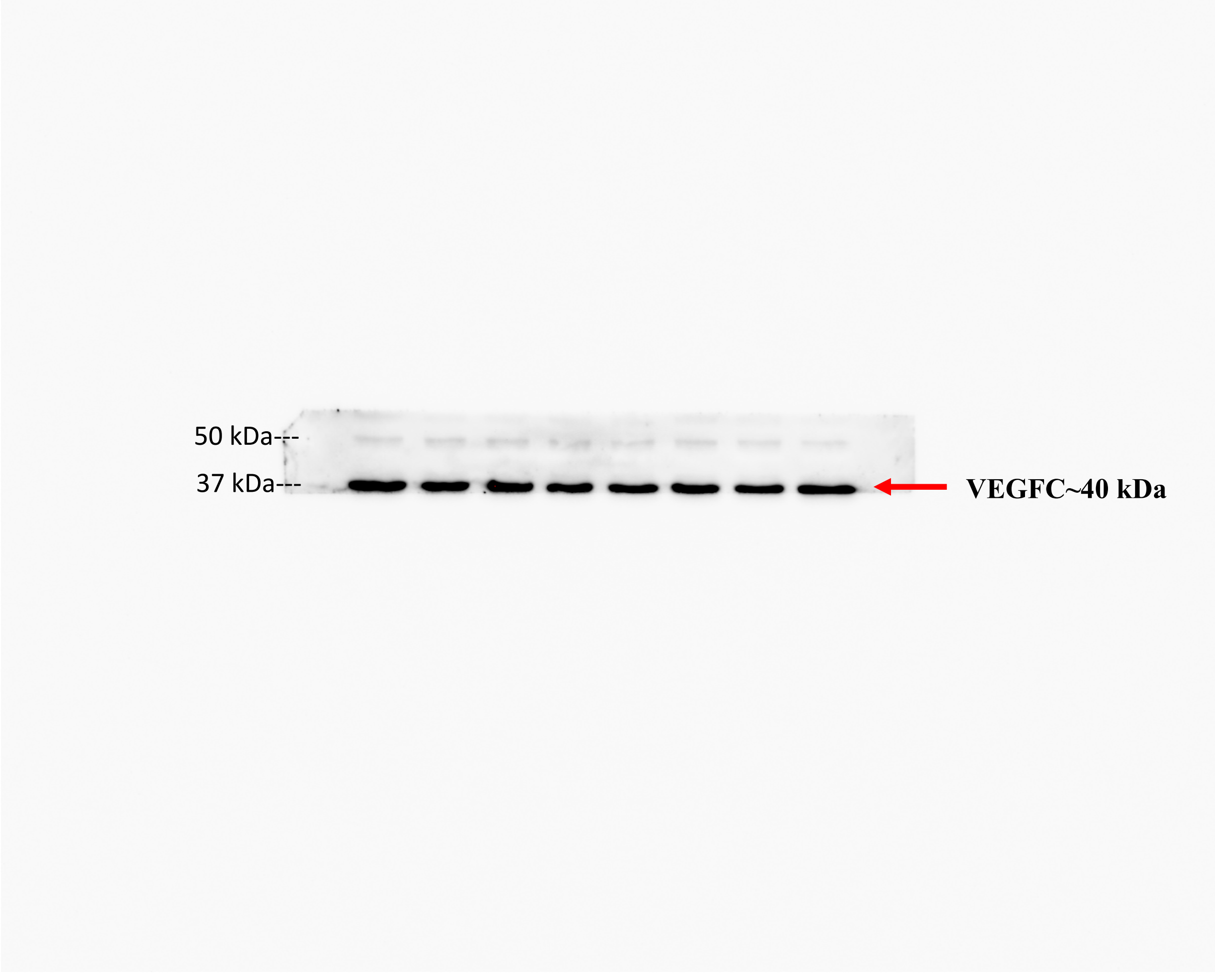

Figure S30. Uncropped PVDF membrane showing β-actin in KE5 cells. The raw image corresponds to the cropped data presented in Figure 3A.

Note: The membrane was sectioned according to the expected molecular weights of the target proteins before antibody incubation to enable the detection of multiple proteins from the same membrane. Representative blot images from the corresponding membrane sections are shown.

β-actin of KE5 cells (lane 1: NoLPS, lane 2: LPS-PG, lane 3: LPS-PG + EIF25, lane 4: LPS-PG + EIF50, lane 5: LPS-PG + EIF75, lane 6: LPS-PG + CGA5, lane 7: LPS-PG + CGA10, lane 8: LPS-PG + CGA20)

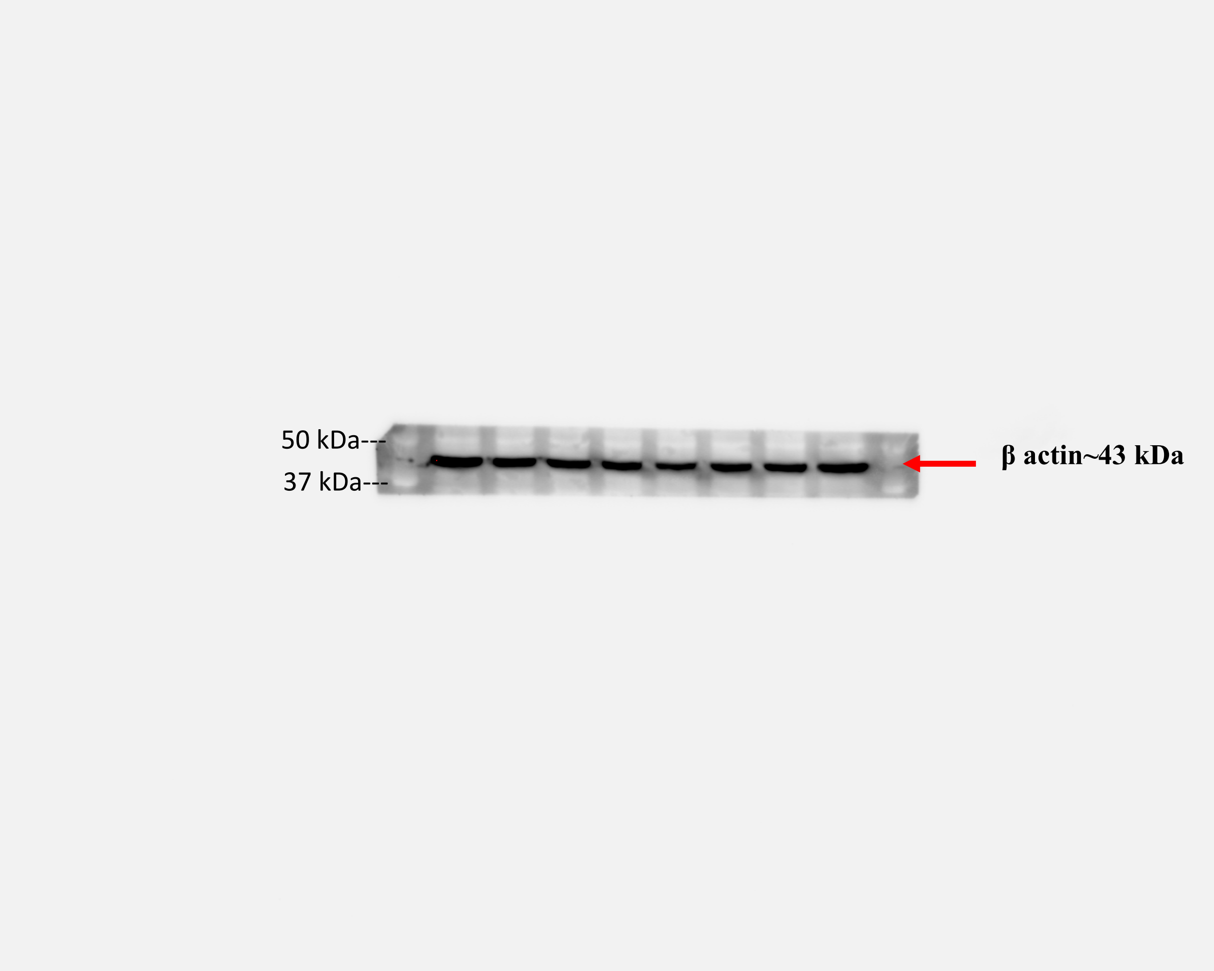

Figure S31. Uncropped PVDF membrane showing uPAR in T.T cells. The raw image corresponds to the cropped data presented in Figure 3B.

Note: The membrane was sectioned according to the expected molecular weights of the target proteins before antibody incubation to enable the detection of multiple proteins from the same membrane. Representative blot images from the corresponding membrane sections are shown.

uPAR of T.T cells (lane 1: NoLPS, lane 2: LPS-PG, lane 3: LPS-PG + EIF25, lane 4: LPS-PG + EIF50, lane 5: LPS-PG + EIF75, lane 6: LPS-PG + CGA5, lane 7: LPS-PG + CGA10, lane 8: LPS-PG + CGA20)

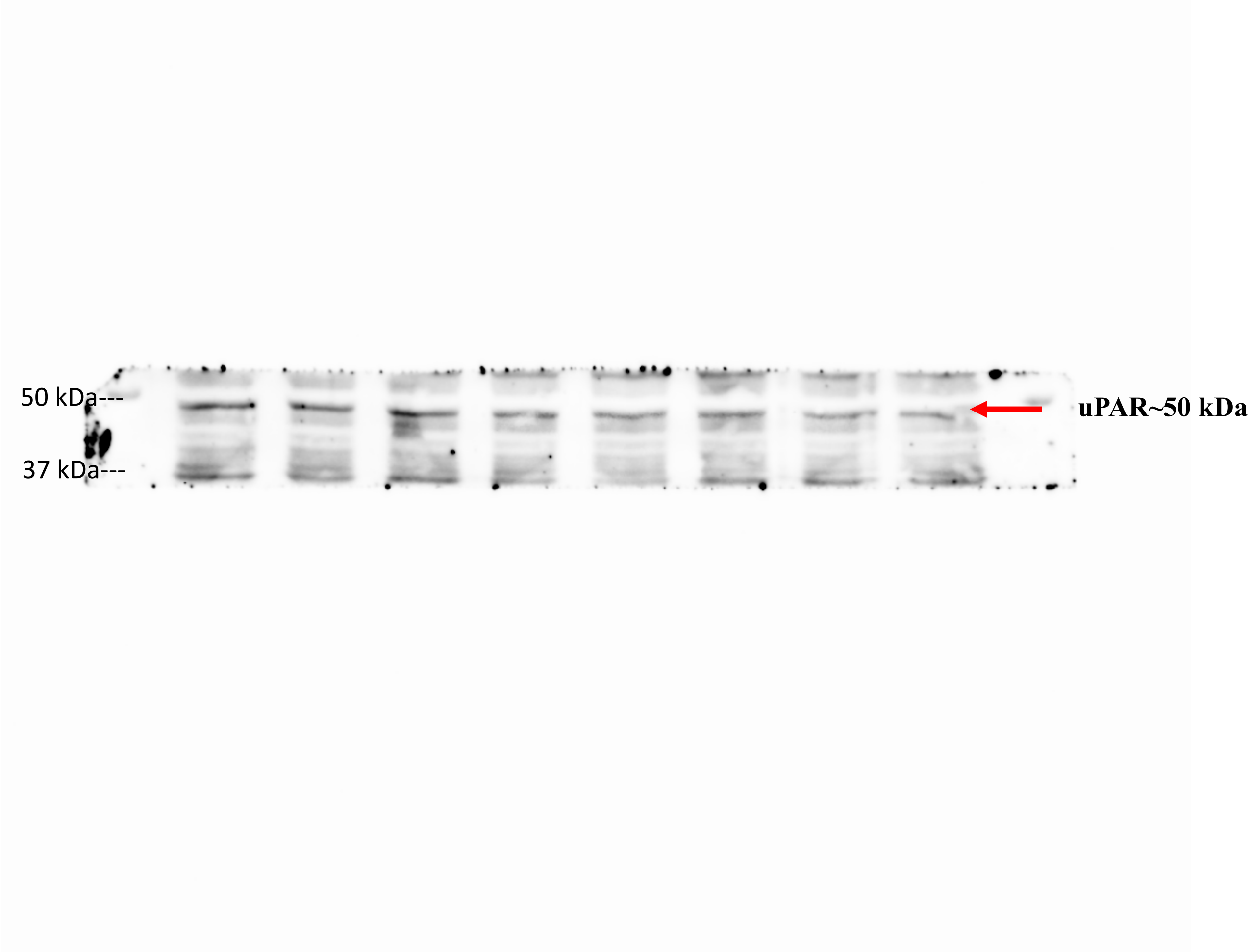

Figure S32. Uncropped PVDF membrane showing β-actin in T.T cells. The raw image corresponds to the cropped data presented in Figure 3B.

Note: The membrane was sectioned according to the expected molecular weights of the target proteins before antibody incubation to enable the detection of multiple proteins from the same membrane. Representative blot images from the corresponding membrane sections are shown.

β-actin of T.T cells (lane 1: NoLPS, lane 2: LPS-PG, lane 3: LPS-PG + EIF25, lane 4: LPS-PG + EIF50, lane 5: LPS-PG + EIF75, lane 6: LPS-PG + CGA5, lane 7: LPS-PG + CGA10, lane 8: LPS-PG + CGA20)

The identical β-actin band was utilized as a shared loading control for uPAR, pp38, and p38 of T.T cells, which were analyzed on the same membrane using cutting and stripping/reprobing methods to ensure accurate normalization.

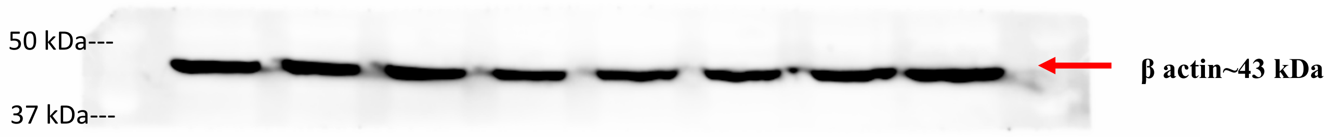

Figure S33. Uncropped PVDF membrane showing uPA in T.T cells. The raw image corresponds to the cropped data presented in Figure 3B.

Note: The membrane was sectioned according to the expected molecular weights of the target proteins before antibody incubation to enable the detection of multiple proteins from the same membrane. Representative blot images from the corresponding membrane sections are shown.

uPA of T.T cells (lane 1: NoLPS, lane 2: LPS-PG, lane 3: LPS-PG + EIF25, lane 4: LPS-PG + EIF50, lane 5: LPS-PG + EIF75, lane 6: LPS-PG + CGA5, lane 7: LPS-PG + CGA10, lane 8: LPS-PG + CGA20)

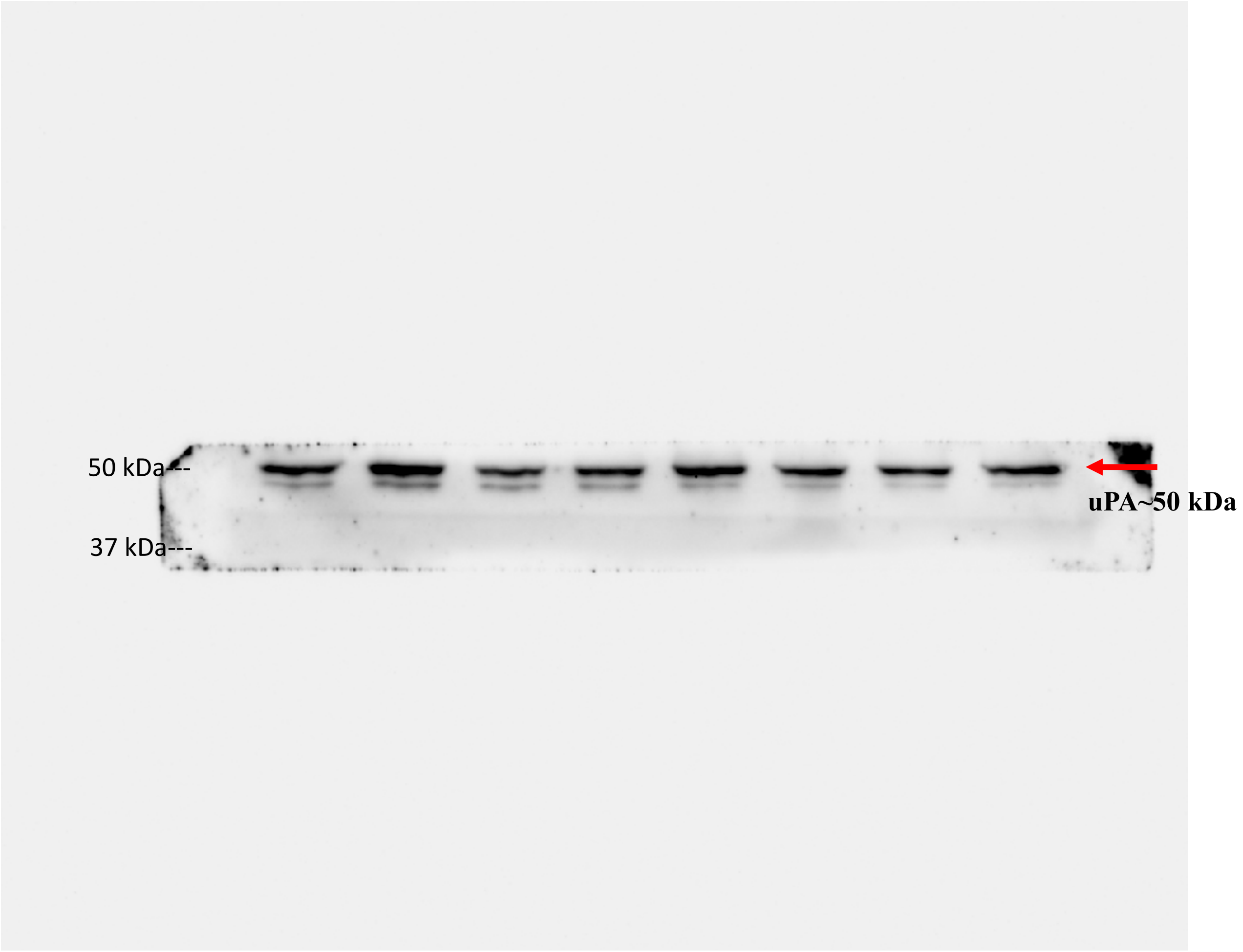

Figure S34. Uncropped PVDF membrane showing β-actin in T.T cells. The raw image corresponds to the cropped data presented in Figure 3B.

Note: The membrane was sectioned according to the expected molecular weights of the target proteins before antibody incubation to enable the detection of multiple proteins from the same membrane. Representative blot images from the corresponding membrane sections are shown.

β-actin of T.T cells (lane 1: NoLPS, lane 2: LPS-PG, lane 3: LPS-PG + EIF25, lane 4: LPS-PG + EIF50, lane 5: LPS-PG + EIF75, lane 6: LPS-PG + CGA5, lane 7: LPS-PG + CGA10, lane 8: LPS-PG + CGA20)

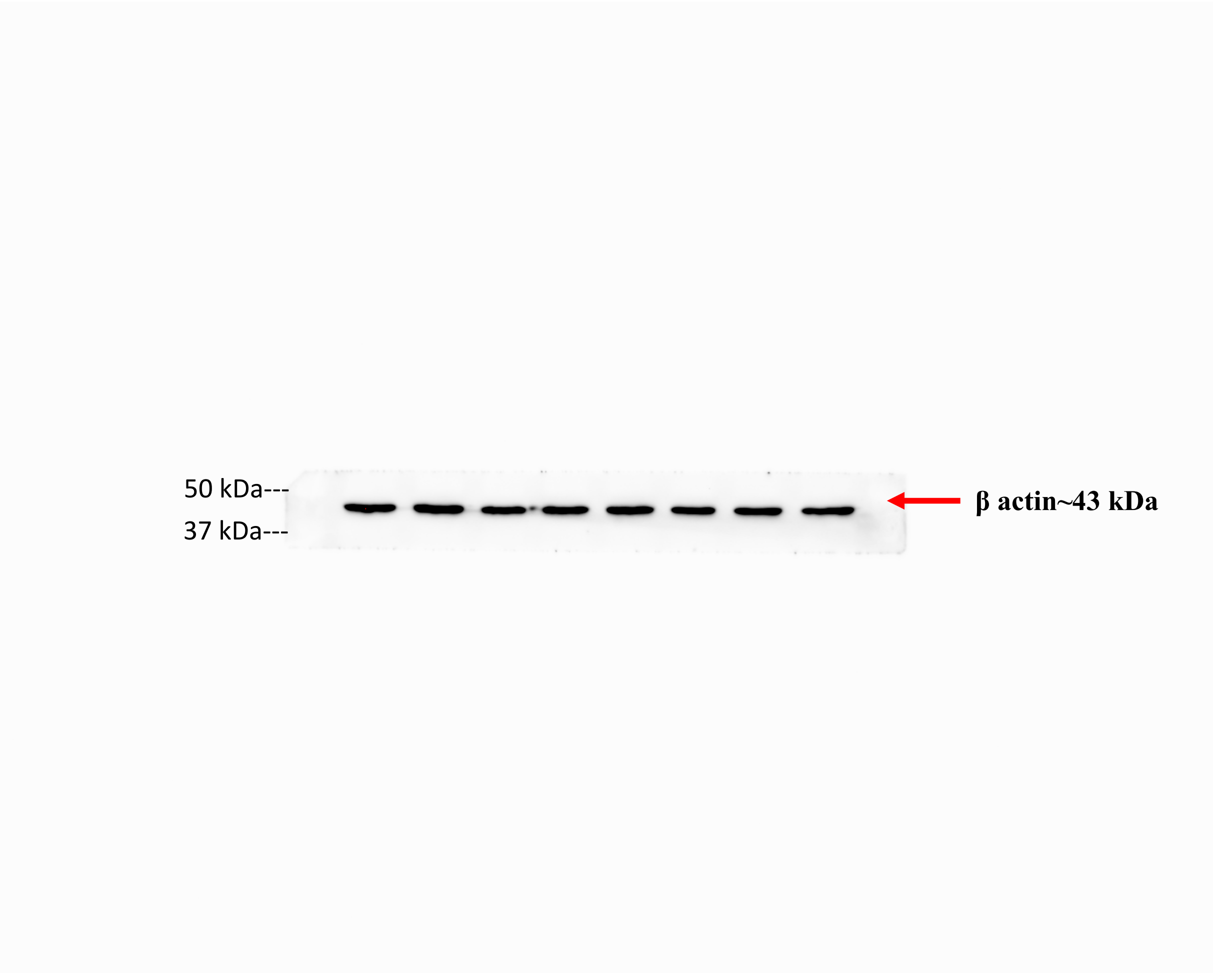

Figure S35. Uncropped PVDF membrane showing VEGFA in T.T cells. The raw image corresponds to the cropped data presented in Figure 3B.

Note: The membrane was sectioned according to the expected molecular weights of the target proteins before antibody incubation to enable the detection of multiple proteins from the same membrane. Representative blot images from the corresponding membrane sections are shown.

VEGFA of T.T cells (lane 1: NoLPS, lane 2: LPS-PG, lane 3: LPS-PG + EIF25, lane 4: LPS-PG + EIF50, lane 5: LPS-PG + EIF75, lane 6: LPS-PG + CGA5, lane 7: LPS-PG + CGA10, lane 8: LPS-PG + CGA20)

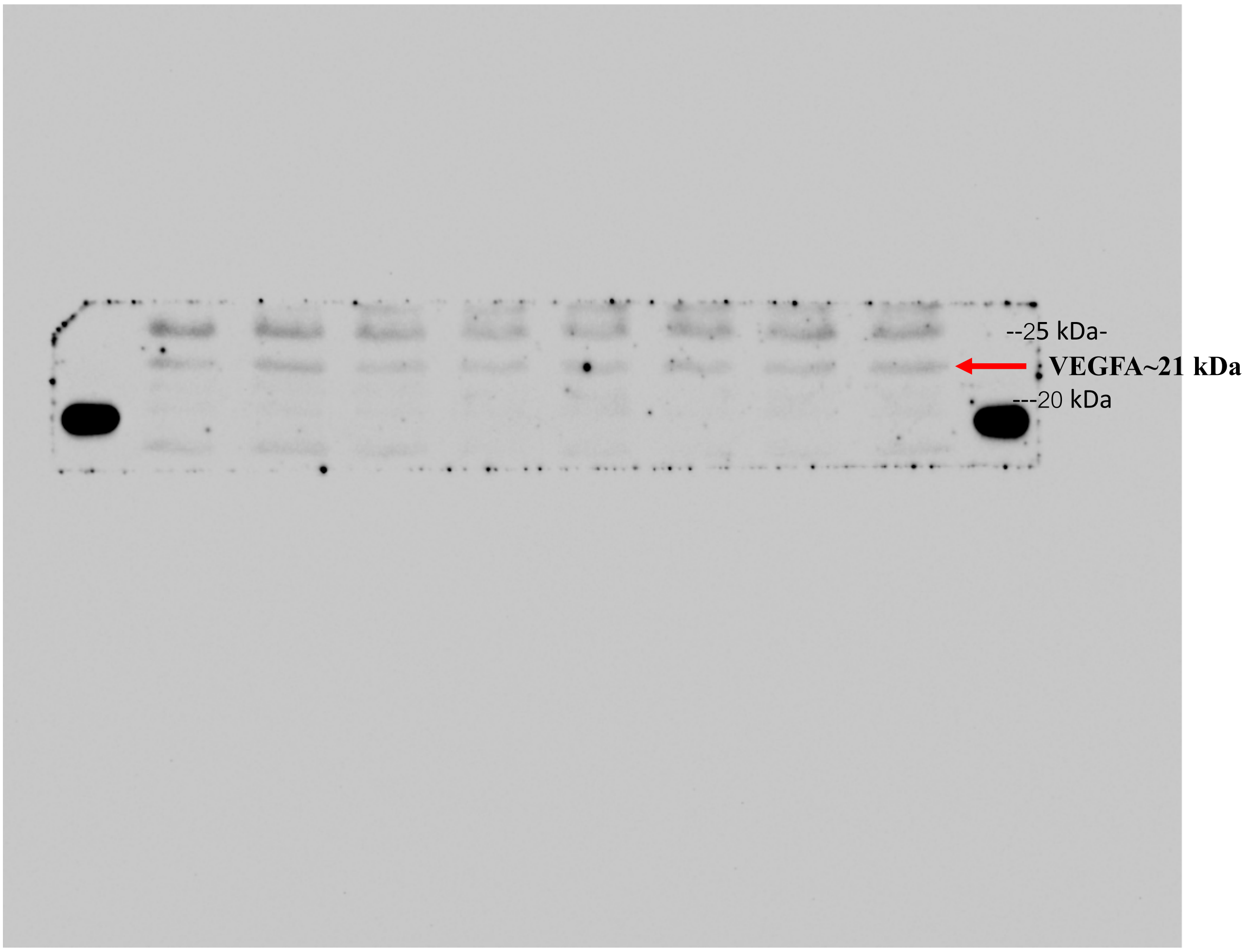

Figure S36. Uncropped PVDF membrane showing β-actin in T.T cells. The raw image corresponds to the cropped data presented in Figure 3B.

Note: The membrane was sectioned according to the expected molecular weights of the target proteins before antibody incubation to enable the detection of multiple proteins from the same membrane. Representative blot images from the corresponding membrane sections are shown.

β-actin of T.T cells (lane 1: NoLPS, lane 2: LPS-PG, lane 3: LPS-PG + EIF25, lane 4: LPS-PG + EIF50, lane 5: LPS-PG + EIF75, lane 6: LPS-PG + CGA5, lane 7: LPS-PG + CGA10, lane 8: LPS-PG + CGA20)

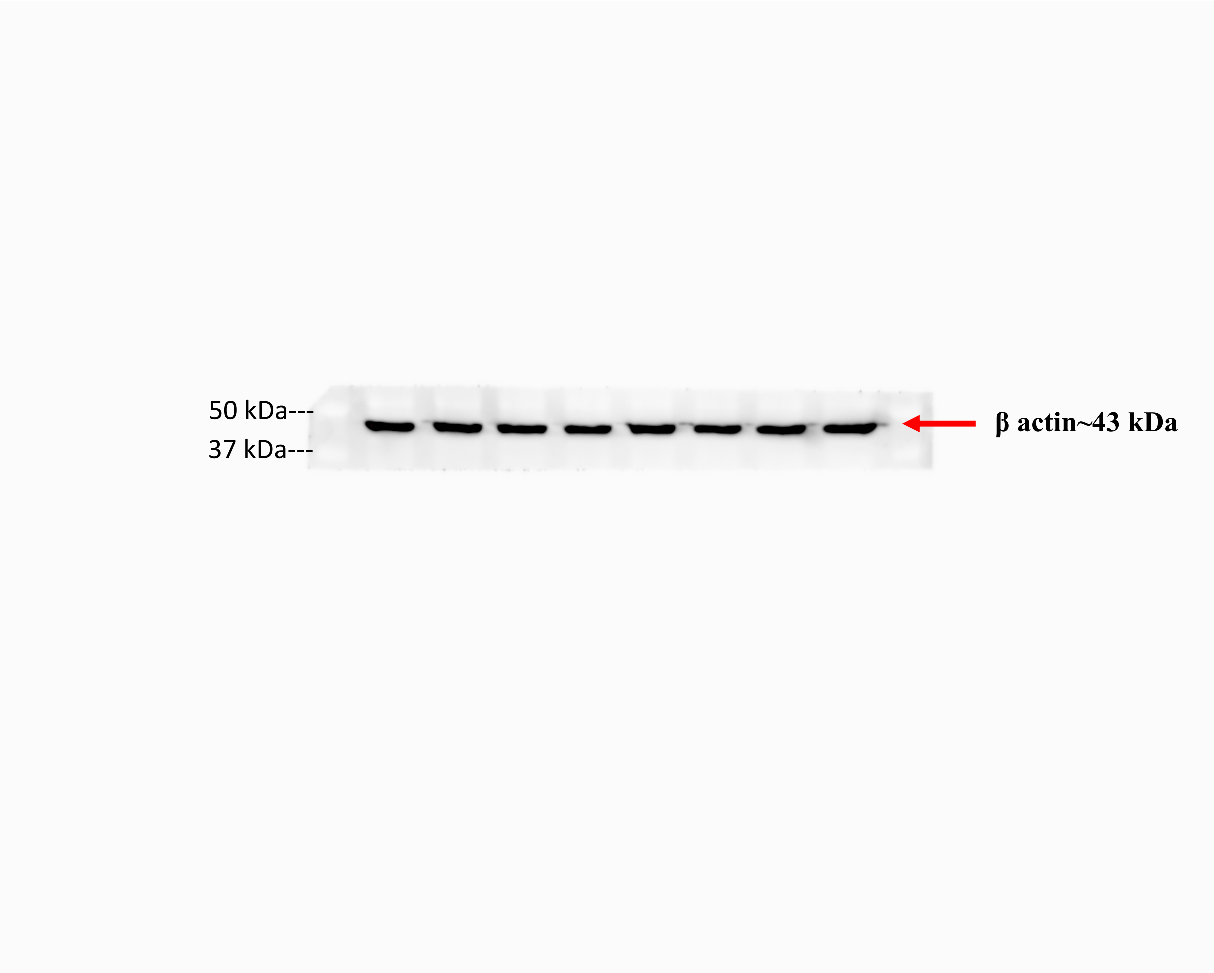

Figure S37. Uncropped PVDF membrane showing VEGFC in T.T cells**.** The raw image corresponds to the cropped data presented in Figure 3B.

Note: The membrane was sectioned according to the expected molecular weights of the target proteins before antibody incubation to enable the detection of multiple proteins from the same membrane. Representative blot images from the corresponding membrane sections are shown.

VEGFC of T.T cells (lane 1: NoLPS, lane 2: LPS-PG, lane 3: LPS-PG + EIF25, lane 4: LPS-PG + EIF50, lane 5: LPS-PG + EIF75, lane 6: LPS-PG + CGA5, lane 7: LPS-PG + CGA10, lane 8: LPS-PG + CGA20)

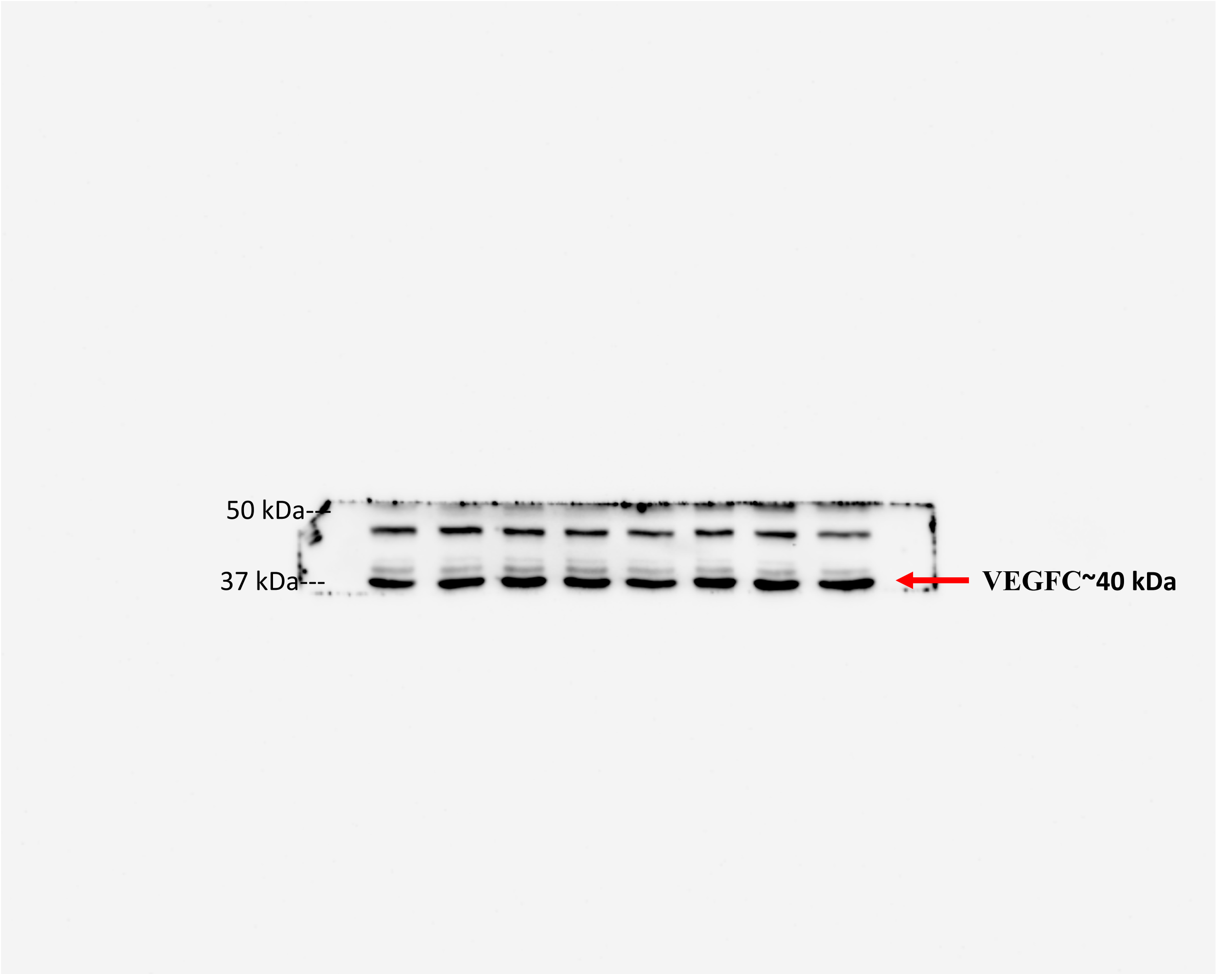

Figure S38. Uncropped PVDF membrane showing β-actin in T.T cells. The raw image corresponds to the cropped data presented in Figure 3B.

Note: The membrane was sectioned according to the expected molecular weights of the target proteins before antibody incubation to enable the detection of multiple proteins from the same membrane. Representative blot images from the corresponding membrane sections are shown.

β-actin of T.T cells (lane 1: NoLPS, lane 2: LPS-PG, lane 3: LPS-PG + EIF25, lane 4: LPS-PG + EIF50, lane 5: LPS-PG + EIF75, lane 6: LPS-PG + CGA5, lane 7: LPS-PG + CGA10, lane 8: LPS-PG + CGA20)

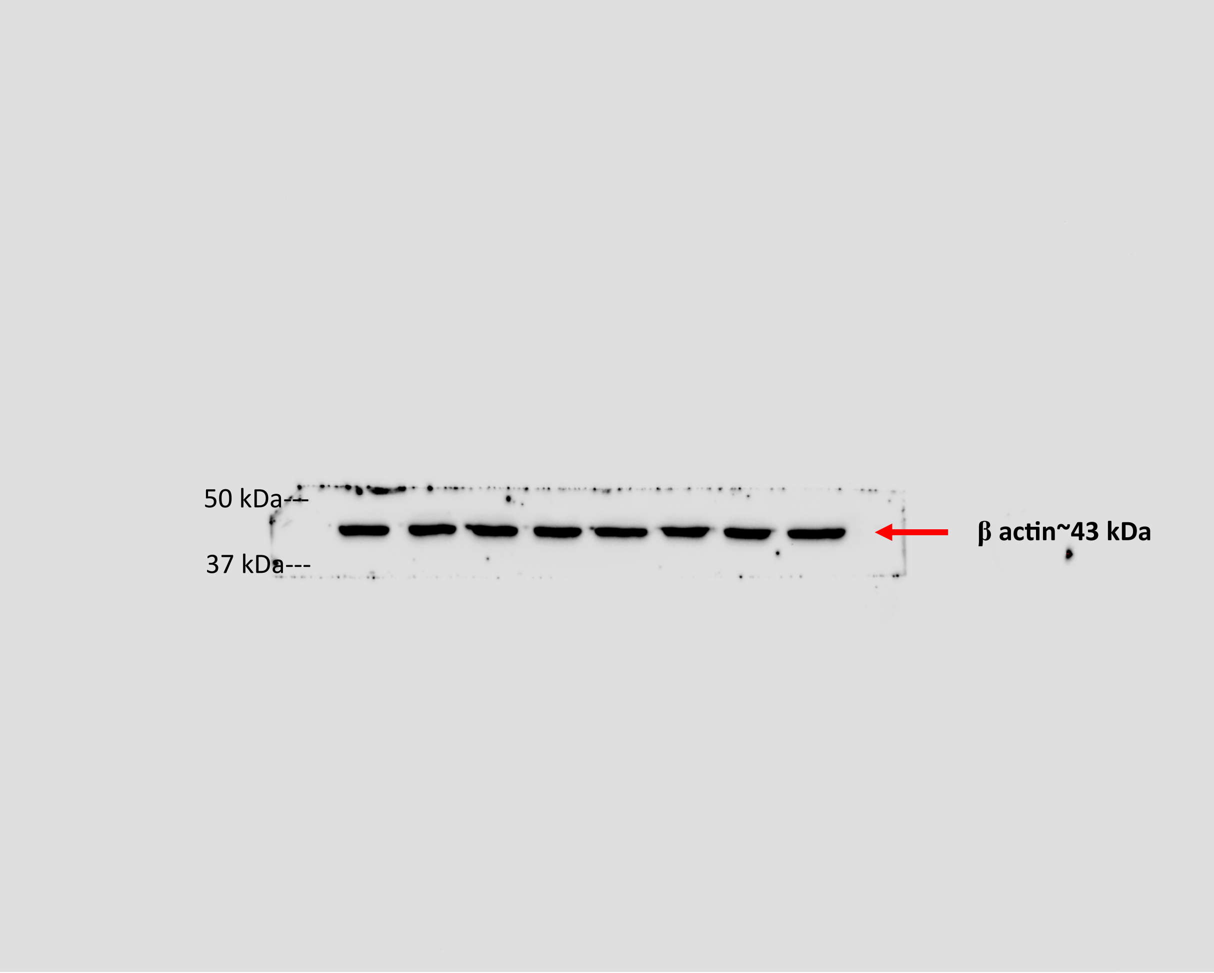

Figure S39. Uncropped PVDF membrane showing pp65 in KE5 cells. The raw image corresponds to the cropped data presented in Figure 6A.

Note: The membrane was sectioned according to the expected molecular weights of the target proteins before antibody incubation to enable the detection of multiple proteins from the same membrane. Representative blot images from the corresponding membrane sections are shown.

pp65 of KE5 cells (lane 1: NoLPS, lane 2: LPS-PG, lane 3: LPS-PG + EIF25, lane 4: LPS-PG + EIF50, lane 5: LPS-PG + EIF75, lane 6: LPS-PG + CGA5, lane 7: LPS-PG + CGA10, lane 8: LPS-PG + CGA20)

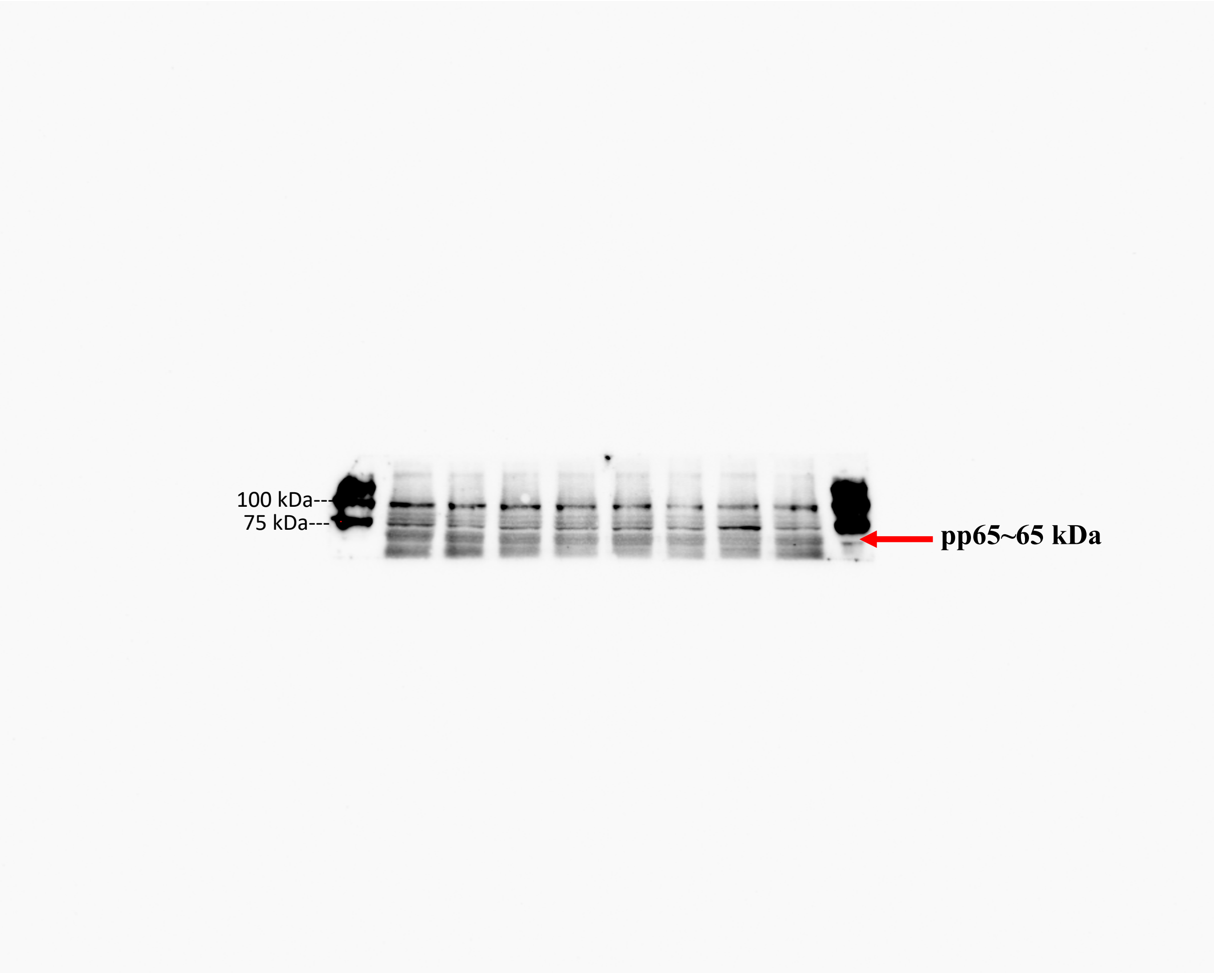

Figure S40. Uncropped PVDF membrane showing β-actin in KE5 cells. The raw image corresponds to the cropped data presented in Figure 6A.

Note: The membrane was sectioned according to the expected molecular weights of the target proteins before antibody incubation to enable the detection of multiple proteins from the same membrane. Representative blot images from the corresponding membrane sections are shown.

β-actin of KE5 cells (lane 1: NoLPS, lane 2: LPS-PG, lane 3: LPS-PG + EIF25, lane 4: LPS-PG + EIF50, lane 5: LPS-PG + EIF75, lane 6: LPS-PG + CGA5, lane 7: LPS-PG + CGA10, lane 8: LPS-PG + CGA20)

The identical β-actin band was utilized as a shared loading control for pp65 and p65 of KE5 cells, which were analyzed on the same membrane using cutting and stripping/reprobing methods to ensure accurate normalization.

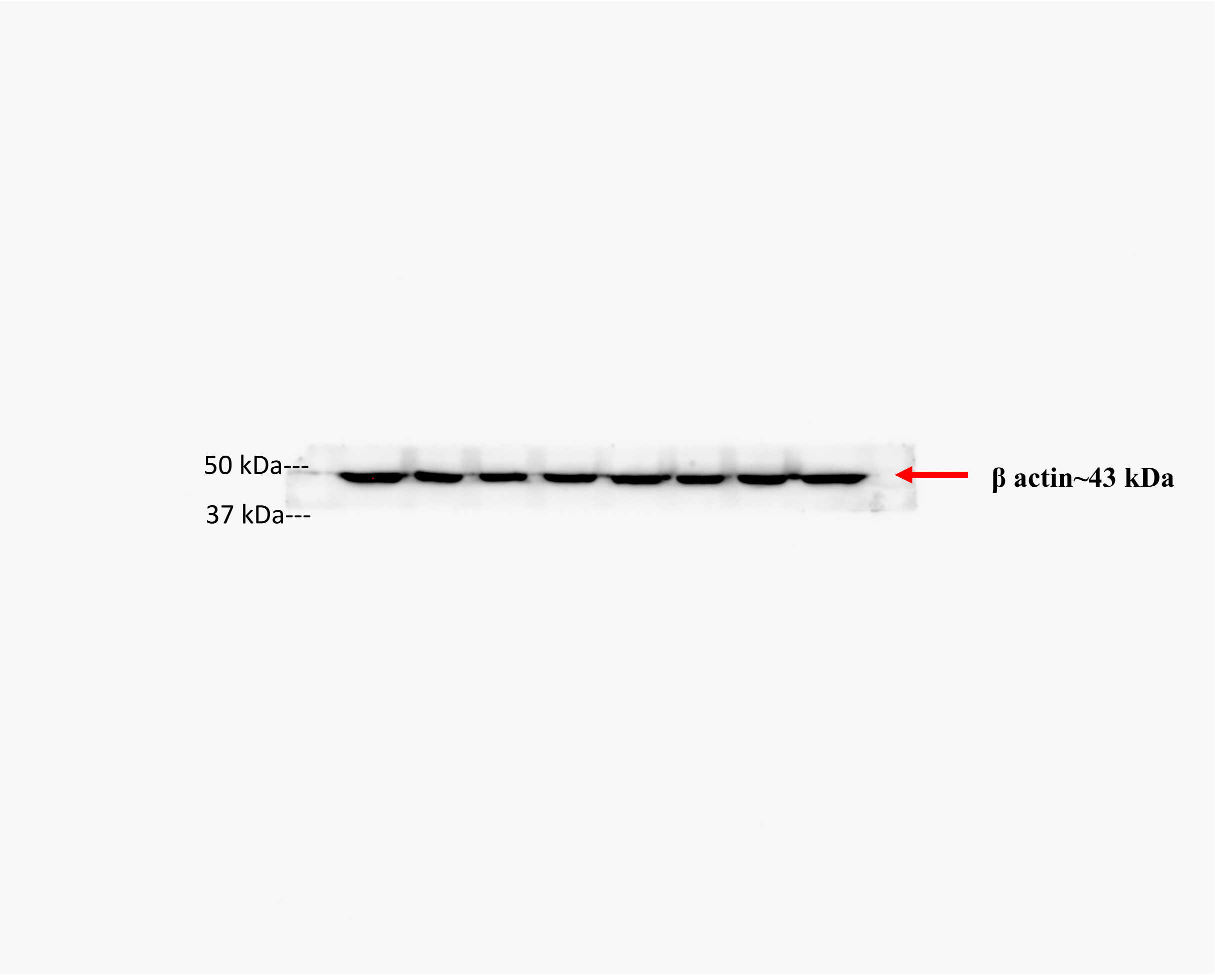

Figure S41. Uncropped PVDF membrane showing p65 in KE5 cells. The raw image corresponds to the cropped data presented in Figure 6A.

Note: The membrane was sectioned according to the expected molecular weights of the target proteins before antibody incubation to enable the detection of multiple proteins from the same membrane. Representative blot images from the corresponding membrane sections are shown.

p65 of KE5 cells (lane 1: NoLPS, lane 2: LPS-PG, lane 3: LPS-PG + EIF25, lane 4: LPS-PG + EIF50, lane 5: LPS-PG + EIF75, lane 6: LPS-PG + CGA5, lane 7: LPS-PG + CGA10, lane 8: LPS-PG + CGA20)

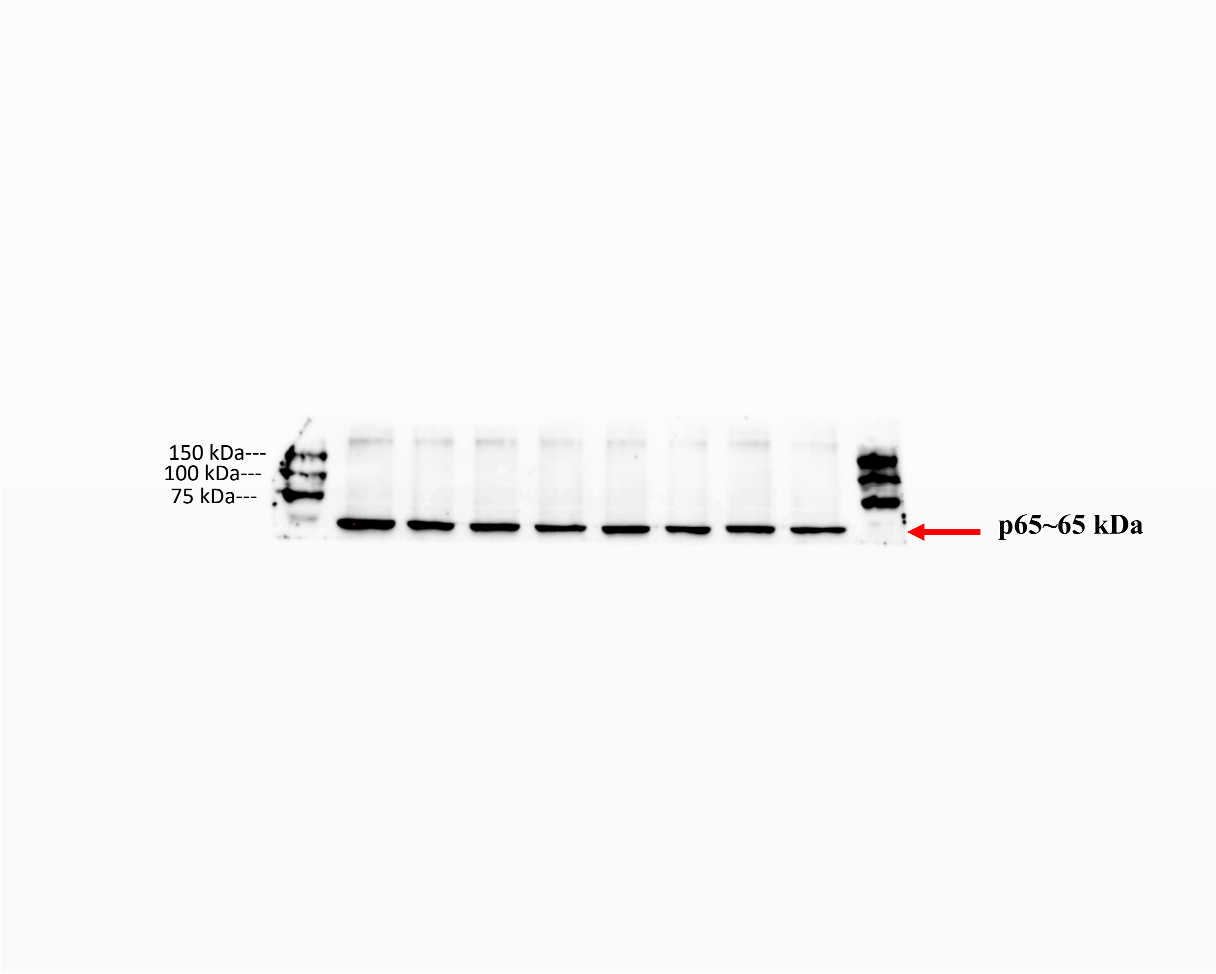

Figure S42. Uncropped PVDF membrane showing β-actin in KE5 cells. The raw image corresponds to the cropped data presented in Figure 6A.

Note: The membrane was sectioned according to the expected molecular weights of the target proteins before antibody incubation to enable the detection of multiple proteins from the same membrane. Representative blot images from the corresponding membrane sections are shown.

β-actin of KE5 cells (lane 1: NoLPS, lane 2: LPS-PG, lane 3: LPS-PG + EIF25, lane 4: LPS-PG + EIF50, lane 5: LPS-PG + EIF75, lane 6: LPS-PG + CGA5, lane 7: LPS-PG + CGA10, lane 8: LPS-PG + CGA20)

The identical β-actin band was utilized as a shared loading control for pp65 and p65 of KE5 cells, which were analyzed on the same membrane using cutting and stripping/reprobing methods to ensure accurate normalization.

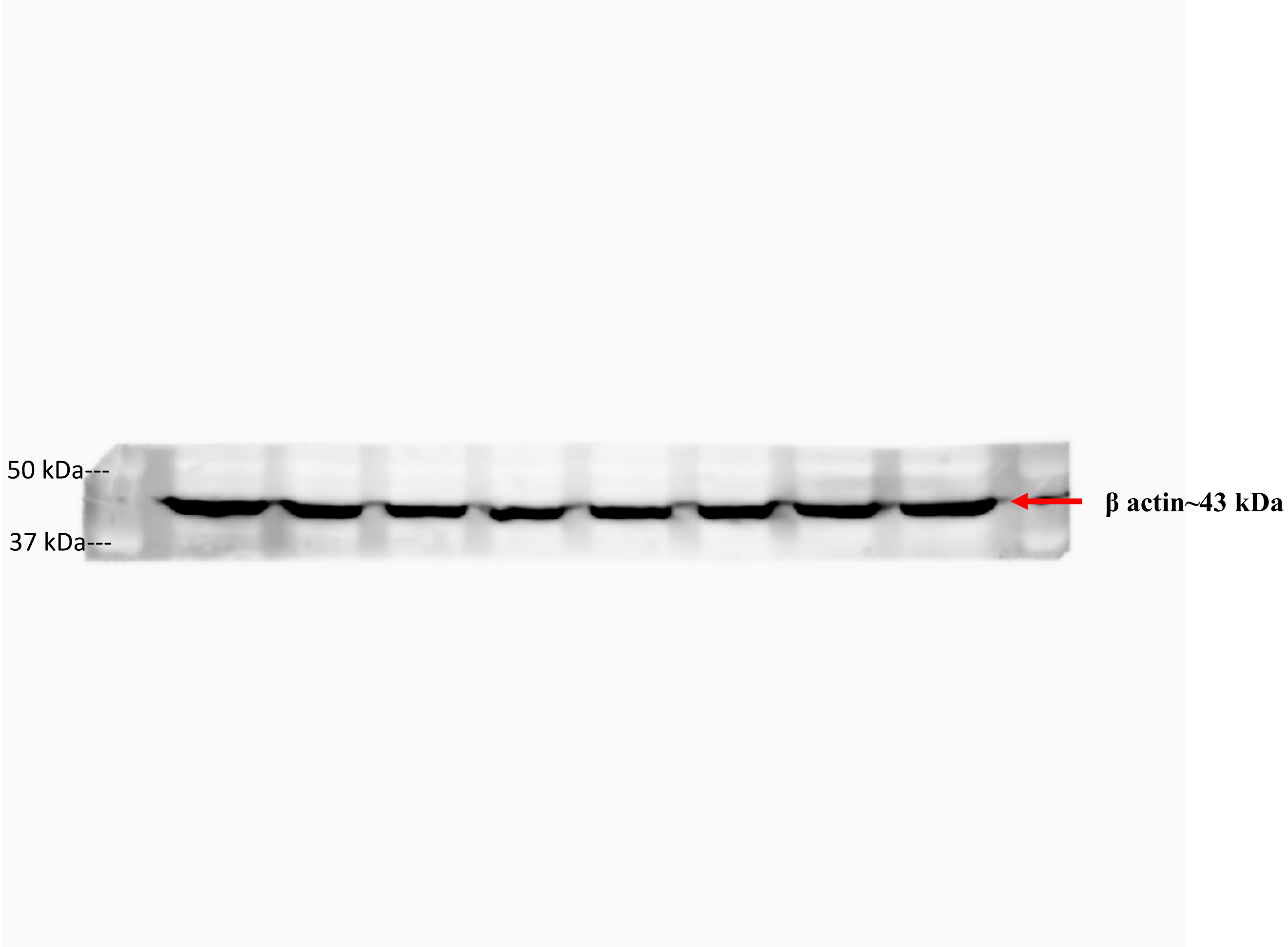

Figure S43. Uncropped PVDF membrane showing pp38 in KE5 cells. The raw image corresponds to the cropped data presented in Figure 6A.

Note: The membrane was sectioned according to the expected molecular weights of the target proteins before antibody incubation to enable the detection of multiple proteins from the same membrane. Representative blot images from the corresponding membrane sections are shown.

pp38 of KE5 cells (lane 1: NoLPS, lane 2: LPS-PG, lane 3: LPS-PG + EIF25, lane 4: LPS-PG + EIF50, lane 5: LPS-PG + EIF75, lane 6: LPS-PG + CGA5, lane 7: LPS-PG + CGA10, lane 8: LPS-PG + CGA20)

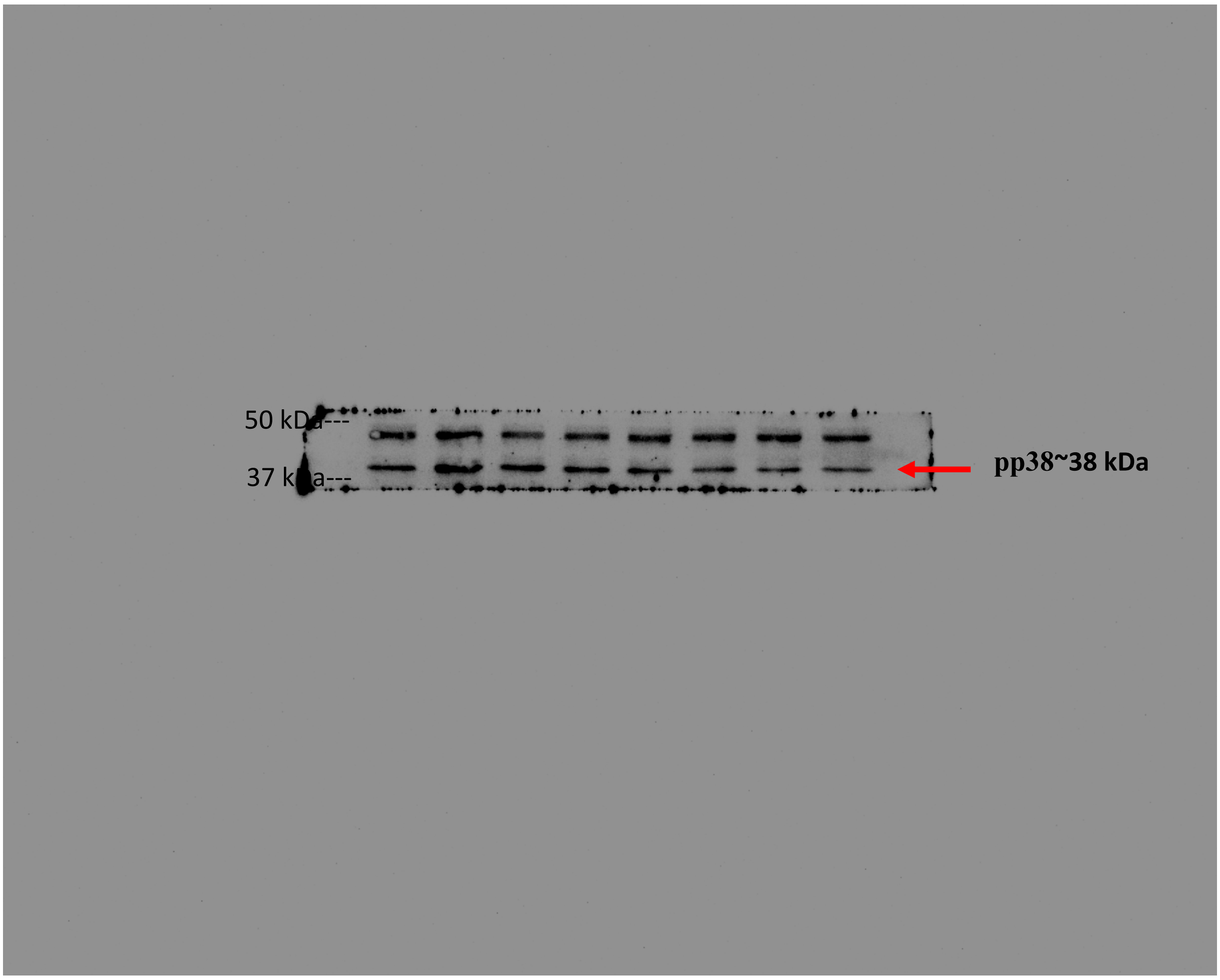

Figure S44. Uncropped PVDF membrane showing β-actin in KE5 cells. The raw image corresponds to the cropped data presented in Figure 6A.

Note: The membrane was sectioned according to the expected molecular weights of the target proteins before antibody incubation to enable the detection of multiple proteins from the same membrane. Representative blot images from the corresponding membrane sections are shown.

β-actin of KE5 cells (lane 1: NoLPS, lane 2: LPS-PG, lane 3: LPS-PG + EIF25, lane 4: LPS-PG + EIF50, lane 5: LPS-PG + EIF75, lane 6: LPS-PG + CGA5, lane 7: LPS-PG + CGA10, lane 8: LPS-PG + CGA20)

The identical β-actin band was utilized as a shared loading control for pp38 and p38 of KE5 cells, which were analyzed on the same membrane using cutting and stripping/reprobing methods to ensure accurate normalization.

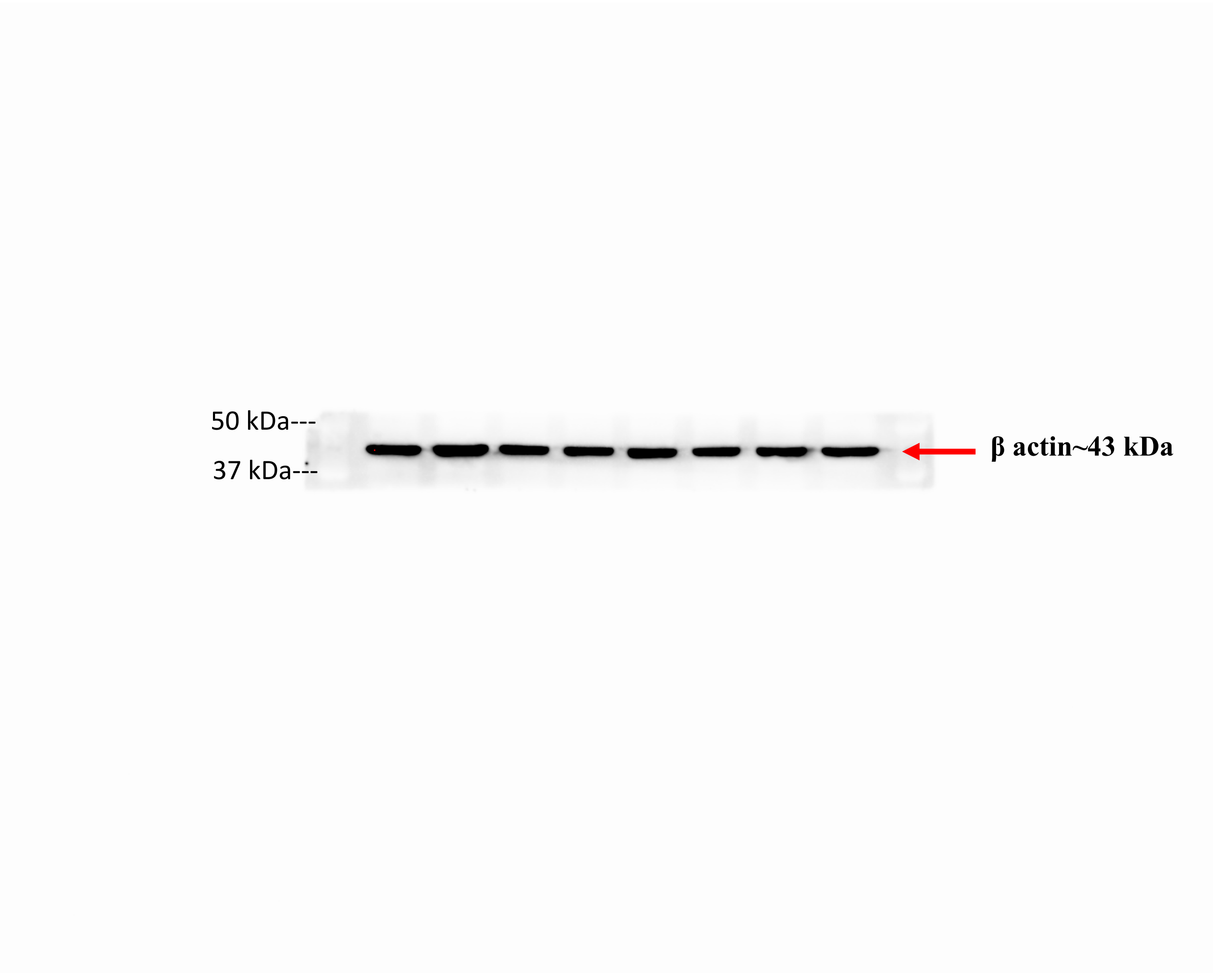

Figure S45. Uncropped PVDF membrane showing p38 in KE5 cells. The raw image corresponds to the cropped data presented in Figure 6A.

Note: The membrane was sectioned according to the expected molecular weights of the target proteins before antibody incubation to enable the detection of multiple proteins from the same membrane. Representative blot images from the corresponding membrane sections are shown.

p38 of KE5 cells (lane 1: NoLPS, lane 2: LPS-PG, lane 3: LPS-PG + EIF25, lane 4: LPS-PG + EIF50, lane 5: LPS-PG + EIF75, lane 6: LPS-PG + CGA5, lane 7: LPS-PG + CGA10, lane 8: LPS-PG + CGA20)

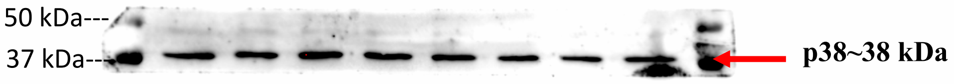

Figure S46. Uncropped PVDF membrane showing β-actin in KE5 cells. The raw image corresponds to the cropped data presented in Figure 6A.

Note: The membrane was sectioned according to the expected molecular weights of the target proteins before antibody incubation to enable the detection of multiple proteins from the same membrane. Representative blot images from the corresponding membrane sections are shown.

β-actin of KE5 cells (lane 1: NoLPS, lane 2: LPS-PG, lane 3: LPS-PG + EIF25, lane 4: LPS-PG + EIF50, lane 5: LPS-PG + EIF75, lane 6: LPS-PG + CGA5, lane 7: LPS-PG + CGA10, lane 8: LPS-PG + CGA20)

The identical β-actin band was utilized as a shared loading control for pp38 and p38 of KE5 cells, which were analyzed on the same membrane using cutting and stripping/reprobing methods to ensure accurate normalization.

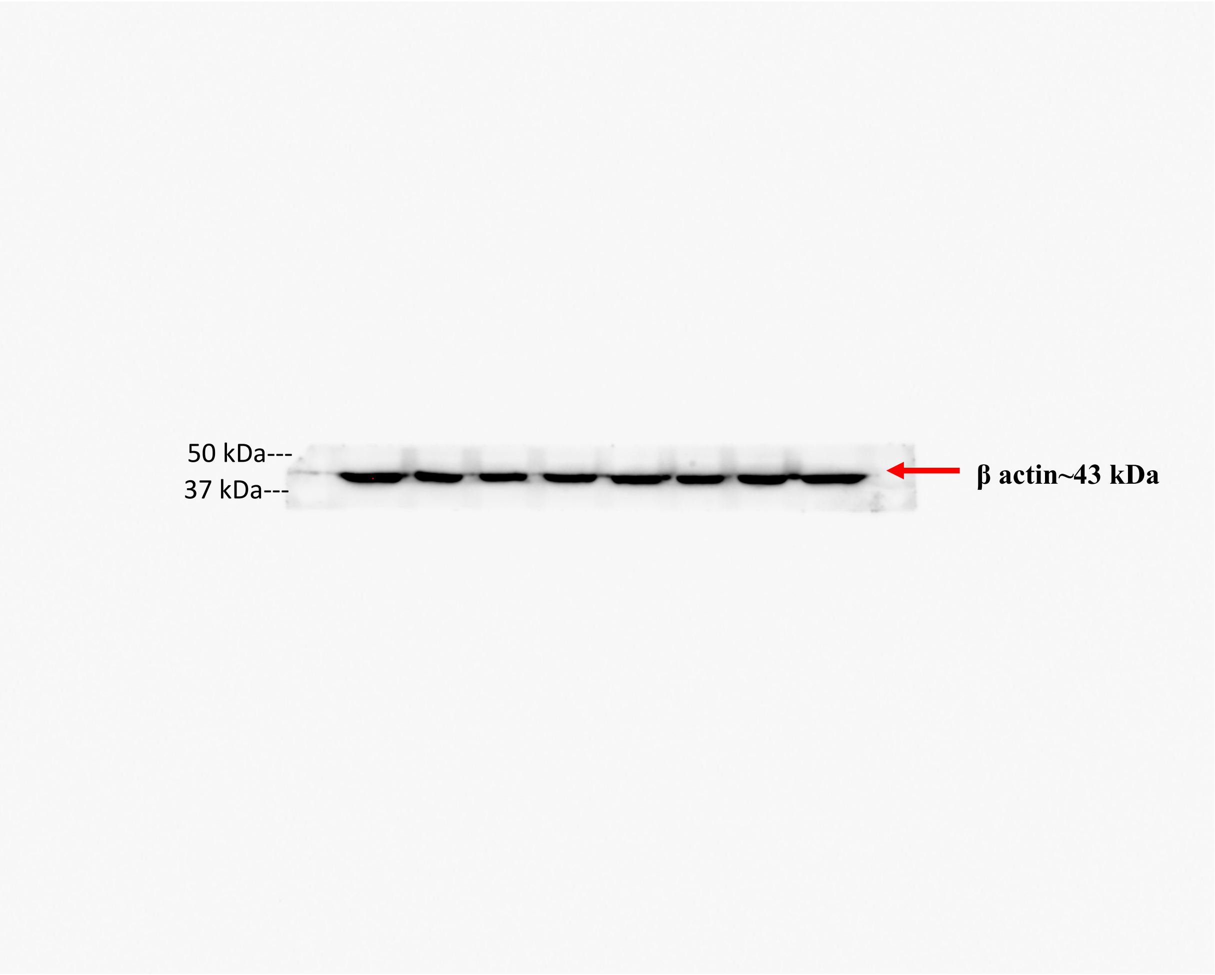

Figure S47. Uncropped PVDF membrane showing pp65 in T.T cells. The raw image corresponds to the cropped data presented in Figure 6B.

Note: The membrane was sectioned according to the expected molecular weights of the target proteins before antibody incubation to enable the detection of multiple proteins from the same membrane. Representative blot images from the corresponding membrane sections are shown.

pp65 of T.T cells (lane 1: NoLPS, lane 2: LPS-PG, lane 3: LPS-PG + EIF25, lane 4: LPS-PG + EIF50, lane 5: LPS-PG + EIF75, lane 6: LPS-PG + CGA5, lane 7: LPS-PG + CGA10, lane 8: LPS-PG + CGA20)

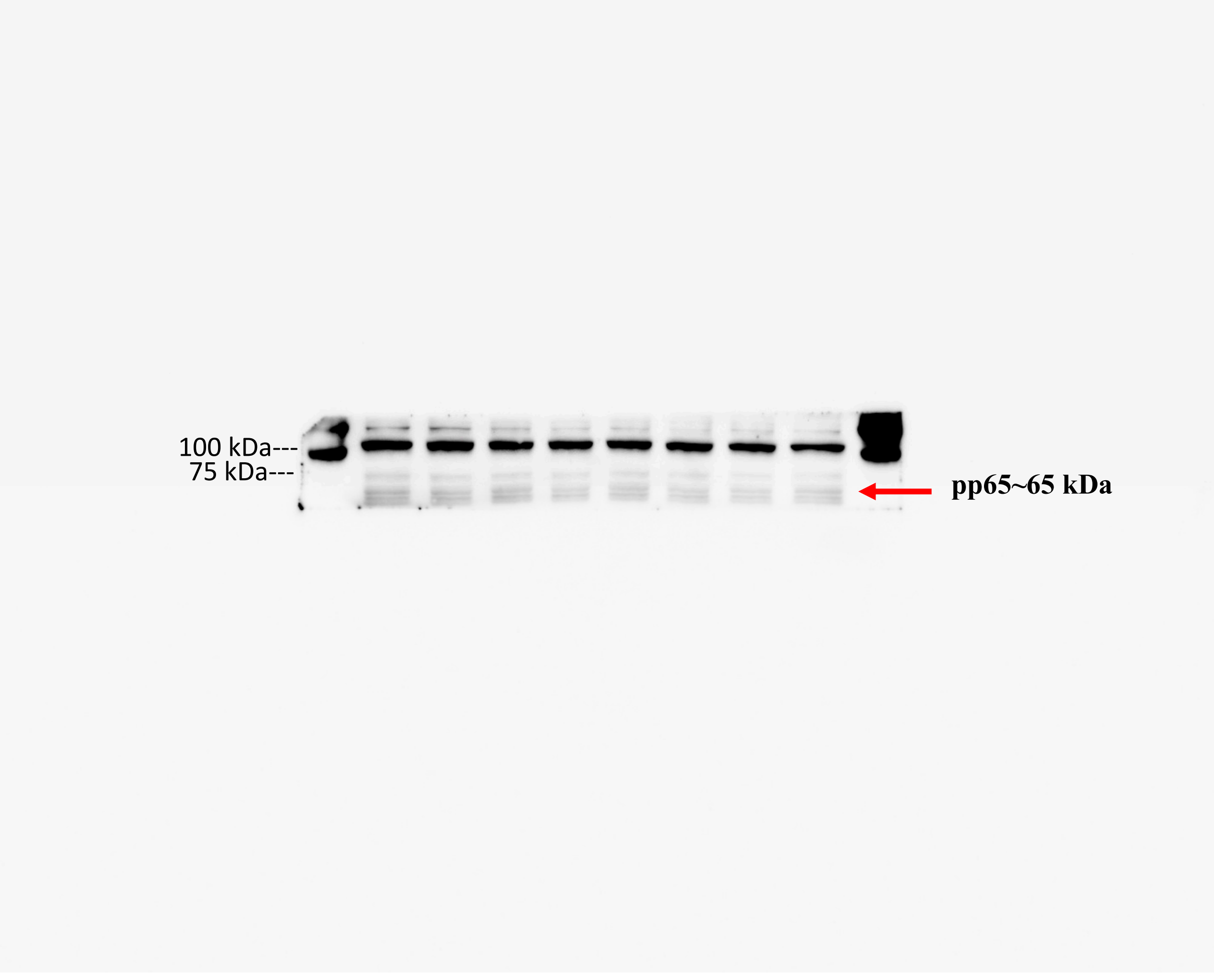

Figure S48. Uncropped PVDF membrane showing β-actin in T.T cells. The raw image corresponds to the cropped data presented in Figure 6B.

Note: The membrane was sectioned according to the expected molecular weights of the target proteins before antibody incubation to enable the detection of multiple proteins from the same membrane. Representative blot images from the corresponding membrane sections are shown.

β-actin of T.T cells (lane 1: NoLPS, lane 2: LPS-PG, lane 3: LPS-PG + EIF25, lane 4: LPS-PG + EIF50, lane 5: LPS-PG + EIF75, lane 6: LPS-PG + CGA5, lane 7: LPS-PG + CGA10, lane 8: LPS-PG + CGA20)

The identical β-actin band was utilized as a shared loading control for pp65 and p65 of T.T cells, which were analyzed on the same membrane using cutting and stripping/reprobing methods to ensure accurate normalization.

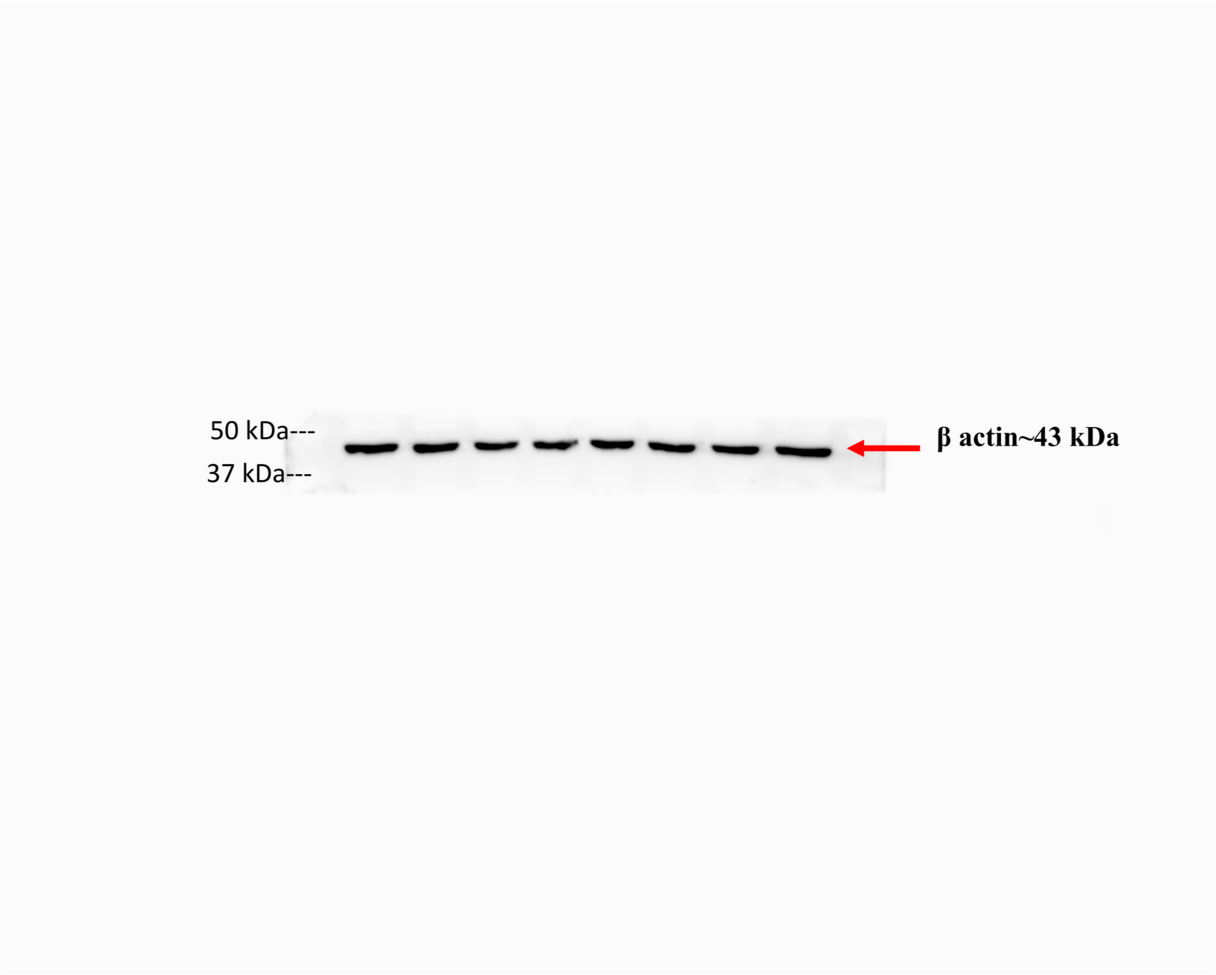

Figure S49. Uncropped PVDF membrane showing p65 in T.T cells. The raw image corresponds to the cropped data presented in Figure 6B.

Note: The membrane was sectioned according to the expected molecular weights of the target proteins before antibody incubation to enable the detection of multiple proteins from the same membrane. Representative blot images from the corresponding membrane sections are shown.

p65 of T.T cells (lane 1: NoLPS, lane 2: LPS-PG, lane 3: LPS-PG + EIF25, lane 4: LPS-PG + EIF50, lane 5: LPS-PG + EIF75, lane 6: LPS-PG + CGA5, lane 7: LPS-PG + CGA10, lane 8: LPS-PG + CGA20)

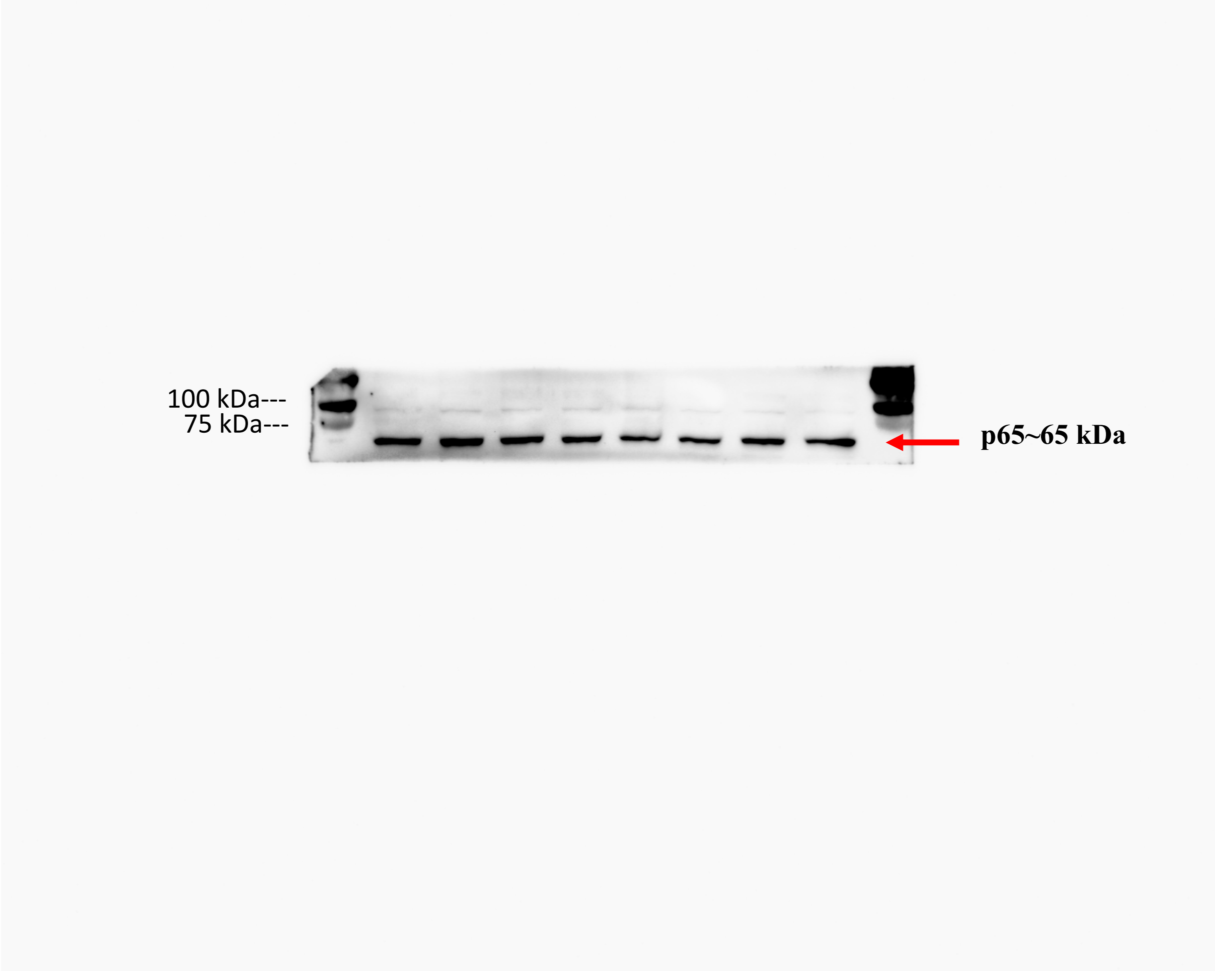

Figure S50. Uncropped PVDF membrane showing β-actin in T.T cells. The raw image corresponds to the cropped data presented in Figure 6B.

Note: The membrane was sectioned according to the expected molecular weights of the target proteins before antibody incubation to enable the detection of multiple proteins from the same membrane. Representative blot images from the corresponding membrane sections are shown.

β-actin of T.T cells (lane 1: NoLPS, lane 2: LPS-PG, lane 3: LPS-PG + EIF25, lane 4: LPS-PG + EIF50, lane 5: LPS-PG + EIF75, lane 6: LPS-PG + CGA5, lane 7: LPS-PG + CGA10, lane 8: LPS-PG + CGA20)

The identical β-actin band was utilized as a shared loading control for pp65 and p65 of T.T cells, which were analyzed on the same membrane using cutting and stripping/reprobing methods to ensure accurate normalization.

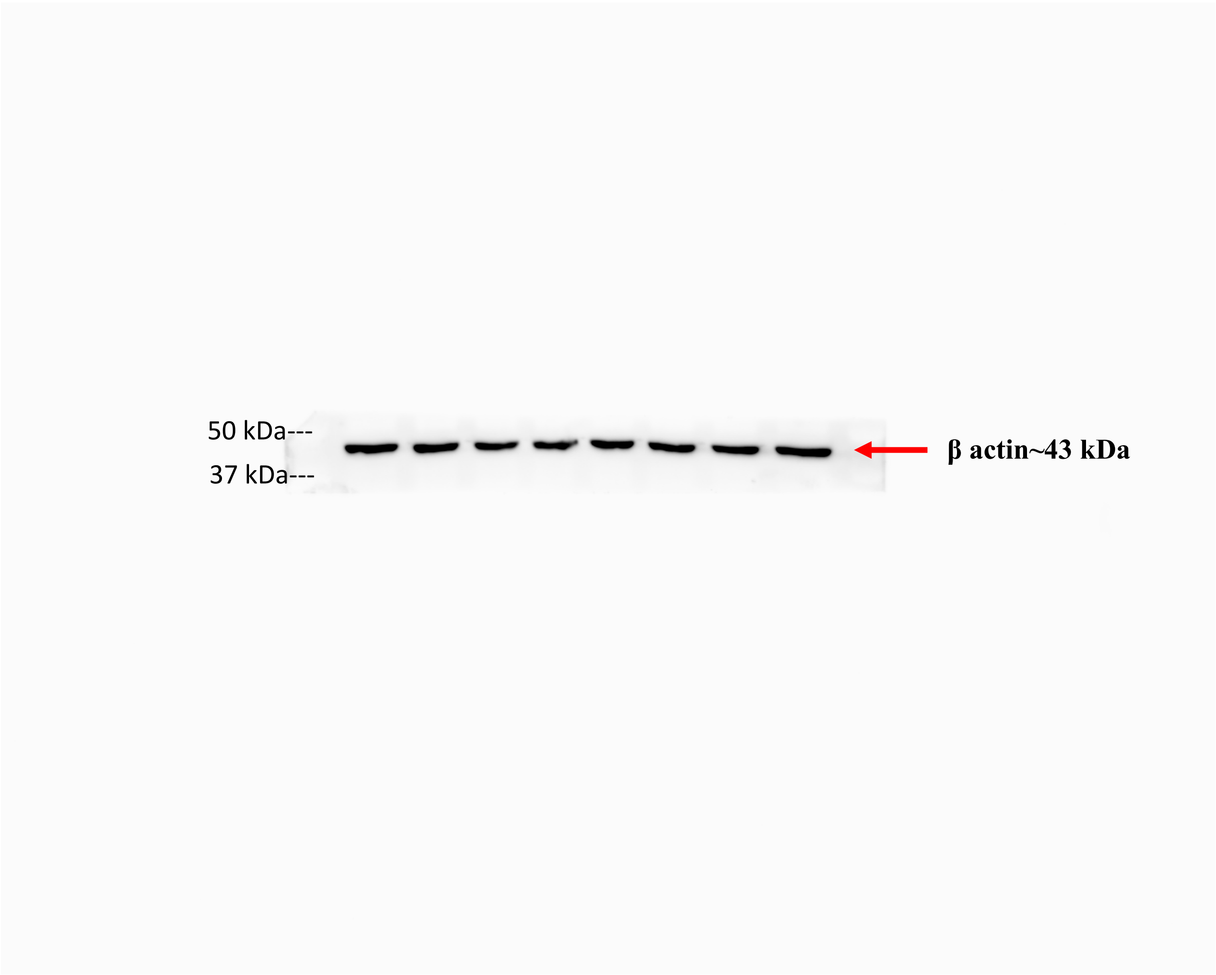

Figure S51. Uncropped PVDF membrane showing pp38 in T.T cells. The raw image corresponds to the cropped data presented in Figure 6B.

Note: The membrane was sectioned according to the expected molecular weights of the target proteins before antibody incubation to enable the detection of multiple proteins from the same membrane. Representative blot images from the corresponding membrane sections are shown.

pp38 of T.T cells (lane 1: NoLPS, lane 2: LPS-PG, lane 3: LPS-PG + EIF25, lane 4: LPS-PG + EIF50, lane 5: LPS-PG + EIF75, lane 6: LPS-PG + CGA5, lane 7: LPS-PG + CGA10, lane 8: LPS-PG + CGA20)

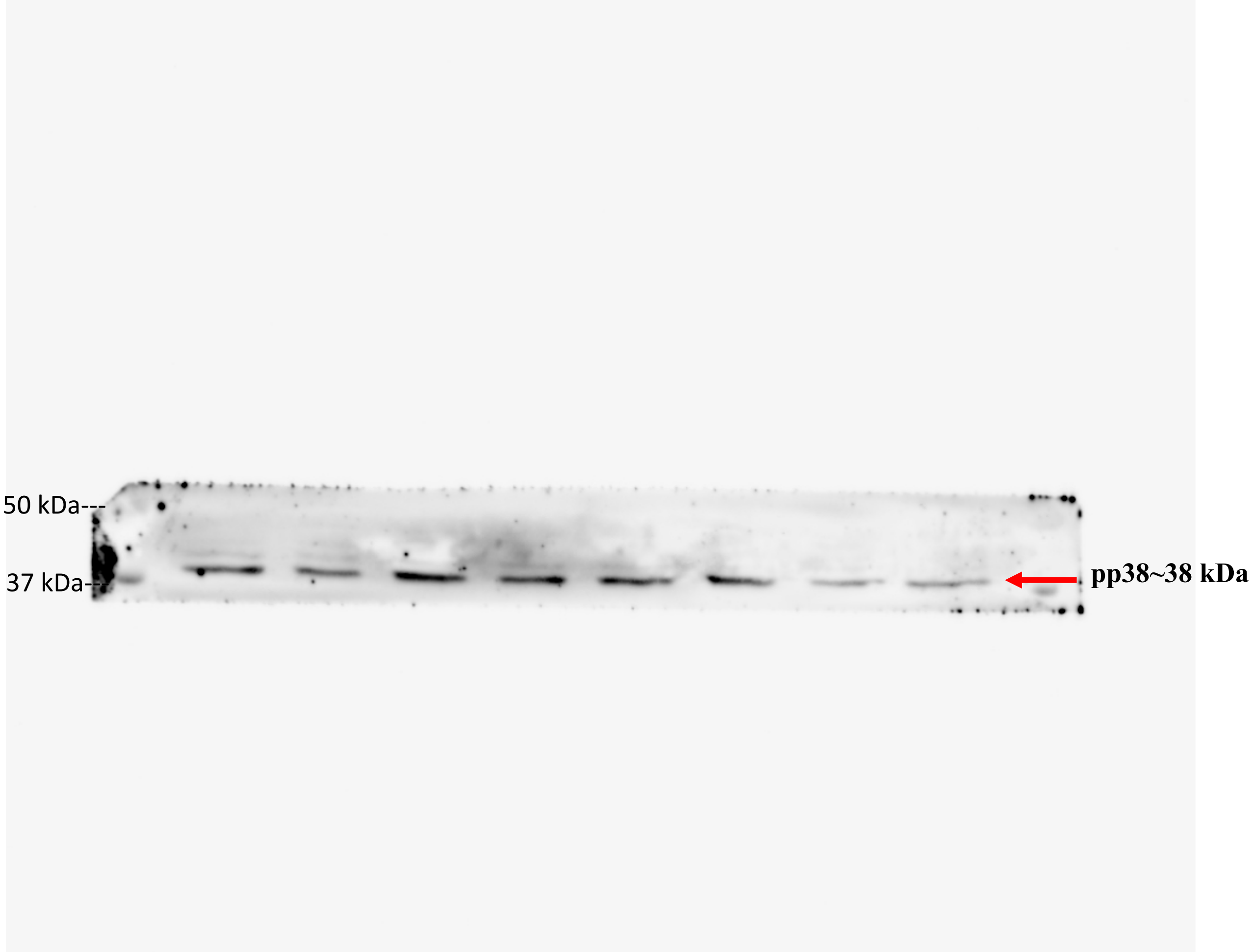

Figure S52. Uncropped PVDF membrane showing β-actin in T.T cells. The raw image corresponds to the cropped data presented in Figure 6B.

Note: The membrane was sectioned according to the expected molecular weights of the target proteins before antibody incubation to enable the detection of multiple proteins from the same membrane. Representative blot images from the corresponding membrane sections are shown.

β-actin of T.T cells (lane 1: NoLPS, lane 2: LPS-PG, lane 3: LPS-PG + EIF25, lane 4: LPS-PG + EIF50, lane 5: LPS-PG + EIF75, lane 6: LPS-PG + CGA5, lane 7: LPS-PG + CGA10, lane 8: LPS-PG + CGA20)

The identical β-actin band was utilized as a shared loading control for uPAR, pp38, and p38 of T.T cells, which were analyzed on the same membrane using cutting and stripping/reprobing methods to ensure accurate normalization.

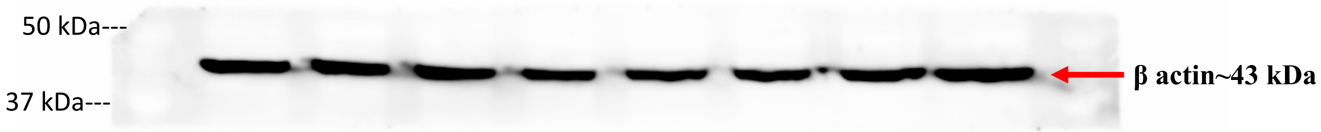

Figure S53. Uncropped PVDF membrane showing p38 in T.T cells. The raw image corresponds to the cropped data presented in Figure 6B.

Note: The membrane was sectioned according to the expected molecular weights of the target proteins before antibody incubation to enable the detection of multiple proteins from the same membrane. Representative blot images from the corresponding membrane sections are shown.

p38 of T.T cells (lane 1: NoLPS, lane 2: LPS-PG, lane 3: LPS-PG + EIF25, lane 4: LPS-PG + EIF50, lane 5: LPS-PG + EIF75, lane 6: LPS-PG + CGA5, lane 7: LPS-PG + CGA10, lane 8: LPS-PG + CGA20)

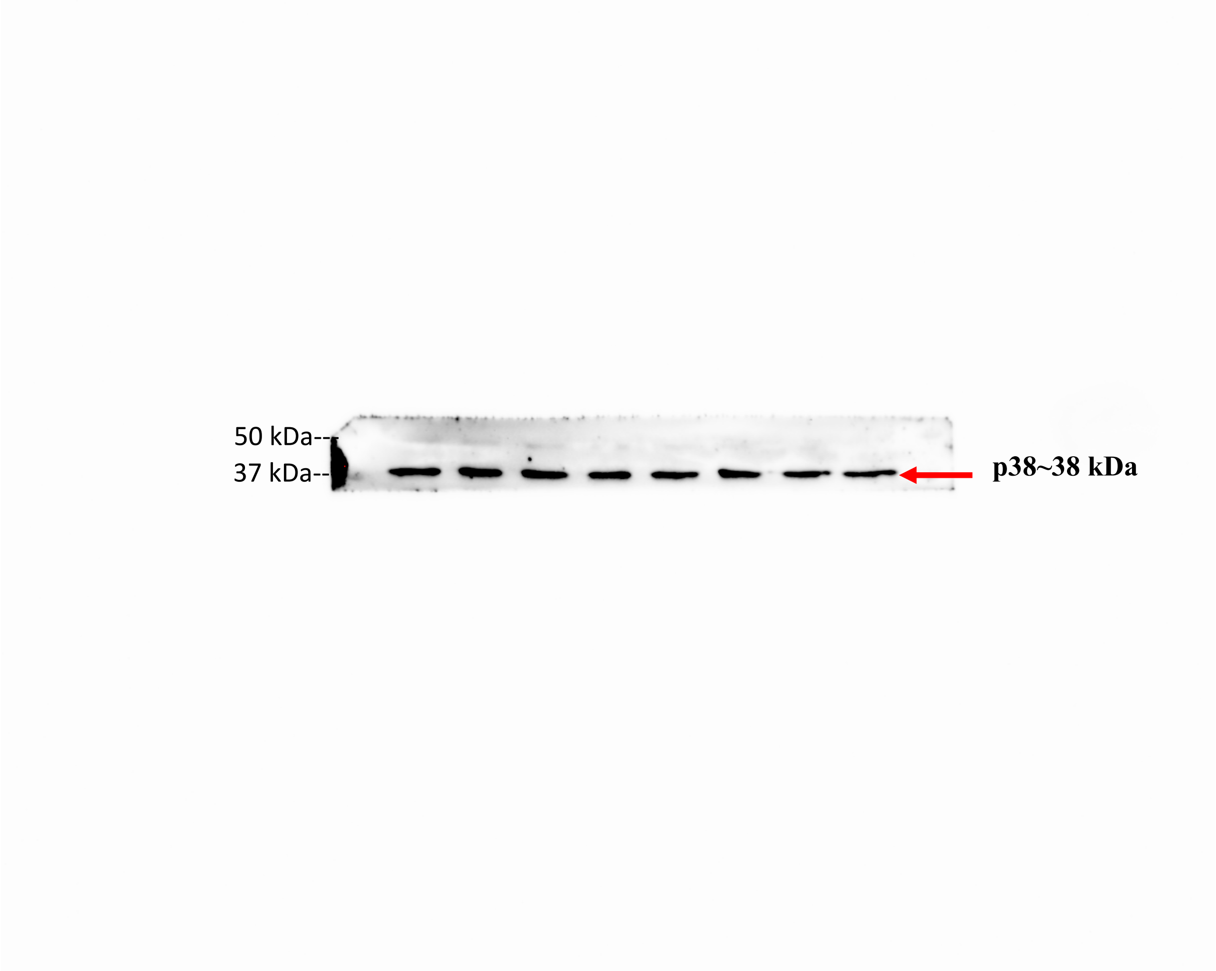

Figure S54. Uncropped PVDF membrane showing β-actin in T.T cells. The raw image corresponds to the cropped data presented in Figure 6B.

Note: The membrane was sectioned according to the expected molecular weights of the target proteins before antibody incubation to enable the detection of multiple proteins from the same membrane. Representative blot images from the corresponding membrane sections are shown.

β-actin of T.T cells (lane 1: NoLPS, lane 2: LPS-PG, lane 3: LPS-PG + EIF25, lane 4: LPS-PG + EIF50, lane 5: LPS-PG + EIF75, lane 6: LPS-PG + CGA5, lane 7: LPS-PG + CGA10, lane 8: LPS-PG + CGA20)

The identical β-actin band was utilized as a shared loading control for uPAR, pp38, and p38 of T.T cells, which were analyzed on the same membrane using cutting and stripping/reprobing methods to ensure accurate normalization.

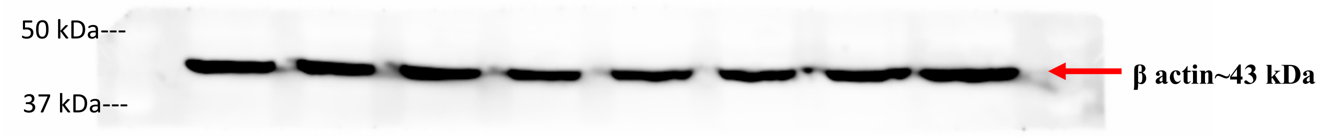

Figure S55. Uncropped PVDF membrane showing TLR4 in T.T cells. The raw image corresponds to the cropped data presented in Figure 7B.

Note: The membrane was sectioned according to the expected molecular weights of the target proteins before antibody incubation to enable the detection of multiple proteins from the same membrane. Representative blot images from the corresponding membrane sections are shown.

TLR4 of T.T cells (lane 1: NoLPS, lane 2: LPS-PG, lane 3: LPS-PG + PTX3, lane 4: LPS-PG + PTX6, lane 5: LPS-PG + PTX9, lane 6: LPS-PG + PTX3 + EIF25, lane 7: LPS-PG + PTX3 + EIF50, lane 8: LPS-PG + PTX3 + EIF75)

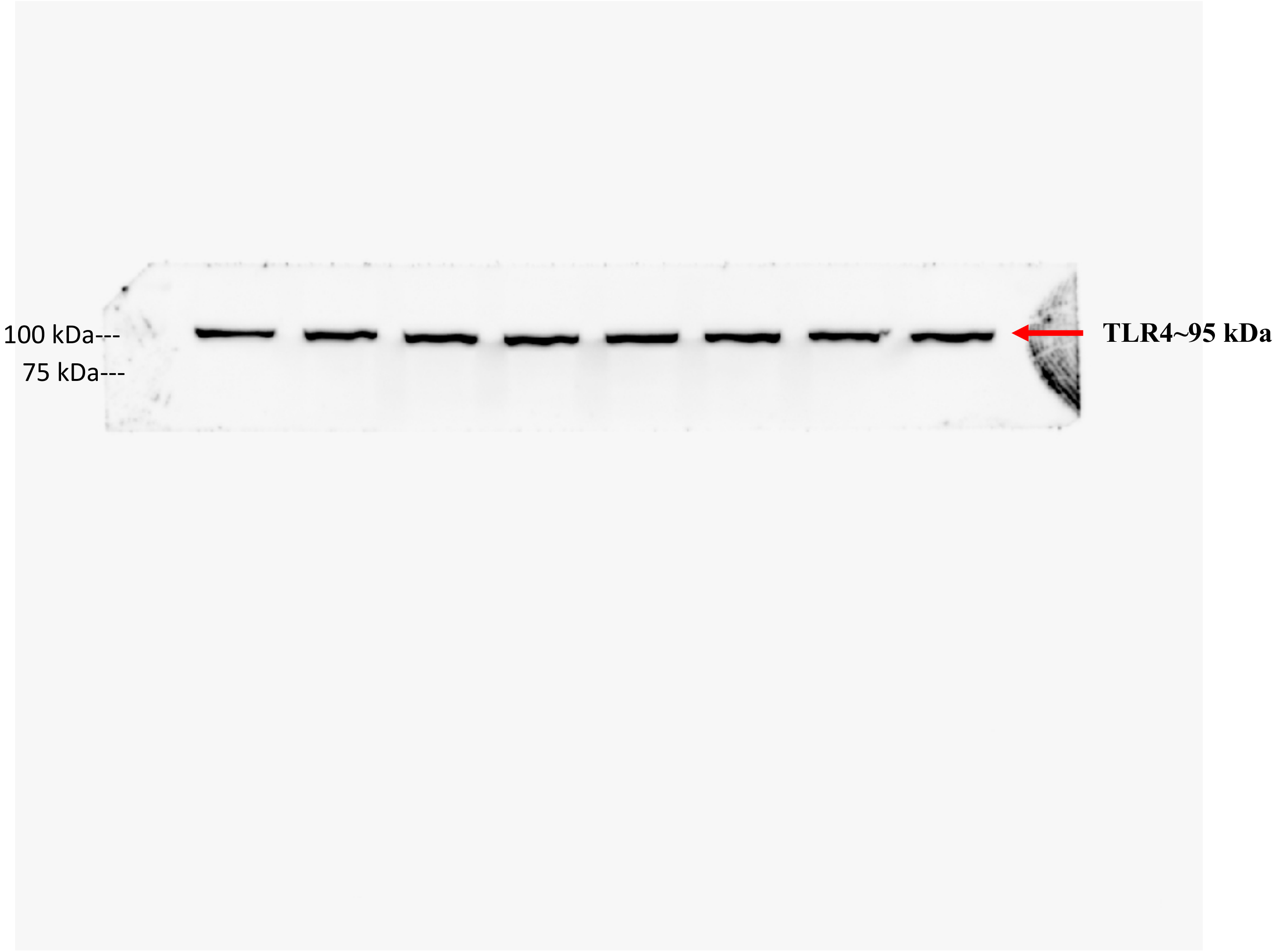

Figure S56. Uncropped PVDF membrane showing β-actin in T.T cells. The raw image corresponds to the cropped data presented in Figure 7B.

Note: The membrane was sectioned according to the expected molecular weights of the target proteins before antibody incubation to enable the detection of multiple proteins from the same membrane. Representative blot images from the corresponding membrane sections are shown.

β-actin of T.T cells (lane 1: NoLPS, lane 2: LPS-PG, lane 3: LPS-PG + PTX3, lane 4: LPS-PG + PTX6, lane 5: LPS-PG + PTX9, lane 6: LPS-PG + PTX3 + EIF25, lane 7: LPS-PG + PTX3 + EIF50, lane 8: LPS-PG + PTX3 + EIF75)

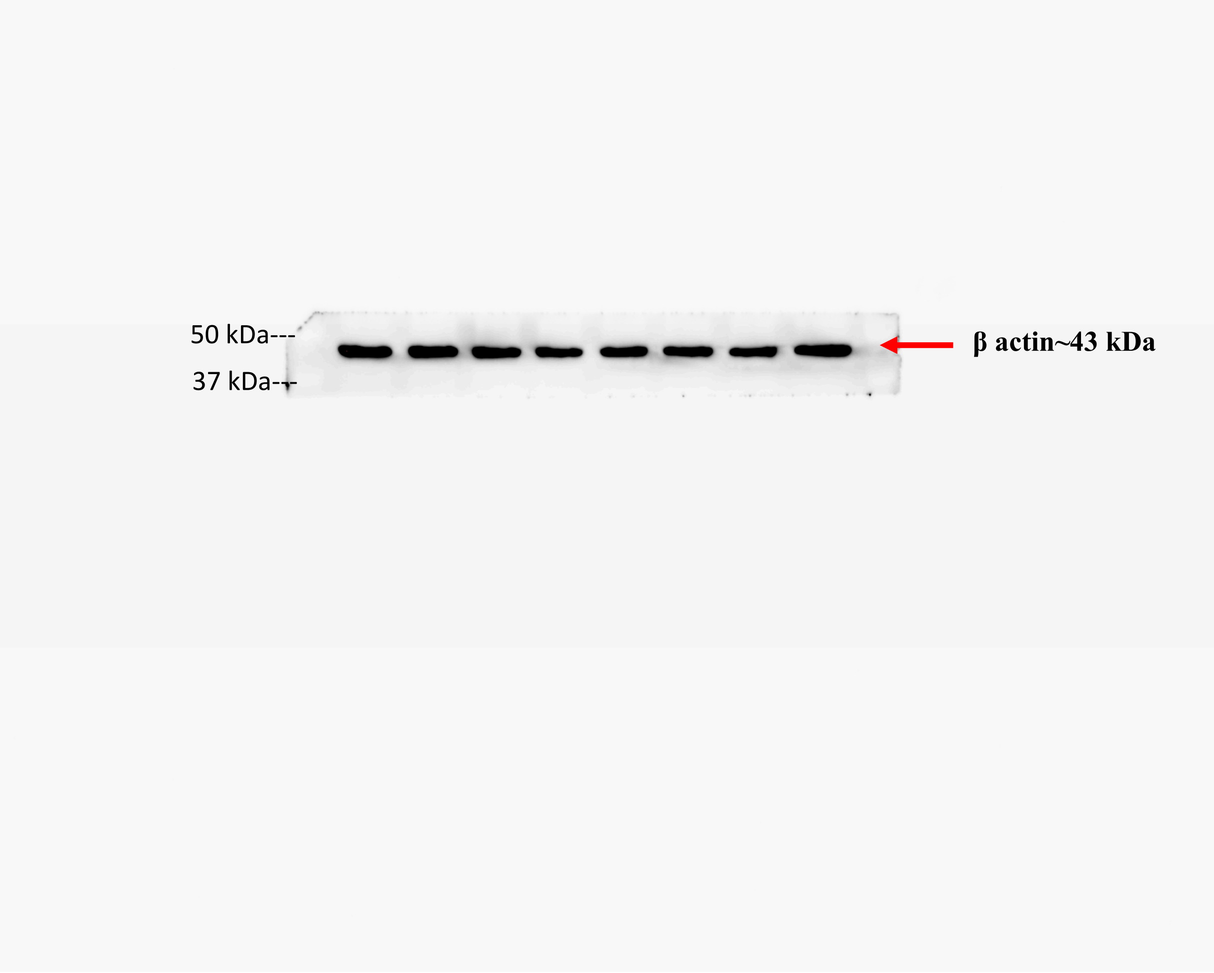

Figure S57. Uncropped PVDF membrane showing COX2 in T.T cells. The raw image corresponds to the cropped data presented in Figure 7B.

Note: The membrane was sectioned according to the expected molecular weights of the target proteins before antibody incubation to enable the detection of multiple proteins from the same membrane. Representative blot images from the corresponding membrane sections are shown.

COX2 of T.T cells (lane 1: NoLPS, lane 2: LPS-PG, lane 3: LPS-PG + PTX3, lane 4: LPS-PG + PTX6, lane 5: LPS-PG + PTX9, lane 6: LPS-PG + PTX3 + EIF25, lane 7: LPS-PG + PTX3 + EIF50, lane 8: LPS-PG + PTX3 + EIF75)

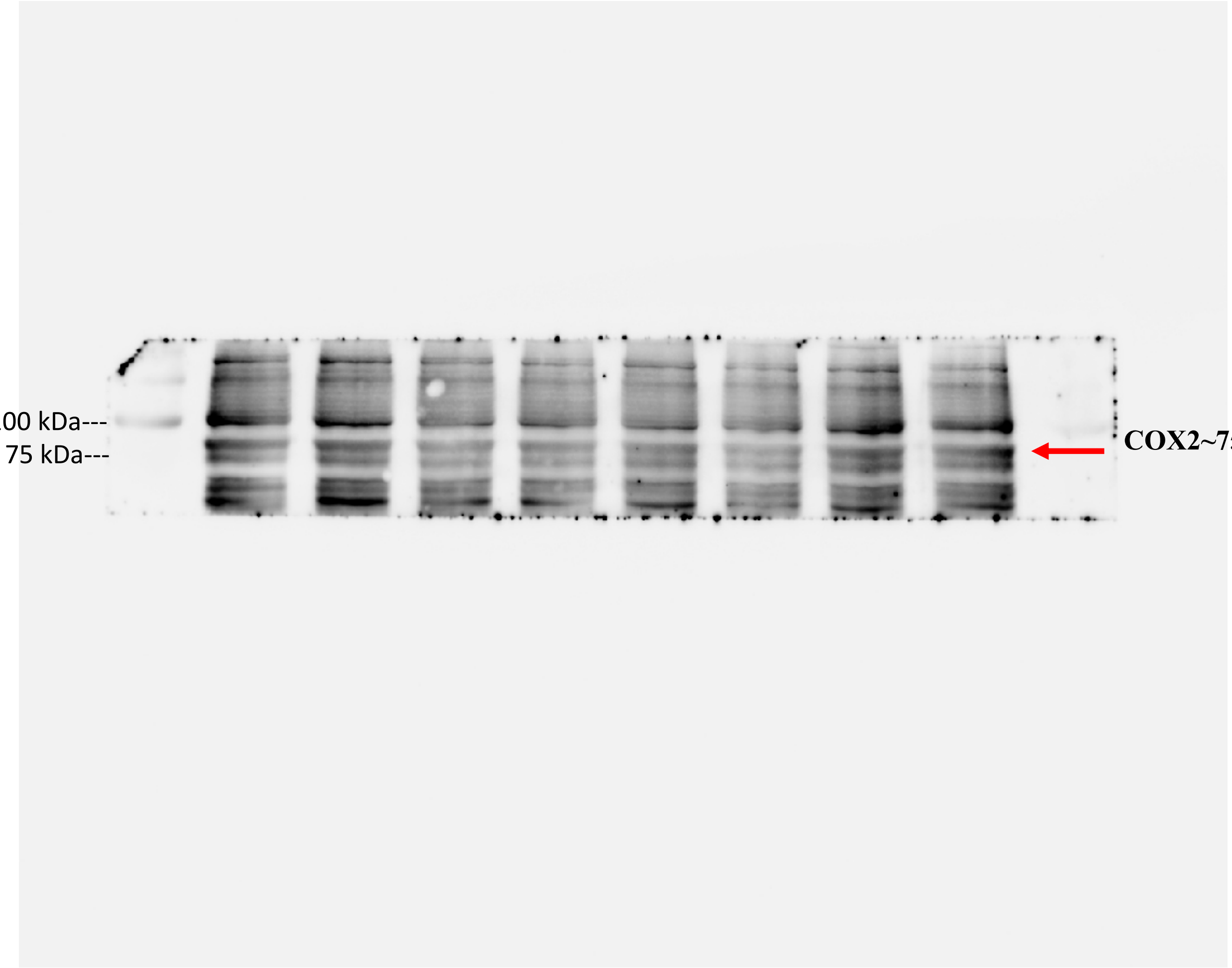

Figure S58. Uncropped PVDF membrane showing β-actin in T.T cells. The raw image corresponds to the cropped data presented in Figure 7B.

Note: The membrane was sectioned according to the expected molecular weights of the target proteins before antibody incubation to enable the detection of multiple proteins from the same membrane. Representative blot images from the corresponding membrane sections are shown.

β-actin of T.T cells (lane 1: NoLPS, lane 2: LPS-PG, lane 3: LPS-PG + PTX3, lane 4: LPS-PG + PTX6, lane 5: LPS-PG + PTX9, lane 6: LPS-PG + PTX3 + EIF25, lane 7: LPS-PG + PTX3 + EIF50, lane 8: LPS-PG + PTX3 + EIF75)

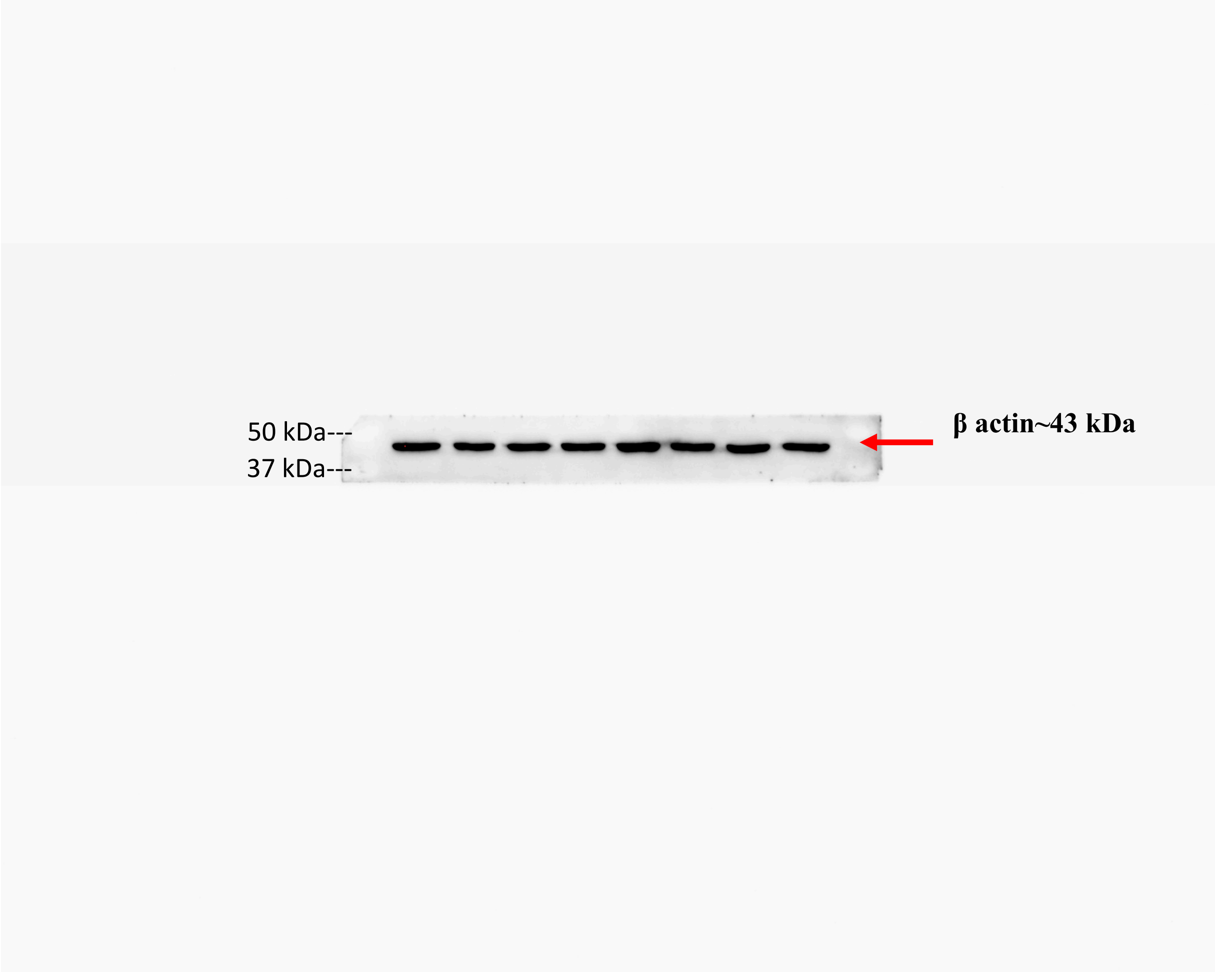

Figure S59. Uncropped PVDF membrane showing IL-1β in T.T cells**.** The raw image corresponds to the cropped data presented in Figure 7B.

Note: The membrane was sectioned according to the expected molecular weights of the target proteins before antibody incubation to enable the detection of multiple proteins from the same membrane. Representative blot images from the corresponding membrane sections are shown.

IL-1β of T.T cells (lane 1: NoLPS, lane 2: LPS-PG, lane 3: LPS-PG + PTX3, lane 4: LPS-PG + PTX6, lane 5: LPS-PG + PTX9, lane 6: LPS-PG + PTX3 + EIF25, lane 7: LPS-PG + PTX3 + EIF50, lane 8: LPS-PG + PTX3 + EIF75)

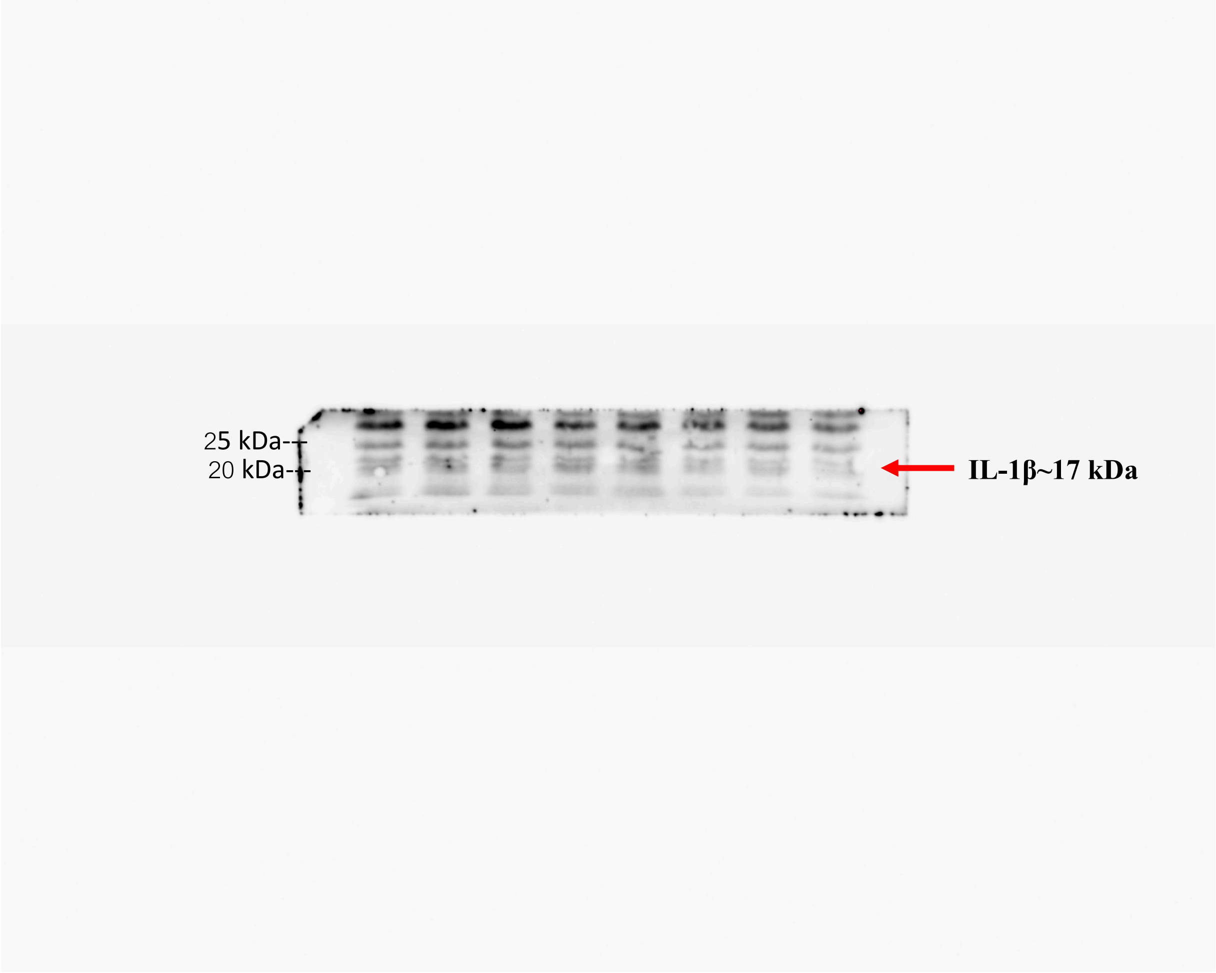

Figure S60. Uncropped PVDF membrane showing β-actin in T.T cells. The raw image corresponds to the cropped data presented in Figure 7B.

Note: The membrane was sectioned according to the expected molecular weights of the target proteins before antibody incubation to enable the detection of multiple proteins from the same membrane. Representative blot images from the corresponding membrane sections are shown.

β-actin of T.T cells (lane 1: NoLPS, lane 2: LPS-PG, lane 3: LPS-PG + PTX3, lane 4: LPS-PG + PTX6, lane 5: LPS-PG + PTX9, lane 6: LPS-PG + PTX3 + EIF25, lane 7: LPS-PG + PTX3 + EIF50, lane 8: LPS-PG + PTX3 + EIF75)

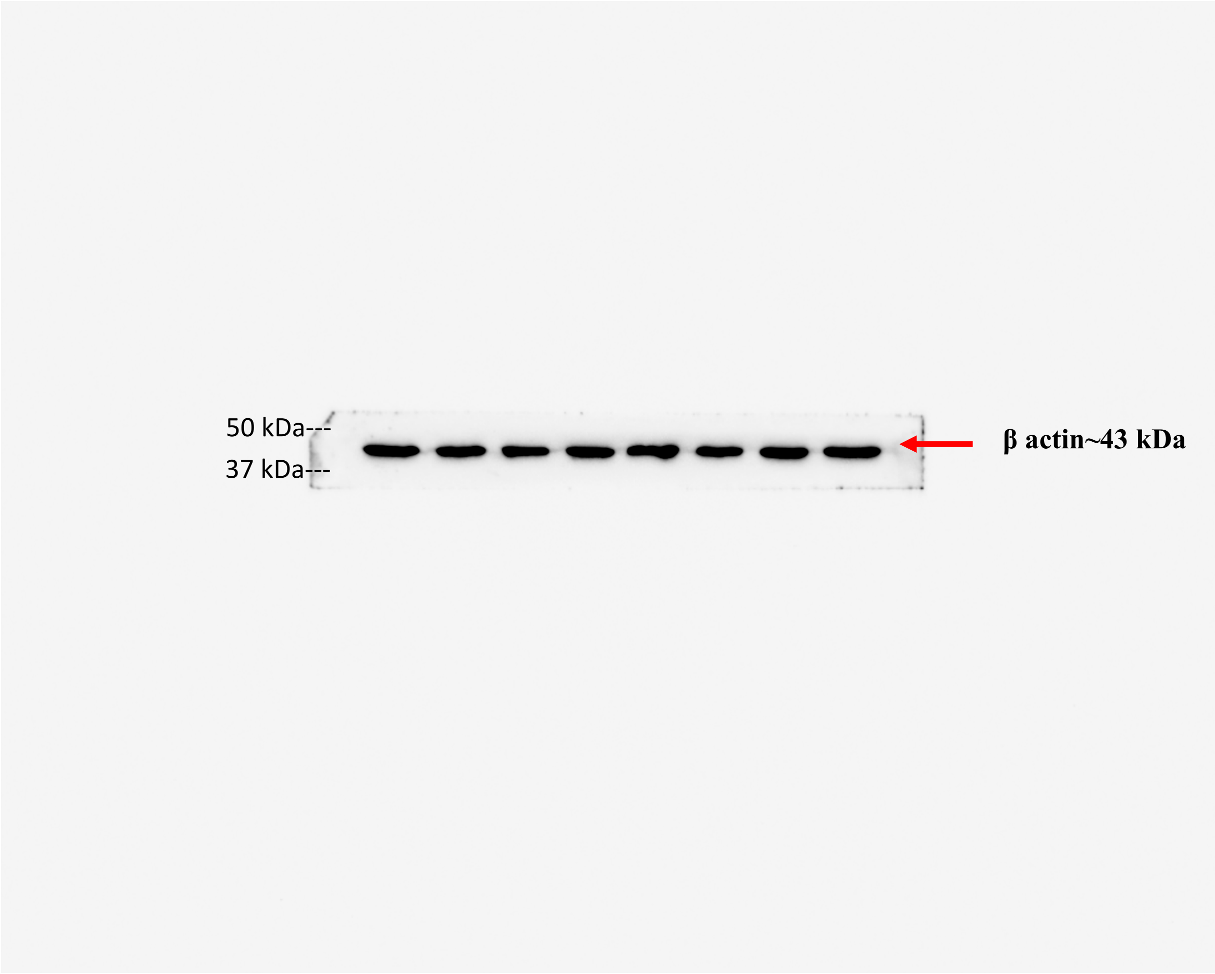

Figure S61. Uncropped PVDF membrane showing TNF-α in T.T cells. The raw image corresponds to the cropped data presented in Figure 7B.

Note: The membrane was sectioned according to the expected molecular weights of the target proteins before antibody incubation to enable the detection of multiple proteins from the same membrane. Representative blot images from the corresponding membrane sections are shown.

TNF-α of T.T cells (lane 1: NoLPS, lane 2: LPS-PG, lane 3: LPS-PG + PTX3, lane 4: LPS-PG + PTX6, lane 5: LPS-PG + PTX9, lane 6: LPS-PG + PTX3 + EIF25, lane 7: LPS-PG + PTX3 + EIF50, lane 8: LPS-PG + PTX3 + EIF75)

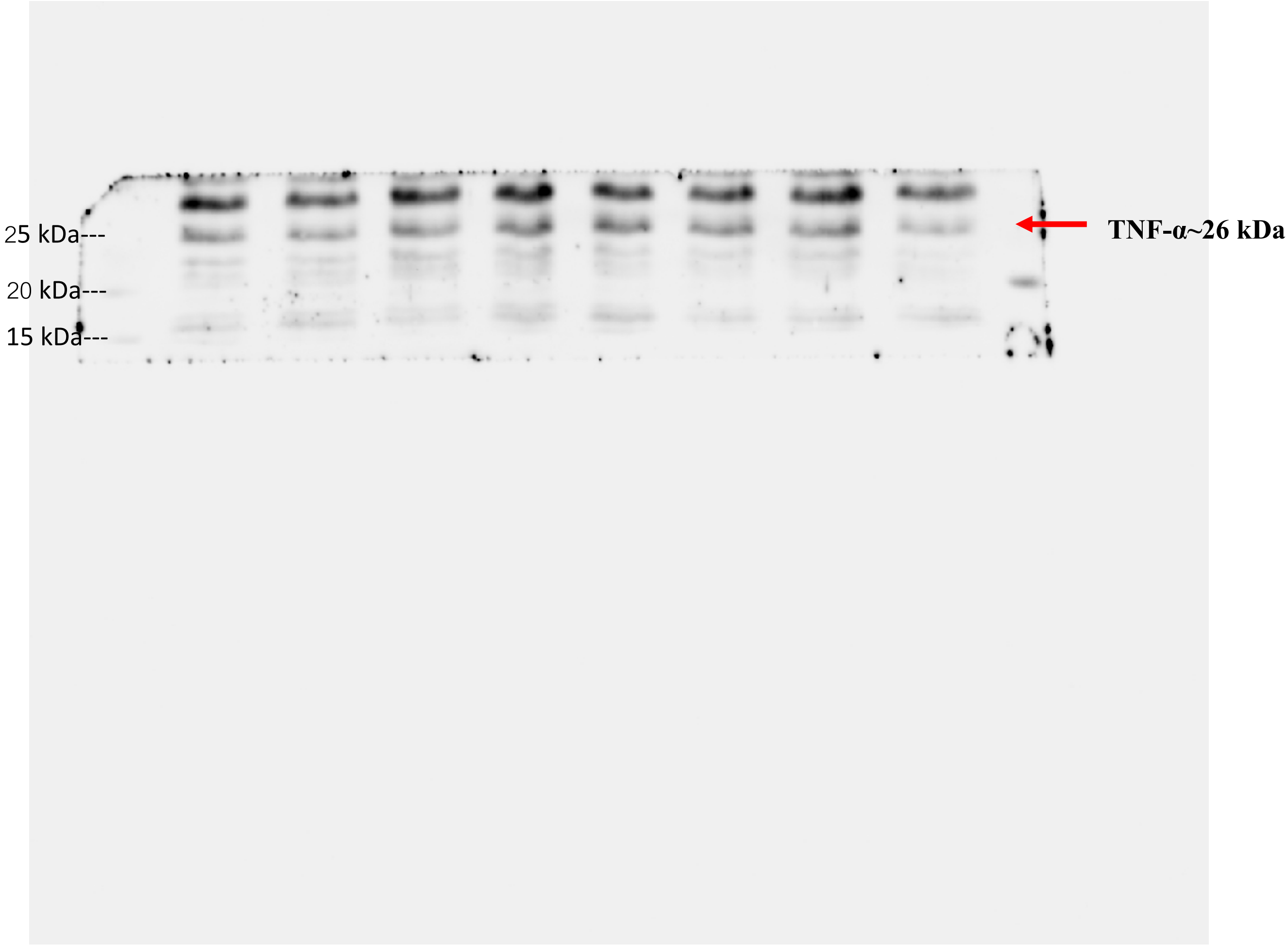

Figure S62. Uncropped PVDF membrane showing β-actin in T.T cells. The raw image corresponds to the cropped data presented in Figure 7B.

Note: The membrane was sectioned according to the expected molecular weights of the target proteins before antibody incubation to enable the detection of multiple proteins from the same membrane. Representative blot images from the corresponding membrane sections are shown.

β-actin of T.T cells (lane 1: NoLPS, lane 2: LPS-PG, lane 3: LPS-PG + PTX3, lane 4: LPS-PG + PTX6, lane 5: LPS-PG + PTX9, lane 6: LPS-PG + PTX3 + EIF25, lane 7: LPS-PG + PTX3 + EIF50, lane 8: LPS-PG + PTX3 + EIF75)

The identical β-actin band was utilized as a shared loading control for TNF-α and uPAR of T.T cells, which were analyzed on the same membrane using cutting and stripping/reprobing methods to ensure accurate normalization.

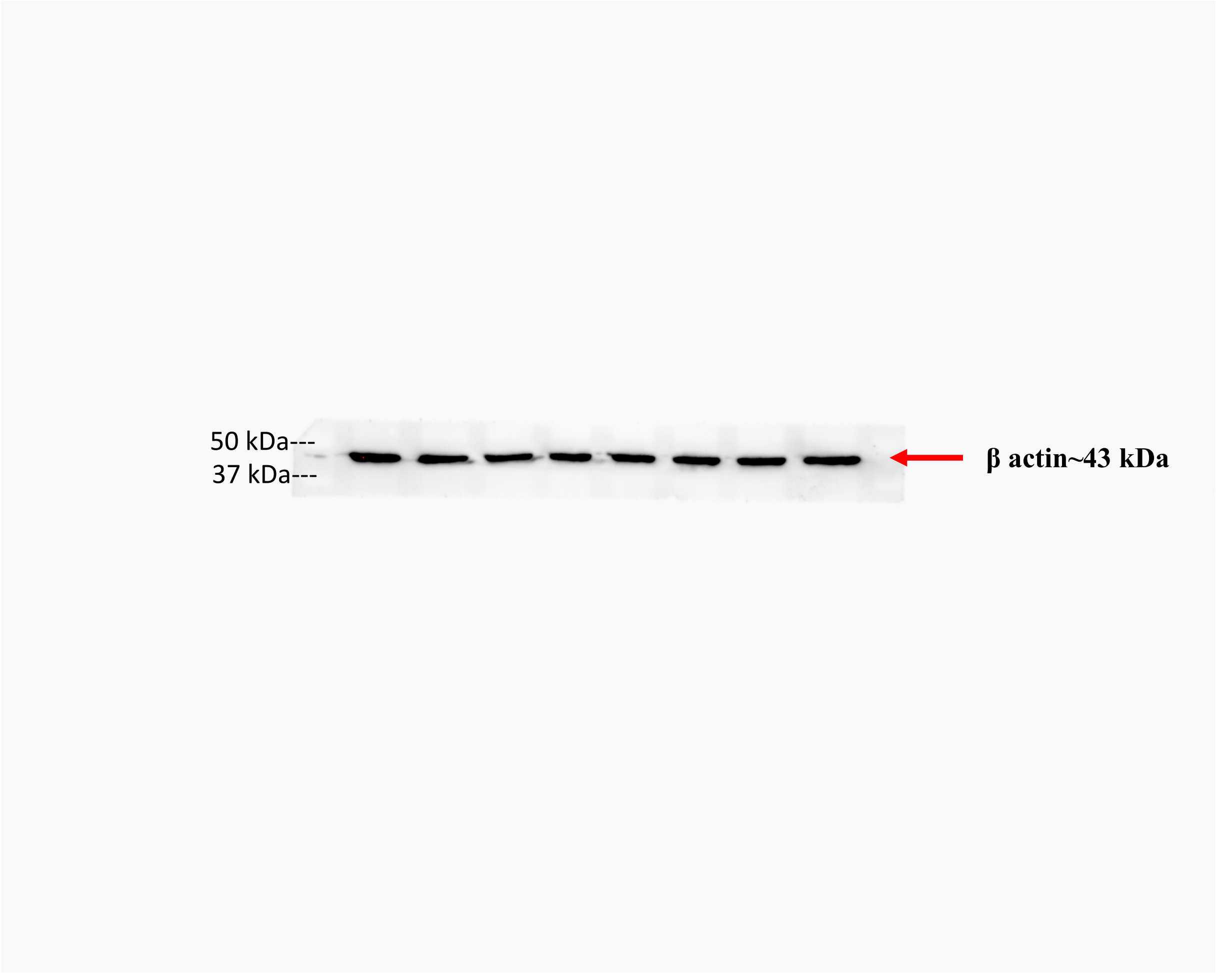

Figure S63. Uncropped PVDF membrane showing uPAR in T.T cells. The raw image corresponds to the cropped data presented in Figure 7C.

Note: The membrane was sectioned according to the expected molecular weights of the target proteins before antibody incubation to enable the detection of multiple proteins from the same membrane. Representative blot images from the corresponding membrane sections are shown.

uPAR of T.T cells (lane 1: NoLPS, lane 2: LPS-PG, lane 3: LPS-PG + PTX3, lane 4: LPS-PG + PTX6, lane 5: LPS-PG + PTX9, lane 6: LPS-PG + PTX3 + EIF25, lane 7: LPS-PG + PTX3 + EIF50, lane 8: LPS-PG + PTX3 + EIF75)

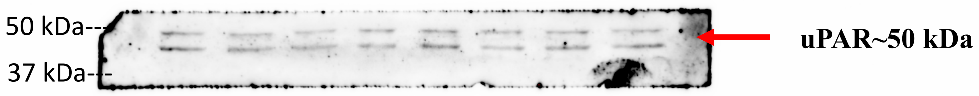

Figure S64. Uncropped PVDF membrane showing β-actin in T.T cells. The raw image corresponds to the cropped data presented in Figure 7C.

Note: The membrane was sectioned according to the expected molecular weights of the target proteins before antibody incubation to enable the detection of multiple proteins from the same membrane. Representative blot images from the corresponding membrane sections are shown.

β-actin of T.T cells (lane 1: NoLPS, lane 2: LPS-PG, lane 3: LPS-PG + PTX3, lane 4: LPS-PG + PTX6, lane 5: LPS-PG + PTX9, lane 6: LPS-PG + PTX3 + EIF25, lane 7: LPS-PG + PTX3 + EIF50, lane 8: LPS-PG + PTX3 + EIF75)

The identical β-actin band was utilized as a shared loading control for TNF-α and uPAR of T.T cells , which were analyzed on the same membrane using cutting and stripping/reprobing methods to ensure accurate normalization.

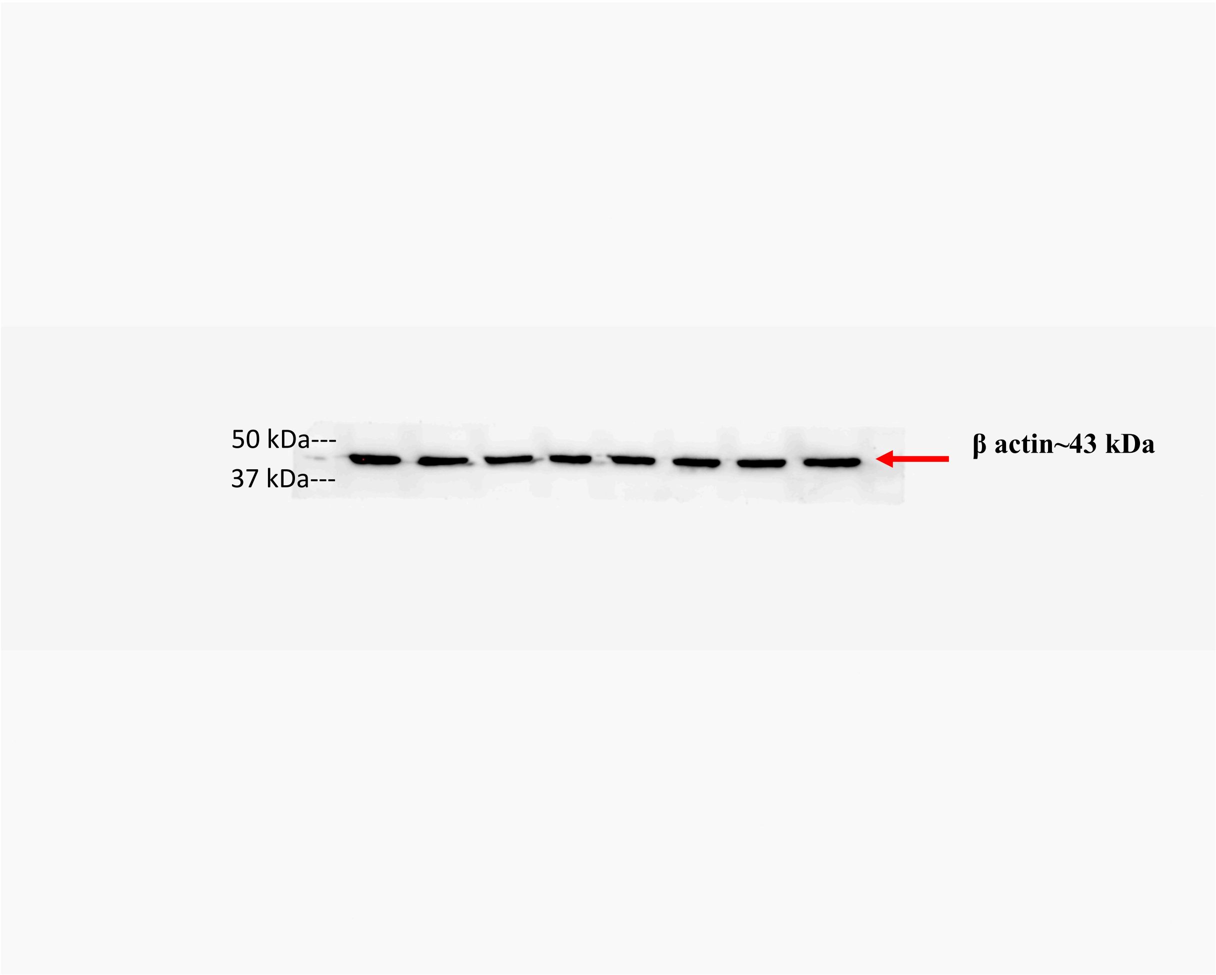

Figure S65. Uncropped PVDF membrane showing uPA in T.T cells. The raw image corresponds to the cropped data presented in Figure 7C.

Note: The membrane was sectioned according to the expected molecular weights of the target proteins before antibody incubation to enable the detection of multiple proteins from the same membrane. Representative blot images from the corresponding membrane sections are shown.

uPA of T.T cells (lane 1: NoLPS, lane 2: LPS-PG, lane 3: LPS-PG + PTX3, lane 4: LPS-PG + PTX6, lane 5: LPS-PG + PTX9, lane 6: LPS-PG + PTX3 + EIF25, lane 7: LPS-PG + PTX3 + EIF50, lane 8: LPS-PG + PTX3 + EIF75)

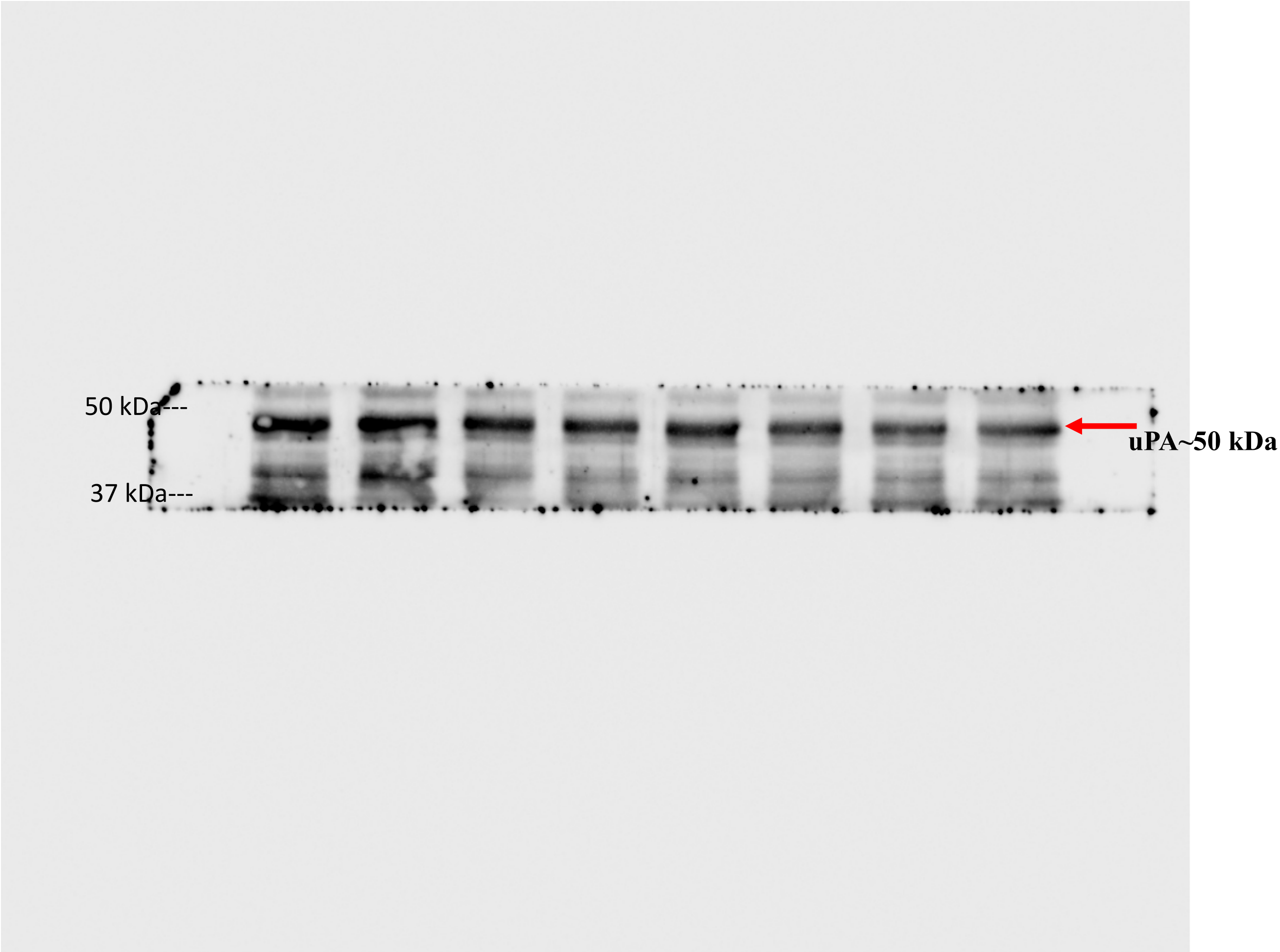

Figure S66. Uncropped PVDF membrane showing β-actin in T.T cells. The raw image corresponds to the cropped data presented in Figure 7C.

Note: The membrane was sectioned according to the expected molecular weights of the target proteins before antibody incubation to enable the detection of multiple proteins from the same membrane. Representative blot images from the corresponding membrane sections are shown.

β-actin of T.T cells (lane 1: NoLPS, lane 2: LPS-PG, lane 3: LPS-PG + PTX3, lane 4: LPS-PG + PTX6, lane 5: LPS-PG + PTX9, lane 6: LPS-PG + PTX3 + EIF25, lane 7: LPS-PG + PTX3 + EIF50, lane 8: LPS-PG + PTX3 + EIF75)

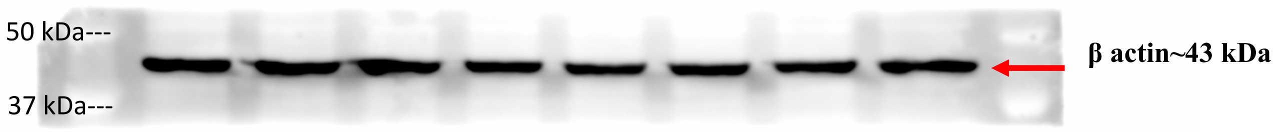

Figure S67. Uncropped PVDF membrane showing VEGFA in T.T cells. The raw image corresponds to the cropped data presented in Figure 7C.

Note: The membrane was sectioned according to the expected molecular weights of the target proteins before antibody incubation to enable the detection of multiple proteins from the same membrane. Representative blot images from the corresponding membrane sections are shown.

VEGFA of T.T cells (lane 1: NoLPS, lane 2: LPS-PG, lane 3: LPS-PG + PTX3, lane 4: LPS-PG + PTX6, lane 5: LPS-PG + PTX9, lane 6: LPS-PG + PTX3 + EIF25, lane 7: LPS-PG + PTX3 + EIF50, lane 8: LPS-PG + PTX3 + EIF75)

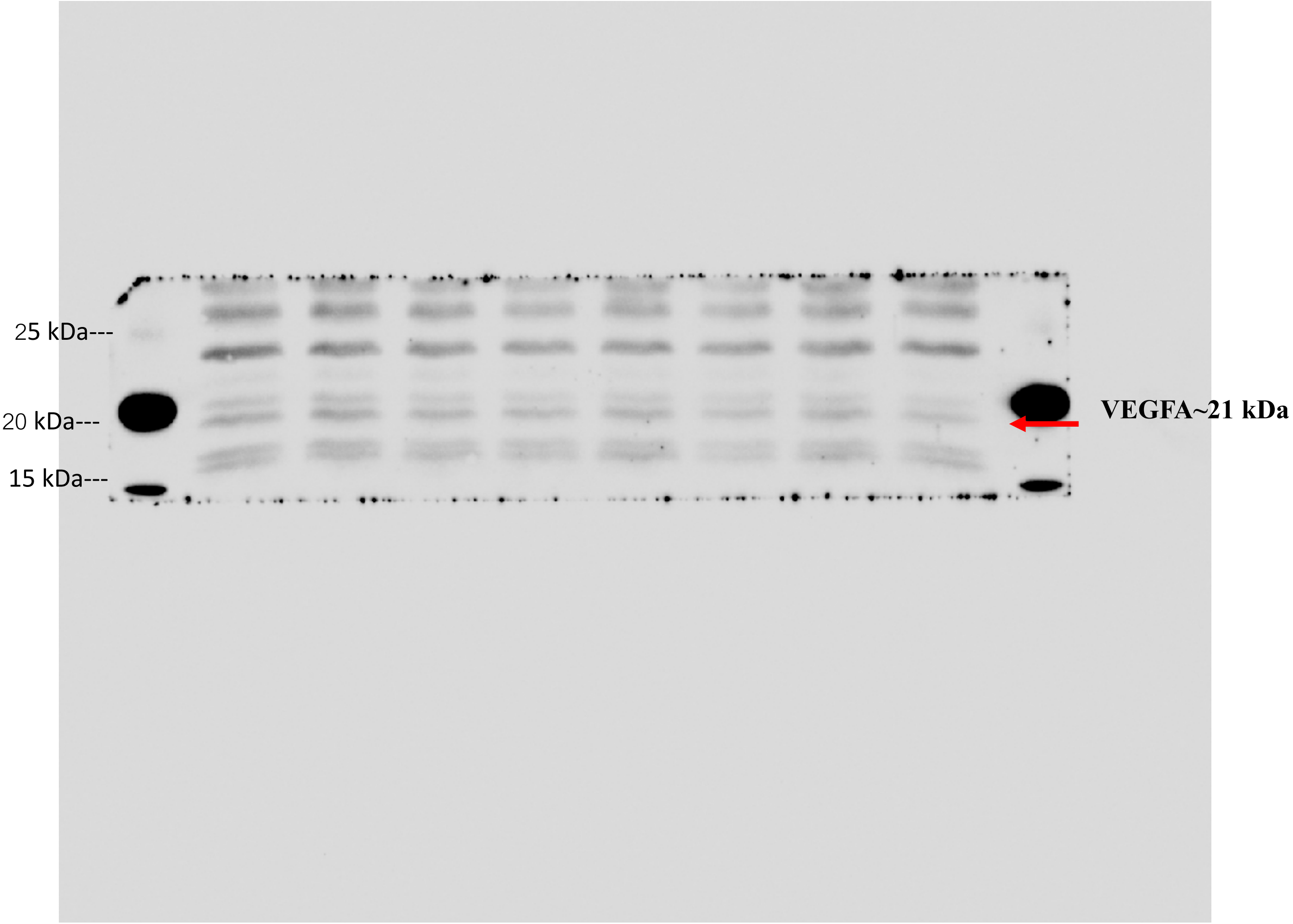

Figure S68. Uncropped PVDF membrane showing β-actin in T.T cells. The raw image corresponds to the cropped data presented in Figure 7C.

Note: The membrane was sectioned according to the expected molecular weights of the target proteins before antibody incubation to enable the detection of multiple proteins from the same membrane. Representative blot images from the corresponding membrane sections are shown.

β-actin of T.T cells (lane 1: NoLPS, lane 2: LPS-PG, lane 3: LPS-PG + PTX3, lane 4: LPS-PG + PTX6, lane 5: LPS-PG + PTX9, lane 6: LPS-PG + PTX3 + EIF25, lane 7: LPS-PG + PTX3 + EIF50, lane 8: LPS-PG + PTX3 + EIF75)

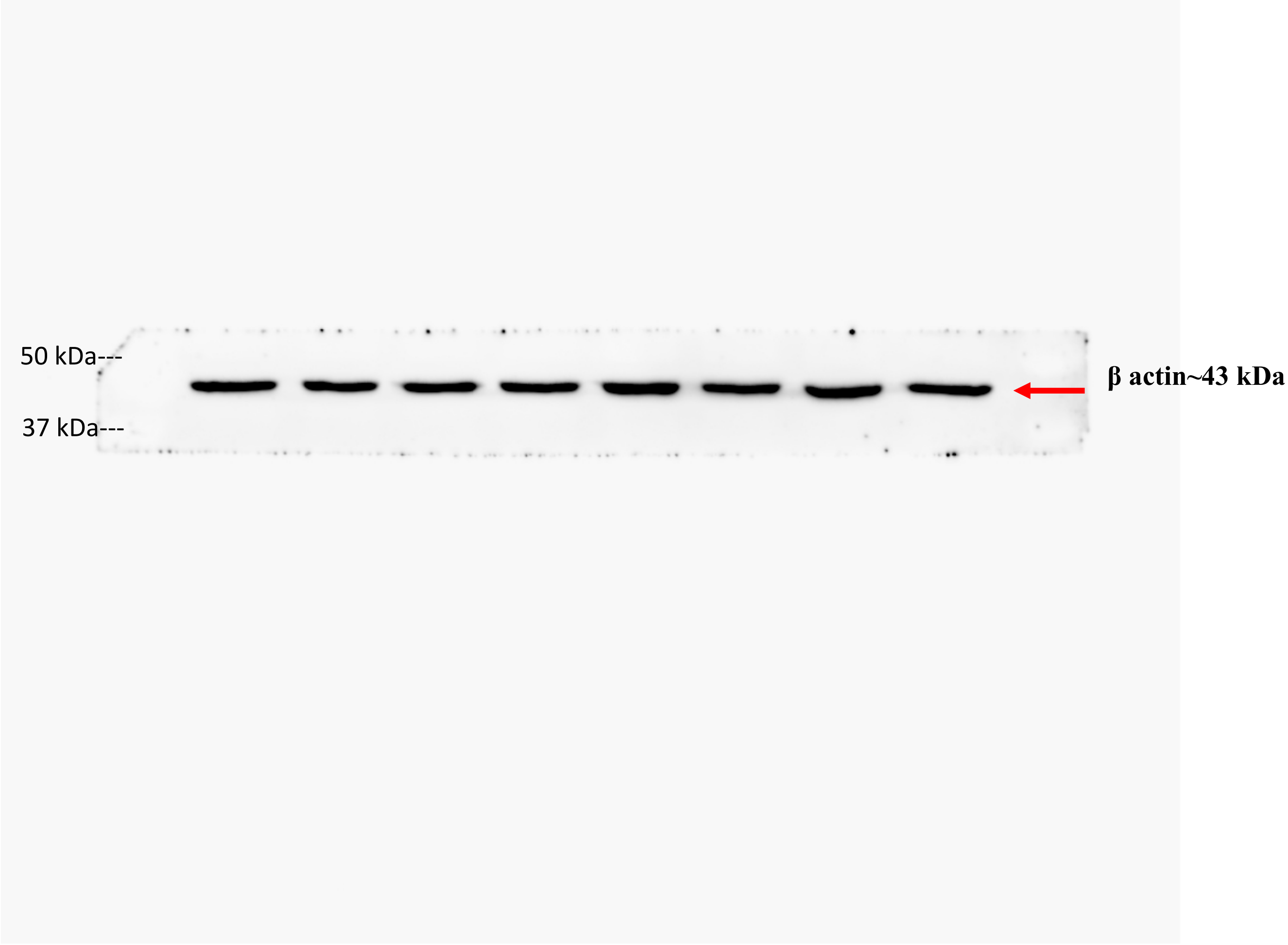

Figure S69. Uncropped PVDF membrane showing VEGFC in T.T cells. The raw image corresponds to the cropped data presented in Figure 7C.

Note: The membrane was sectioned according to the expected molecular weights of the target proteins before antibody incubation to enable the detection of multiple proteins from the same membrane. Representative blot images from the corresponding membrane sections are shown.

VEGFC of T.T cells (lane 1: NoLPS, lane 2: LPS-PG, lane 3: LPS-PG + PTX3, lane 4: LPS-PG + PTX6, lane 5: LPS-PG + PTX9, lane 6: LPS-PG + PTX3 + EIF25, lane 7: LPS-PG + PTX3 + EIF50, lane 8: LPS-PG + PTX3 + EIF75)

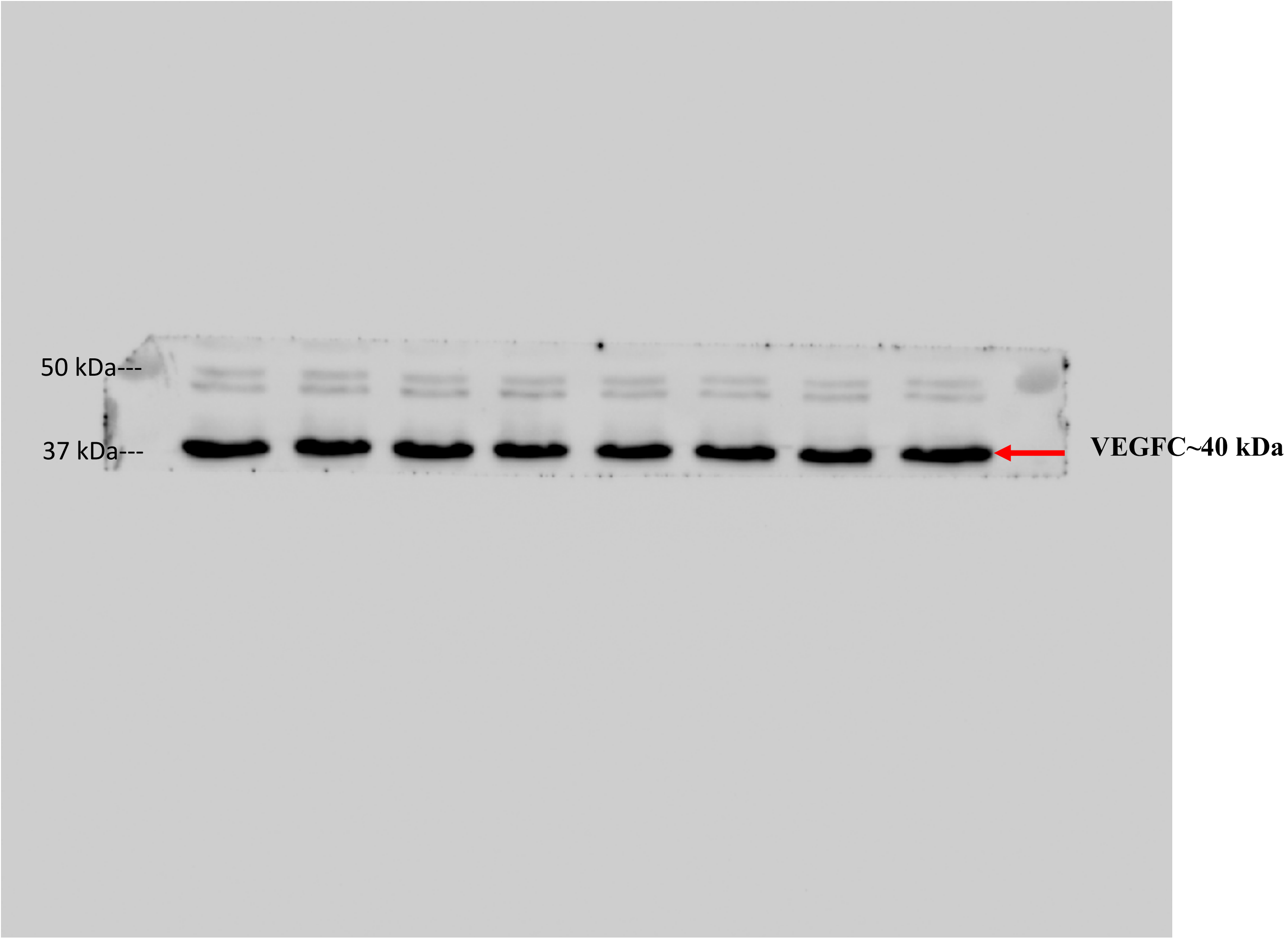

Figure S70. Uncropped PVDF membrane showing β-actin in T.T cells. The raw image corresponds to the cropped data presented in Figure 7C.

Note: The membrane was sectioned according to the expected molecular weights of the target proteins before antibody incubation to enable the detection of multiple proteins from the same membrane. Representative blot images from the corresponding membrane sections are shown.

β-actin of T.T cells (lane 1: NoLPS, lane 2: LPS-PG, lane 3: LPS-PG + PTX3, lane 4: LPS-PG + PTX6, lane 5: LPS-PG + PTX9, lane 6: LPS-PG + PTX3 + EIF25, lane 7: LPS-PG + PTX3 + EIF50, lane 8: LPS-PG + PTX3 + EIF75)

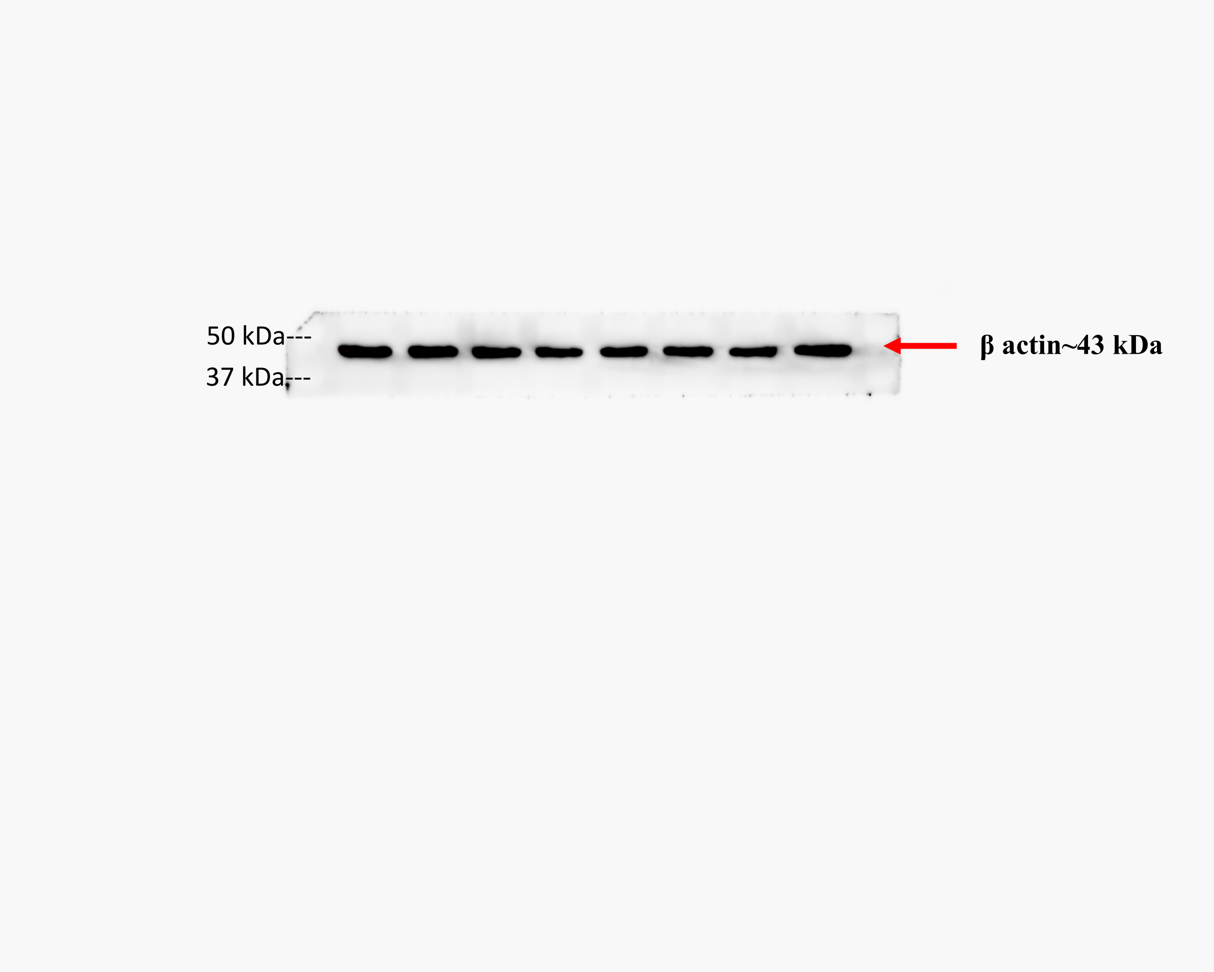

Figure S71. Uncropped PVDF membrane showing pp65 in T.T cells. The raw image corresponds to the cropped data presented in Figure 7L.

Note: The membrane was sectioned according to the expected molecular weights of the target proteins before antibody incubation to enable the detection of multiple proteins from the same membrane. Representative blot images from the corresponding membrane sections are shown.

pp65 of T.T cells (lane 1: NoLPS, lane 2: LPS-PG, lane 3: LPS-PG + PTX3, lane 4: LPS-PG + PTX6, lane 5: LPS-PG + PTX9, lane 6: LPS-PG + PTX3 + EIF25, lane 7: LPS-PG + PTX3 + EIF50, lane 8: LPS-PG + PTX3 + EIF75)

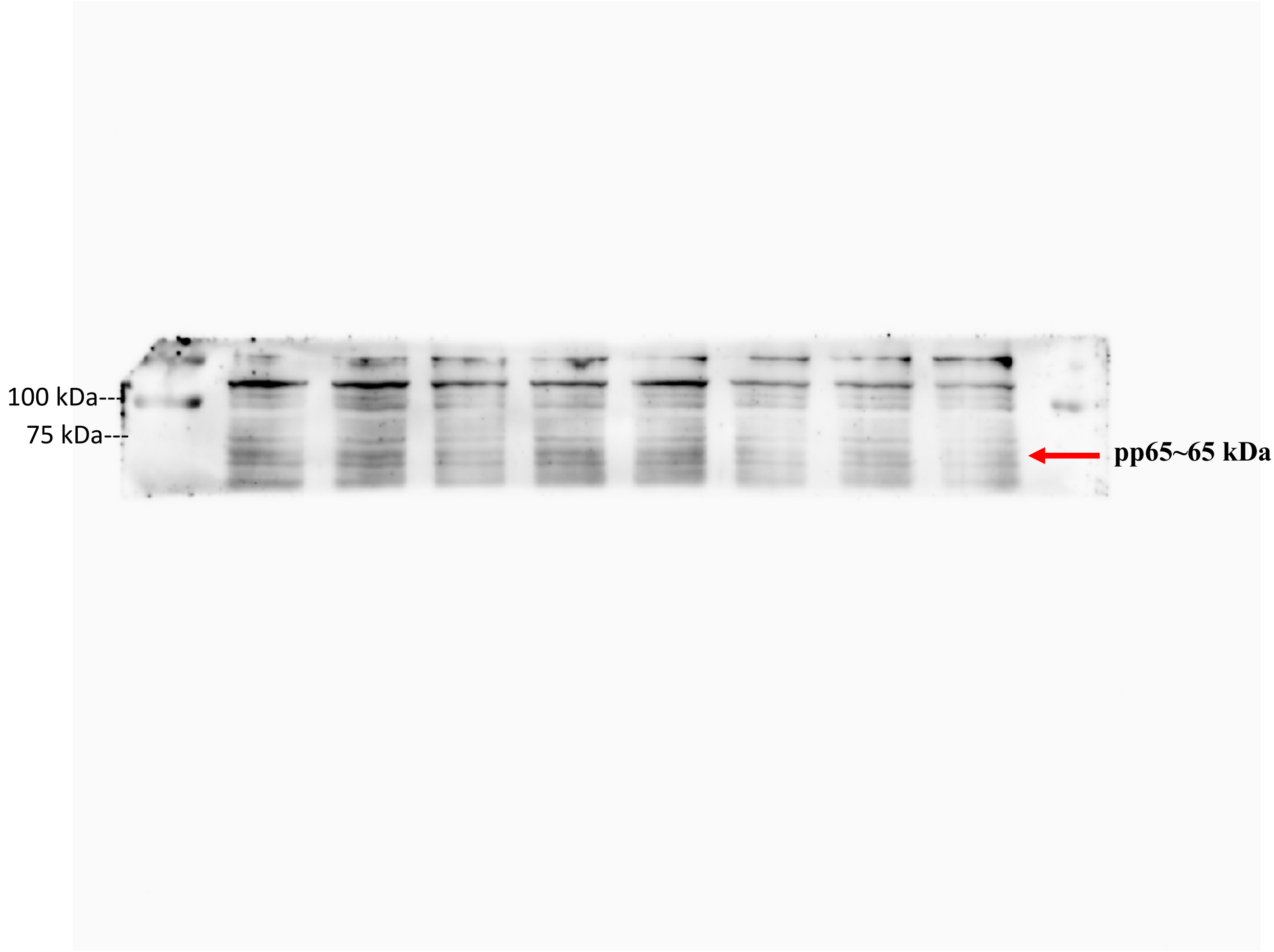

Figure S72. Uncropped PVDF membrane showing β-actin in T.T cells. The raw image corresponds to the cropped data presented in Figure 7L.

Note: The membrane was sectioned according to the expected molecular weights of the target proteins before antibody incubation to enable the detection of multiple proteins from the same membrane. Representative blot images from the corresponding membrane sections are shown.

β-actin of T.T cells (lane 1: NoLPS, lane 2: LPS-PG, lane 3: LPS-PG + PTX3, lane 4: LPS-PG + PTX6, lane 5: LPS-PG + PTX9, lane 6: LPS-PG + PTX3 + EIF25, lane 7: LPS-PG + PTX3 + EIF50, lane 8: LPS-PG + PTX3 + EIF75)

The identical β-actin band was utilized as a shared loading control for pp65 and p65 of T.T cells, which were analyzed on the same membrane using cutting and stripping/reprobing methods to ensure accurate normalization.

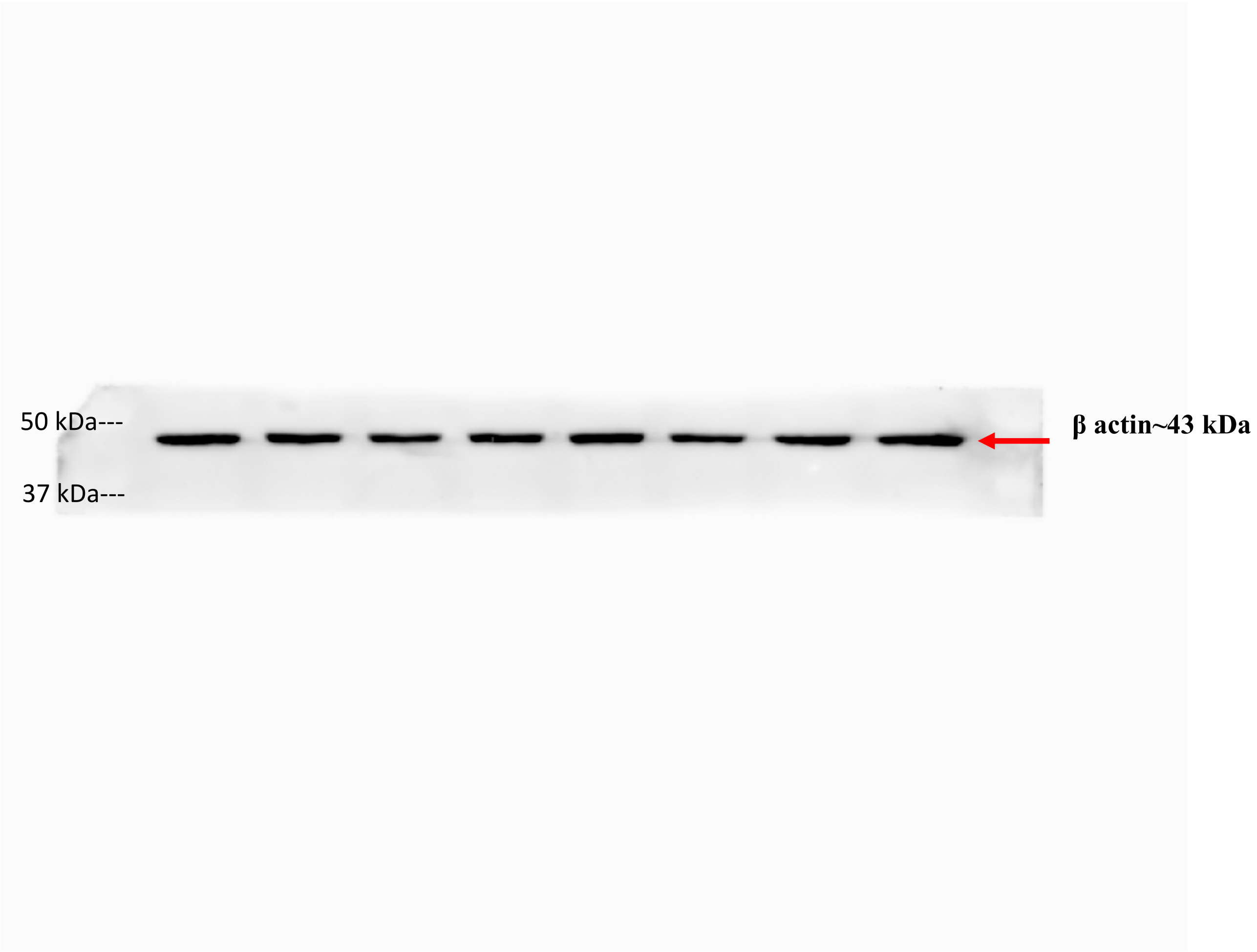

Figure S73. Uncropped PVDF membrane showing p65 in T.T cells. The raw image corresponds to the cropped data presented in Figure 7L.

Note: The membrane was sectioned according to the expected molecular weights of the target proteins before antibody incubation to enable the detection of multiple proteins from the same membrane. Representative blot images from the corresponding membrane sections are shown.

p65 of T.T cells (lane 1: NoLPS, lane 2: LPS-PG, lane 3: LPS-PG + PTX3, lane 4: LPS-PG + PTX6, lane 5: LPS-PG + PTX9, lane 6: LPS-PG + PTX3 + EIF25, lane 7: LPS-PG + PTX3 + EIF50, lane 8: LPS-PG + PTX3 + EIF75)

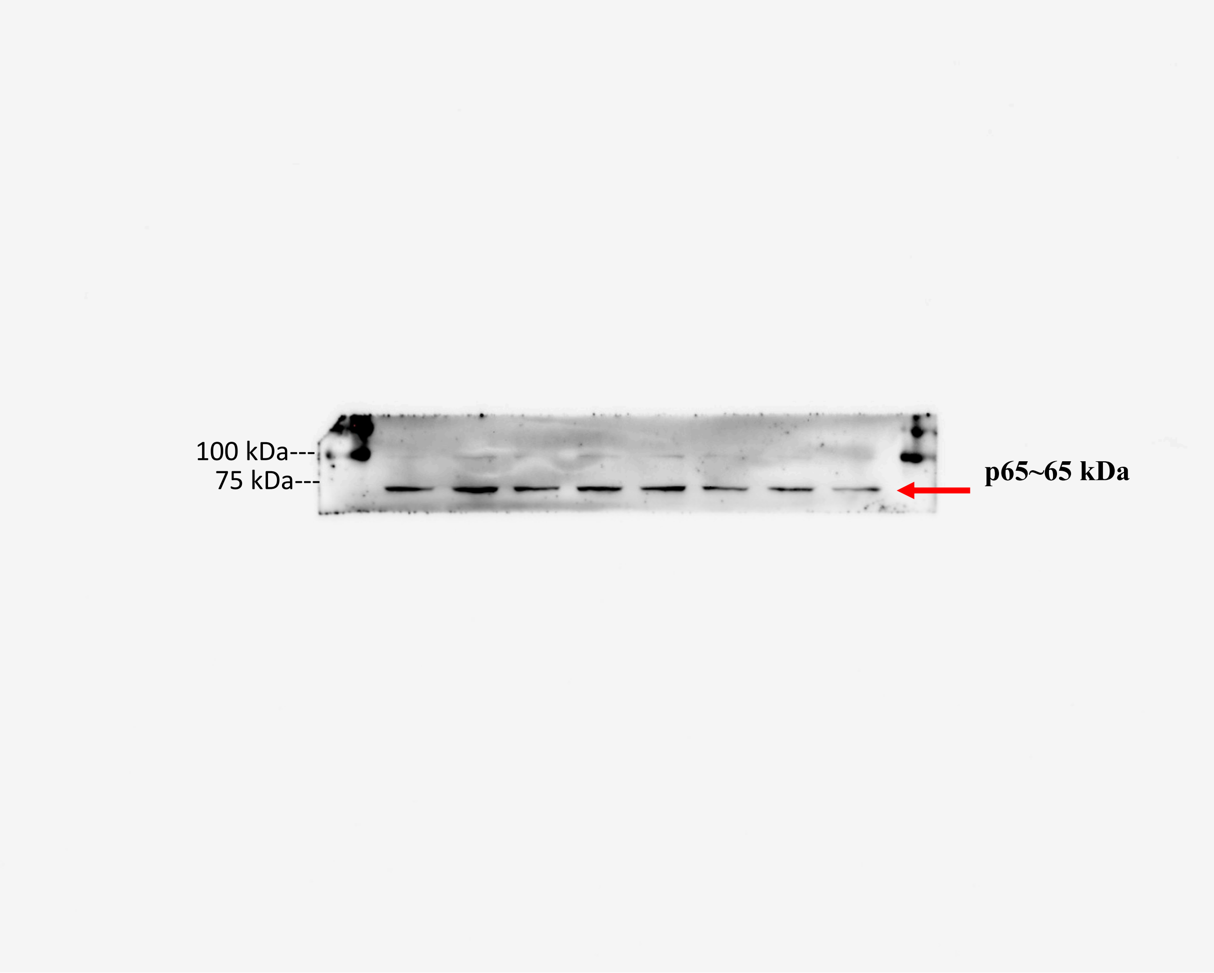

Figure S74. Uncropped PVDF membrane showing β-actin in T.T cells. The raw image corresponds to the cropped data presented in Figure 7L.

Note: The membrane was sectioned according to the expected molecular weights of the target proteins before antibody incubation to enable the detection of multiple proteins from the same membrane. Representative blot images from the corresponding membrane sections are shown.

β-actin of T.T cells (lane 1: NoLPS, lane 2: LPS-PG, lane 3: LPS-PG + PTX3, lane 4: LPS-PG + PTX6, lane 5: LPS-PG + PTX9, lane 6: LPS-PG + PTX3 + EIF25, lane 7: LPS-PG + PTX3 + EIF50, lane 8: LPS-PG + PTX3 + EIF75)

The identical β-actin band was utilized as a shared loading control for pp65 and p65 of T.T cells, which were analyzed on the same membrane using cutting and stripping/reprobing methods to ensure accurate normalization.

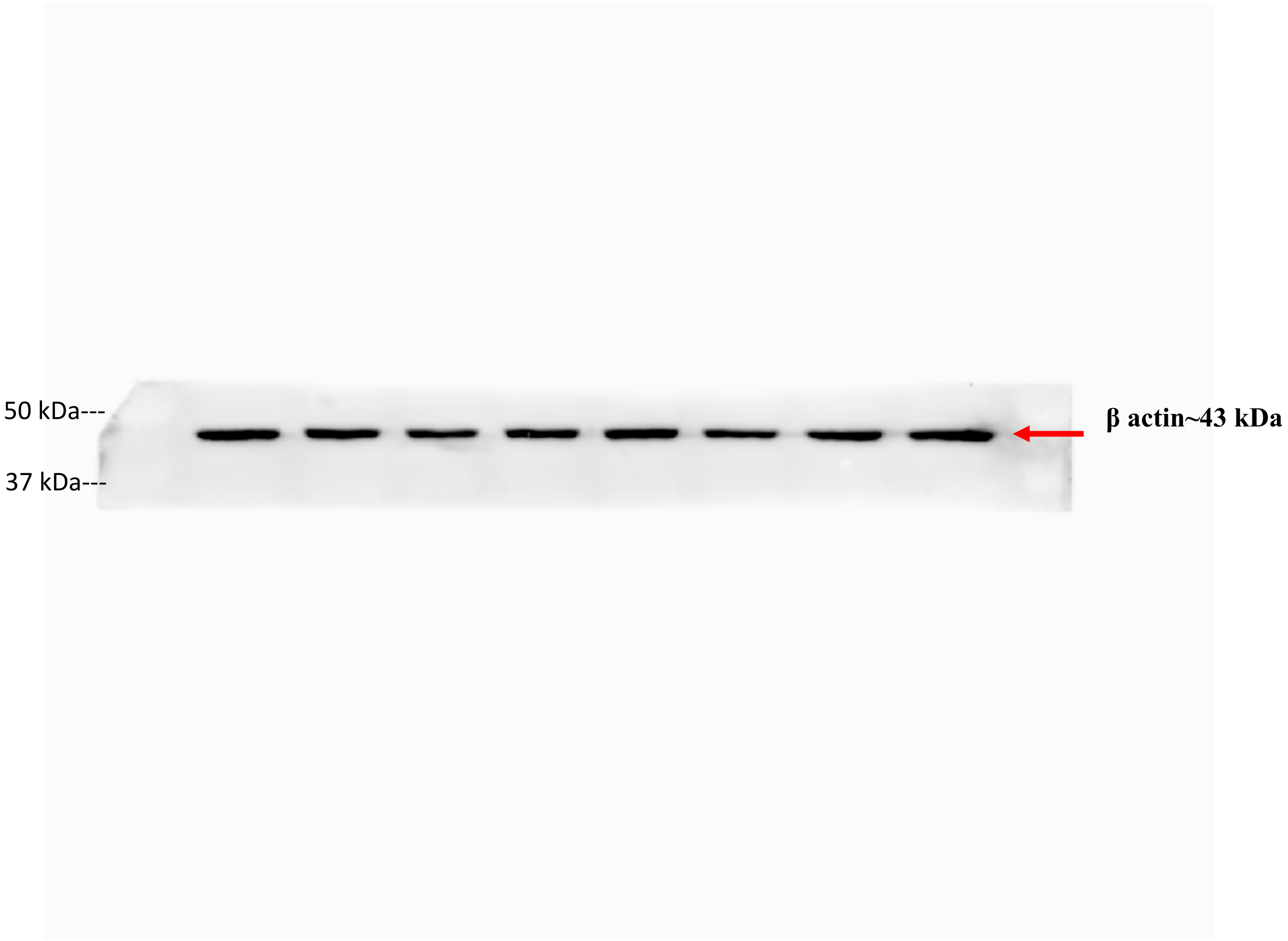

Figure S75. Uncropped PVDF membrane showing pp38 in T.T cells. The raw image corresponds to the cropped data presented in Figure 7L.

Note: The membrane was sectioned according to the expected molecular weights of the target proteins before antibody incubation to enable the detection of multiple proteins from the same membrane. Representative blot images from the corresponding membrane sections are shown.

pp38 of T.T cells (lane 1: NoLPS, lane 2: LPS-PG, lane 3: LPS-PG + PTX3, lane 4: LPS-PG + PTX6, lane 5: LPS-PG + PTX9, lane 6: LPS-PG + PTX3 + EIF25, lane 7: LPS-PG + PTX3 + EIF50, lane 8: LPS-PG + PTX3 + EIF75)

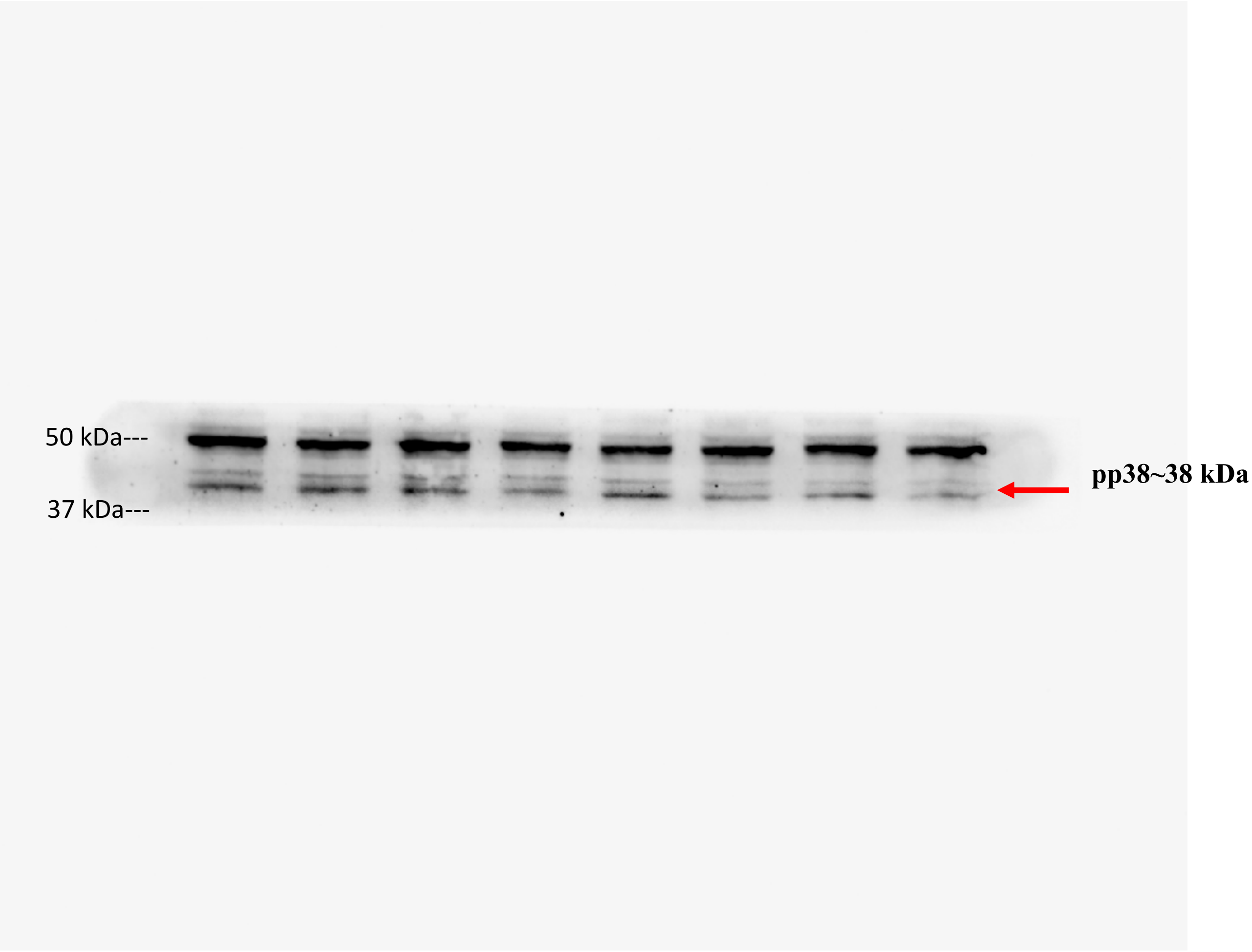

Figure S76. Uncropped PVDF membrane showing β-actin in T.T cells. The raw image corresponds to the cropped data presented in Figure 7L.

Note: The membrane was sectioned according to the expected molecular weights of the target proteins before antibody incubation to enable the detection of multiple proteins from the same membrane. Representative blot images from the corresponding membrane sections are shown.

β-actin of T.T cells (lane 1: NoLPS, lane 2: LPS-PG, lane 3: LPS-PG + PTX3, lane 4: LPS-PG + PTX6, lane 5: LPS-PG + PTX9, lane 6: LPS-PG + PTX3 + EIF25, lane 7: LPS-PG + PTX3 + EIF50, lane 8: LPS-PG + PTX3 + EIF75)

The identical β-actin band was utilized as a shared loading control for uPA, pp38 and p38 of T.T cells , which were analyzed on the same membrane using cutting and stripping/reprobing methods to ensure accurate normalization.

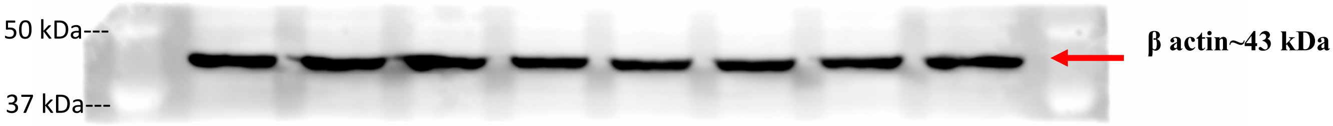

Figure S77. Uncropped PVDF membrane showing p38 in T.T cells. The raw image corresponds to the cropped data presented in Figure 7L.

Note: The membrane was sectioned according to the expected molecular weights of the target proteins before antibody incubation to enable the detection of multiple proteins from the same membrane. Representative blot images from the corresponding membrane sections are shown.

p38 of T.T cells (lane 1: NoLPS, lane 2: LPS-PG, lane 3: LPS-PG + PTX3, lane 4: LPS-PG + PTX6, lane 5: LPS-PG + PTX9, lane 6: LPS-PG + PTX3 + EIF25, lane 7: LPS-PG + PTX3 + EIF50, lane 8: LPS-PG + PTX3 + EIF75)

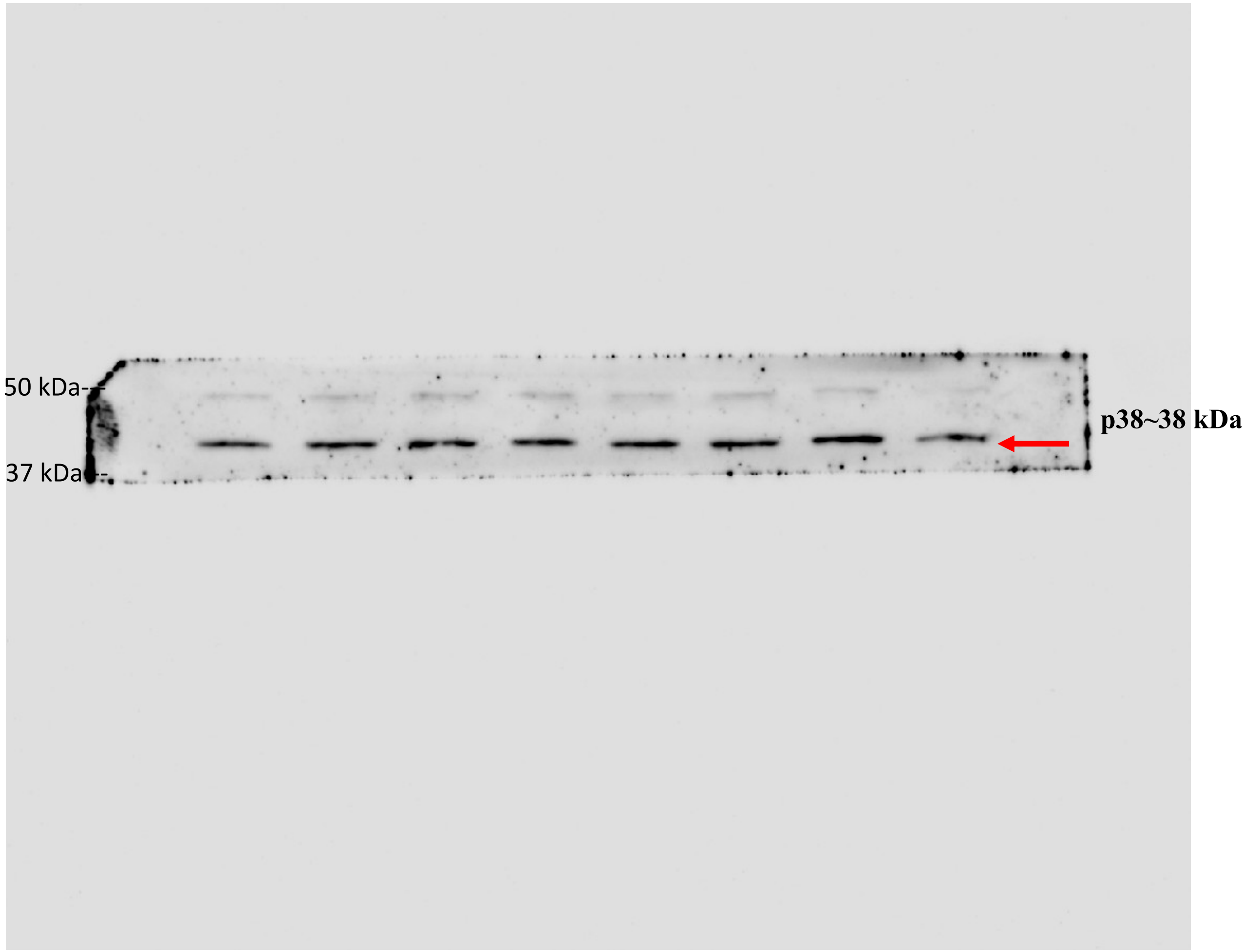

Figure S78. Uncropped PVDF membrane showing β-actin in T.T cells. The raw image corresponds to the cropped data presented in Figure 7L.

Note: The membrane was sectioned according to the expected molecular weights of the target proteins before antibody incubation to enable the detection of multiple proteins from the same membrane. Representative blot images from the corresponding membrane sections are shown.

β-actin of T.T cells (lane 1: NoLPS, lane 2: LPS-PG, lane 3: LPS-PG + PTX3, lane 4: LPS-PG + PTX6, lane 5: LPS-PG + PTX9, lane 6: LPS-PG + PTX3 + EIF25, lane 7: LPS-PG + PTX3 + EIF50, lane 8: LPS-PG + PTX3 + EIF75)

The identical β-actin band was utilized as a shared loading control for uPA, pp38 and p38 of T.T cells, which were analyzed on the same membrane using cutting and stripping/reprobing methods to ensure accurate normalization.

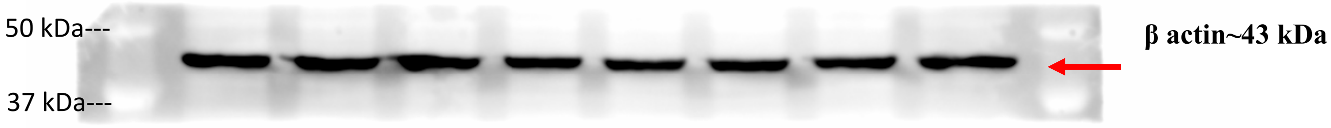

Supplement: Supplementary file 1 [file ijms-27-09464-s001.zip › ijms-4609922-supplementary.pdf]
